# Spontaneous Peptide Ligation Mediated by Cysteamine

**DOI:** 10.1021/jacsau.4c00243

**Published:** 2024-04-29

**Authors:** Abid Barat, Matthew W. Powner

**Affiliations:** † Department of Chemistry, University College London, 20 Gordon Street, London WC1H 0AJ, United Kingdom

**Keywords:** cysteamine, nitrile, peptide, prebiotic, Strecker

## Abstract

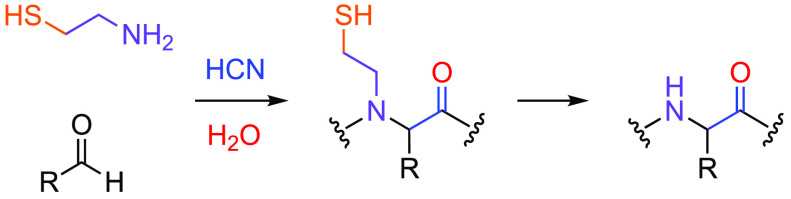

The fundamental and universal nature of life’s exploitation of peptides suggests they must have played a vital role during the onset of life, but their spontaneous chemoselective synthesis in water remains unknown. Aminonitriles (**1**) are widely accepted as prebiotic precursors of both amino acids and peptides, but they do not spontaneously polymerize in water to yield peptides. Here, we demonstrate that the simple prebiotically plausible aminothiol, cysteamine (**5**), participates in Strecker chemistry to furnish β-mercaptoethyl-α-aminonitriles (**8**) and β-mercaptoethyl-amino acids (**16**), which are predisposed to spontaneously form peptides in water. Intramolecular thiol catalyzed ligation is faster, higher-yielding, and more α-selective than previously reported prebiotic peptide ligation chemistries, enabling, for example, the highly regioselective α-ligation of Asp- and Glu-dinitriles in quantitative yields. Our findings suggest that cysteamine (**5**), the thiol bearing moiety of the universal thiol cofactor coenzyme A, may have played an important role in the selective chemical synthesis of prebiotic α-peptides.

The universal genetic code establishes that peptide biosynthesis predated life’s last universal common ancestor;^1−5^ however, peptide biosynthesis is highly evolved^1−7^ and could not spontaneously appear in its current form at the onset of life. Therefore, peptide biosynthesis must have been preceded by nonenzymatic peptide syntheses during the early stages of evolution;^8^ however, the nature of prebiotic peptide synthesis remains elusive despite decades of research. Particularly, the spontaneous, chemoselective formation of prebiotic α-peptides in neutral water has not been demonstrated.

Chemical strategies for peptide synthesis rely upon monomer activation and coupling to the *N*-terminal amine of a growing peptide.^9−11^ However, interestingly, this stands in stark contrast to biosynthesis; both ribosomal and nonribosomal peptide syntheses proceed in the opposite direction, from the *N*- to *C*-terminus, and both rely upon activation of the growing peptide chain toward nucleophilic addition of the monomer amine.^6,12−14^ This observation suggests that there may be important clues in the direction and strategy of peptide biosynthesis that would facilitate prebiotic peptide synthesis.

Seeking to explore the advantages of the biomimetic strategy, we have previously recognized that aminonitriles **1** and peptide nitriles **2** are thermodynamically activated but kinetically stable substrates for prebiotic peptide synthesis (Figure 1).^15−19^ Coupling these nitriles, through transformation into thioacids **3** or thioimidates **4** (Figure 1B), allows efficient protecting-group-free peptide synthesis in water. However, despite these advances, the spontaneous synthesis of peptides remains an elusive goal with direct ligation of α-aminonitriles yielding amino-imidazoles that block peptide synthesis (Figure 1A).^20^

**Figure 1:**
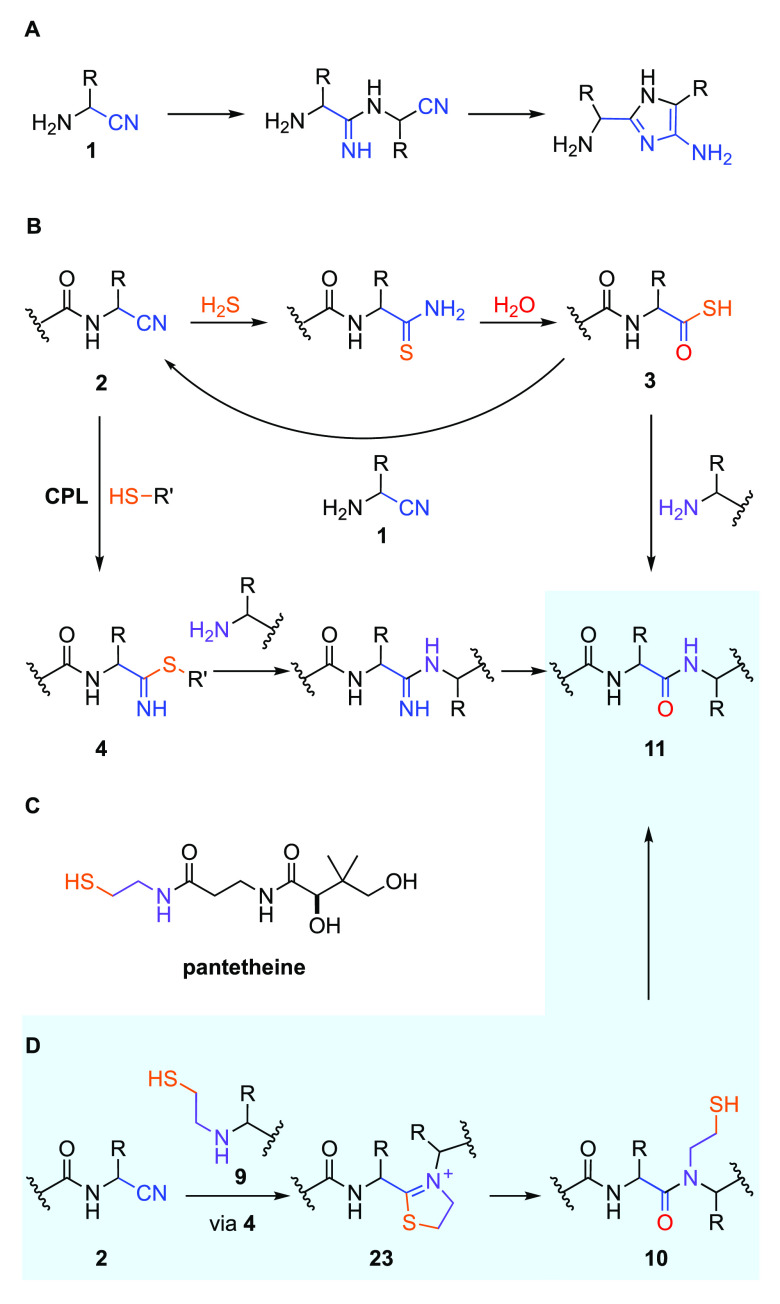
(A) Direct aminonitrile ligation would yield aminoimidazoles rather than peptides. (B) Previous work: Prebiotic nitrile mediated peptide ligations. (C) Previous work: Prebiotic synthesis of pantetheine. (D) This work: Intramolecular thiol-catalyzed ligation of peptide-nitriles and reductive fragmentation yielded peptide **11**. R = amino acid side chain; R′ = alkyl.

We recently demonstrated a chemoselective prebiotic synthesis of pantetheine (Figure 1C),^20^ the functional fragment of the universal cofactor, coenzyme A, which is essential throughout all domains of life as an acyl-carrier.^13,14^ The deep-seated role of pantetheine in biochemistry, for example in the Krebs cycle, fatty acid, and polyketide syntheses, has suggested that thiols may have played a key role in prebiotic chemistry, the “Thioester World” model for the origins of life.^21−23^ Importantly, in the context of peptides, pantetheine is required in nonribosomal protein modules to generate active thioesters for peptide elongation, and it has been previously proposed that this mode of peptide synthesis may predate ribosomal peptide synthesis during the evolution of life.^21^ Therefore, our prebiotic pantetheine synthesis opens exciting questions about the role of pantetheine and its precursor, cysteamine (**5**),^20,24−27^ in prebiotic peptide synthesis. Specifically, whether **5** could play a role in developing and directing a mechanism for spontaneous peptide synthesis in water that would prevent amino-imidazole formation and foreshadow its incorporation into nonribosomal peptide synthesis as a component of pantetheine.

We reasoned that if **5** (or it is oxidized disulfide, cystamine **6**) were to participate in Strecker synthesis, with hydrogen cyanide (HCN) and an aldehyde **7**, the reaction would afford β-mercaptoethyl-aminonitrile **8** (Figure 2), where a thiol catalyst, a nucleophilic amine,^28^ and an electrophilic nitrile are built into one molecule. We anticipated that tethering a thiol-catalyst, required for catalytic peptide ligation (CPL; Figure 1B),^17,18^ to the amine coupling partner (**9**) would greatly enhance the rate of peptide ligation and importantly would lead to spontaneous peptide ligation in near-neutral water (Figure 1D).^15−19^ We also recognize that the thiol-tethered ligation products would be β-mercaptoethyl-peptoids **10**, which undergo reductive fragmentation to yield proteinogenic peptides **11** (Figure 1D).^29−31^

**Figure 2:**
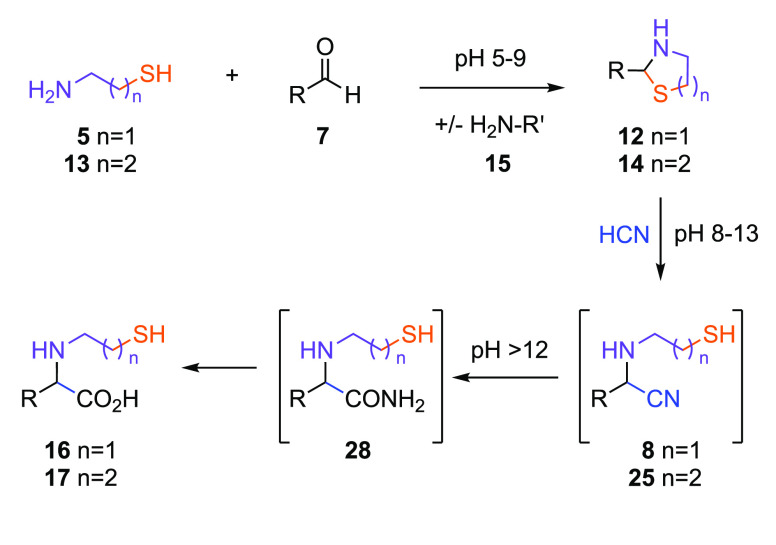
Strecker synthesis of mercaptoalkyl-aminonitriles and mercaptoalkyl-amino acids in water. The reaction of aldehyde **7** and aminothiol (**5** or **13**) yields thiazolidine **12** or thiazinane **14**, which react with HCN to yield β-mercaptoethyl-aminonitrile **8** or γ-mercaptopropyl-aminonitrile **25**. Hydrolysis of β-mercaptoethyl-aminonitrile **8** or γ-mercaptopropyl-aminonitrile **25** is observed to yield amino acid **16** or **17**, respectively, under alkaline conditions. R = amino acid side chain; R′ = H, alkyl, CH_2_CH_2_OH, or CH_2_CH_2_NH_2_.

To begin our investigation, we first incubated cysteamine (**5**) and formaldehyde (**7a**) in water. We observed quantitative formation of thiazolidine **12a** (R = H) across a broad pH range (pH 5–9; Figure 2; Supplementary Figures 1–4). Incubating aldehyde **7a** with a mixture of aminothiols (**5** and **13**) yielded a mixture of thiazolidine **12a** and thiazinane **14a** (R = H; Supplementary Figure 5). However, selective formation of thiazolidine **12a** was observed in equimolar competition with other amines, including ammonia **15a** (R′ = H), ethylamine **15b** (R′ = Et), ethanolamine **15c** (R′ = CH_2_CH_2_OH), and ethylene diamine **15d** (R′ = CH_2_CH_2_NH_2_) (Figure 2; Supplementary Figures 6–9), demonstrating the selective association of aminothiols with aldehydes that is required for Strecker chemistry to furnish mercaptoalkyl-amino acids.

The addition of HCN to thiazolidine **12a**, at pH 8–10, led to the formation of a heterogeneous precipitate, which underwent acid catalyzed hydrolysis to yield mercaptoethyl-amino acid **16**_**G**_ (R = H; 55%; Supplementary Figure 11). Hydrolysis of the precipitate to **16**_**G**_ implicated the in situ Strecker synthesis of aminonitrile **8**_**G**_, and its reaction onward by CPL. CPL is curtailed at highly alkaline pH,^18^ therefore we next investigated the Strecker reaction of thiazolidine **12** at pH 13, where we expected precipitate formation would be avoided if the reaction proceeded through CPL. As anticipated at pH 13, the addition of HCN to **12a** directly yielded glycine **16**_**G**_ (65%) (Figure 2; Supplementary Figure 12). Under the same conditions, thiazolidine **12b** (R = Me) yielded alanine **16**_**A**_ (64%) and thiazinane **14a** yielded glycine **17**_**G**_ (62%; Figure 2; Supplementary Figures 13–20). While unlikely to be prebiotic, these high pH reactions demonstrate that the reaction of thiazolidine **12** with HCN proceeded via a Strecker reaction.

The Strecker reaction is widely considered to be the foremost pathway for prebiotic amino acid synthesis;^15,32^ however, α-ketoacids **18** play a role in amino acid biosynthesis.^33−35^ Therefore, we next incubated **18** with cysteamine (**5**) (Figure 3). Quantitative conversion of **18** to thiazolidine **19** was observed across a broad pH range (pH 5–9). Thiazolidine **19** formation was highly selective in competition with other amines **15** (Supplementary Figures 26–29).

**Figure 3:**
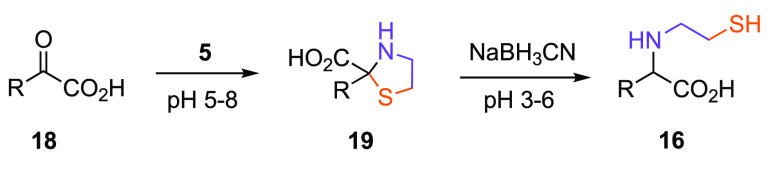
Synthesis of mercaptoalkyl-amino acid from α-keto acids in water. The reaction of α-ketoacid **18** and cysteamine **5** yields thiazolidine **19** that undergoes hydride reduction to yield amino acid **16**. R = amino acid side chain.

Reduction of thiazolidine **19** with NaBH_3_CN was observed across a range of acidic-to-neutral conditions (pH 3–6; Figure 3), for example furnishing glycine **16**_**G**_ (90%), alanine **16**_**A**_ (77%), and valine **16**_**V**_ (69%) in good-to-excellent yield. In the presence of ammonia (**15a**; 2 equiv), the reduction of **19a** (R = H) led to the selective formation of **16**_**G**_ (85%)—no glycine from reductive amination with ammonia (**15a**) was observed (Supplementary Figure 32), demonstrating the ambident nucleophilicity of **5** can drive the selective synthesis of β-mercaptoethyl-amino acids (**16**). However, these amino acids are not activated, and a prebiotic borohydride equivalent remains to be discovered; therefore, we returned our attention to nitrile chemistry.

Having observed the synthesis and spontaneous CPL reaction of aminonitrile **8**, we next investigated the Strecker reaction of cystamine (**6**). We suspected thiol-oxidation would render disulfide-aminonitrile **20** stable and block CPL-type nitrile reactions (Figure 4); however, we also recognized that disulfide **6** could be reduced by HCN,^36^ and if sufficiently fast, this reduction would block the synthesis of disulfide **20**. However, we suspected disulfide reduction would be inhibited by the formation of cyanohydrin **21**.^37−39^ Therefore, we were pleased to observe that incubating cyanohydrin **21a** (R = H) and cystamine (**6**) at pH 9.5 yielded disulfide **20**_**G**_ (50–60%; Supplementary Table 3). Demonstrating thiol oxidation (**5** → **6**) stabilized β-mercaptoethyl-aminonitriles (**8**) by curtailing the spontaneous CPL reaction that they are predisposed to undergo.

**Figure 4:**
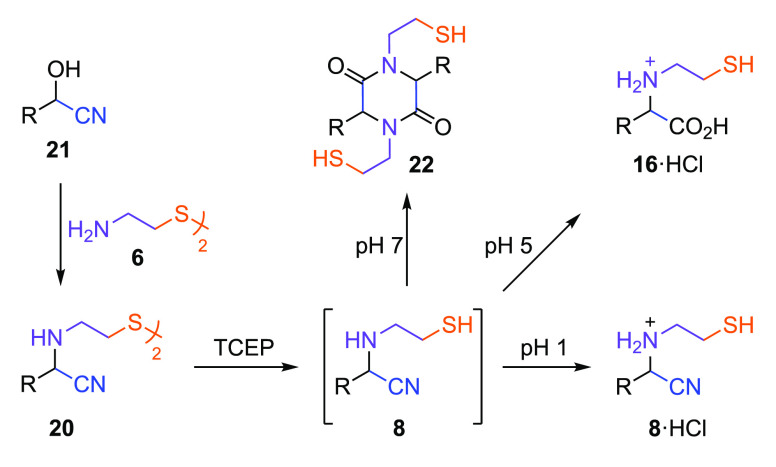
Reductive synthesis of mercaptoalkyl-aminonitrile in water. The reaction of **7** and HCN yields cyanohydrin **21**, which reacts with cystamine **6** to yield β,β′-disulfide-α,α′-dinitrile **20** under Strecker conditions, that undergoes disulfide reduction with tris(2-carboxyethyl)-phosphine (TCEP) to yield aminonitrile **8**. Aminonitrile **8** undergoes spontaneous ligation at pH 7 to yield peptoid **22** and spontaneous hydrolysis at pH 5 to yield amino acid **16**. (B) R = amino acid side chain.

Reduction of disulfide-dinitrile **20** was observed to yield aminonitrile **8** (>95%) across a broad pH range (Figure 4; Supplementary Figures 50–57); under all conditions, except extremely acidic conditions, **8** was observed to react spontaneously to yield secondary products. At acidic pH, the ligation of **8** was not observed; nitrile **8** was observed to spontaneously hydrolyze to amino acid **16** (80% after 7 days; Figure 4) at pH 5, likely via intramolecular thiol-catalyzed hydrolysis, and upon further acidification (pH 1), **8**·HCl was observed to be stable, for example yielding glycine **8**_**G**_·HCl (66%) or alanine **8**_**A**_·HCl (85%; Figure 4; Supplementary Figures 50, 51, 62, and 63). At neutral-to-alkaline pH (pH 7.0–9.5), aminonitrile **8** underwent spontaneous ligation; at neutral pH, ligation of nitrile **8**_**G**_ was observed to yield cyclic dipeptoid **22**_**GG**_ (65%) in good yield (Figure 4; Supplementary Figures 53–56). This demonstrates the ligation of **8** with a second equivalent of **8** to yield a dimeric peptoid, which remains activated at the *C*-terminus, resulting in cyclization to yield **22**_**GG**_. Prior studies of α-aminonitrile–nitrile ligation have yielded imidazoles, blocking amide bond synthesis (Figure 1A);^20^ however, the incorporation of cysteamine (**5**) into the aminonitrile, through Strecker reactions, leads to efficient peptide synthesis by aminonitrile coupling.

To further examine the efficacy of cysteamine-mediated nitrile ligation, we next investigated the reaction of peptide nitrile **2**_**G**_ with amino acid **16**_**G**_. After 24 h, at pH 7, this ligation furnished peptoid **10a** (>80%) as a mixture of two rotamers (Table 1; Supplementary Figures 64–66). The ambident nature of **16** resulted in rapid ligation with **2**, via the intramolecular reaction of thioimidate (**4**) with the tethered amine. The secondary amine moiety of **9** prevents tautomerization to a stable thiazoline,^40^ and so progresses through a transient thiazolinium **23** to furnish amide **10** (Figure 1D). The ligation was observed to be highly pH dependent (Table 1). At pH 5, only partial conversion to **10a** was observed in 24 h, whereas at pH 9 near quantitative formation of **10a** was observed within 3 h. The ligation of β-mercaptoethyl-amino acids (**16**) with a wide range of peptide-nitriles **2** resulted in high yields of peptoid **10** (Figure 5). Sterically demanding β-branched nitriles ligated slowly but afforded good-to-excellent yields of **10**.

**Table 1:**
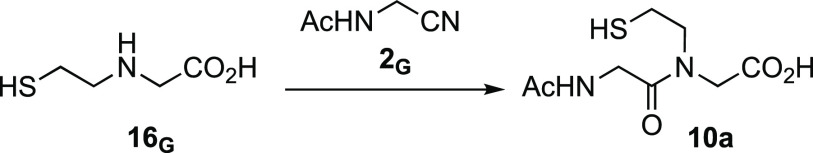
Ligation of Glycyl Nitrile with β-Mercaptoethyl Glycine

entry	**2**_**G**_/mM	**16**_**G**_/mM	T/°C	pH	buffer^a^	time/h	yield/%
**1**	115	100	r.t.	5	ABS	24	7
**2**	130	100	r.t.	7^b^		24	85
**3**	100	100	r.t.	7	PBS	24	84
**4**	100	100	25	7	PBS	15	79
**5**	100	100	40	7	PBS	15	90
**6**	100	100	60	7	PBS	15	91
**7**	110	100	20	9	PBS	3	>95

a Ligation of nitrile **2**_**G**_ and glycine **16**_**G**_ to form peptoid **10a**. 500 mM buffer solution; PBS = phosphate; ABS = acetate; BBS = borate. r.t. = room temperature.

b Final pH = 7.8.

**Figure 5:**
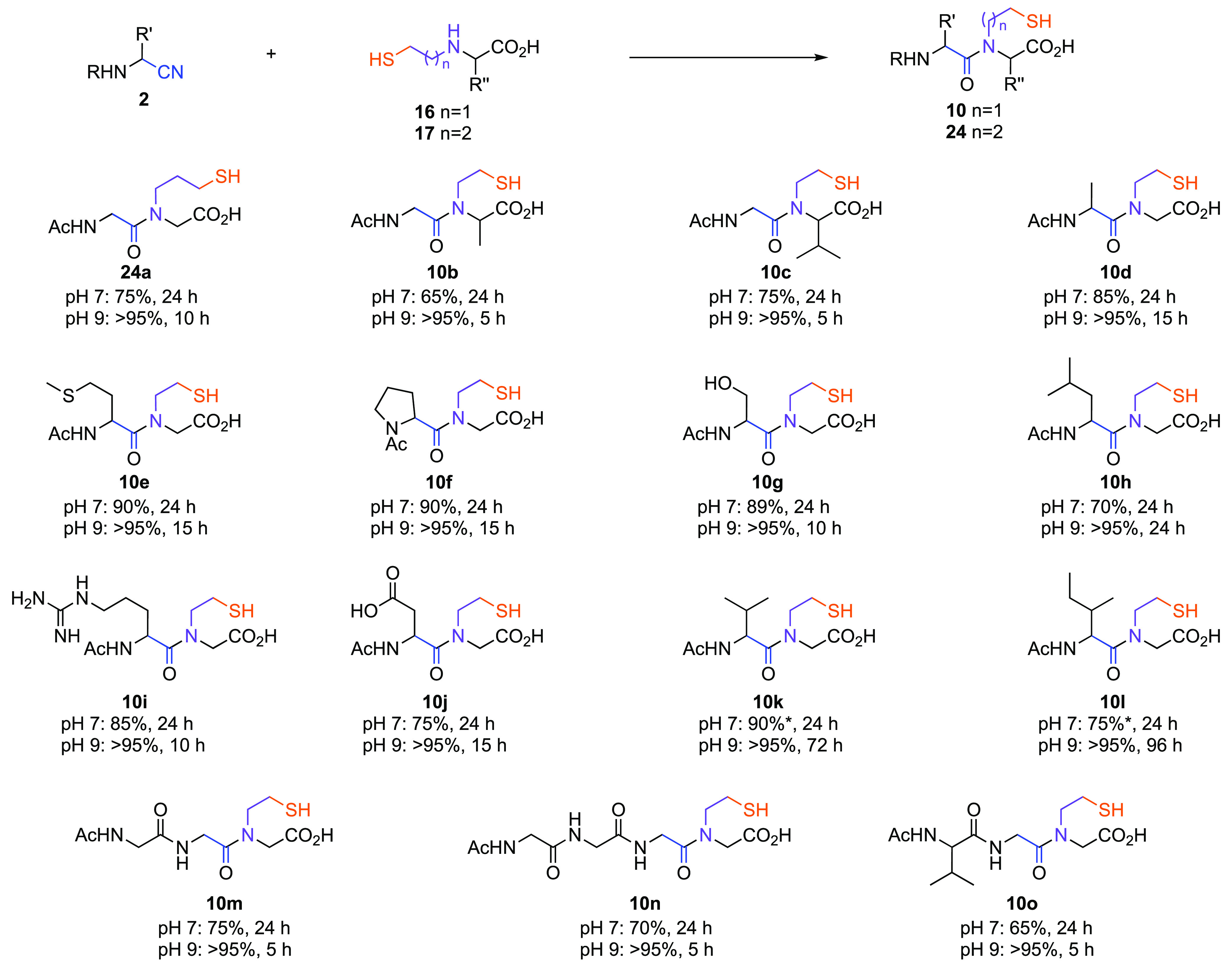
Ligation of peptide–nitriles with mercaptoalkyl-amino acids. Ligation of nitriles **2** (37.5–50 mM) with β-mercaptoethyl-amino acid **16** or γ-mercaptopropyl-amino acid **17** (1 equiv) in PBS (pH 7, 500 mM) or BBS (pH 9, 500 mM) at room temperature. *60 °C. R = acetyl or peptide. R′ and R″ = *rac*-amino acid side chain as indicated by subscript single letter code.

The ligation was strongly promoted by the five-atom intramolecular disposition^41^ of the amine and thioimidate moieties formed upon reaction of amino acid **16** with nitrile **2** but also achieved through a six-atom disposition (Figure 5; **2_G_** + **17a** → **24a**). However, incubating equimolar **16**_**G**_ and **17**_**G**_ with nitrile **2**_**G**_ selectively yielded **10a** as the major product (**10a**/**24a** 77:23; Supplementary Figure 88), demonstrating preferential ligation of amino acid **16**_**G**_ derived from cysteamine (**5**).

Next, to explore the selectivity of coupling amino acid **16** with proteinogenic α-amidonitriles, we incubated **16**_**G**_ with nonproteinogenic-nitriles. β-Alanine nitrile (**2**_**βA**_) and α-aminoisobutyric acid nitrile (**2**_**Aib**_) were both incubated with **16**_**G**_ and nitrile **2**_**G**_. Exclusive ligation of **16**_**G**_ with **2**_**G**_, forming **10a**, was observed (Figure 6; Supplementary Figures 131–133), demonstrating that the α-amide selectivity observed in nitrile-activated CPL,^17^ is retained with intramolecular thiol-catalysis. We next investigated the reaction of the dinitriles. Incubating α,β-dinitrile **2**_**EX**_ or α,γ-dinitrile **2**_**DX**_ with **16**_**G**_ led to exclusive ligation through the α-nitrile (Figure 6C; Supplementary Figures 134, 135, 139, and 140). Moreover, upon further incubation of β-nitrile **10q** (up to 2 days) with excess **16**_**G**_, we did not observe β-nitrile ligation. This remarkable selectivity differentiates α-nitriles from longer homologues; it is also of note that α-ligation, in **2**_**DX**_, is significantly accelerated by the electron-withdrawing effect of the β-nitrile (Supplementary Figures 137 and 138).

**Figure 6:**
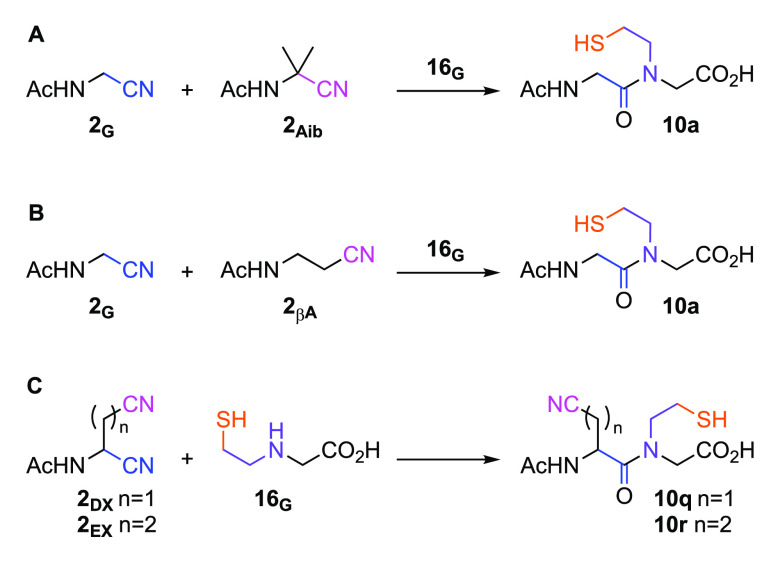
Selective ligation of α-amidonitriles with β-mercaptoethyl-glycine. (A) The reaction of **2**_**G**_ (100 mM), **16**_**G**_ (100 mM), and **2**_**Aib**_ (100 mM) in PBS (pH 7, 500 mM) yields **10a** (74%) after 24 h. (B). The reaction of **2**_**G**_ (100 mM), **16**_**G**_ (100 mM), and **2**_**βA**_ (100 mM) in PBS (pH 7, 500 mM) yields **10a** (87%) after 24 h. (C) The reaction **2**_**DX**_ (50 mM) and **2**_**EX**_ (50 mM) with **16**_**G**_ (50 mM) in PBS (pH 7, 500 mM) yields **10q** (>95%) and **10r** (>95%) after 1.5 and 10 h, respectively.

Finally, having recognized that fragmentation of peptoid **10** would furnish α-peptide **11** (Figure 1D),^29,30^ we incubated **10a** with TCEP at pH 8.5 and observed the formation of peptide **11**_**GG**_ (70%) in good yield. It is expected that other phosphines (PR_3_), and potentially phosphine (PH_3_), can achieve this fragmentation. While PH_3_ is the primary volatile form of phosphorus and a trace constituent of the Earth’s atmosphere, it is likely produced biologically (or industrially) on the Earth.^42−46^ It remains unclear whether phosphines would be prebiotically available, and other prebiotic reagents may be required to induce this fragementation.^44−46^ Exploration of the prebiotic conditions that could achieve this fragmentation on the early Earth remains to be tested; however, the effective and spontaneous ligation of cysteamine-peptoids warrants further study of this fragmentation in a prebiotic context.

Our observations support a scenario in which nitriles may have served as activated substrates for peptide synthesis on the primordial Earth, with cysteamine (**5**) playing a role in their ligation. It is of note that **5** is a component of pantetheine,^20,47−49^ which is essential throughout all domains of life as an acyl-carrier, suggesting that **5** could have been intimately associated with peptide synthesis since the origins of life.

## Supporting Information Available

Additional experimental details, materials, and methods, including NMR spectra of ligation reactions and isolated compound, mass spectrometry, and IR spectroscopic data (PDF)
