# Supplementary material for: Spontaneous Peptide Ligation Mediated by Cysteamine
Source: JACS Au. 2024 Apr 29;4(5):1752–7. doi: 10.1021/jacsau.4c00243 (PMC11134366; doi:10.1021/jacsau.4c00243)
Supplement: Supplementary file 1 — au4c00243_si_001.pdf [file au4c00243_si_001.pdf]

Spontaneous peptide ligation mediated by cysteamine.

Abid Barat[1] and Matthew W. Powner[1]*

[1] Department of Chemistry, University College London, 20 Gordon Street, London, WC1H 0AJ, UK

This is a Supplementary Information (SI) transcript containing experimental information, NMR spectra, mass spectrometry data, yields of reactions, melting points and infra-red data.

Contents

General experimental: 4

Prebiotic synthesis of *N*-thioalkyl amino acids (**16** and **17**): 6

Synthesis of $\mathbf{16_G}$: 6

Thiazolidine **12a**: 6

Reaction of thiazolidine (**12a**) with HCN to yield *N*-thioethyl amino acid ($\mathbf{16_G}$) via *N*-thioethyl aminonitrile ($\mathbf{8_G}$): 18

Synthesis of $\mathbf{16_A}$: 21

Thiazolidine **12b** synthesis: 21

Reaction of thiazolidine (**12b**) with HCN to yield *N*-thioethyl amino acid ($\mathbf{16_A}$) via *N*-thioethyl aminonitrile ($\mathbf{8_A}$): 23

Synthesis of $\mathbf{17_G}$: 28

Reductive amination of α-keto acids (**18**) with cysteamine (**5**) to yield **16**: 31

19a synthesis: 31

Selective **19a** synthesis in presence of competing amine nucleophiles: 36

19a reduction with Na[$CNBH_3$] to yield $\mathbf{16_G}$: 42

Synthesis of $\mathbf{17_A}$: 45

Synthesis of $\mathbf{16_V}$: 50

Strecker Reaction from cyanohydrin (**21**) and cystamine (**6**) to yield **20**: 55

Synthesis of thiazoline **26** from cysteamine (**5**) and cyanohydrin (**21a**): 55

Synthesis of thiazoline **27** from cysteamine **6** and HCN: 59

Synthesis of $\mathbf{8_G}$: 61

Synthesis of $\mathbf{8_A}$: 76

Ligation of amidonitriles **2** with β or γ-*N*-thiolkyl amino acids **16**, **17** or **30**: 82

Ac-Gly-CN ($\mathbf{2_G}$) Ligation with $\mathbf{16_G}$ to yield **10a**: 84

Intermolecular CPL with $\mathbf{2_G}$ and $\mathbf{33_G}$ to yield $\mathbf{34_{GG}}$: 101

$\mathbf{32_G}$ ligation of Ac-Gly-CN $\mathbf{2_G}$ to yield **33a**: 103

Selective ligation of $\mathbf{2_G}$ with $\mathbf{16_G}$ in presence of $\mathbf{17_G}$: 105

Ac-Gly-CN ($\mathbf{2_G}$) Ligation with $\mathbf{16_A}$ to yield **10b**: 111

Ac-Gly-CN ($\mathbf{2_G}$) Ligation with $\mathbf{16_V}$ to yield **10c**: 114

$\mathbf{16_G}$ ligation of $\mathbf{2_{(AA)m}}$ to yield **10**: 117

Ac-Ala-CN ($\mathbf{2_A}$): 117

Ac-Met-CN ($\mathbf{2_M}$): 121

Ac-Pro-CN ($\mathbf{2_P}$): 124

Ac-Ser-CN ($\mathbf{2_S}$): 127

Ac-Leu-CN (**2**$_L$): 130
Ac-Arg-CN (**2**$_R$): 133
Ac-Asp-CN (**2**$_D$): 136
Ac-Val-CN (**2**$_V$): 139
Ac-Ile-CN (**2**$_I$) 144
Ac-Gly-Gly-CN (**2**$_{GG}$): 148
Ac-Gly-Gly-Gly-CN (**2**$_{GGG}$): 150
Ac-Val-Gly-CN (**2**$_{VG}$): 152
16$_G$ selective ligation of α-amidonitriles: 154
2$_{βA}$ and **2**$_G$ competition for **16**$_G$: 154
2$_{Aib}$ and **2**$_G$ competition for **16**$_G$: 156
Selective **2**$_{DX}$ ligation with **16**$_G$: 157
Selectibe **2**$_{EX}$ ligation with **16**$_G$: 162
Reductive fragmentation: 165
Reductive fragmentation of **31a** to yield **11**$_{GG}$: 165
Reductive fragmentation of **10a** to yield **11**$_{GG}$: 166
Preparative synthesis: 167
Synthesis of **16**$_{AA}$: 167
Synthesis of **17**$_G$: 179
Synthesis of auxiliary **30**$_G$: 187
Synthetic preparation of ligation product **10a**: 194
Synthesis of **19a**: 207
References: 209

General experimental:

Reagents and solvents were obtained from commercial sources (Fluorochem, Sigma Aldrich, Apollo scientific, Alfa Aesar and Fischer scientific) and used without further purification, unless stated otherwise – commercial cysteamine was 10-20% oxidised and used without further purification. Deionised water was obtained from an Elga Option 3 purification system. Thin layer chromatography (TLC) was carried out on Merck aluminium backed DC 60 F254 0.2 mm pre-coated plates. Visualization of the TLC plates was achieved under ultraviolet light or by stain (permanganate or ninhydrin solution) as required. Sonication (30-40 KHz) was carried out by using an *U500H* ultrasonication cleaner. Solution pH values were measured using a Mettler Toledo Seven Compact pH meter with a Mettler Toledo InLab semi-micro pH probe, or a Corning 430 pH meter with a Fisherbrand FB68801 semi-micro pH probe. Flash column chromatography was performed on a Biotage Isolera One using Biotage SNAP KP-Sil brand columns. Infrared (IR) spectra were recorded on a Bruker Alpha FT-IR with a Platinum-ATR (attenuated total reflection) attachment as a solid or neat oil/liquid. Absorption maxima are reported in wavenumber (cm^{-1}) and the spectral range was between 400 cm^{-1} and 4000 cm^{-1} with a resolution of 0.01 cm^{-1}. Spectra were recorded by carrying out 16 scan cycles with a 16-cycle background scan performed before each sample.

Mass spectra and accurate mass measurements were recorded on a Waters LCT Premier QTOF connected to a Waters Autosampler Manager 2777C, Thermo Finnigan MAT900, and an Agilent LC connected to an Agilent 6510 QTOF mass spectrometer.

^{1}H and ^{13}C NMR spectra were recorded on Bruker NMR spectrometers (AVANCE Neo 700, AVANCE III 600, AVANCE III 400, AVANCE 500 and AVANCE 300) equipped with a Bruker room temperature 5 mm multinuclear gradient probe and a 5 mm DCH cryoprobe (700 and 600 MHz) or a gradient probe (500, 400 and 300 MHz). ^{13}C NMR spectra were proton decoupled. All chemical shifts (δ) are reported in parts per million (ppm) relative to residual solvent peaks, and ^{1}H NMR chemical shifts relative to TMS were calibrated using the residual solvent peak (residual solvent peaks: (δ) D_2O – 4.79; DMSO-d_6 – 2.50; $CDCl_3$ – 7.26; CD_3OD – 3.31; H_2O/D_2O 9:1 – 4.79). Nuclear assignments were made using 2D NMR homonuclear and heteronuclear correlation spectroscopy ($^{1}H^{1}H$ COSY; $^{1}H^{13}C$ HSQC; $^{1}H^{13}C$ HMBC). Where noted, solvent suppression pulse sequence with pre-saturation and spoil gradients were used to obtain ^{1}H NMR spectra (noesygppr1d, Bruker) and $^{1}H^{13}C$ HMBC NMR spectra (hmbcgplpndprqf, Bruker). Coupling constants are reported in Hertz (Hz). Spin multiplicities are indicated by symbols: s (singlet); d (doublet); t (triplet); q (quartet); qn (quintet); spt (septet); oct (octet), m (multiplet); obs. (obscured signal); app. (apparent), or a combination of these. Diastereotopic geminal (AB) spin systems coupled to one or two additional nuclei are reported as ABX and ABXY, respectively. NMR data are reported as follows: chemical shift (multiplicity, coupling constants (*J*), number of protons,

nuclear assignment). All coupling constants are $^{3}J_{HH}$ unless otherwise stated and quoted to the nearest 0.1 Hz. Spectra were recorded at 298 K except VT-NMRs. ^{1}H NMR spectra for peptoid coupling reactions are shown 0.0-5.5 ppm. Potassium hydrogen phthalate (KHPH; 7.32-7.43 ppm) internal standard not shown. Methylsulfonylmethane (MSM; 3.16 ppm) internal standard shown. Where coloured dots are used on the spectra, its colour corresponds to the colour of compounds in the scheme.

Prebiotic synthesis of *N*-thioalkyl amino acids (**16** and **17**):

Synthesis of **16_G**:

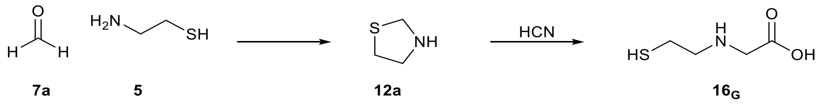

Thiazolidine **12a**:

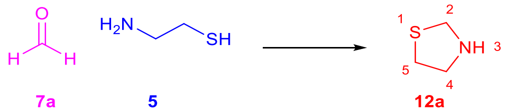

A solution of formaldehyde **7a** (50 mM), cysteamine **5** (60 mM) and MSM (25 mM; internal standard) was adjusted to pH 5-9 with 1-4 N HCl/NaOH and then incubated in H_2O/D_2O (9:1) at room temperature. The reaction was monitored by periodic acquisition of ^{1}H NMR spectra. After 1 hour at pH 9 and 24 hours at pH 5-7, near-complete conversion to **12a** (>95%) was observed. Identity of product (**12a**) was confirmed by spiking with authentic commercial standard (see Supplementary Figure 1-4).

Compound 12a: ^{1}H NMR (600 MHz, H_2O/D_2O 9:1, pH 9): δ_H 2.80 (t, *J* = 6.3 Hz, 2H, (C5)-H), 3.05 (t, *J* = 6.3 Hz, 2H, (C4)-H), 4.01 (s, 2H, (C2)-H). **^{13}C NMR (150 MHz, H_2O/D_2O 9:1, pH 9):** δ_C 33.2 (C5), 52.2 (C4), 53.9 (C2).

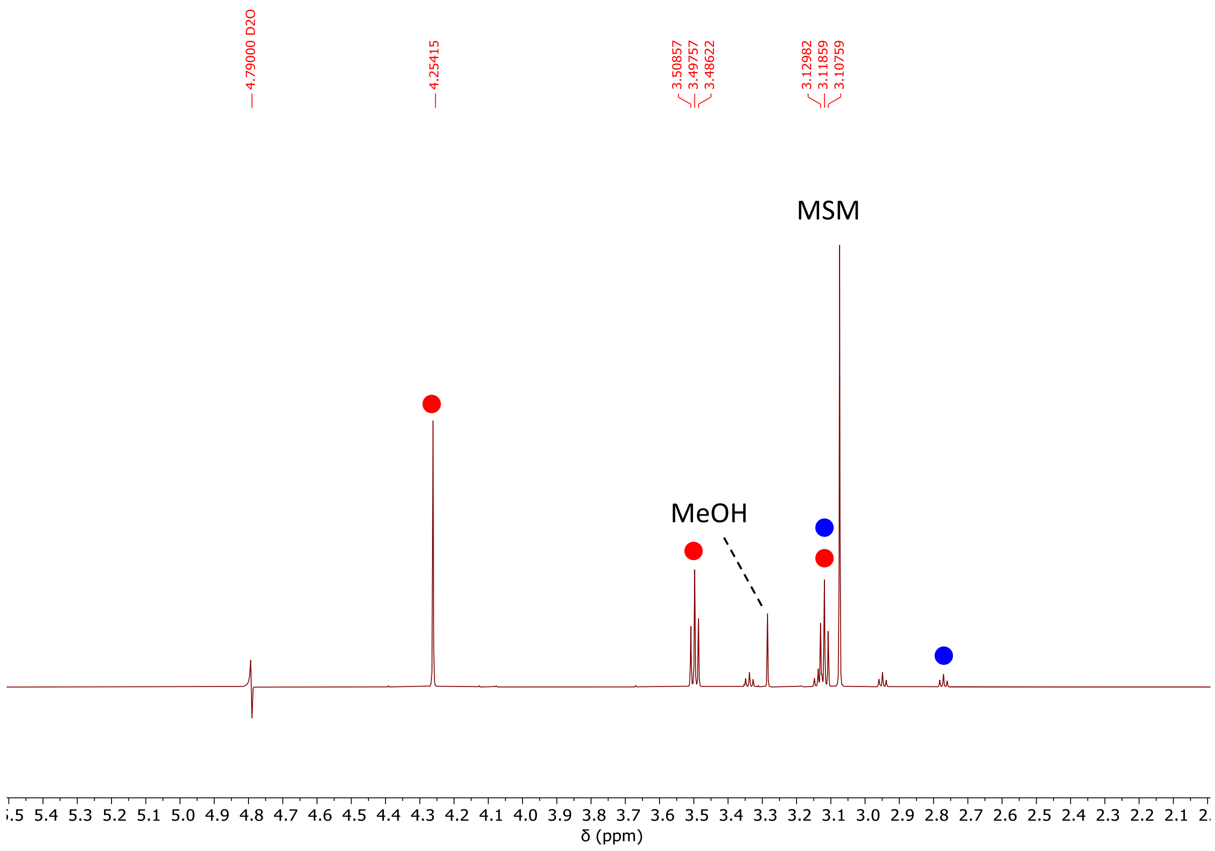

Figure S1: ^{1}H NMR (600 MHz, H_2O/D_2O 9:1, noesygppr1d, 2.0-5.5 ppm) spectrum to show the reaction of cysteamine (**5**, 60 mM) and formaldehyde (**7a**, 50 mM) with MSM (25 mM; internal standard) at pH 5 and room temperature, which yields **12a**.

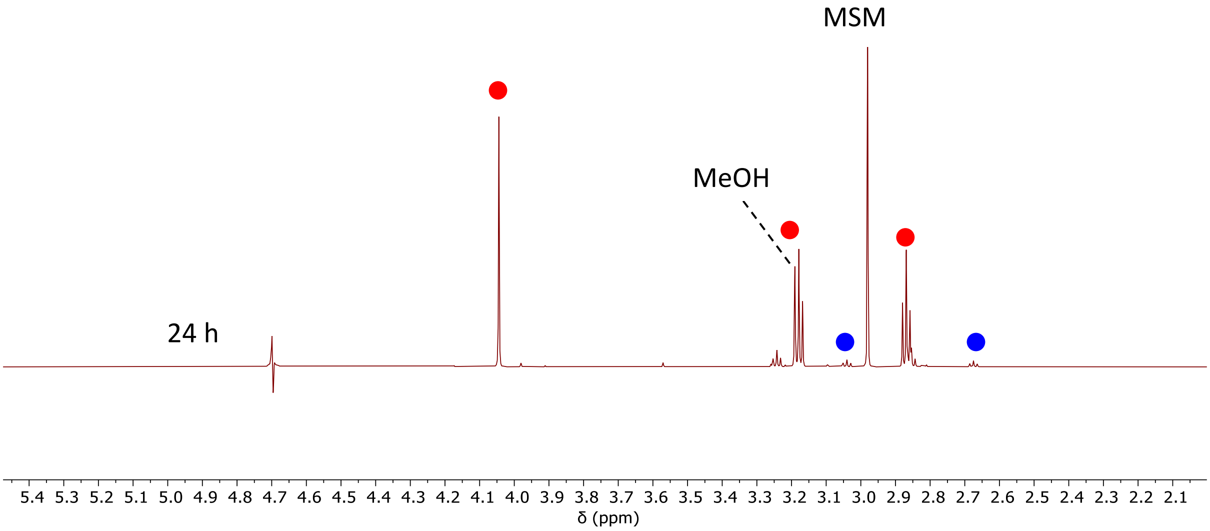

Figure S2: ^{1}H NMR (600 MHz, H_2O/D_2O 9:1, noesygppr1d, 2.0-5.5 ppm) spectrum to show the reaction of cysteamine (**5**, 60 mM) and formaldehyde (**7a**, 50 mM) with MSM (25 mM; internal standard) at pH 7 and room temperature, which yields **12a**.

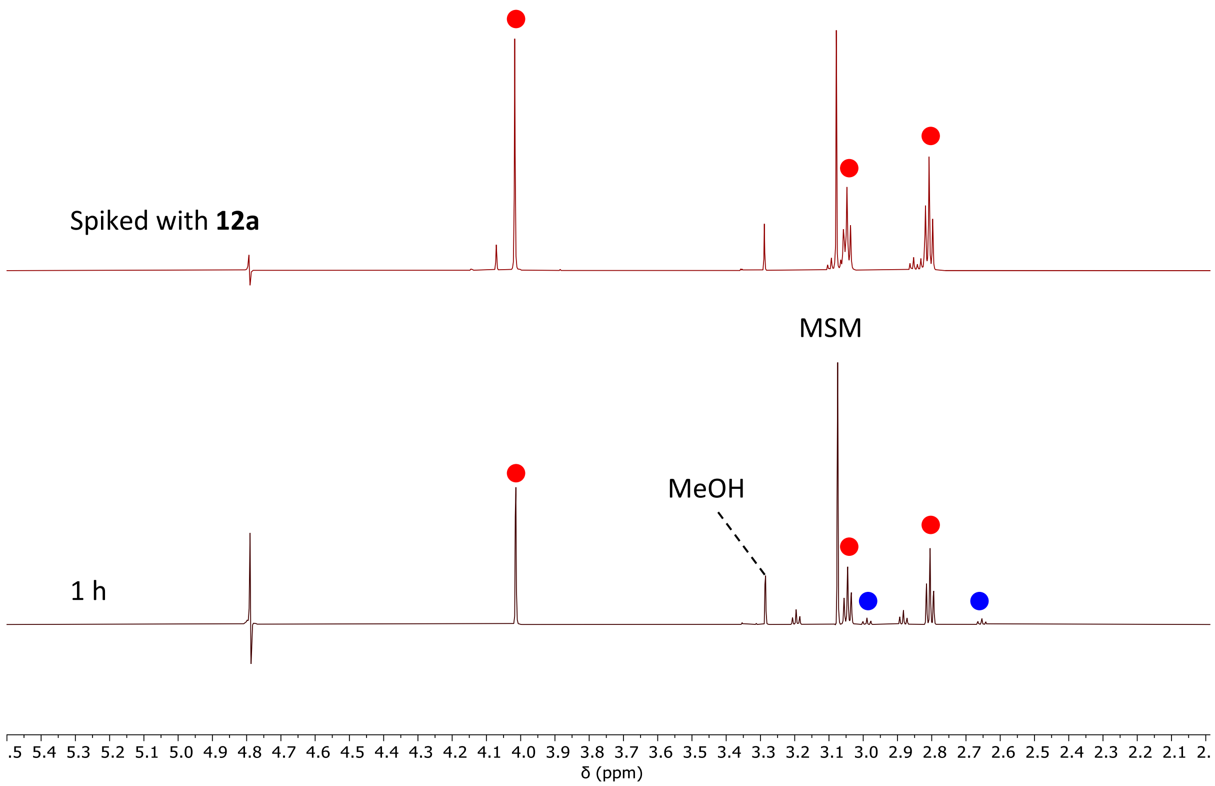

Figure S3: ^{1}H NMR (600 MHz, H_2O/D_2O 9:1, noesygppr1d, 2.0-5.5 ppm) spectra to show the reaction of cysteamine (**5**, 60 mM) and formaldehyde (**7a**, 50 mM) with MSM (25 mM; internal standard) at pH 9 and room temperature, which yields **12a** and identified by spiking with authentic synthetic standard.

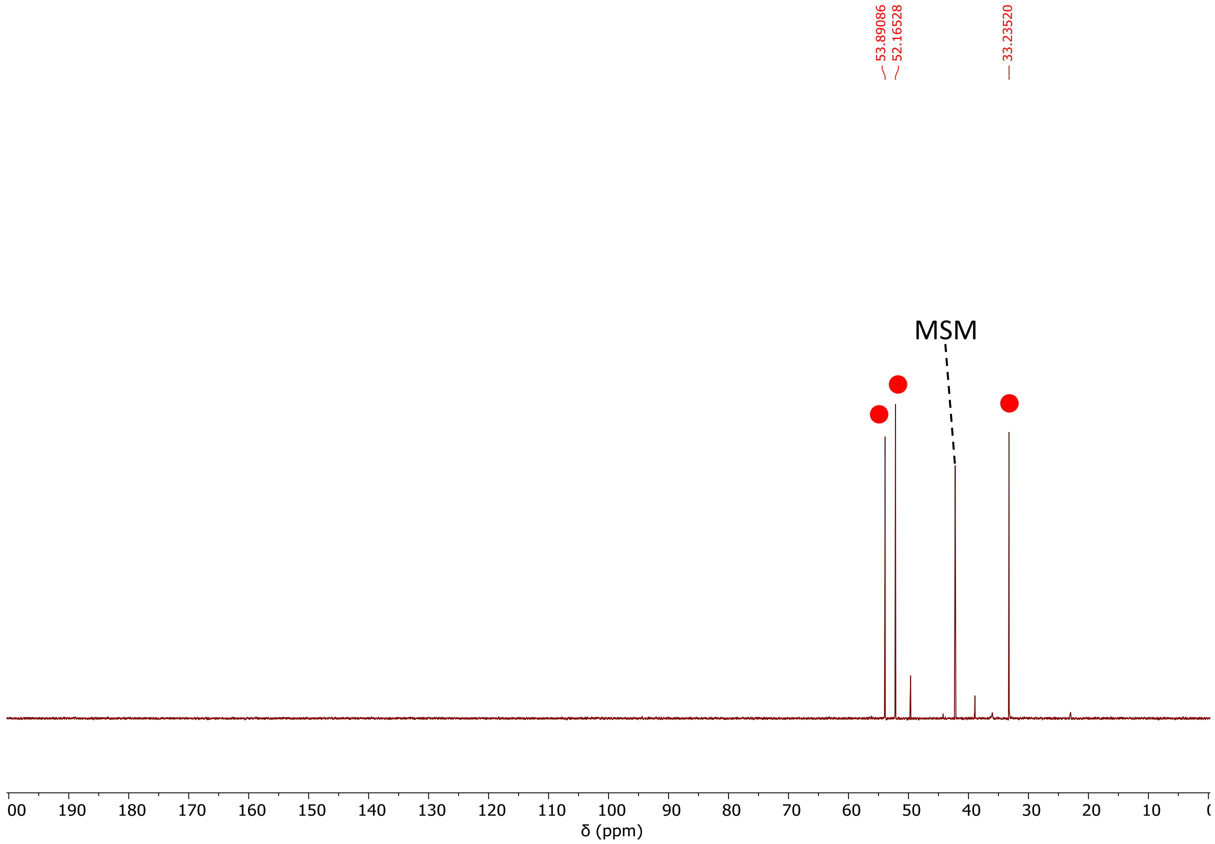

Figure S4: ^{13}C NMR (150 MHz, H_2O/D_2O, 0.0-200 ppm) spectrum to show the reaction of cysteamine (**5**, 60 mM) and formaldehyde (**7a**, 50 mM) with MSM (25 mM; internal standard) at pH 9 and room temperature, which yields **12a**.

Selective thiazolidine (**12a**) formation in presence of competing amine nucleophiles:

General procedure **A**:

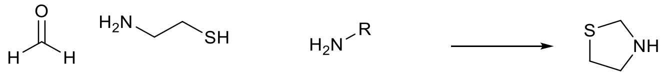

7a **5** **6, 13 or 15a-d** **12a**

15a R=H, **15b** R=CH_2CH_3, **15c** R=CH_2CH_2OH, **15d** R=$CH_2CH_2NH_2$
6 R=$CH_2CH_2SSCH_2CH_2NH_2$, **13** R=$CH_2CH_2CH_2SH$

A soluton of formaldehyde **7a** (100 mM), cysteamine **5** (150 mM), competing amine nucleophile **13** (150 mM) and MSM (25 mM; internal standard) were incubated at pH 9 in H_2O/D_2O (9:1, 1 mL) at room temperature. The reaction was monitored by periodic acqisition of ^1H NMR spectra to yield a mixture of thiazolidine (**12a**)/1,3-thazinane (**14a**) 47:53 in 30 mins.

A solution of formaldehyde **7a** (100 mM), cysteamine **5** (150 mM), competing amine nucleophile **6** or **15a-d** (150 mM) and MSM (25 mM; internal standard) were incubated at pH 9 in H_2O/D_2O (9:1, 1 mL) at room temperature. The reaction was monitored by periodic acqisition of ^1H NMR spectra to yield exclusively **12a** in near quantitative yield (>95%) in 30 mins.

Homocysteamine **13**:

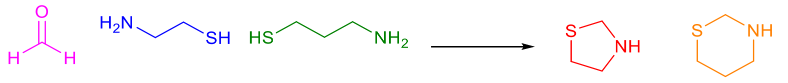

7a **5** **13** **12a** **14a**

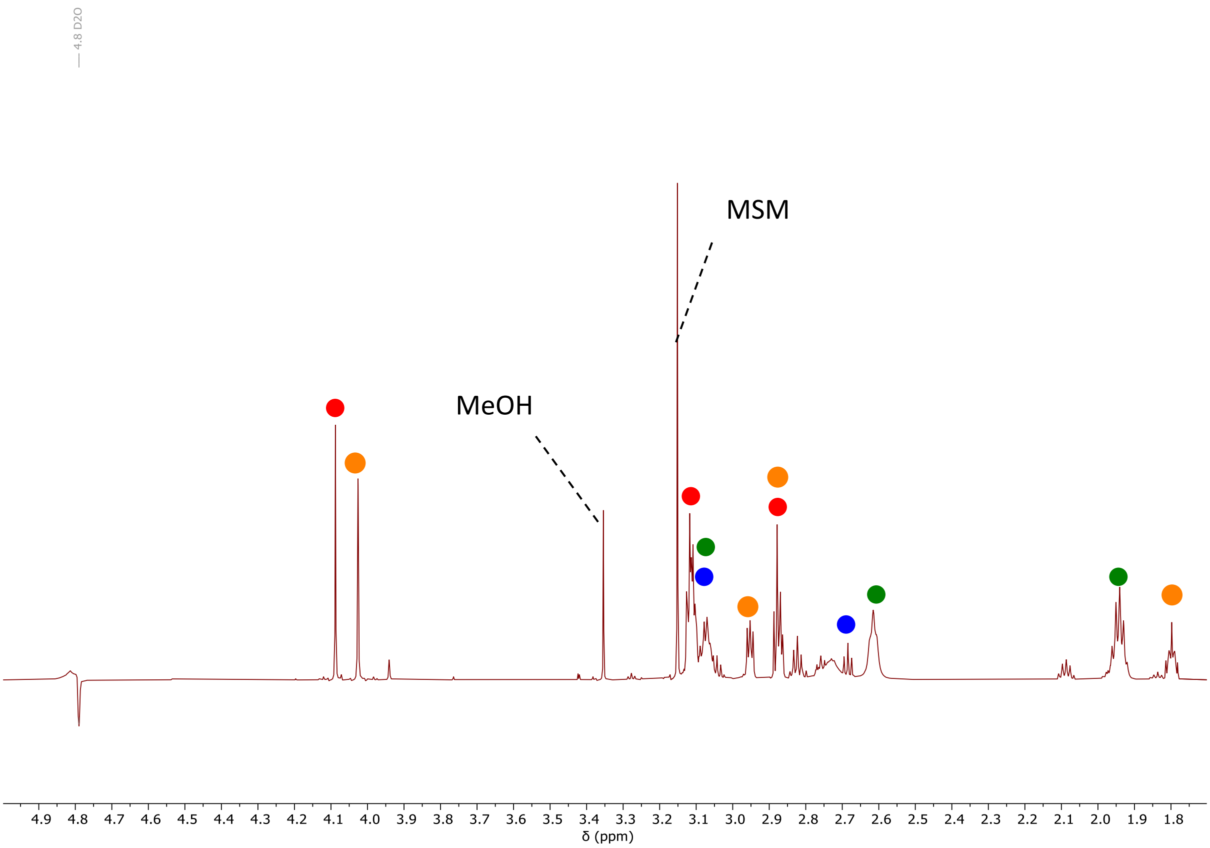

Figure S5: ^{1}H NMR (700 MHz, H_2O/D_2O 9:1, noesygppr1d, 2.8-5.0 ppm) spectrum to show the reaction of cysteamine (**5**, 150 mM), formaldehyde (**7a**, 100 mM) and homocysteamine (**13**, 150 mM) with MSM (25 mM; internal standard) at pH 9 and room temperature, which yields a mixture of **12a** and **14a**.

Ammonia **15a**:

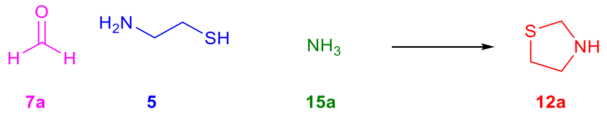

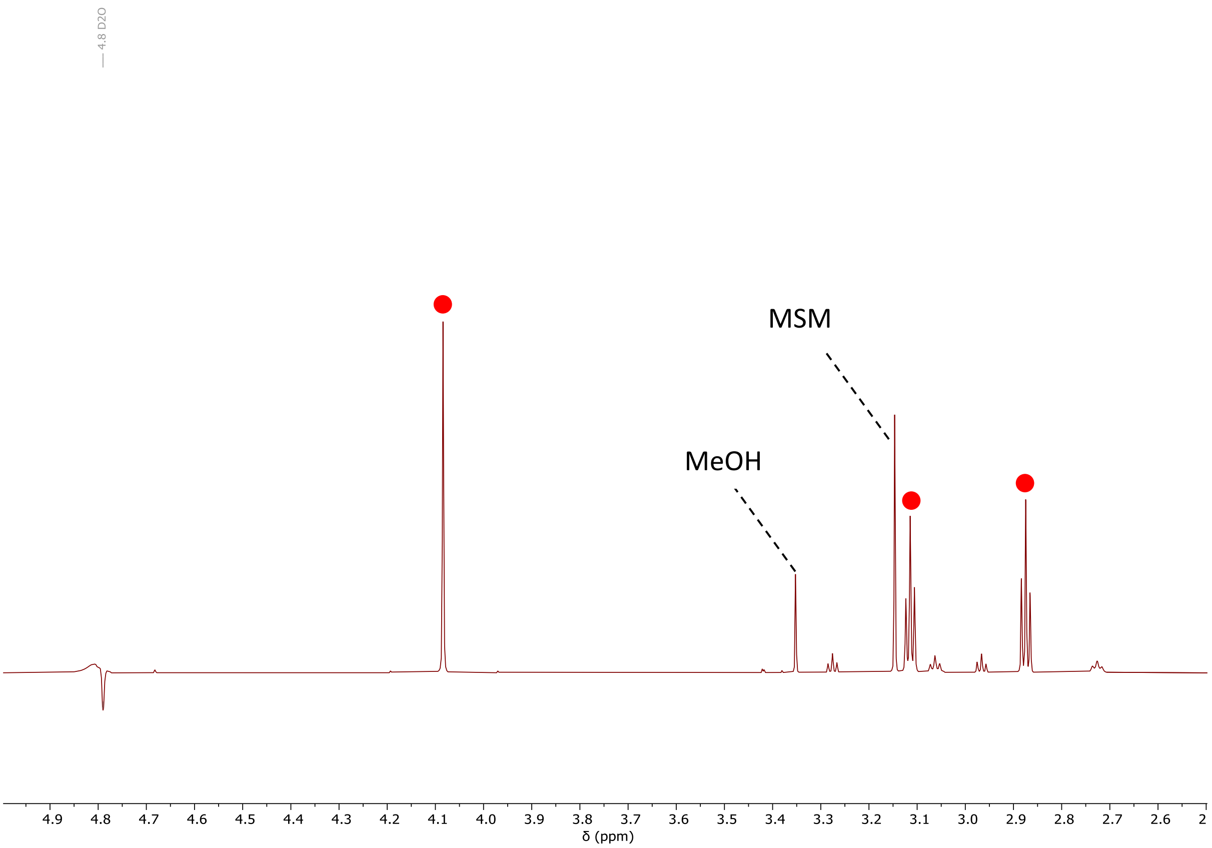

Figure S6: ^{1}H NMR (700 MHz, H_2O/D_2O 9:1, noesygppr1d, 2.5-5.0 ppm) spectrum to show the reaction of cysteamine (**5**, 150 mM), formaldehyde (**7a**, 100 mM) and ammonia (**15a**, 150 mM) with MSM (25 mM; internal standard) at pH 9 and room temperature, which selectively yields **12a**.

Ethylamine **15b**:

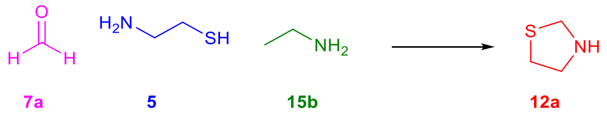

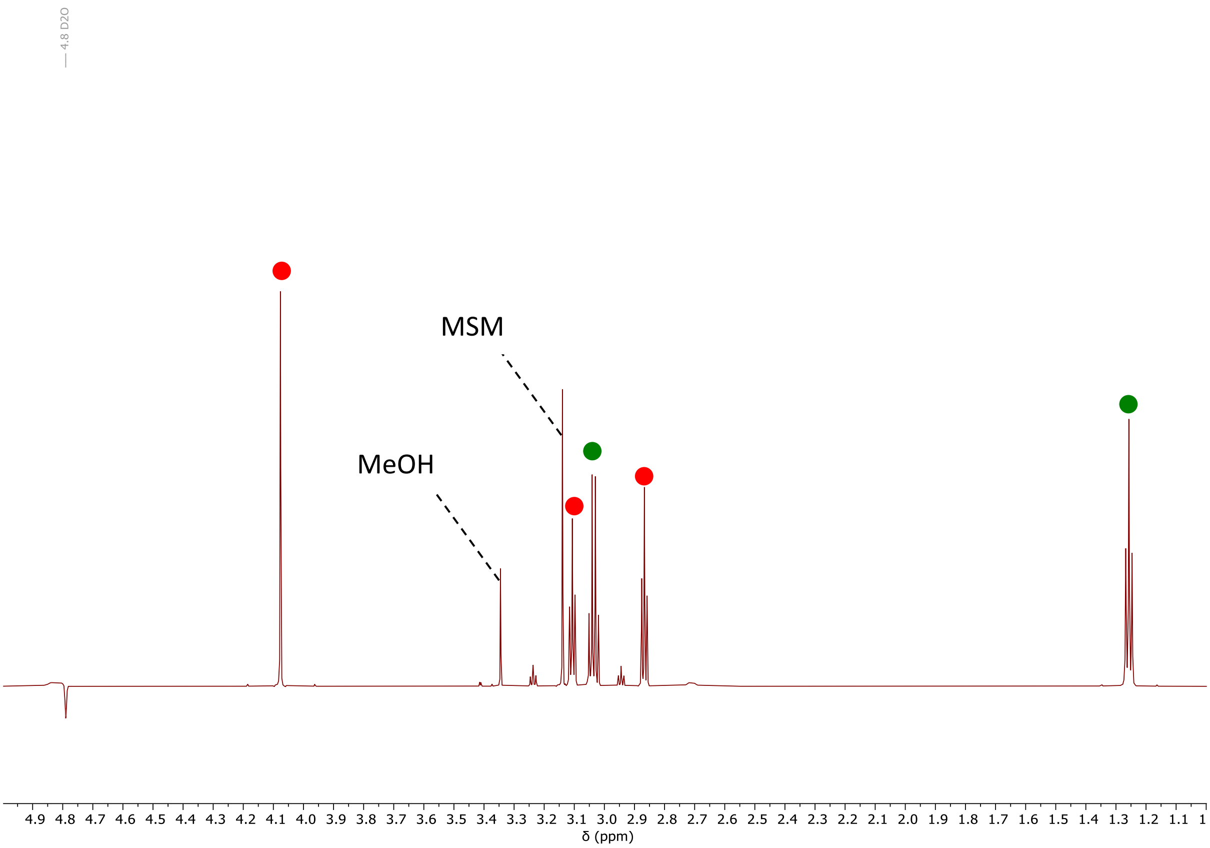

Figure S7: ^1H NMR (700 MHz, H_2O/D_2O 9:1, noesygppr1d, 1.0-5.0 ppm) spectrum to show the reaction of cysteamine (**5**, 150 mM), formaldehyde (**7a**, 100 mM) and ethylamine (**15b**, 150 mM) with MSM (25 mM; internal standard) at pH 9 and room temperature, which selectively yields **12a**.

Ethanolamine **15c**:

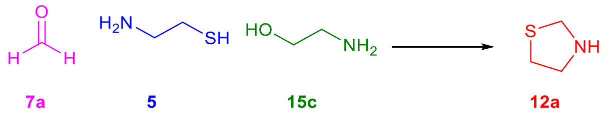

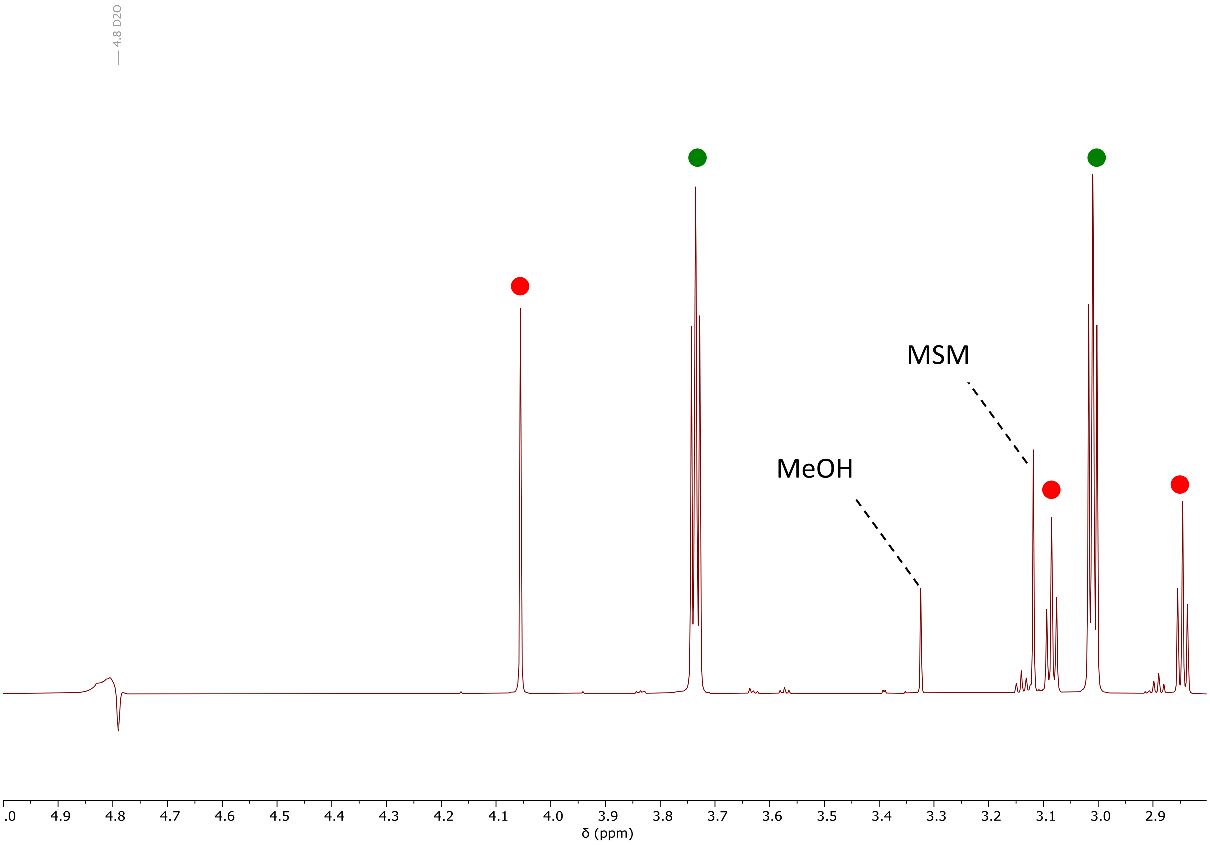

Figure S8: ^1H NMR (700 MHz, H_2O/D_2O 9:1, noesygppr1d, 2.8-5.0 ppm) spectrum to show the reaction of cysteamine (**5**, 150 mM), formaldehyde (**7a**, 100 mM) and ethanolamine (**15c**, 150 mM) with MSM (25 mM; internal standard) at pH 9 and room temperature, which selectively yields **12a**.

Ethylenediamine **15d**:

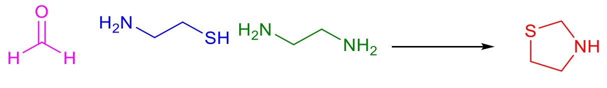

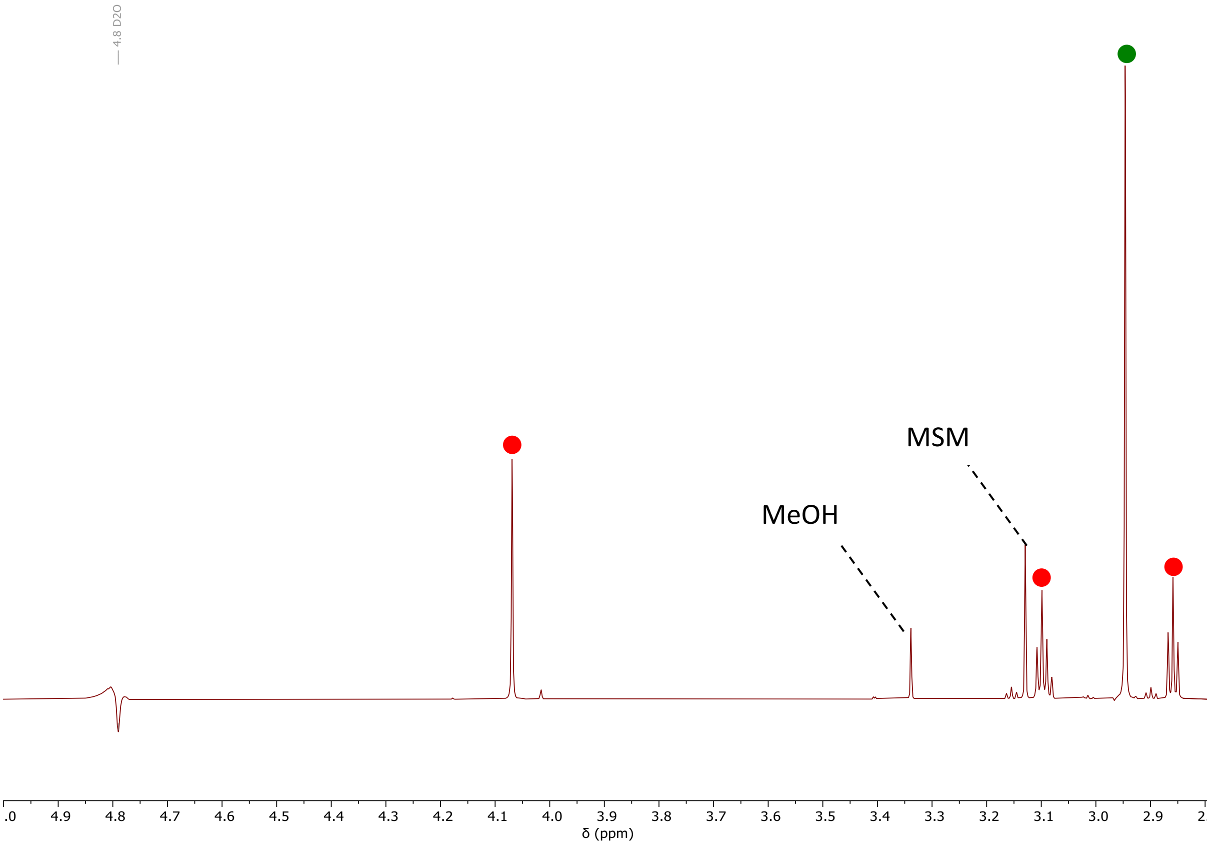

Figure S9: ^{1}H NMR (700 MHz, H_2O/D_2O 9:1, noesygppr1d, 2.8-5.0 ppm) spectrum to show the reaction of cysteamine (**5**, 150 mM), formaldehyde (**7a**, 100 mM) and ethylenediamine (**15d**, 150 mM) with MSM (25 mM; internal standard) at pH 9 and room temperature, which selectively yields **12a**.

Cystamine **6**:

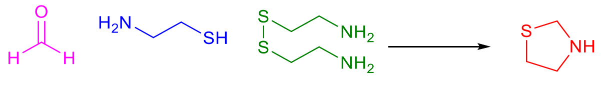

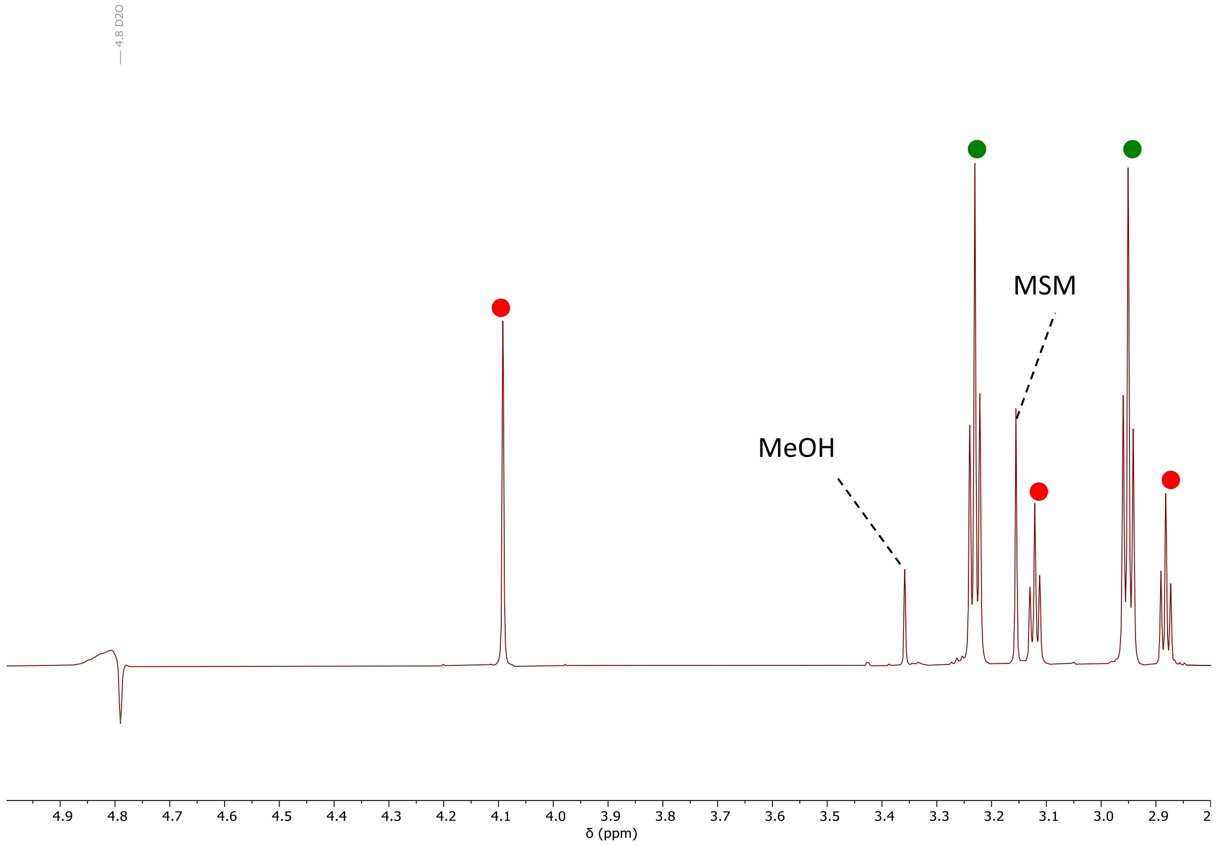

Figure S10: ^{1}H NMR (700 MHz, H_2O/D_2O 9:1, noesygppr1d, 2.8-5.0 ppm) spectrum to show the reaction of cysteamine (**5**, 150 mM), formaldehyde (**7a**, 100 mM) and cystamine (**6**, 150 mM) with MSM (25 mM; internal standard) at pH 9 and room temperature, which selectively yields **12a**.

Reaction of thiazolidine (**12a**) with HCN to yield *N*-thioethyl amino acid (**16**$_{G}$) via *N*-thioethyl aminonitrile (**8**$_{G}$):

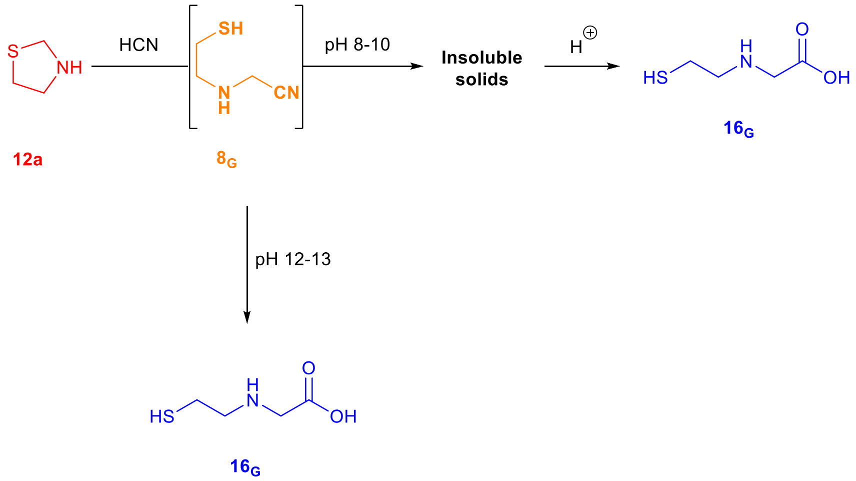

Thiazolidine **12a** (50 mM), NaCN (156 mM) and MSM (25 mM; internal standard) were dissolved in H_2O (5 mL) and the solution was adjusted to pH 8 – 13 with HCl/NaOH (1-4N). The resultant solution was then incubated at room temperature or 60 °C. After 24 hours (at 60 °C) solids were observed in reactions that were incubated between pH 8 and pH 10. After the specified time the samples were homogenised, at room temperature by vortex, and an aliquot (500 µL) of the homogenised sample was diluted with D_2O (60 µL) and NMR spectra of the reaction (supernatant) were acquired (Supplementary Table 1). HCl (12 M, 500 µL) was added to another aliquot (500 µL) of the homogenised reaction mixtures and the resultant solutions were incubated at 100 °C. After 2 hours the solution was cooled to room temperature and an aliquot of this acidic solution (100 µL) was diluted with H_2O/D_2O (3:1, 400 µL) and crystal of TCEP·HCl (~5 mg) was added. The solution was then adjusted to between pH 6 to pH 8 with NaOH (6 M) and NMR spectra were acquired. Formation of **16**$_{G}$ (Supplementary Table 1 & Supplementary Figure 11-12 and) was observed and confirmed by sample spiking with authentic synthetic standard of **16**$_{G}$.

		16_G/%		
**Entry**	**pH**	**r.t. (5 d)**	**60 °C (1 d)**	**60 °C (1 day) then HCl**
**1**	8.0	-	-	30
**2**	9.0	-	-	39
**3**	9.5	-	-	55
**4**	10.0	-	-	60
**5**	12.0	20	25	60
**6**	13.0	50	58	65

Table S1: Table to show yield of **16_G** observed from the reaction of thiazolidine **12a** and HCN before and after HCl hydrolysis at pH 8-13.

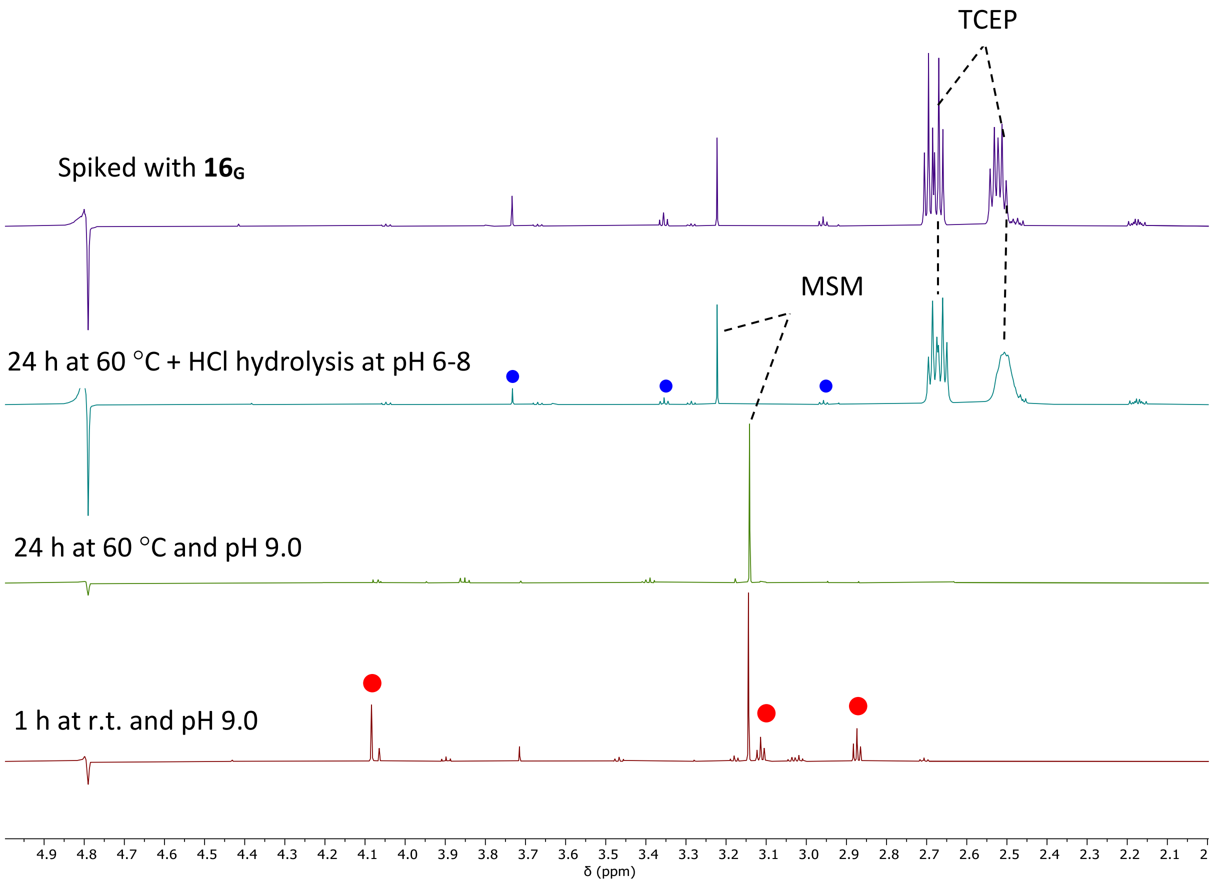

Figure S11: ^{1}H NMR (700 MHz, H_2O/D_2O 9:1, noesygppr1d, 2.0-5.0 ppm) spectra to show the formation of **16_G** from thiazolidine (**12a,** 50 mM) and NaCN (156 mM), with MSM (25 mM; internal standard) at pH 9.0 and at 60 °C (before and after HCl (6M) hydrolysis followed by the addition of TCEP.HCl (~5 mg)). Spiking the HCl hydrolyzed reaction mixture with **16_G** enhanced the signals from 39% to 80%.

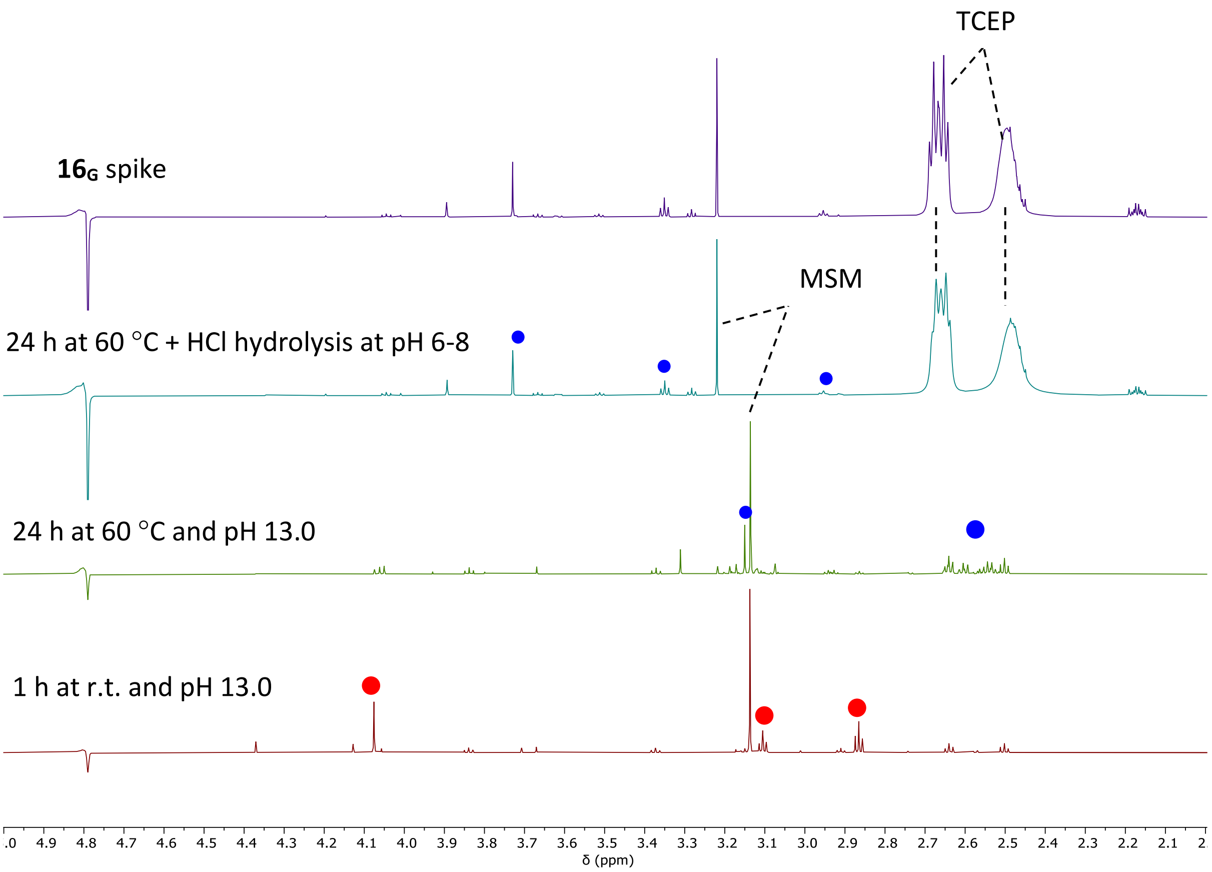

Figure S12: ^{1}H NMR (700 MHz, H_2O/D_2O 9:1, noesygppr1d, 2.0-5.0 ppm) spectra to show the formation of **16**$_G$ from thiazolidine (**12a**, 50 mM) and NaCN (156 mM), with MSM (25 mM; internal standard) at pH 13.0, both at room temperature and 60 °C (before and after HCl (6M) hydrolysis following the addition of TCEP.HCl (~5 mg)). Spiking the HCl hydrolyzed reaction mixture with **16**$_G$ enhanced the signals from 65% to 85%.

Synthesis of **16_A**:

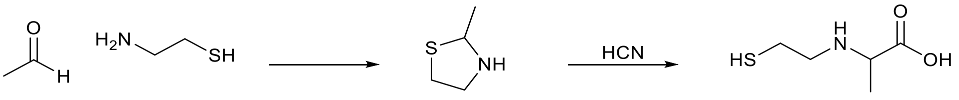

7b **5** **12b** **16_A**

Thiazolidine **12b** synthesis:

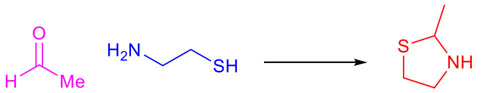

7b **5** **12b**

Acetaldehyde **7b** (61 mM), cysteamine **5** (60 mM)[a] and MSM (25 mM; internal standard) were dissolved in H_2O/D_2O (9:1, 2 mL) and adjusted to pH 9. The solution was then monitored by ^1H NMR spectroscopy at room temperature. Near quantitative formation of **12b** (>95% with respect to cysteamine **5**) was observed after 20 mins. The product was confirmed by spiking with authentic commercial standard.

[a] Commercial cysteamine **5** is 10% oxidised and not further purified.

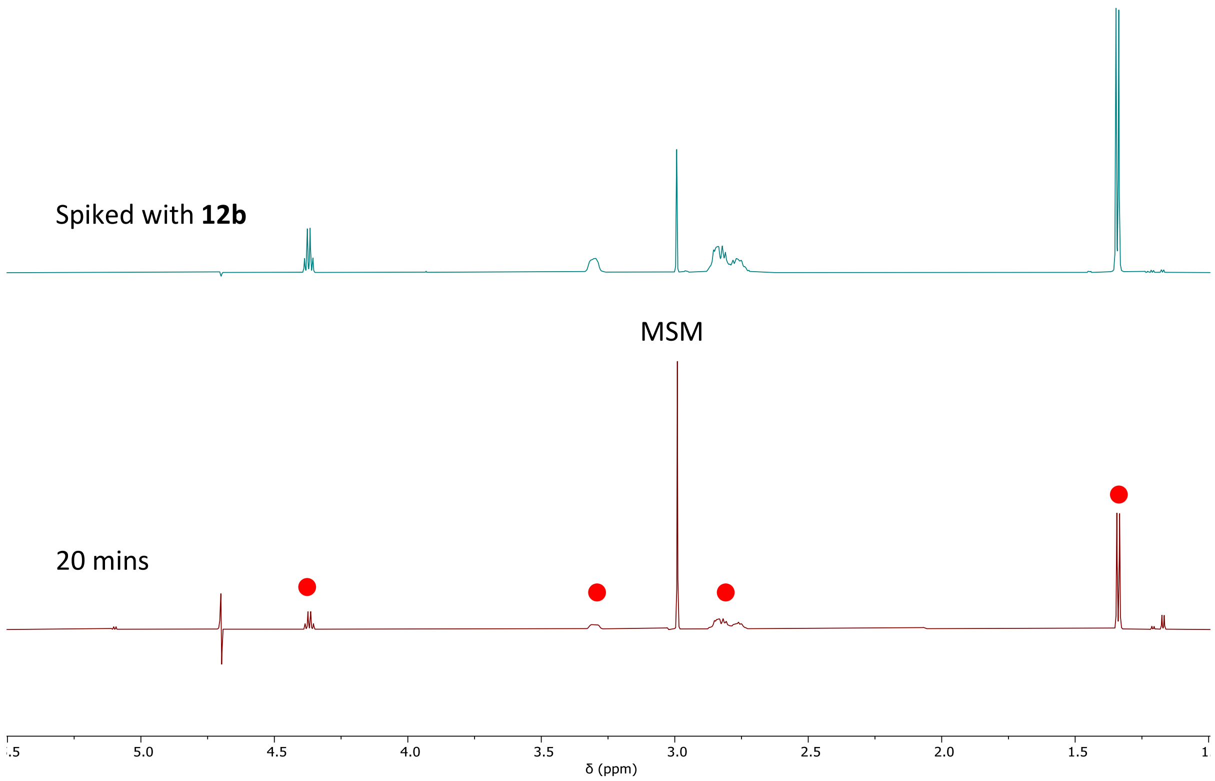

Figure S13: ^{1}H NMR (600 MHz, H_2O/D_2O 9:1, noesygppr1d, 1.0-5.5 ppm) spectra to show the reaction of cysteamine (**5**, 60 mM) and acetaldehyde (**7b**, 55 mM) with MSM (25 mM; internal standard) at pH 9.0 and room temperature, which yields **12b** and identified by spiking with authentic commercial standard.

Reaction of thiazolidine (**12b**) with HCN to yield *N*-thioethyl amino acid (**16**$_A$) via *N*-thioethyl aminonitrile (**8**$_A$):

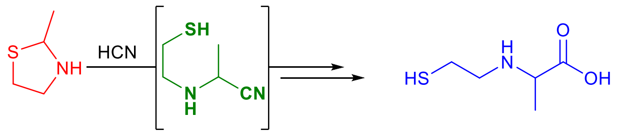

12b **8**$_A$ **16**$_A$

Thiazolidine **12b** (50 mM), NaCN (155 mM) and MSM (25 mM; internal standard) were dissolved in H_2O (5 mL) at pH 9.5 and then the resultant solution was heated at 60 °C for 24 hours before cooling to room temperature to yield a pale-yellow solution. HCl (12 M, 250 µL) was added to an aliquot of the cooled solution (250 µL) and the reaction was then heated at 100 °C for 1.5 hours. The reaction was then cooled to room temperature before a crystal of TCEP.HCl (~5 mg) and D_2O (100 µL) were added, and the resultant solution adjusted to pH ~7 (with HCl/NaOH 1-4N). ^{1}H NMR analysis indicated the synthesis of **16**$_A$ (75%). The identity of **16**$_A$ was confirmed by spiking with authentic synthetic standard.

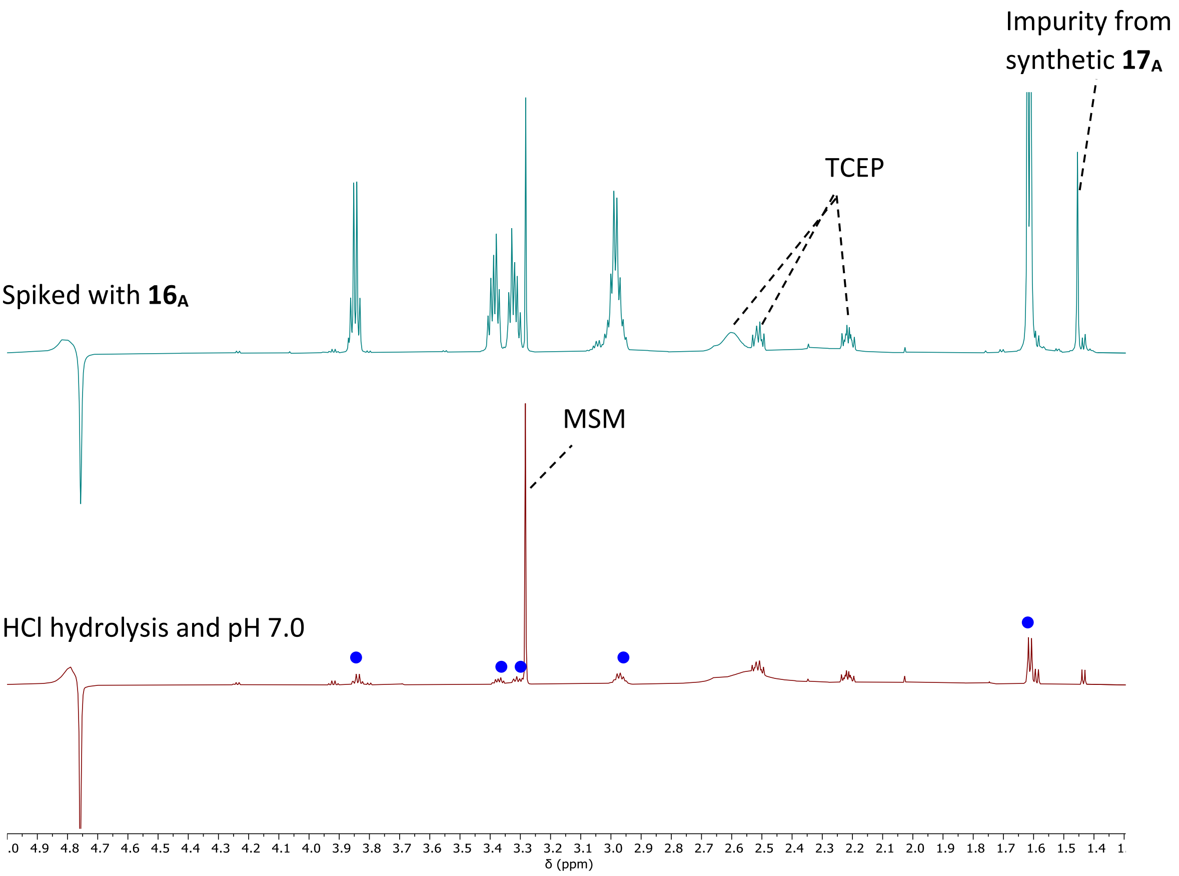

Figure S14: ^{1}H NMR (600 MHz, H_2O/D_2O 9:1, noesygppr1d, 1.3-5.0 ppm) spectra to show the formation of **16**$_A$ (identified by spiking with authentic synthetic standard) from thiazolidine (**12b**, 50 mM) and NaCN (155 mM), with MSM (25 mM; internal standard) at pH 9.5 and at 60 °C (after HCl (6M) hydrolysis and the addition of TCEP.HCl (~5 mg)).

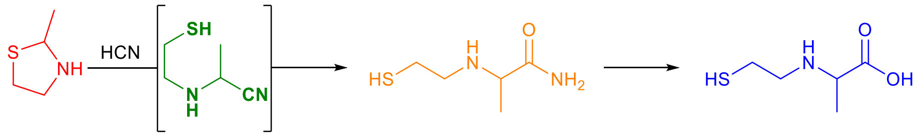

Thiazolidine **12b** (50 mM), NaCN (160 mM) and potassium hydrogen phthalate (KHPH; 25 mM, internal standard) were dissolved in H_2O (1 mL) and the solution was adjusted to pH 13 with NaOH (3 M), and then incubated at room temperature or 60 °C. An aliquot (500 µL) of the solution incubated at room temperature was diluted with D_2O (60 µL) and monitored by regular acquisition of NMR spectra, whilst D_2O (60 µL) was added to an aliquot (500 µL) of the solution incubated at 60 °C after heating was stopped and analysed by acquisition of NMR spectra. The formation of **28**$_A$ and then **16**$_A$ was observed (Supplementary Figure 15-17). At room temperature, **28**$_A$ (78%) and **16**$_A$ (6%) were observed after 14.5 hours. At 60 °C, **28**$_A$ (22%) and **16**$_A$ (56%) were observed after 5.5 hours.

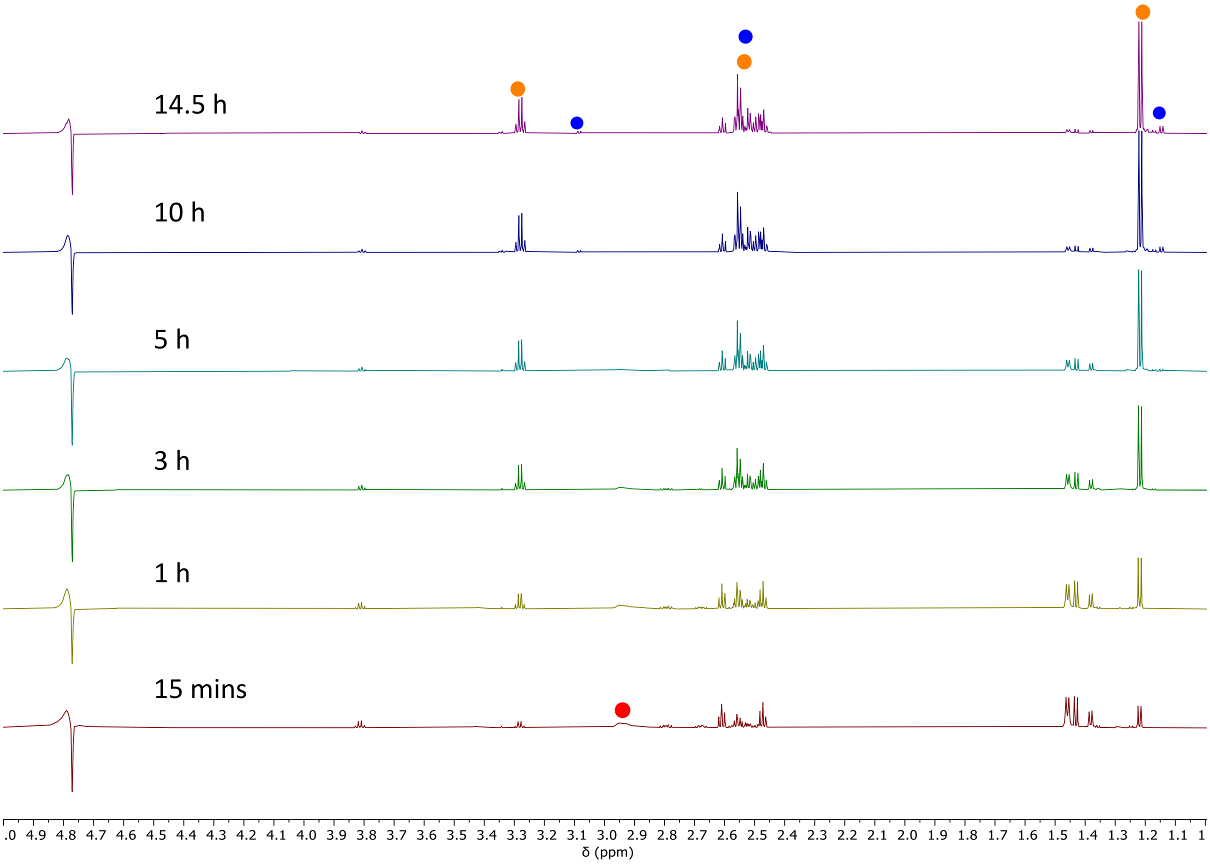

Figure S15: ^{1}H NMR (700 MHz, H_2O/D_2O 9:1, noesygppr1d, 1.0-5.0 ppm) spectra to show the formation of **28**$_A$ and **16**$_A$ from thiazolidine (**12b**, 50 mM) and NaCN (160 mM), at pH 13.0 and at room temperature.

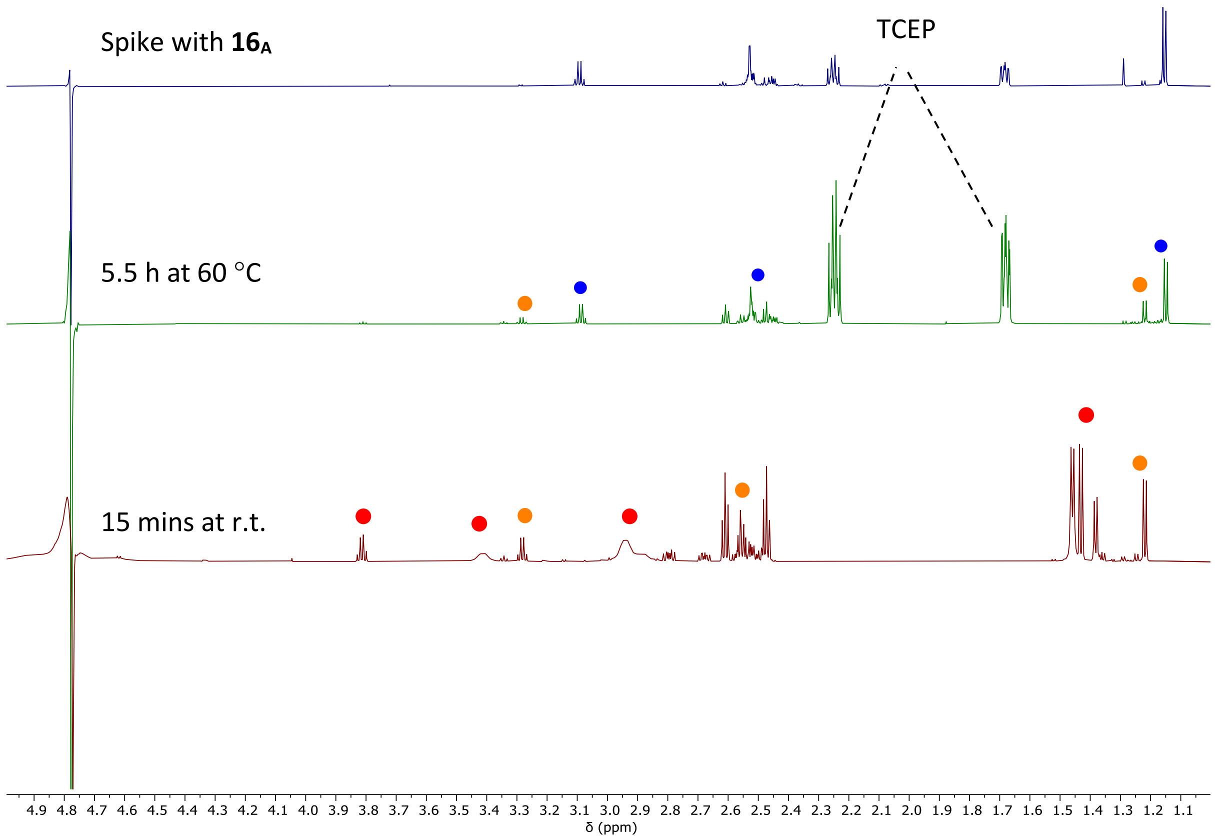

Figure S16: ^{1}H NMR (700 MHz, H_2O/D_2O 9:1, noesygppr1d, 1.0-5.0 ppm) spectra to show the formation of **28**$_{A}$ and **16**$_{A}$ from thiazolidine (**12b**, 50 mM) and NaCN (160 mM), at pH 13.0 and at 60 °C (NMR acquired after TCEP addition). The identity of **16**$_{A}$ was confirmed by spiking with authentic synthetic standard.

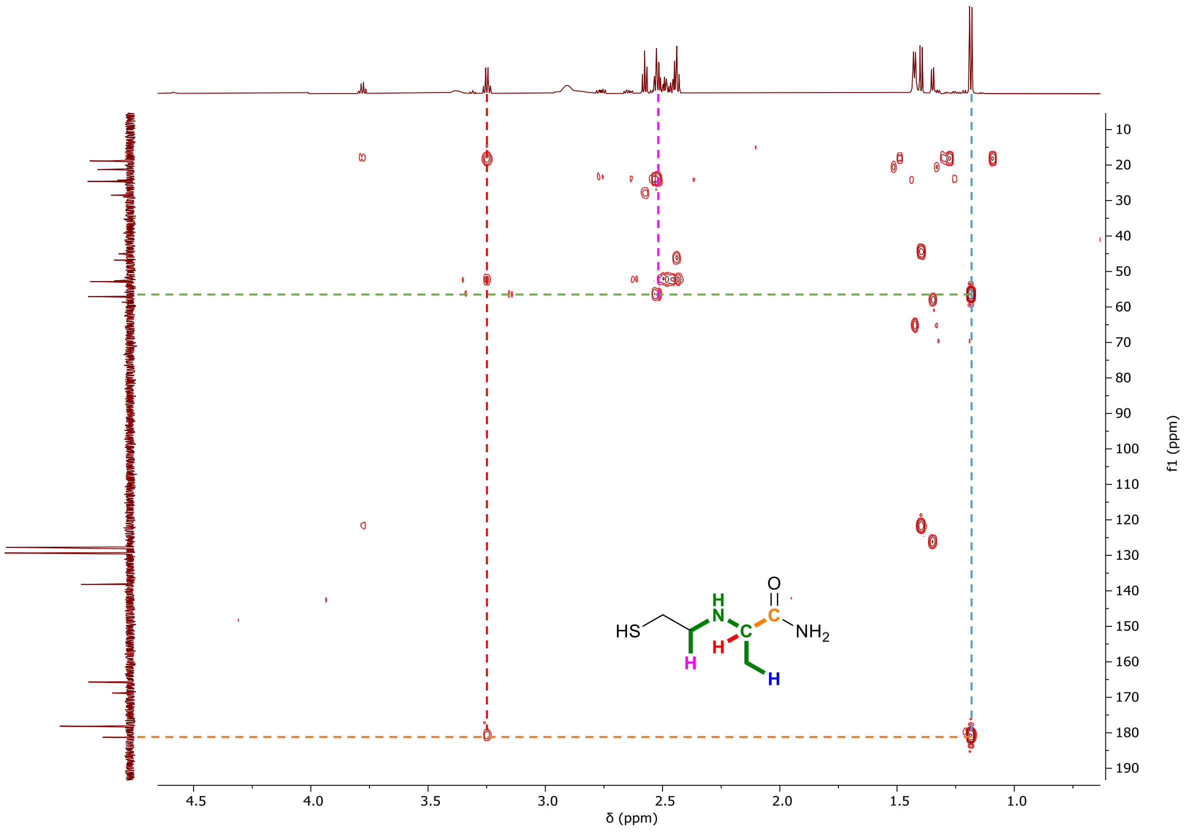

Figure S17: ^1H– ^{13}C HMBC (^1H: 700 MHz [0.0-4.7 ppm], ^{13}C: 175 MHz [0-195ppm]) spectrum to show the diagnostic $^3J_{CH}$ and $^2J_{CH}$ at pH 13.0, that is characteristic for **28_A**

Thiazolidine **12b** (50 mM), NaCN (160 mM) and MSM (25 mM; internal standard) were dissolved in H_2O (2 mL) at pH 13 and then the resulting solution was heated at 60 °C for 24 hours. Excess TCEP·HCl and D_2O (10%) was added to the solution before ^{1}H NMR spectra acquisition indicated the formation of product **16$_A$** (64%). The identity of **16$_A$** was confirmed by spiking with authentic synthetic standard.

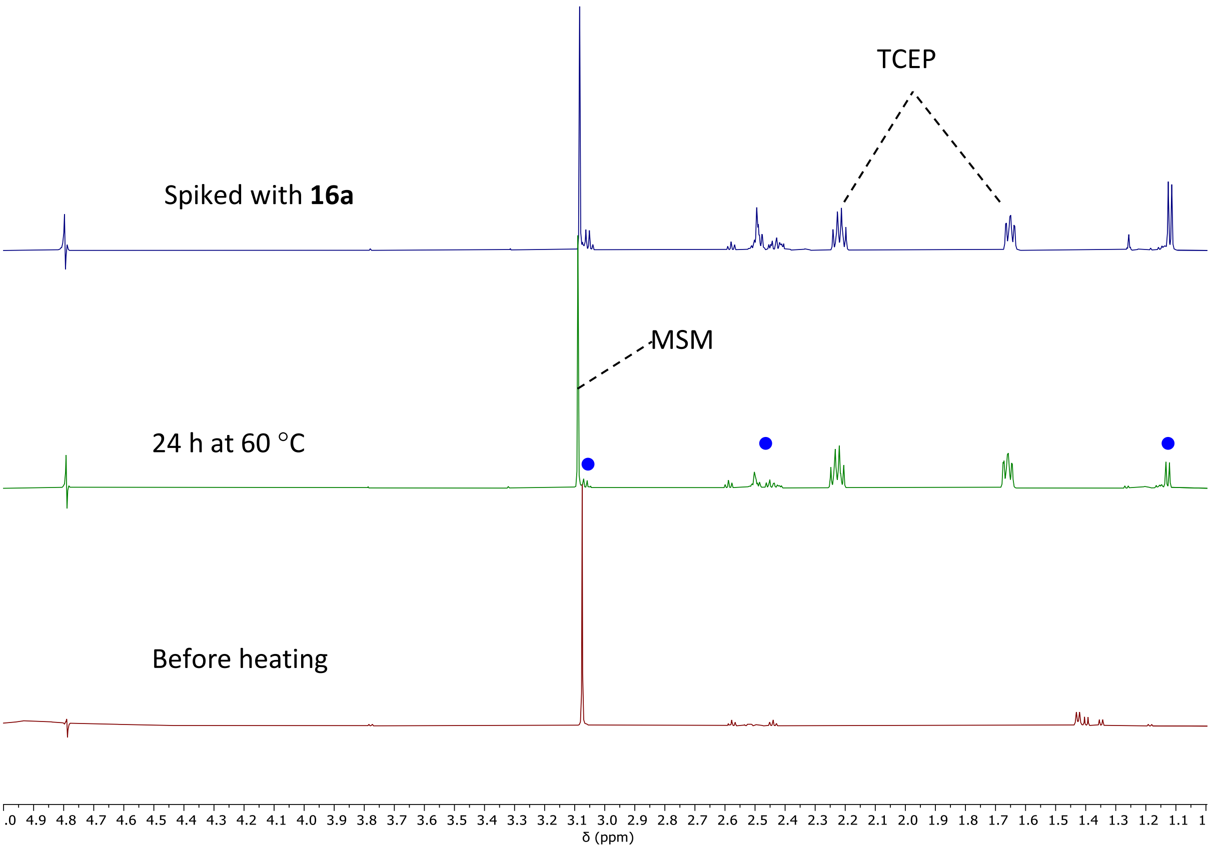

Figure S18: ^{1}H NMR (700 MHz, H_2O/D_2O 9:1, noesygppr1d, 1.0-5.0 ppm) spectra to show the formation of **16$_A$** from thiazolidine (**12b**, 50 mM) and NaCN (160 mM), with MSM (25 mM; internal standard) at pH 13 and at 60 °C. 24 hour ^{1}H NMR spectrum was recorded following TCEP·HCl addition.

Synthesis of **17$_G$**:

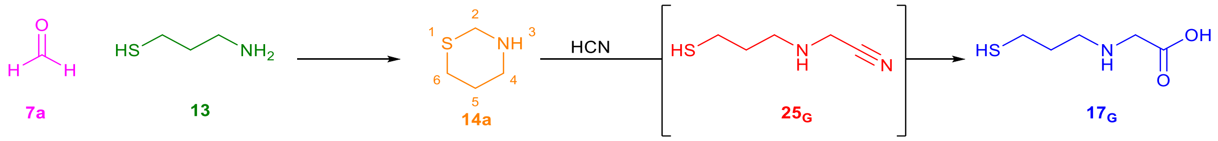

Homocysteamine **13** (200 mM), formaldehyde **7a** (170 mM) and MSM (25 mM; internal standard) were incubated in H_2O/D_2O (9:1, 1 mL) at pH 9 and room temperature. After 30 minute the formation of **14a** (>95%) was observed. NaCN (320 mM) was then added, and the solution was adjusted to pH 13.0 with NaOH (3 M). This solution was incubated at room temperature for 24 hours, and then TCEP (~17 mg, excess with respect to disulfide) was added. The solution was adjusted to pH 9.6 and NMR spectra were acquired. The formation of *N*-thiopropyl-glycine **17$_G$** (62% conversion from **14a**) was observed. The identity of **17$_G$** was confirmed by spiking with authentic synthetic standard.

Compound 17$_G$: ^{1}H NMR (700 MHz, H_2O, pH 9): δ_H 1.80 (m, 2H, (C5)-H), 2.88 (app t, J = 5.7 Hz, 2H, (C6)-H), 2.95 (app t, J = 5.6 Hz, 2H, (C4)-H), 4.02 (s, 2H, (C2)-H). **^{13}C NMR (175 MHz, H_2O, pH 9):** δ_C 26.5 (C5), 27.8 (C6), 46.1 (C4), 48.7 (C2). **HRMS (ESI$^+$):** calcd. for $[C_4H_9NS+H]^+$: 104.0529; Observed 104.0533.

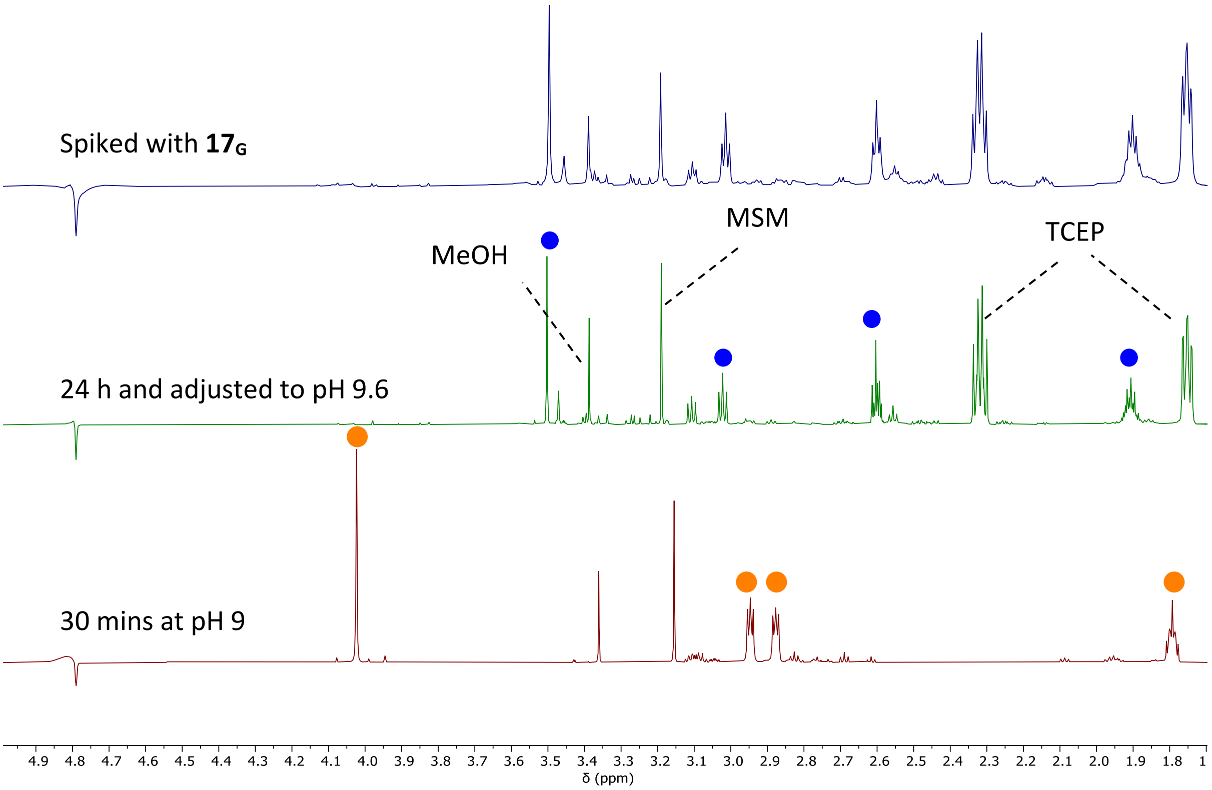

Figure S19: ^{1}H NMR (700 MHz, H_2O/D_2O 9:1, noesygppr1d, 1.0-5.5 ppm) spectra to show the reaction of formaldehyde (**7a**, 170 mM) and homocysteamine (**13**, 200 mM) with MSM (25 mM; internal standard) at pH 9 and room temperature, which yields **14a**. The solution of **14a** (170 mM) was then incubated with NaCN (320 mM) at pH 13 and at 60 °C for 24 hours to yield **17$_G$** (TCEP was added to the reaction and adjusted to pH 9.6 before ^{1}H NMR spectra acquisition), which was identified by spiking with authentic synthetic standard.

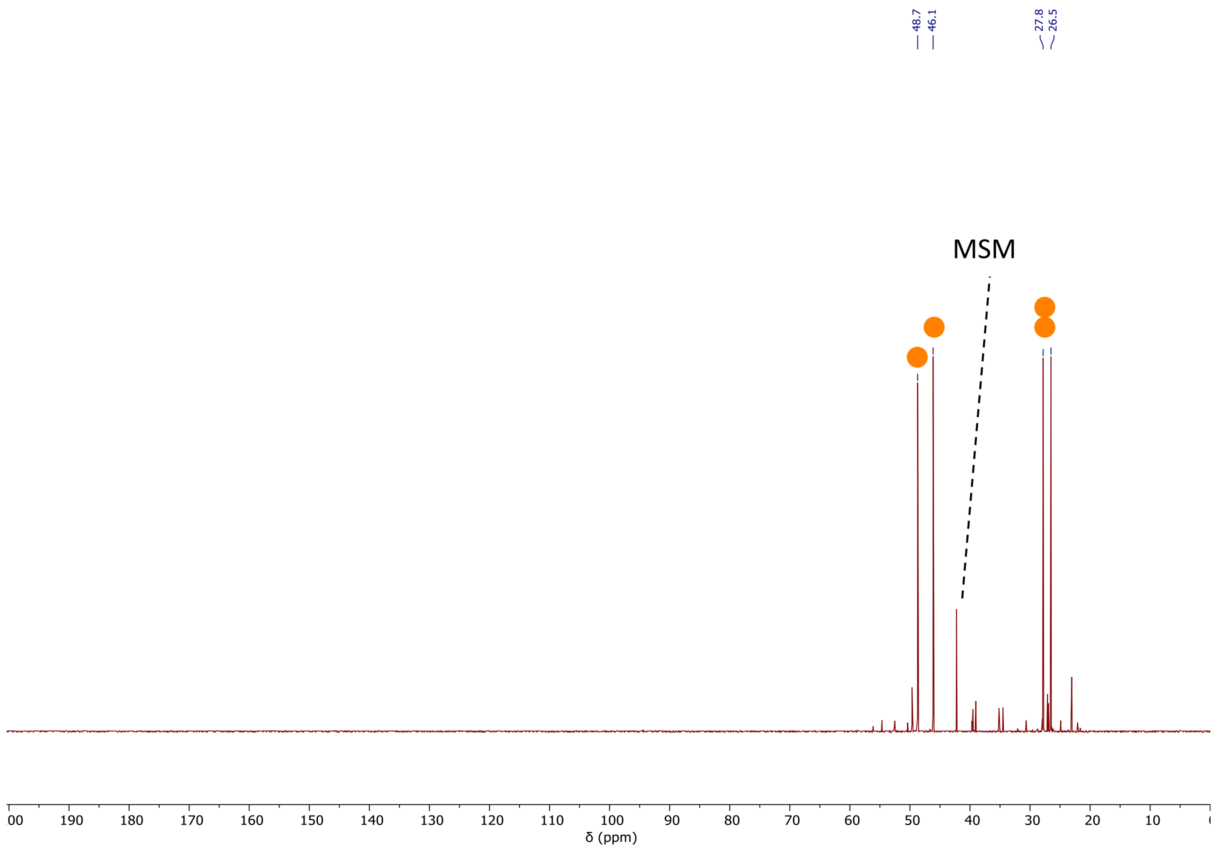

Figure S20: ^{13}C NMR (175 MHz, H_2O/D_2O 9:1, noesygppr1d, 0.0-200 ppm) spectra to show the reaction of formaldehyde (**7a**, 170 mM) and homocysteamine (**13**, 200 mM) with MSM (25 mM; internal standard) at pH 9 and room temperature, which yields **14a**.

Reductive amination of α-keto acids (**18**) with cysteamine (**5**) to yield **16**:

19a synthesis:

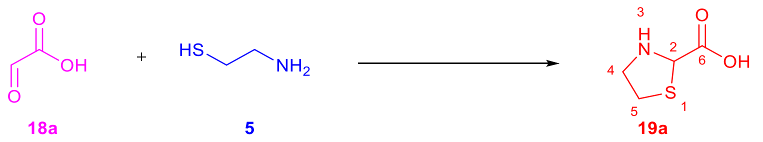

A solution of cysteamine **5** (150 mM, 1.5 equiv.), glyoxylic acid **18a** (100 mmol, 1.0 equiv.) and MSM (12.5 mM) in H_2O (2 mL) at pH 5, 7 or 8.5 (adjusted with 1-8M NaOH/HCl) was incubated at room temperature. The solution was monitored by regular acquisition of ^{1}H NMR spectra. The product, **19a**, was obtained in near quantitative yield (~95%) within 30 mins at pH 7 or pH 8.5, and after 2 hours at pH 5. The identity of the product (**19a**) was confirmed by spiking with authentic synthetic standard.

Compound 19a: ^{1}H NMR (600 MHz, H_2O, pH 7): δ_H 2.95 (m, 1H, (C5)-H), 3.06 (m, 1H, (C5)-H), 3.14 (m, 1H, (C4)-H), 3.64 (ddd, *J* = 12.0, 6.1 & 3.9 Hz, 1H, (C4)-H), 4.84 (obs s, 1H, (C2)-H). **^{13}C NMR (150 MHz, H_2O, pH 7):** δ_C 32.6 (C5), 51.4 (C4), 66.3 (C2), 175.6 (C6). **HRMS (ESI$^+$):** calcd. for $[C_4H_7NO_2S+H]^+$: 134.0270; Observed 134.0270.

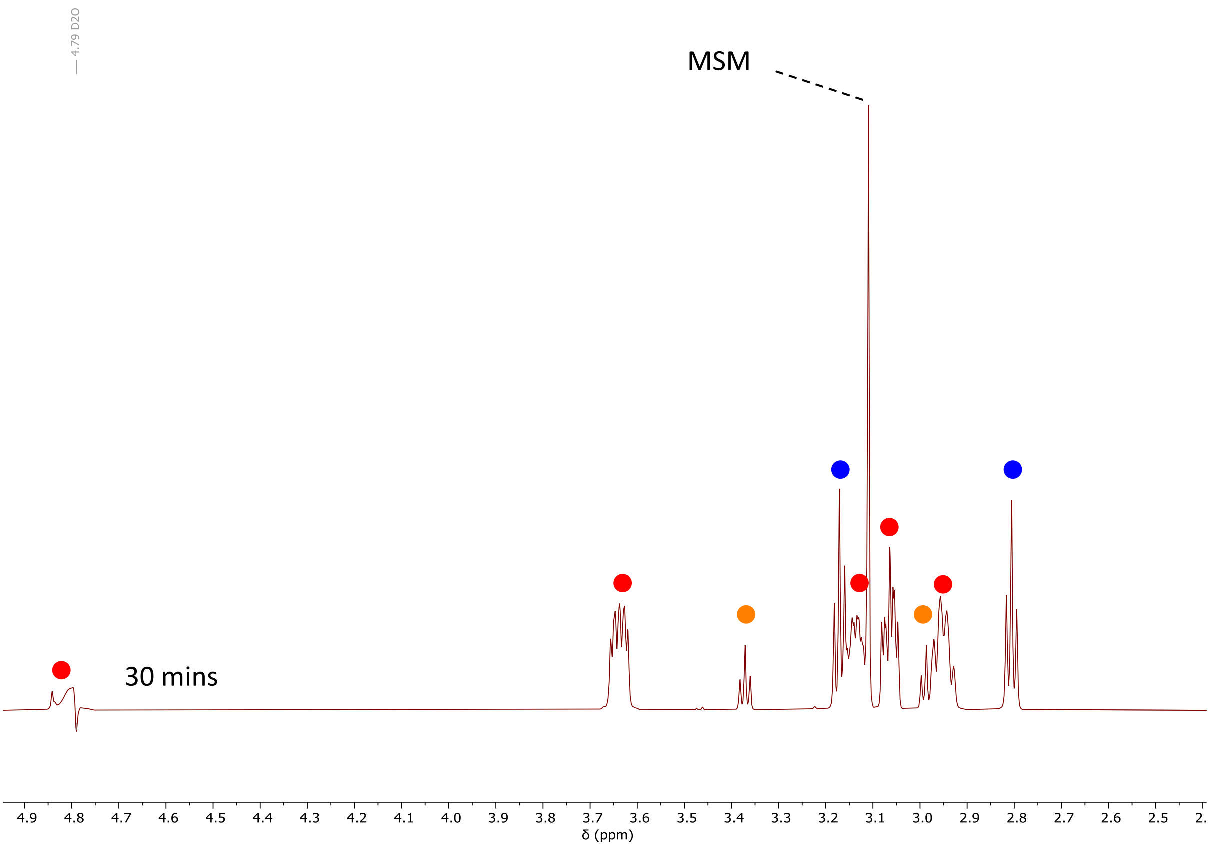

Figure S21: ^{1}H NMR (600 MHz, H_2O, noesygppr1d, 2.4-4.9 ppm) spectrum to show the reaction of cysteamine (**5**, 150 mM) and glyoxylic acid (**18a**, 100 mM) with MSM (12.5 mM; internal standard) at pH 7 and room temperature, which yields **19a**.

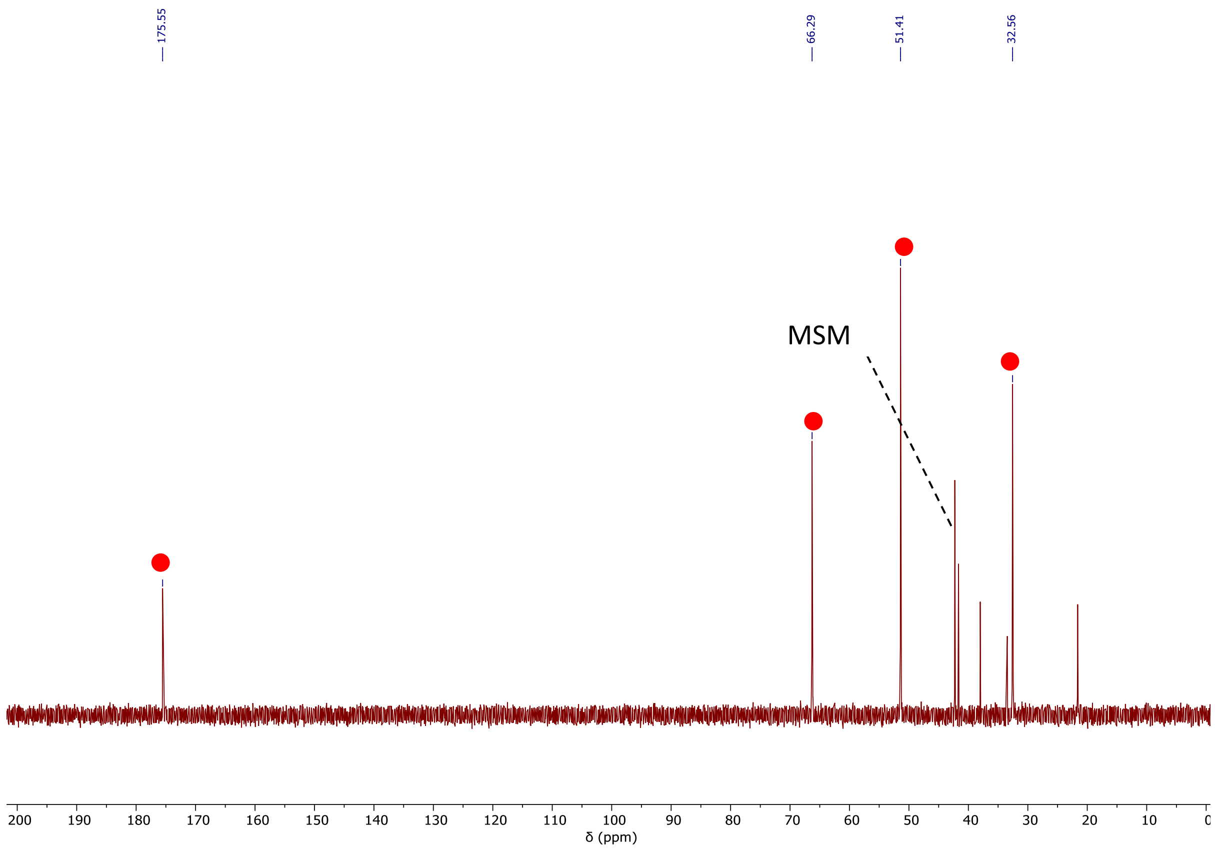

Figure S22: ^{13}C NMR (150 MHz, H_2O, 0.0-200 ppm) spectrum to show the reaction of cysteamine (**5**, 150 mM) and glyoxylic acid (**18a**, 100 mM) with MSM (12.5 mM; internal standard) at pH 7 and room temperature, which yields **19a**.

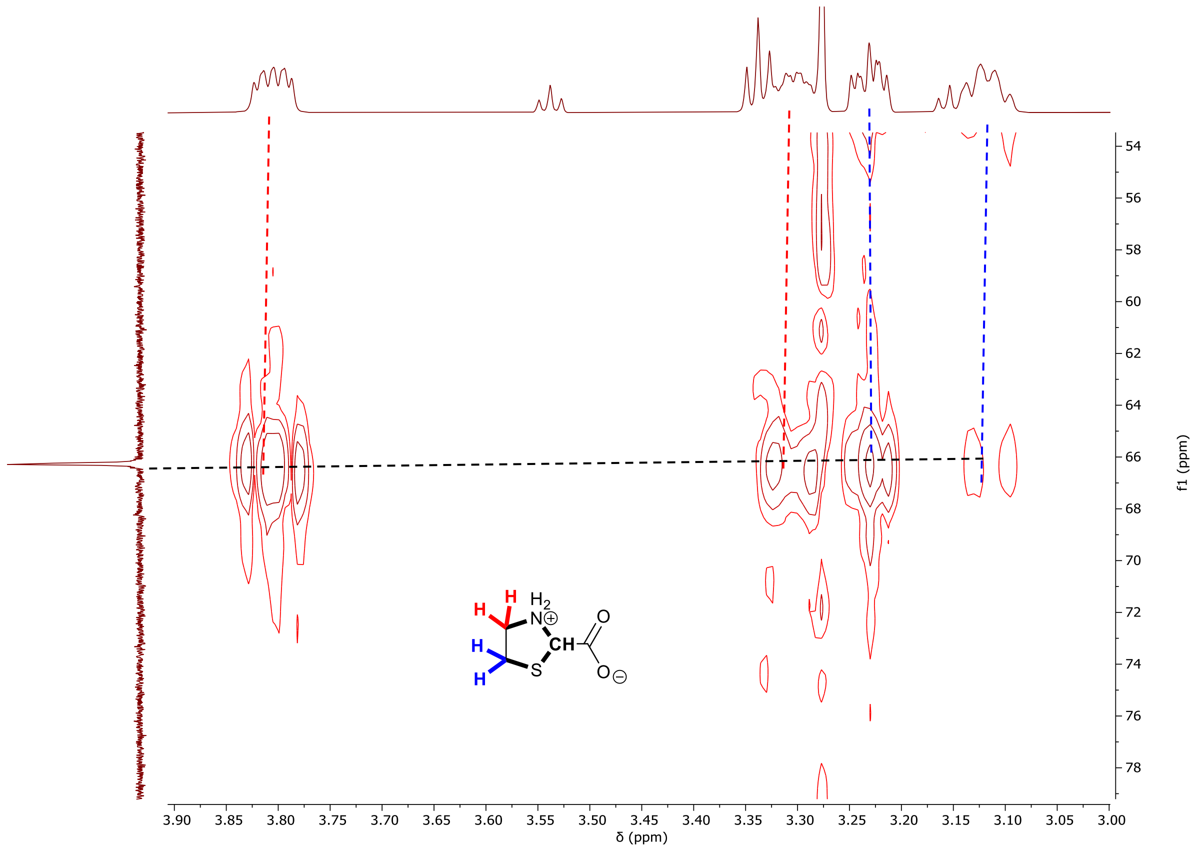

Figure S23: ^{1}H– ^{13}C HMBC (^{1}H: 600 MHz [3.0-3.9 ppm], ^{13}C: 150 MHz [53-79ppm]) spectrum to show the diagnostic $^{3}J_{CH}$ coupling of 2 x CH_2 to α-CH at pH 7, that is characteristic for thiazolidine **19a** formation.

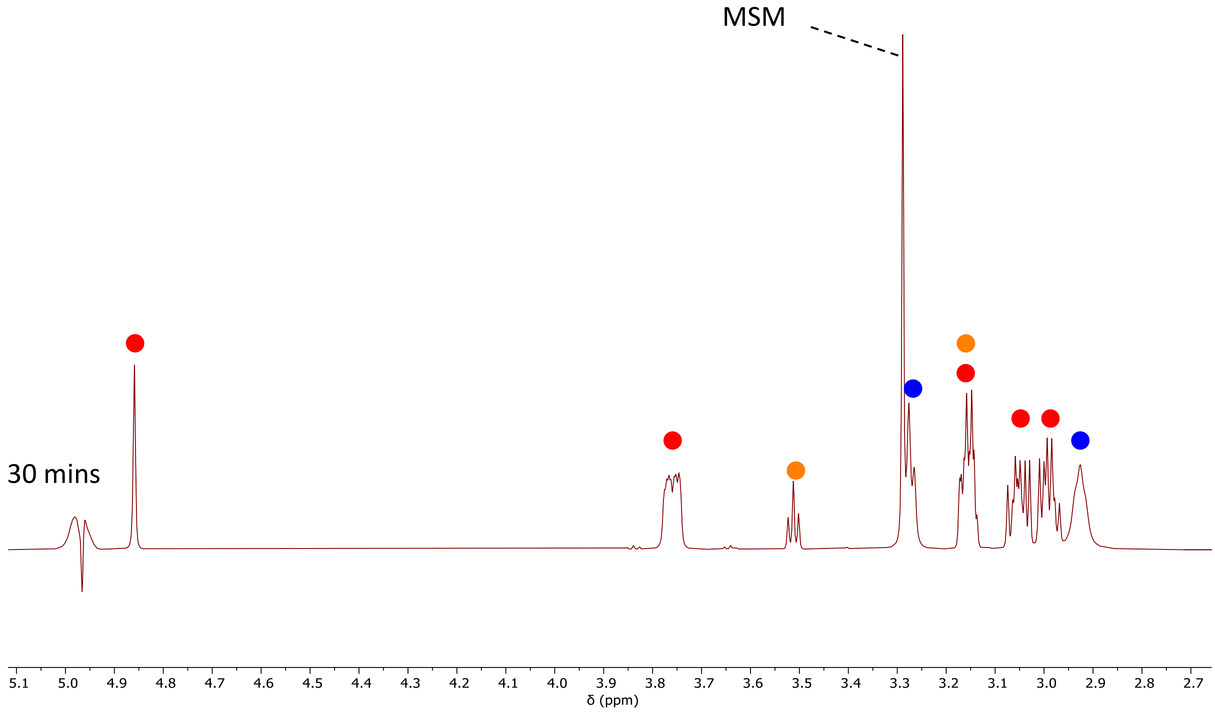

Figure S24: ^{1}H NMR (600 MHz, H_2O, noesygppr1d, 2.65-5.1 ppm) spectrum to show the reaction of cysteamine (**5**, 150 mM) and glyoxylic acid (**18a**, 100 mM) with MSM (12.5 mM; internal standard) at pH 8.5 and room temperature, which yields **19a**.

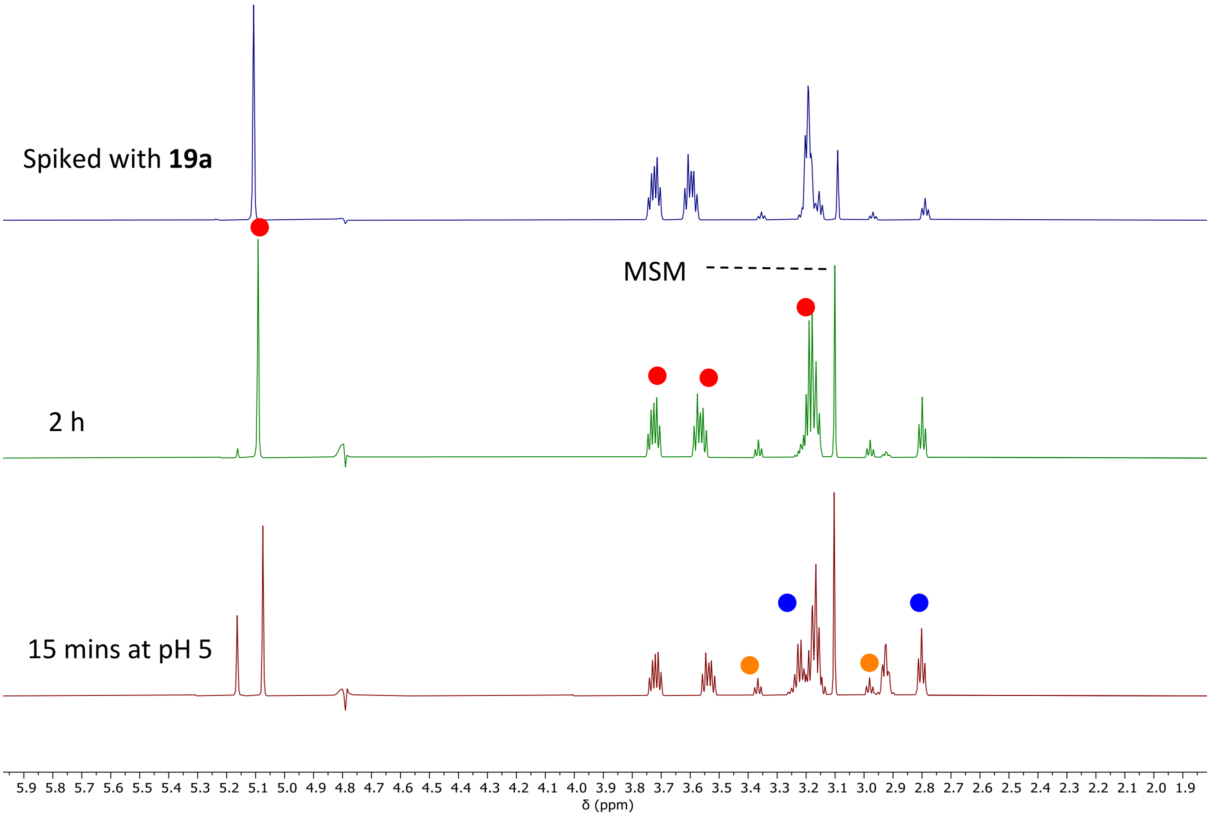

Figure S25: ^{1}H NMR (600 MHz, H_2O, noesygppr1d, 1.8-5.95 ppm) spectrum to show the reaction of cysteamine (**5**, 150 mM) and glyoxylic acid (**18a**, 100 mM) with MSM (12.5 mM; internal standard) at pH 5 and room temperature, which yields **19a** and identified by spiking with authentic synthetic standard.

Selective **19a** synthesis in presence of competing amine nucleophiles:

General Procedure B:

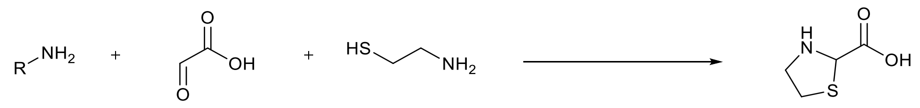

6, 15a-d **18a** **5** **19a**

15a R=H, **15b** R-CH_2CH_3, **15c** R=CH_2CH_2OH, **15d** R=$CH_2CH_2NH_2$ and **6** R=$CH_2CH_2SSCH_2CH_2NH_2$

Glyoxylic acid **18a** (100 mM), cysteamine **5** (150 mM), amine **6** or **15a-d** (147-401 mM) and MSM (25 mM; internal standard) were dissolved in H_2O (0.4-0.8 mL) and the solution was adjusted to pH 7.0 with NaOH/HCl (1-4 M). The solution was then volumetrically increased to 1 mL with H_2O and incubated at room temperature. The reaction was monitored regularly by acquisition of ^{1}H NMR spectra. Selective formation of **19a** was observed within 3 hours.

Ammonia **15a**:

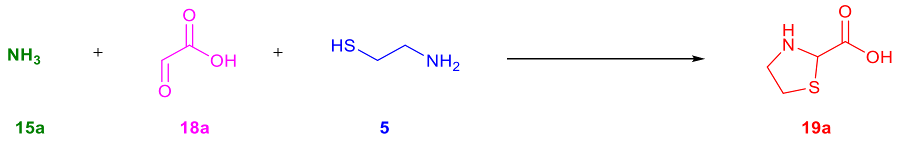

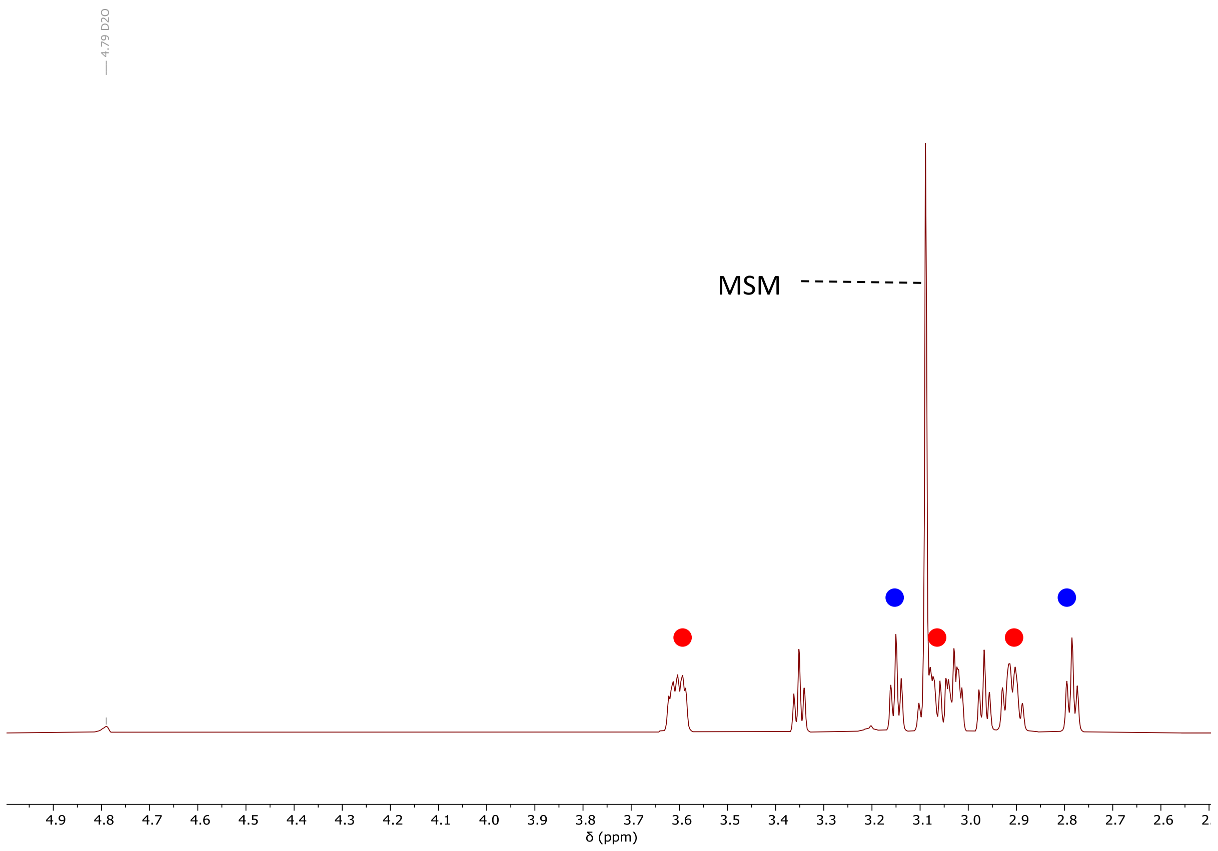

Figure S26: ^{1}H NMR (600 MHz, H_2O, noesygppr1d, 2.5-5.0 ppm) spectrum to show the reaction of cysteamine (**5**, 150 mM) and glyoxylic acid (**18a**, 100 mM) in presence of competing amine nucleophile, ammonia (**15a**, 150 mM), with MSM (25 mM; internal standard) at pH 7 and room temperature, which selectively yields **19a.**

Ethylamine **15b**:

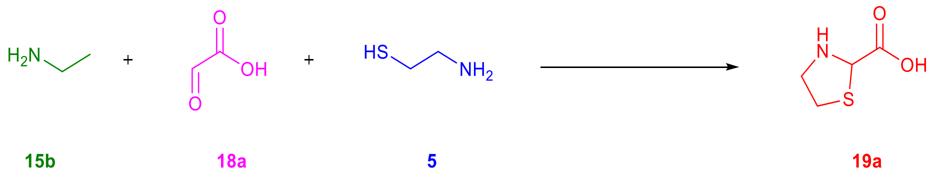

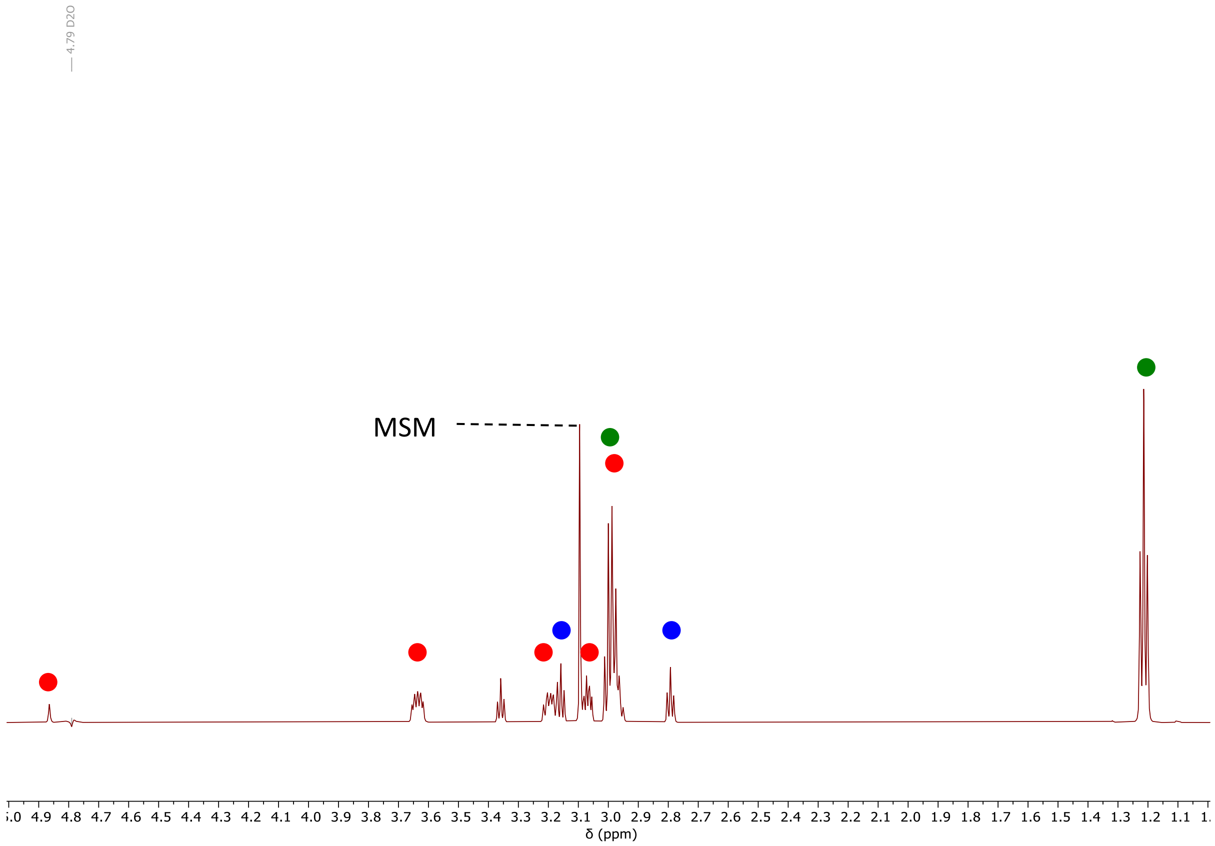

Figure S27: ^{1}H NMR (600 MHz, H_2O, noesygppr1d, 1.0-5.0 ppm) spectrum to show the reaction of cysteamine (**5**, 150 mM) and glyoxylic acid (**18a**, 100 mM) in presence of competing amine nucleophile, ethylamine (**15b**, 147 mM), with MSM (25 mm; internal standard) at pH 7 and room temperature, which selectively yields **19a**.

Ethanolamine **15c**:

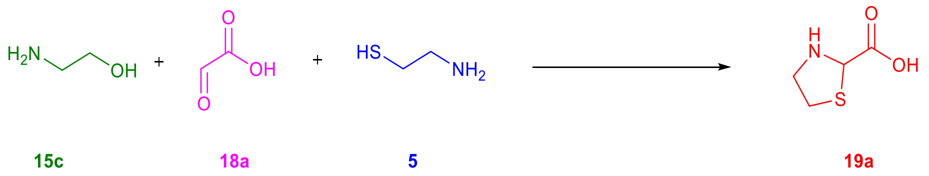

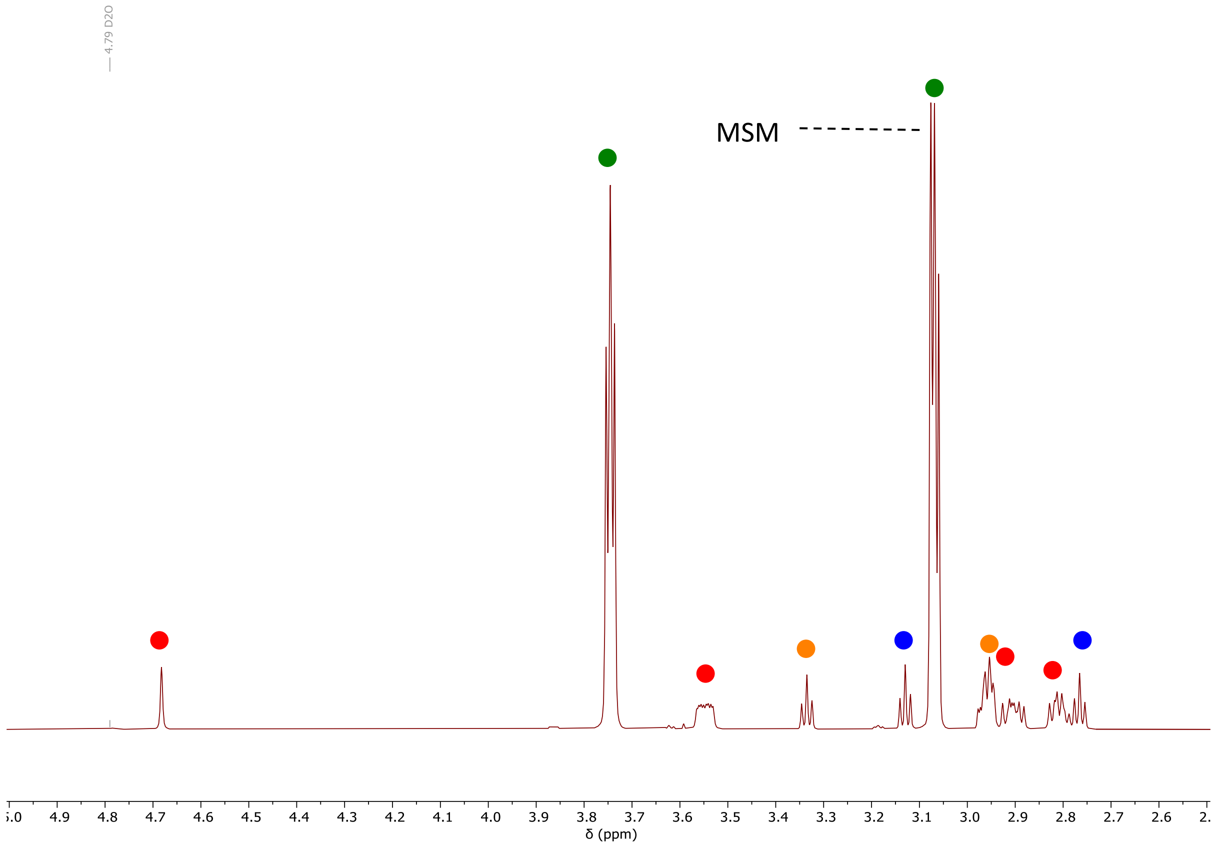

Figure S28: ^{1}H NMR (600 MHz, H_2O, noesygppr1d, 2.5-5.0 ppm) spectrum to show the reaction of cysteamine (**5**, 150 mM) and glyoxylic acid (**18a**, 100 mM) in presence of competing amine nucleophile, ethanolamine (**15c**, 401 mM), with MSM (25 mM; internal standard) at pH 7 and room temperature, which selectively yields **19a**.

Ethylenediamine **15d**:

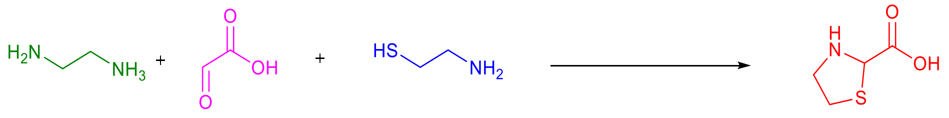

15d **18a** **5** **19a**

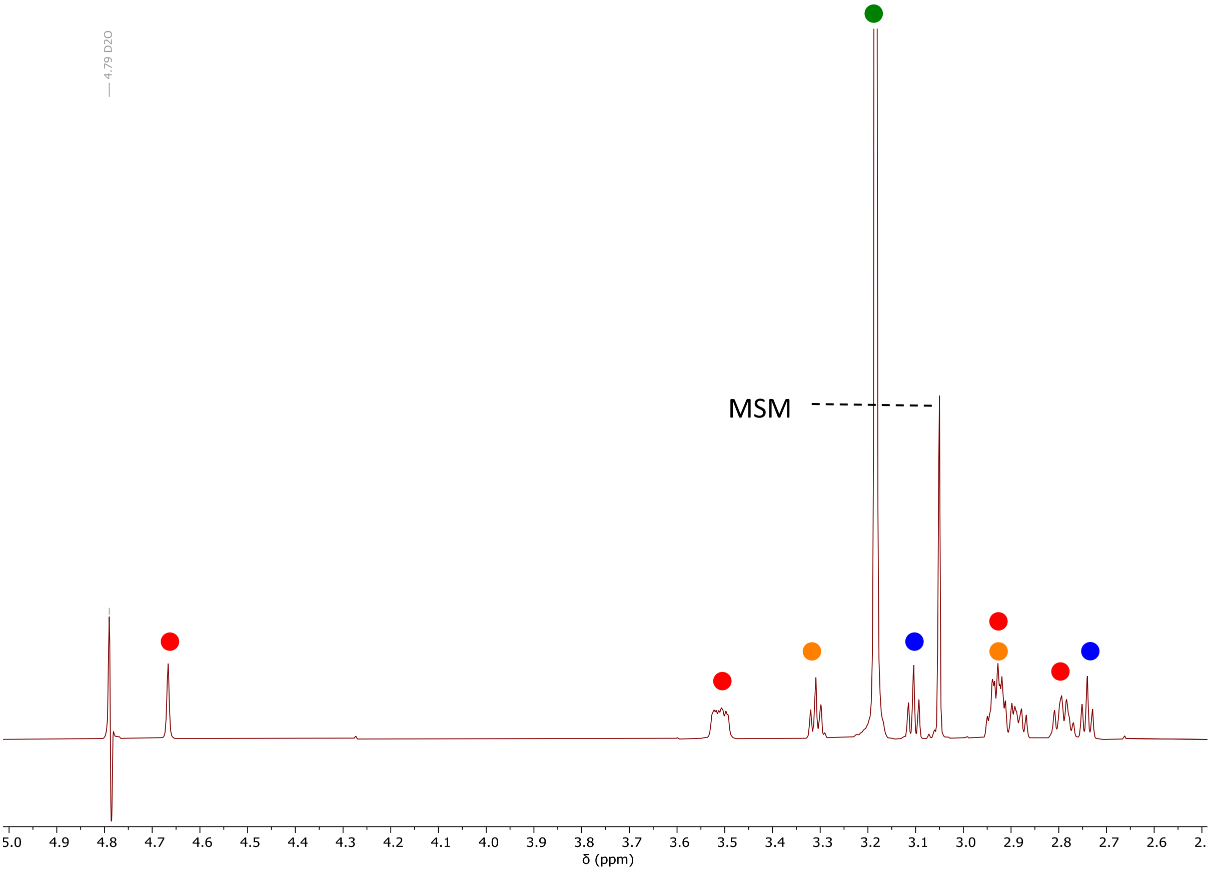

Figure S29: ^{1}H NMR (600 MHz, H_2O, noesygppr1d, 2.5-5.0 ppm) spectrum to show the reaction of cysteamine (**5**, 150 mM) and glyoxylic acid (**18a**, 100 mM) in presence of competing amine nucleophile, ethylenediamine (**15d**, 385 mM), with MSM (25 mM; internal standard) at pH 7 and room temperature, which yields **19a**.

Cystamine **6**:

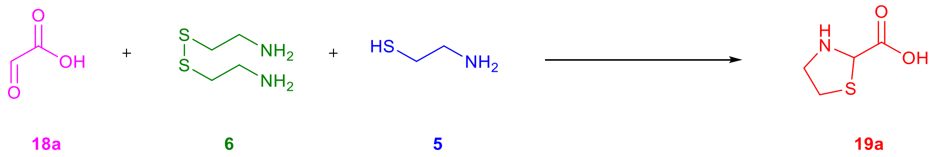

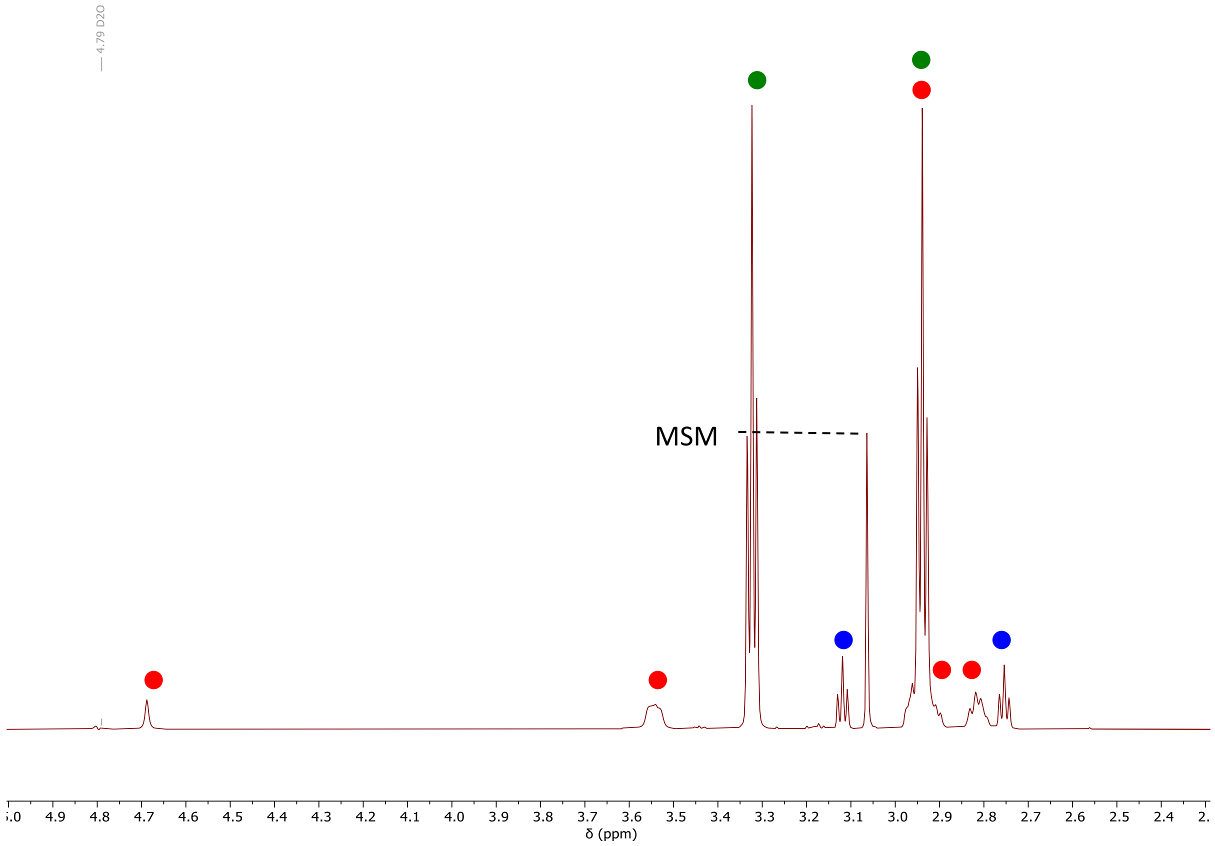

Figure S30: ^{1}H NMR (600 MHz, H_2O, noesygppr1d, 2.5-5.0 ppm) spectrum to show the reaction of cysteamine (**5**, 150 mM) and glyoxylic acid (**18a**, 100 mM) in presence of competing amine nucleophile, cystamine (**6**, 177 mM), with MSM (25 mM; internal standard) at pH 7 and room temperature, which selectively yields **19a**.

19a reduction with Na[CNBH$_3$] to yield **16$_G$**:

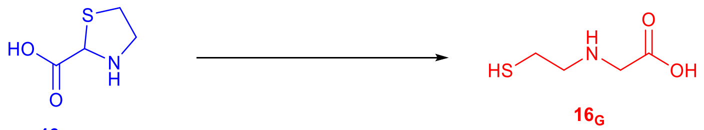

19a   **16$_G$**

19a (0.1 mmol) and MSM (0.05 mmol; internal standard) were dissolved in aqueous buffer at the specified pH. $NaCNBH_3$ (0.5 mmol) was added to the solution and, if required, the pH was adjusted with NaOH/HCl (4 M). The resultant solution was then volumetrically diluted with H_2O to 2 mL, and pH was recorded (Supplementary Table 2). An aliquot of the solution (1 mL) was incubated at 60 °C or room temperature. ^{1}H NMR spectra of each solution were periodically acquired. The formation of *N*-thioethyl-glycine (**16$_G$**) was observed (Supplementary Table 2), and the product identity was confirmed by sample spiking with an authentic synthetic standard.

			Buffer				
**Entry**	**Initial pH**	**Final pH**	**Type**	**Conc./mM**	**T/°C**	**t/h**	**Yield/%**
**1**	3.0	4.2	ABS	200	r.t.	20	27
**2**	3.0	4.0	ABS	500	r.t.	20	40
**3**	3.0	4.7	ABS	500	60	20	63
**4**	4.0	4.5	ABS	200	r.t.	20	36
**5**	4.0	4.5	ABS	200	r.t.	336	90
**6**	4.0	4.6	ABS	200	60	20	75
**7**	4.0	4.2	ABS	500	r.t.	20	31
**8**	4.0	4.9	ABS	500	60	20	60
**9**	4.9	5.1	ABS	200	r.t.	20	35
**10**	4.9	6.5	ABS	200	60	20	9
**11**	5.3	6.3	-	-	r.t.	20	24
**12**	6.1	6.3	CiBS	200	r.t.	20	15

Table S2: Table showing results for reduction of **19a** (50 mM) with $NaCNBH_3$ (250 mM) at various pHs, with and without buffer, at room temperature and 60 °C, with MSM (25 mM; internal standard), which yields **16$_G$**. ABS (acetate buffer), CiBS (citrate buffer).

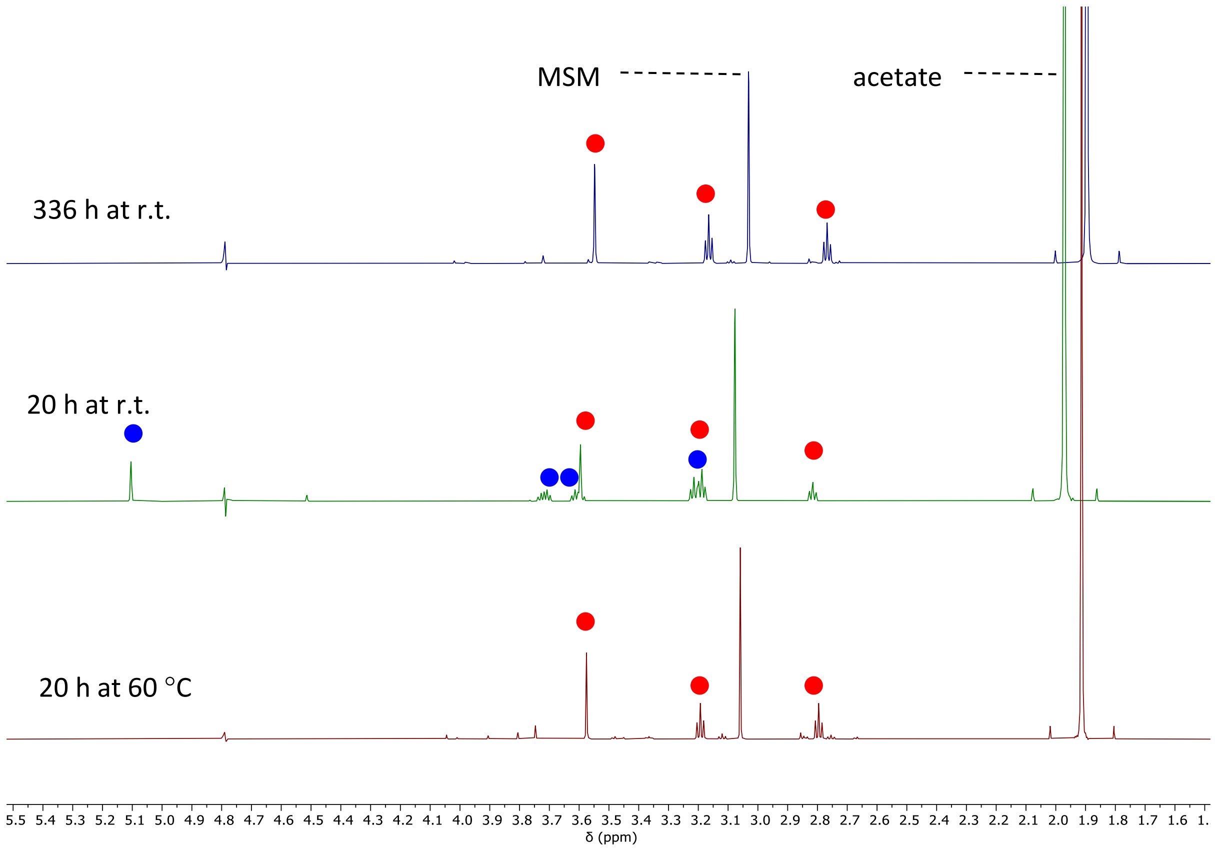

Figure S31: ^{1}H NMR (600 MHz, H_2O, noesygppr1d, 1.5-5.5 ppm) spectra to show the reduction of **19a** (50 mM) with $NaCNBH_3$ (250 mM), with MSM (25 mM; internal standard) in acetate buffer (pH 4.0, 200 mM) to yield **16**$_G$ after 20 hours (room temperature and at 60 °C) and after 336 hours (room temperature).

Selective **19a** reduction with Na[CNBH$_3$] to yield **16$_G$** in presence of competing ammonia (**15a**):

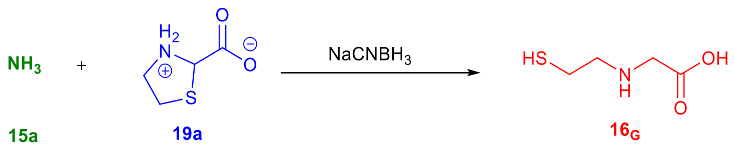

NaCNBH$_3$ (250 mM), NH$_4$Cl **15a**·HCl (100 mM), **19a** (50 mM) and MSM (25 mM; internal standard) in acetate buffer (pH 4, 500 mM) was incubated for 6 days at room temperature. After 6 days, 500 µL of reaction mixture and D$_2$O (100 µL) were combined for ^{1}H NMR spectra acquisition which indicated that **16$_G$** was synthesised in 80% yield with no glycine formation. The identity of **16$_G$** was confirmed by spiking with authentic synthetic standard.

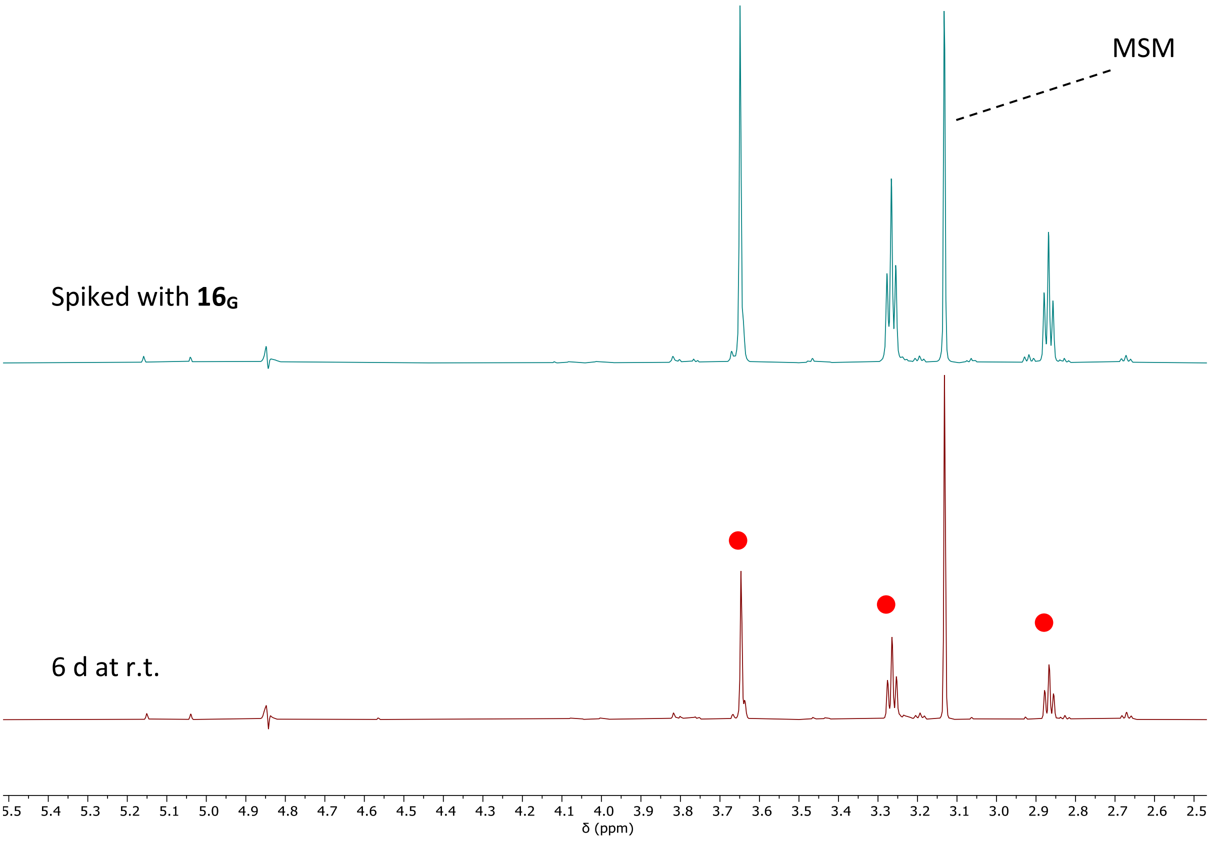

Figure S32: ^{1}H NMR (600 MHz, H$_2$O/D$_2$O 9:1, noesygppr1d, 2.5-5.5 ppm) spectra to show the reaction of **19a** (50 mM) with NaCNBH$_3$ (250 mM), with MSM (25 mM; internal standard) in acetate buffer (pH 4.0, 500 mM) to yield **16$_G$** after 6 days at room temperature with competing amine, NH$_3$ (**15a**, 100 mM).

General procedure C:

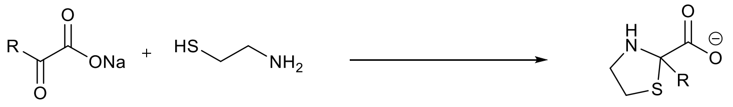

18b-c 5 19b-c

α-Ketoacid **18b-c** (100 mM), cysteamine **5** (150 mM) and MSM (12.5 mM; internal standard) in H_2O (2 mL) were adjusted to pH 7 with 1-4 N HCl/NaOH. The solution was then incubated at room temperature and monitored by regular acquisition of ^{1}H NMR spectra and observed to yield **19b-c**.

Synthesis of **17**$_A$:

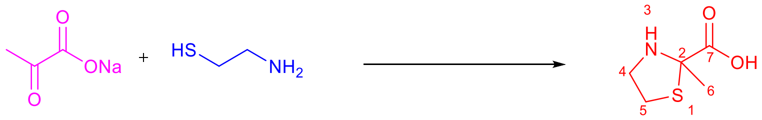

18b 5 19b

Following **General procedure C**, quantitative formation of **19b** (>95%) was observed after 4 hours.

Compound 19b: ^{1}H NMR (600 MHz, H_2O, pH 7): δ_H 1.75 (s, 3H, (C6)-H), 3.08 (obs m, 1H, (C5)-H), 3.20 (app ddd, *J* = 11.2, 6.3 & 4.8 Hz, 1H, (C5)-H), 3.46 (dq, *J* = 12.9 & 6.5 Hz, 1H, (C4)-H), 3.68 (m, 1H, (C4)-H). **^{13}C NMR (150 MHz, H_2O, pH 7):** δ_C 26.2 (C6), 33.2 (C5), 51.3 (C4), 77.0 (C2), 177.6 (C7). **HRMS (ESI$^+$):** calcd. for $[C_5H_9NO_2S+H]^+$: 148.0427; Observed 148.0425.

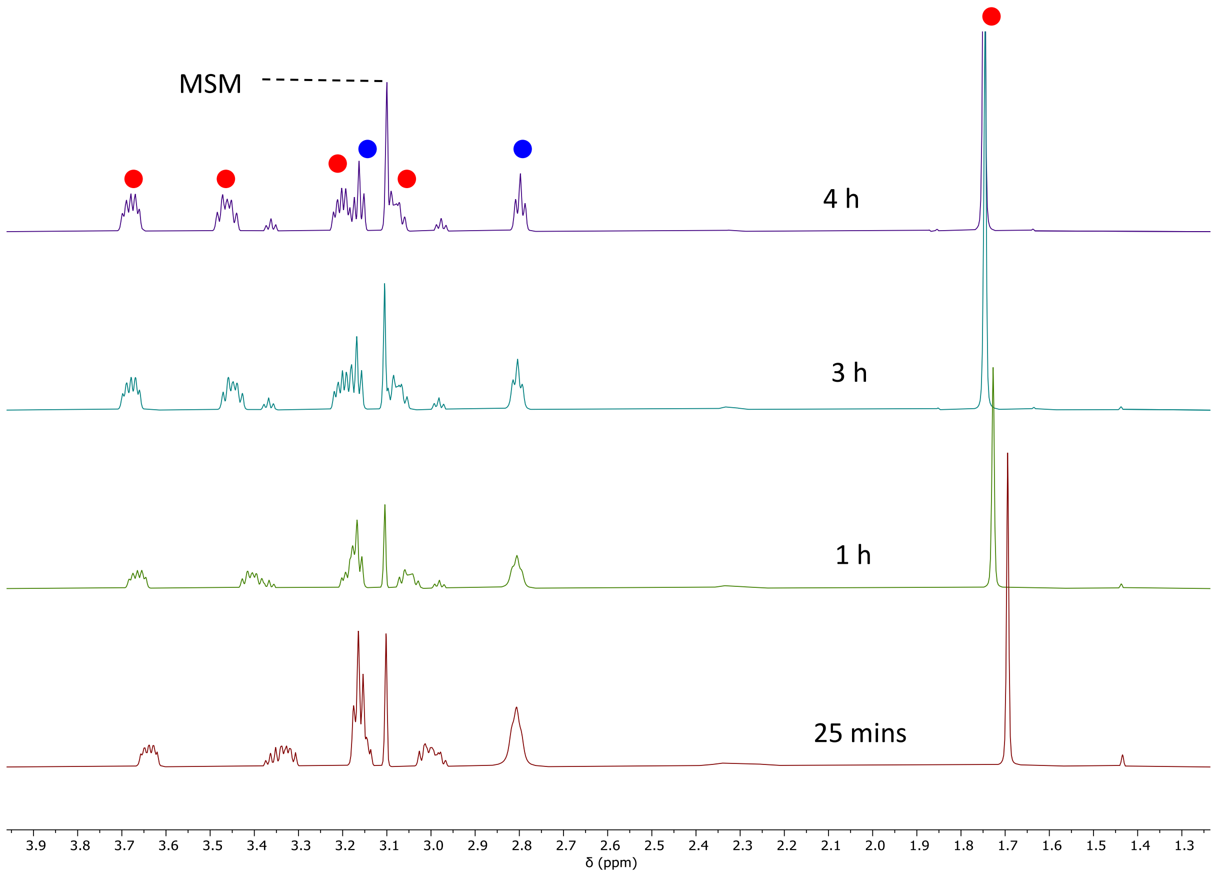

Figure S33: ^{1}H NMR (600 MHz, H_2O, noesygppr1d, 1.35-3.95 ppm) spectra to show the reaction of cysteamine (**5**, 150 mM) and pyruvate (**18b**, 100 mM) with MSM (12.5 mM; internal standard) at pH 7 and room temperature, which yields **19b**.

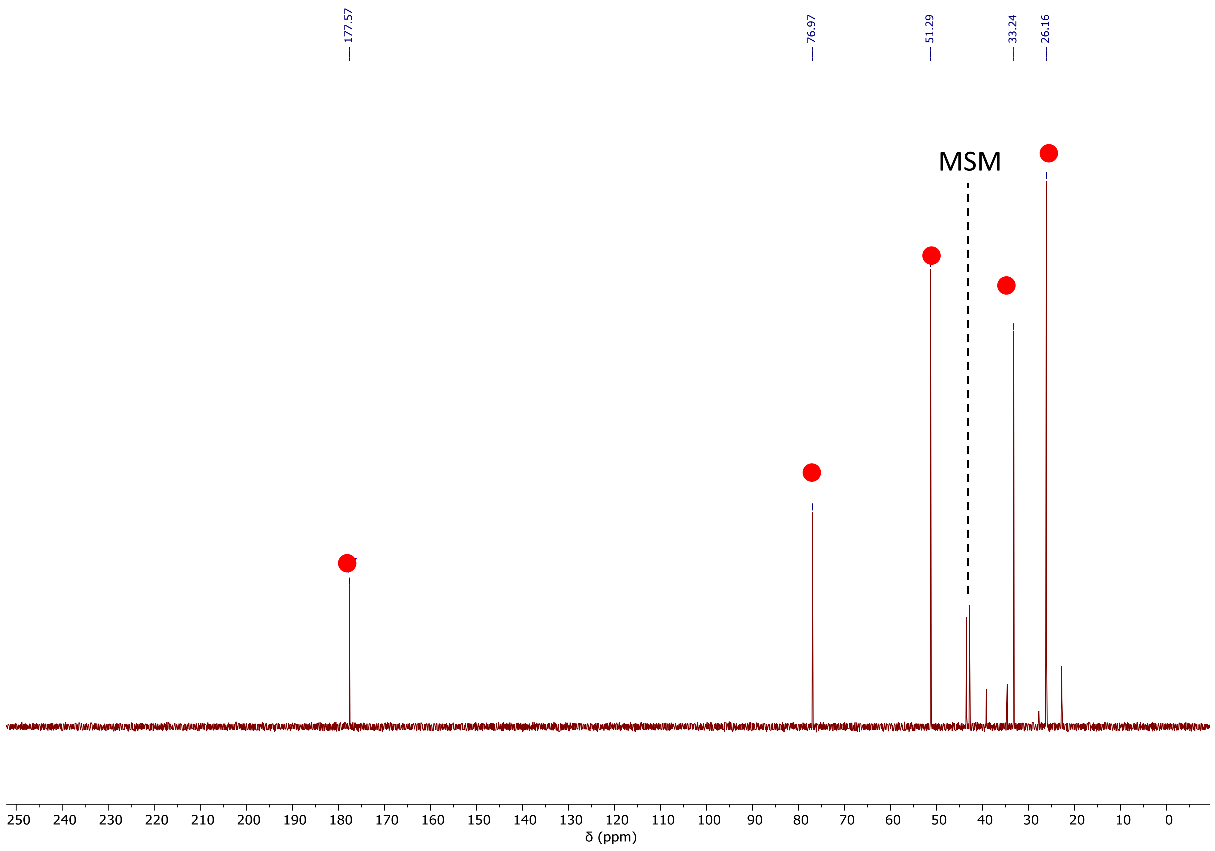

Figure S34: ^{13}C NMR (150 MHz, H_2O, -10.0-250 ppm) spectrum to show the reaction of cysteamine (**5**, 150 mM) with pyruvate (**18b**, 100 mM) with MSM (12.5 mM; internal standard) at pH 7 and room temperature, which yields **19b**.

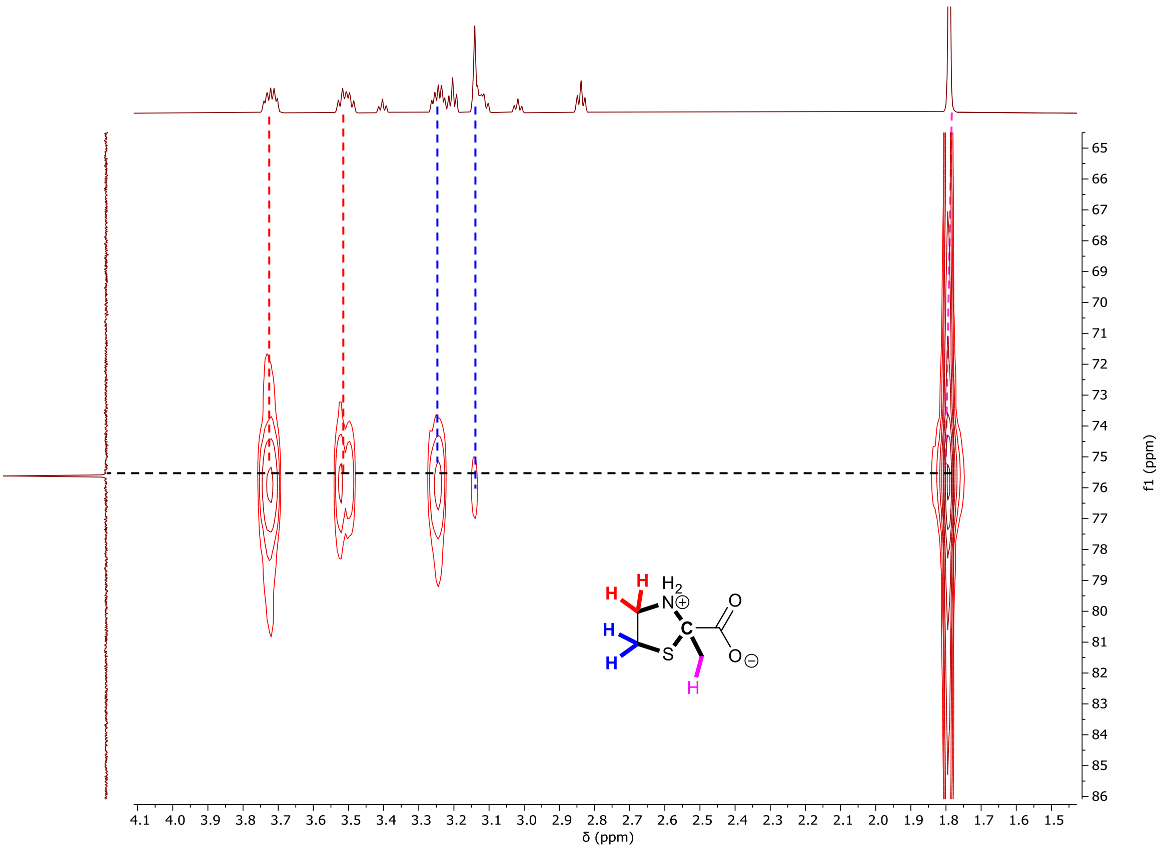

Figure S35: ^{1}H– ^{13}C HMBC (^{1}H: 600 MHz [1.5-4.1 ppm], ^{13}C: 150 MHz [65-86ppm]) spectrum to show the diagnostic $^{3}J_{CH}$ and $^{2}J_{CH}$ coupling of 2 x CH_2 and CH_3 to C-CO_2H at pH 7, that is characteristic of thiazolidine **19b** formation.

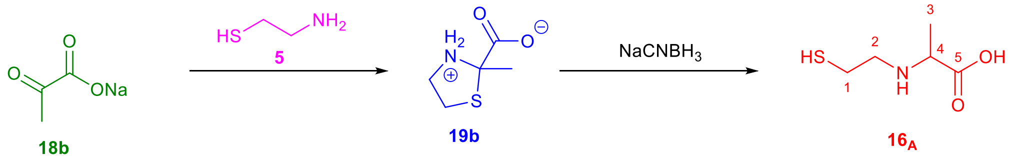

A solution of pyruvate (**18b**, 100 mM, 1 equiv.), cysteamine (**5**, 150 mM, 1.5 equiv.) and MSM (50 mM; internal standard) in H_2O (2 mL) at pH 7 was incubated for 8 hours to form the corresponding thiazolidine **19b** (>95%). The thiazolidine **19b** solution (1 mL) was added to a solution of $NaCNBH_3$ (500 mM) in acetate buffer (pH 4, 1 M, 1 mL). The resultant solution was adjusted to pH 4.0 with NaOH/HCl (1-4M) and incubated at room temperature for 8 days, which yielded **16**$_A$ (77%) over two steps. The identity of the product was confirmed by spiking with authentic synthetic standard.

Compound 16$_A$: ^{1}H NMR (600 MHz, H_2O, pH 7 – partial assignment): δ_H 1.44 (d, *J* = 7.2 Hz, 3H, (C3)-H), 3.22 (dt, *J* = 13.3, 6.7 Hz, 1H, (C2)-H), 3.67 (q, *J* = 7.2 Hz, (C4)-H).

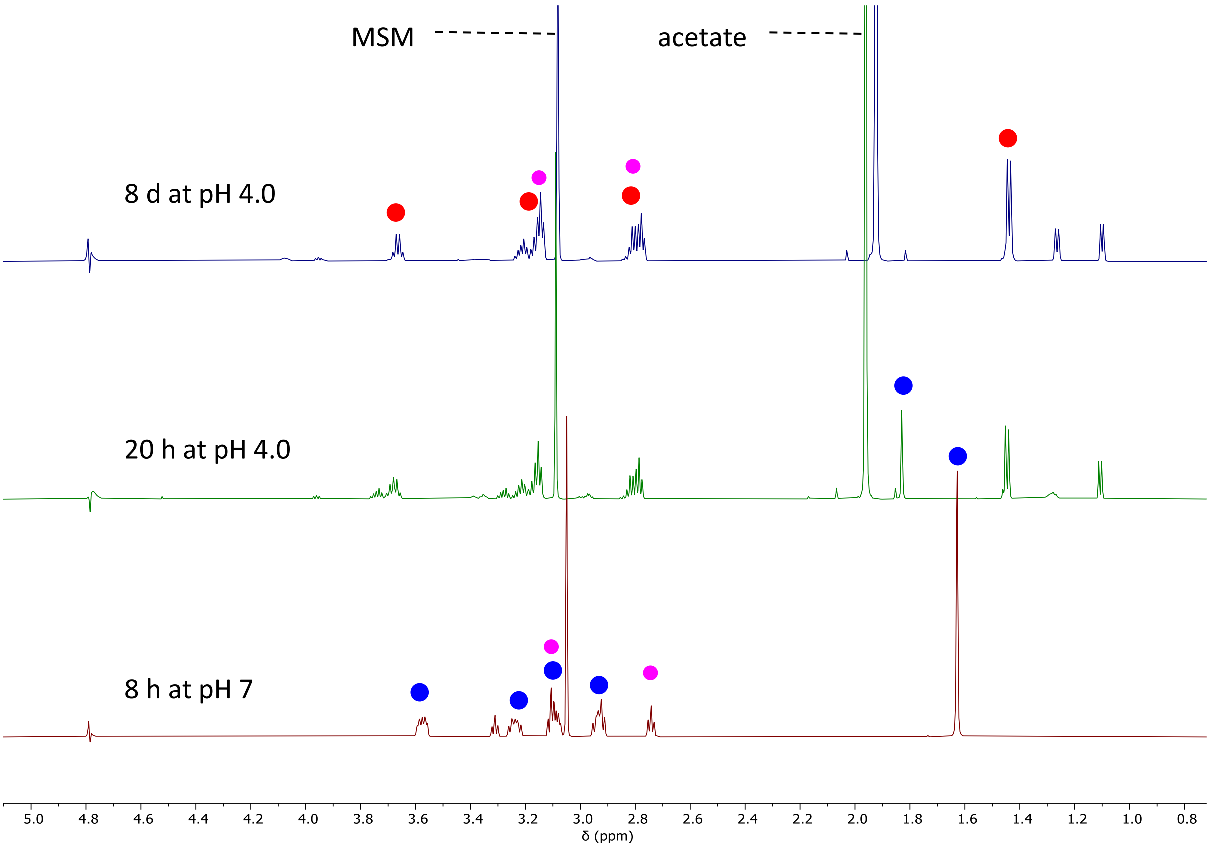

Figure S36: ^{1}H NMR (600 MHz, H_2O, noesygppr1d, 0.7-5.5 ppm) spectra to show the formation of **19b** from cysteamine (**5**, 150 mM) and pyruvate (**18b**, 100 mM), with MSM (50 mM; internal standard), for 8 hours at pH 7.0 (bottom spectrum). This was followed by the reduction with $NaCNBH_3$ (5 equiv.) in acetate buffer (pH 4.0, 500 mM) to yield **16**$_A$ at room temperature (middle and top spectrum).

Synthesis of **16_V:**

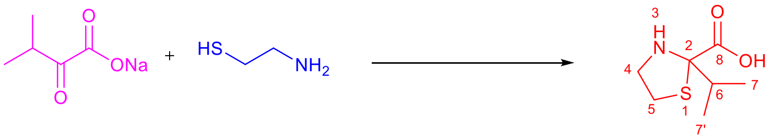

18c **5** **19c**

Following **General procedure C**, quantitative formation of **19c** (>95%) was observed after 15 hours.

Compound 19c: ^{1}H NMR (600 MHz, H_2O, pH 7): δ_H 1.05 (d, J = 7.0 Hz, 3H, (C7)-H), 1.07 (d, J = 7.0 Hz, 3H, (C7')-H), 2.34 (hept, J = 7.0 Hz, 1H, (C6)-H), 3.10 (m, 2H, (C5)-H), 3.55 (dt, J = 11.3, 6.8 Hz, 1H, (C4)-H), 3.63 (dt, J = 11.7, 5.7 Hz, 1H, (C4)-H). **^{13}C NMR (150 MHz, H_2O, pH 7):** δ_C 18.1 (C7), 18.2 (C7') 30.0 (C5), 34.3 (C6), 50.1 (C4), 83.8 (C2), 173.9 (C8). **HRMS (ESI+):** calcd. for $[C_7H_{13}NO_2S+H]^+$: 176.0739; Observed 176.0738.

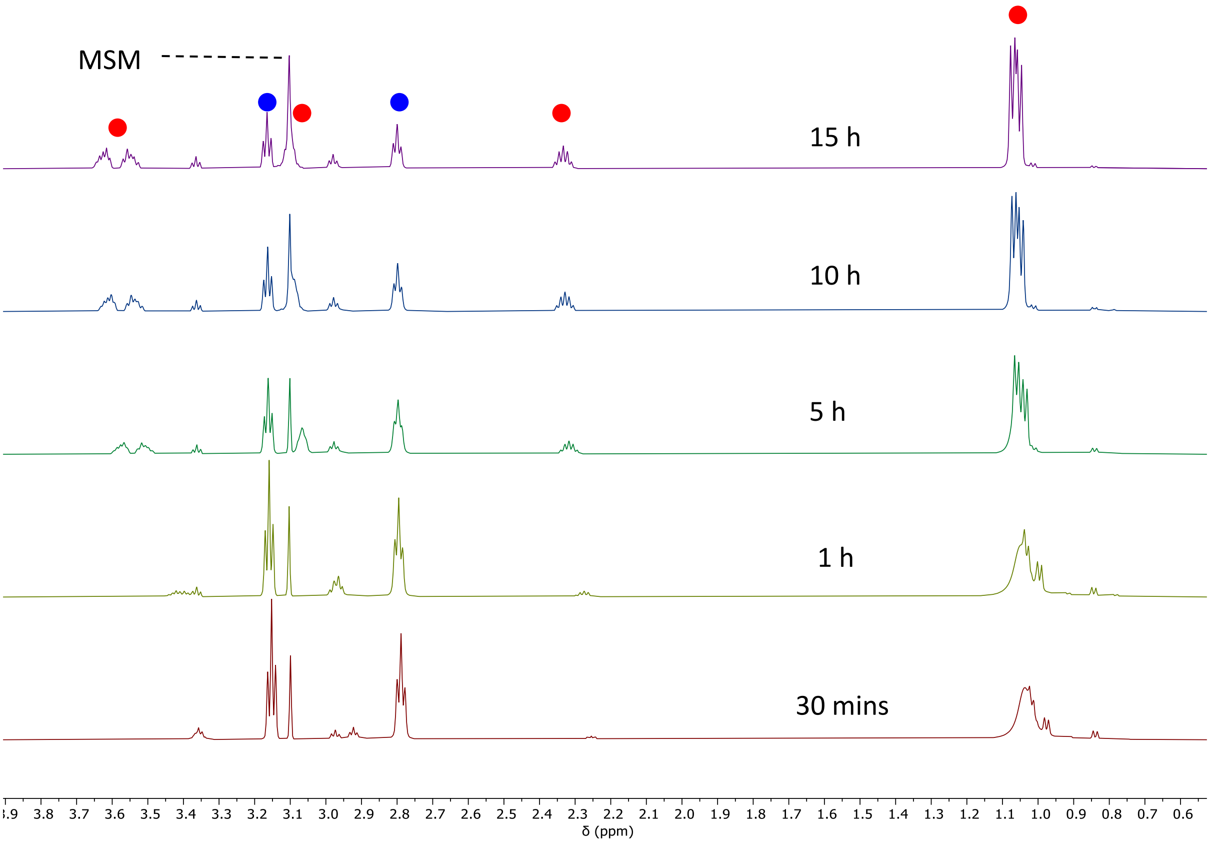

Figure S37: ^{1}H NMR (600 MHz, H_2O, noesygppr1d, 0.5-3.9 ppm) spectra to show the reaction of cysteamine (**5**, 150 mM) and 3-methyl-2-oxobutyrate (**18c**, 100 mM) with MSM (12.5 mM; internal standard) at pH 7 and room temperature, which yields **19c**.

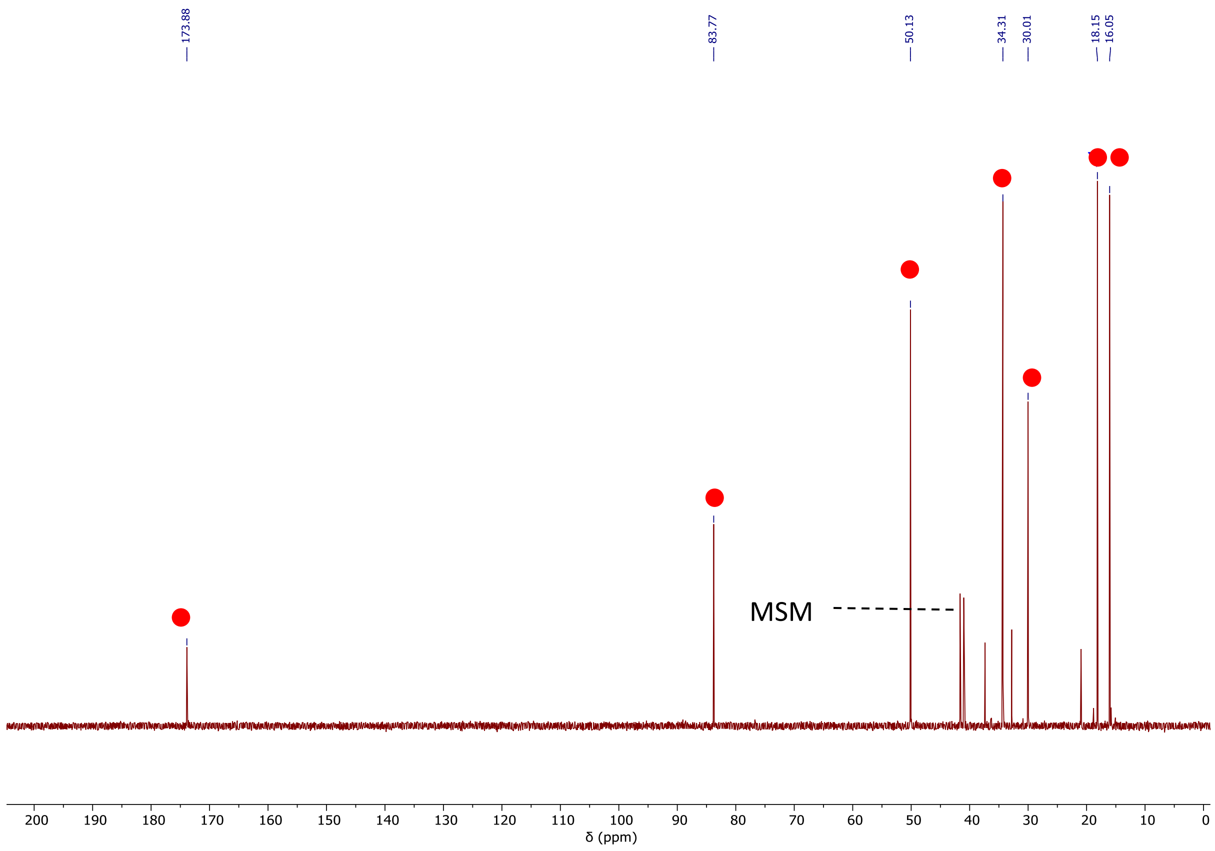

Figure S38: ^{13}C NMR (150 MHz, H_2O, 0.0-205 ppm) spectrum to show the reaction of cysteamine (**5**, 150 mM) and 3-methyl-2-oxobutyrate (**18c**, 100 mM) with MSM (12.5 mM; internal standard) at pH 7 and room temperature, which yields **19c**.

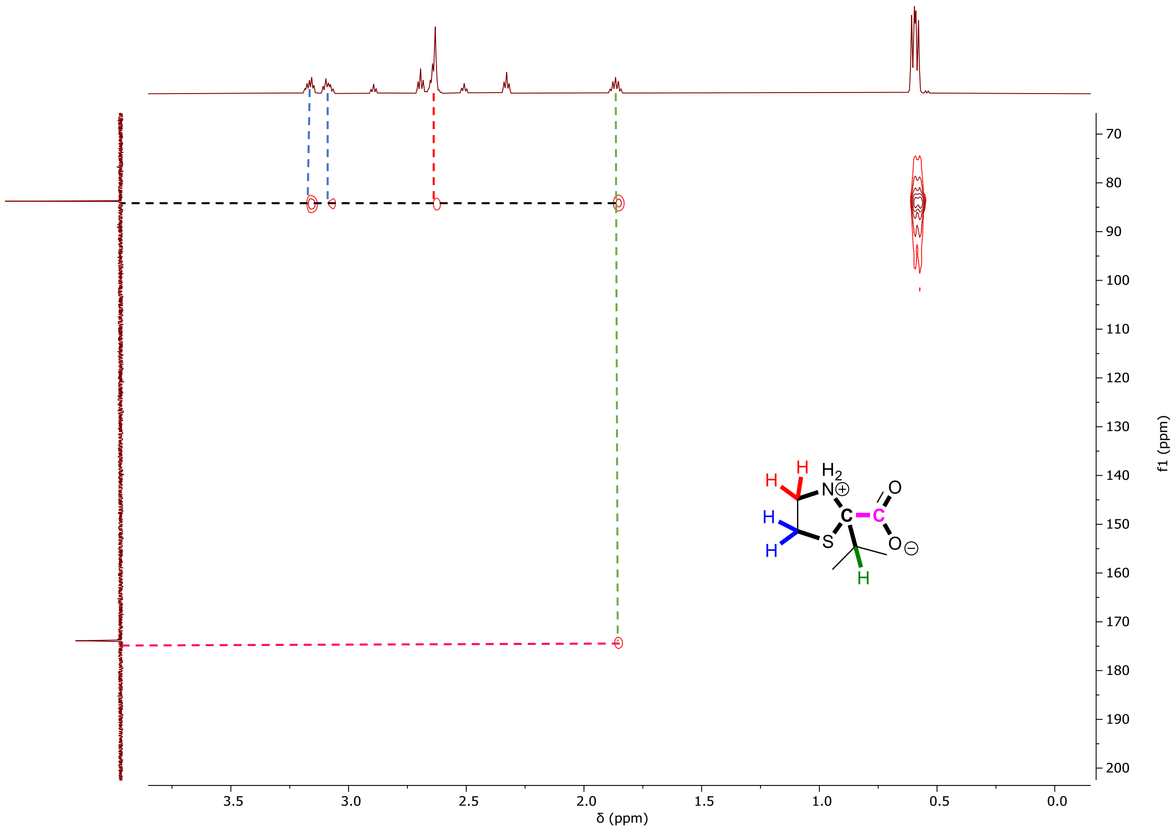

Figure S39: ^{1}H– ^{13}C HMBC (^{1}H: 600 MHz [-0.25-3.8 ppm], ^{13}C: 150 MHz [65-200ppm]) spectrum to show the diagnostic $^{3}J_{CH}$ and $^{2}J_{CH}$ coupling of 2 x CH_2 and CH to C-CO_2H, and CH to -CO_2H at pH 7, that is characteristic of thiazolidine **19c** formation.

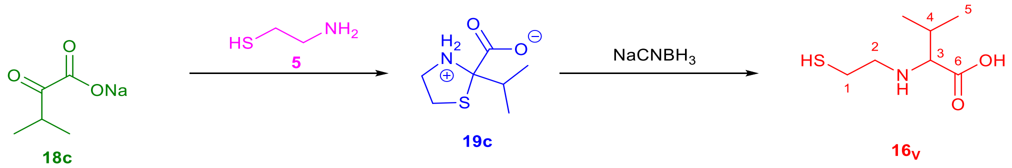

A solution of 3-methyl-2-oxobutyrate **18c** (100 mM, 1 equiv.), cysteamine **5** (150 mM, 1.5 equiv.) and MSM (50 mM; internal standard) in H_2O (2 mL) at pH 7 was incubated for 51 hours. NMR spectra were acquired and the formation of thiazolidine **19c** (>95%) was observed. An aliquot of the resultant thiazolidine solution (1 mL) was added to a solution of $NaCNBH_3$ (500 mM) in acetate buffer (pH 4, 400 mM, 1 mL) and incubated at room temperature for 10 days. NMR spectra were acquired and the formation of amino acid **16$_V$** (68%) over two steps was observed.

Compound 16$_V$: ^{1}H NMR (600 MHz, H_2O/D_2O 9:1 – partial assignment): δ_H 0.95 (app dd, *J* = 6.9, 1.8 Hz, 3H, (C5)-H), 0.99 (app dd, 7.0, 1.8 Hz, 3H, (C5')-H), 2.19 (m, 1H, (C4)-H), 2.84 (app dddd, *J* = 13.4, 7.7, 5.9, 1.9 Hz, 1H, (C1)-H), 3.23 (app dddd, *J* = 13.2, 7.7, 5.8, 1.8 Hz, 1H, (C2)-H), 3.46 (app m, 1H, (C3)-H). **HRMS (ESI$^+$):** calcd. for $[C_7H_{15}O_2NS+H]^+$: 178.0896; Observed 178.0892.

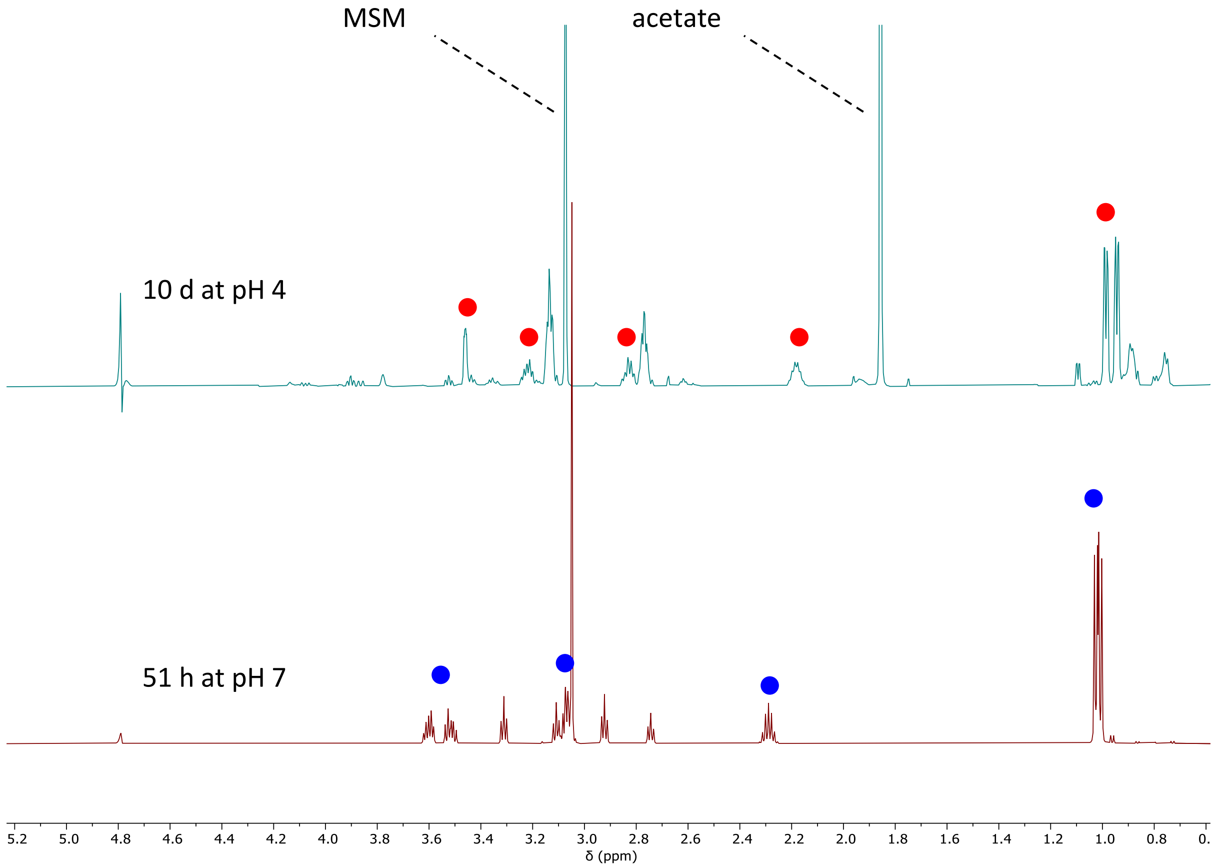

Figure S40: ^{1}H NMR (600 MHz, H_2O, noesygppr1d, 0.7-5.5 ppm) spectra to show the formation of thiazolidine **19c** from incubation of cysteamine (**5**, 150 mM) and 3-methyl-2-oxobutyrate (**18c**, 100 mM) in H_2O (2 mL) with MSM (50 mM; internal standard), for 51 hours at pH 7.0 (lower spectrum). This was followed by the reduction of **19c** with $NaCNBH_3$ (5 equiv.) in acetate buffer (pH 4.0, 200 mM) to yield amino acid **16**$_V$ after 10 days at room temperature (top spectrum).

Strecker Reaction from cyanohydrin (**21**) and cystamine (**6**) to yield **20**:

Synthesis of thiazoline **26** from cysteamine (**5**) and cyanohydrin (**21a**):

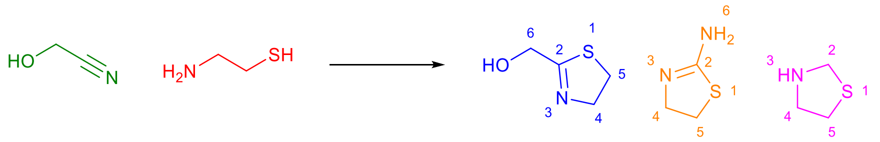

A solution of glycolonitrile **21a** (50 mM), cysteamine (**5**, 75 mM)[b] and MSM (25 mM; internal standard) in H_2O/D_2O (9:1, 5 mL) was incubated at pH 8 – 10 at room temperature. The reaction was monitored periodically by the acquisition of ^{1}H NMR spectra. After 2 days, **26** was obtained in 85% yield at pH 8 and 62% yield at pH 10. The side product **27** (<10%) was observed at pH 8 – 10 due to partial oxidation of cysteamine **5** to cystamine **6**, and **12a** was also obtained in <10% yield at pH 8 and 20% yield at pH 10 (see Supplementary Figures 41-43).

Compound 26: ^{1}H NMR (700 MHz, H_2O/D_2O 9:1, pH 10): δ_H 3.35 (t, *J* = 8.5 Hz, 2H, (C5)-4), 4.20 (tt, *J* = 8.5 & 1.7 Hz, 2H, (C4)-H), 4.43 (t, *J* = 1.7 Hz, 2H, (C6)-H). **^{13}C NMR (175 MHz, H_2O/D_2O 9:1, pH 10):** δ_C 32.9 (C5), 61.8 (C6), 63.8 (C4), 176.7 (C2). **HRMS (ESI$^+$):** calcd. for $[C_4H_7ONS+H]^+$: 118.0321; Observed 118.0320.

Compound 27: ^{1}H NMR (700 MHz, H_2O/D_2O 9:1, pH 10): δ_H 3.39 (t, *J* = 7.5 Hz, 2H, (C5)-H), 3.85 (t, *J* = 7.5 Hz, 2H, (C4)-H). **^{13}C NMR (175 MHz, H_2O/D_2O 9:1, pH 10 – partial assignment):** δ_C 35.4 (C5), 58.4 (C4). **HRMS (ESI$^+$):** calcd. for $[C_3H_6N_2S+H]^+$: 103.0325; Observed 103.0325.

Compound 12a: ^{1}H NMR (700 MHz, H_2O/D_2O 9:1, pH 10): δ_H 2.87 (t, *J* = 6.3 Hz, 2H, (C5)-H), 3.11 (t, *J* = 6.3 Hz, 2H, (C4)-H), 4.08 (s, 2H, (C2)-H). **^{13}C NMR (175 MHz, H_2O/D_2O 9:1, pH 10):** δ_C 33.2 (C5), 52.1 (C4), 53.9 (C2).

---

[b] Commerical cysteamine **5** was partially oxidised **6** (<30%). The reaction of **6** with hydrogen cyanide (HCN) afforded thiazoline **27** (10-20% yield), see Supplementary Figure 41-43 for further details.

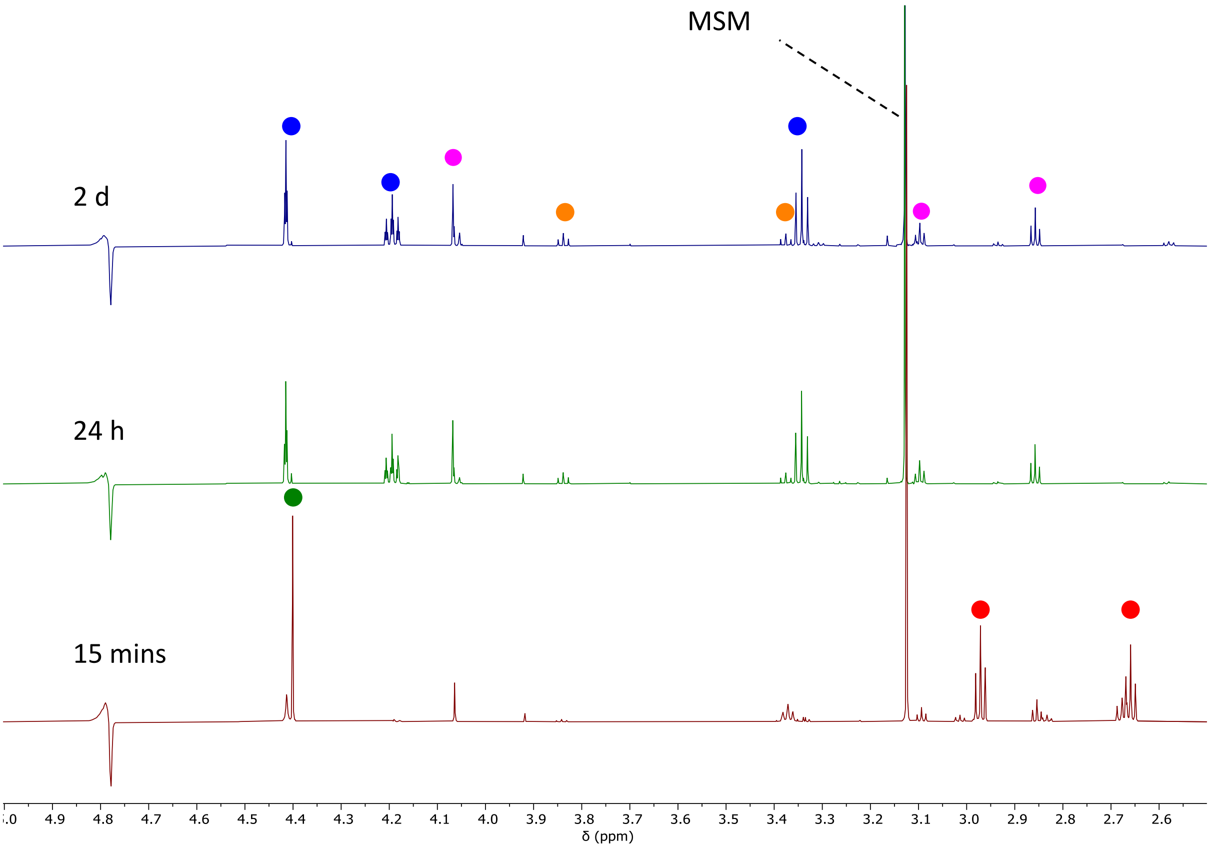

Figure S41: ^1H NMR (700 MHz, H_2O/D_2O 9:1, noesygppr1d, 2.5-5.0 ppm) spectra to show the reaction of cysteamine (**5**, 75 mM) and glycolonitrile (**21a**, 50 mM) with MSM (25 mM; internal standard) at pH 10 and room temperature, which yields **26**, **27** and **12a**. The reaction of cystamine **6** (formed from the oxidation of cysteamine **5**) with hydrogen cyanide (HCN) was observed to afford thiazoline **27**.

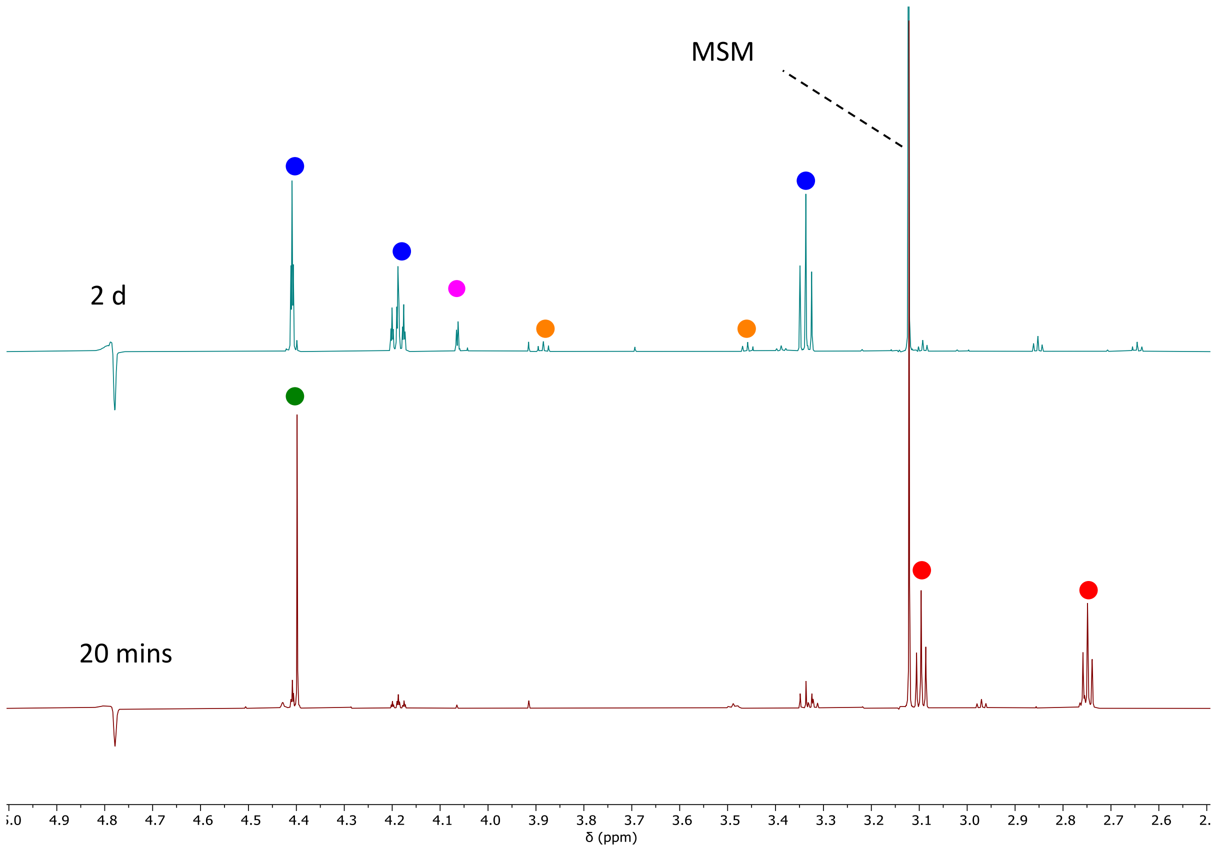

Figure S42: ^{1}H NMR (700 MHz, H_2O/D_2O 9:1, noesygppr1d, 2.5-5.0 ppm) spectra to show the reaction of cysteamine (**5**, 75 mM) and glycolonitrile (**21a**, 50 mM) with MSM (25 mM; internal standard) at pH 8 and room temperature, which yields **26**, **27** and **12a.** The reaction of cystamine **6** (formed from the oxidation of cysteamine **5**) with hydrogen cyanide (HCN) was observed to afford thiazoline **27**.

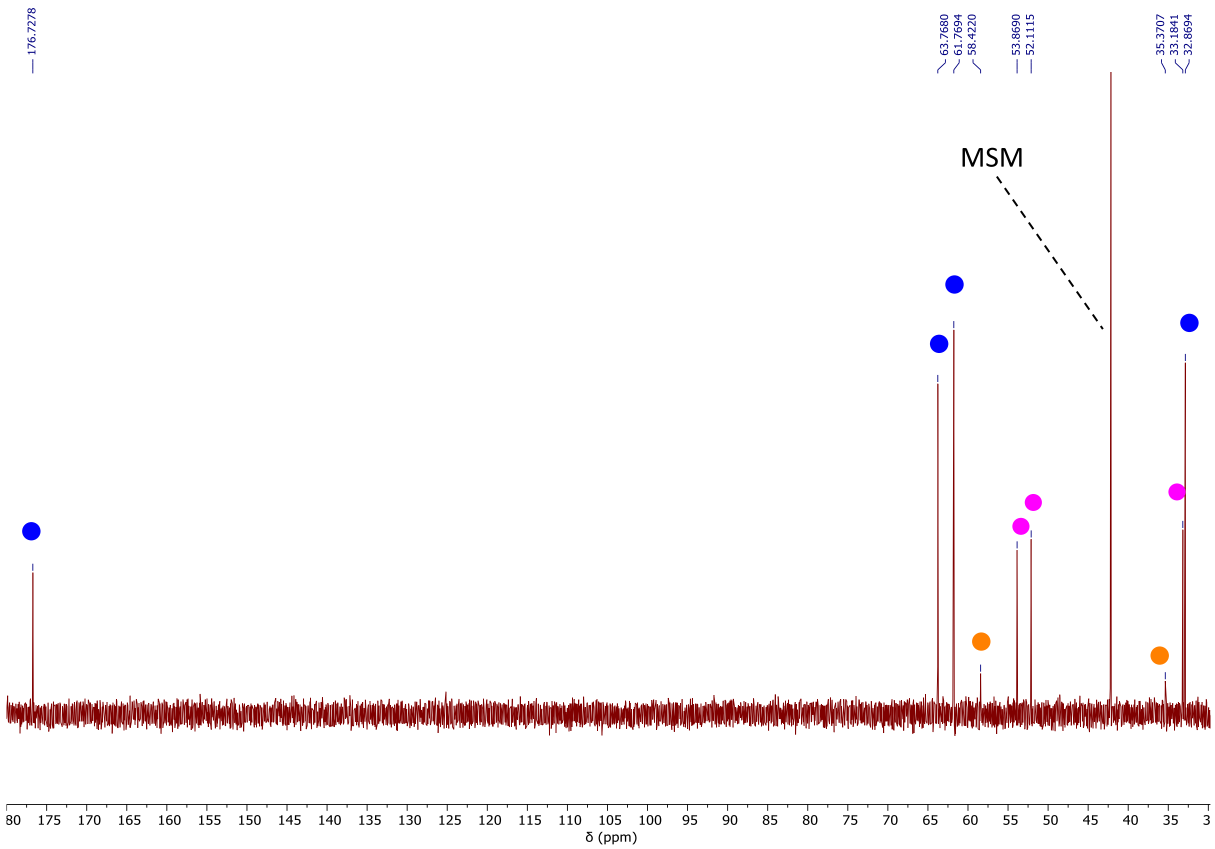

Figure S43: ^{13}C NMR (175 MHz, H_2O/D_2O 9:1, noesygppr1d, 20-200 ppm) spectrum to show the reaction of cysteamine (**5**, 75 mM) and glycolonitrile (**21a**, 50 mM) with MSM (25 mM; internal standard) at pH 10 and room temperature, which yields **26**, **27** and **12a**. The reaction of cystamine **6** (formed from the oxidation of cysteamine **5**) with hydrogen cyanide (HCN) was observed to afford thiazoline **27**.

Synthesis of thiazoline **27** from cysteamine **6** and HCN:

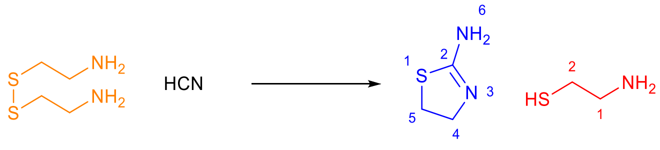

A solution of cystamine **6** (100 mM), NaCN (100 – 300 mM) and MSM (25 mM) in H_2O/D_2O (9:1, 2 mL) was incubated at pH 9.5 and room temperature. The reaction was monitored by periodic acquisition of ^{1}H NMR spectra. After 30 minutes, thiazoline **27** (70 - >95%, confirmed by spiking with authentic synthetic standard) and cysteamine **5** were observed (See Supplementary Figure 44-45).

Compound 27: ^{1}H NMR (600 MHz, H_2O/D_2O 9:1): δ_H 3.38 (t, J = 7.5 Hz, 2H, (C5)-H), 3.82 (t, J = 7.5 Hz, 2H, (C4)-H). **^{13}C NMR (150 MHz, H_2O/D_2O 9:1):** δ_C 34.0 (C5), 55.8 (C4), 168.4 (C2).

Compound 5: ^{1}H NMR (600 MHz, H_2O/D_2O 9:1): δ_H 2.96 (t, J = 7.0 Hz, 2H, (C1)-H), 2.63 (t, J = 6.9 Hz, 2H, (C2)-H). **^{13}C NMR (150 MHz, H_2O/D_2O 9:1):** δ_C 22.6 (C2), 43.9 (C1).

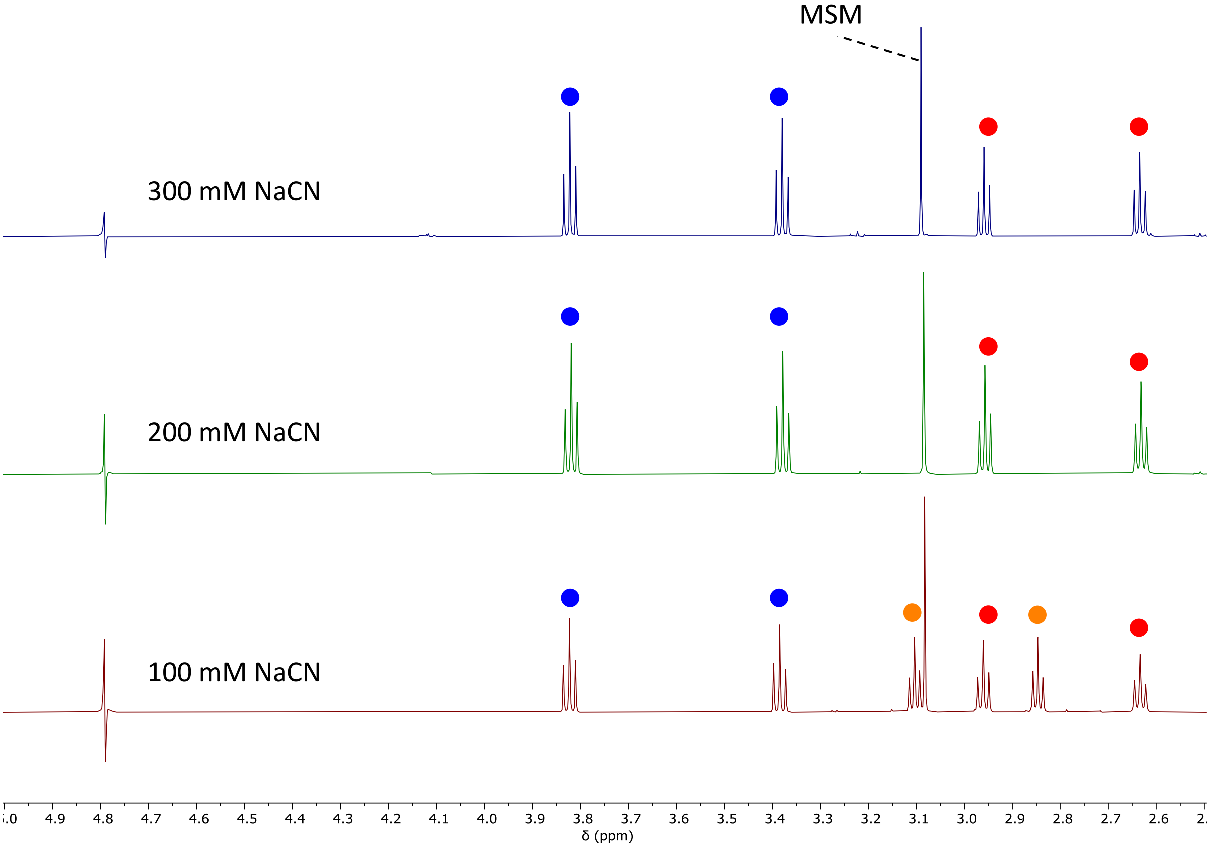

Figure S44: ^{1}H NMR (600 MHz, H_2O/D_2O 9:1, noesygppr1d, 2.5-5.0 ppm) spectra to show the reaction of cystamine (**6**, 100 mM) and NaCN (100-300 mM) with MSM (25 mM; internal standard) at pH 9.5 and room temperature for 30 minutes, which yields **27** and **5**.

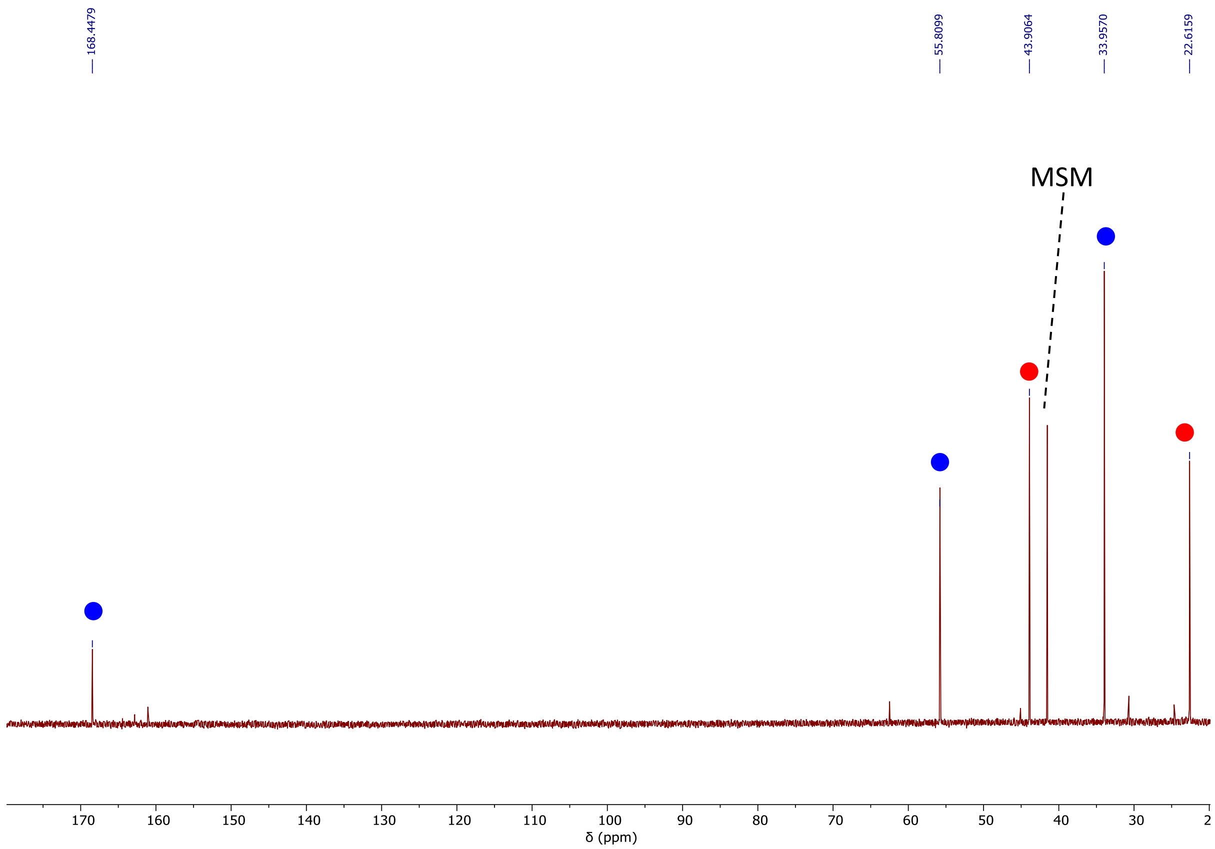

Figure S45: ^{13}C NMR (150 MHz, H_2O/D_2O 9:1, noesygppr1d, 20-180 ppm) spectrum to show the reaction of cystamine (**6**, 100 mM) and NaCN (200 mM) with MSM (25 mM; internal standard) at pH 9.5 and room temperature for 30 minutes, which yields **27** and **5**.

Synthesis of **8**$_G$:

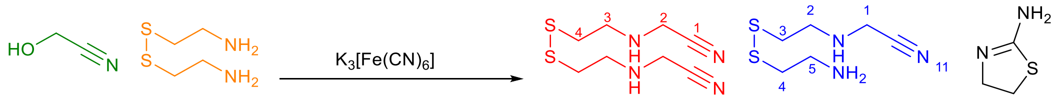

A solution of cystamine **6** (50-200 mM), glycolonitrile **21a** (50-110 mM), $K_3(Fe(CN)_6]$ (0-100 mM) and KHPH (25 mM) in water (2 mL, H_2O/D_2O 9:1) or borate buffer (BBS, 2 mL, 500 mM, H_2O/D_2O 9:1) was incubated at pH 9.5 and room temperature. The reaction was monitored by periodic acquisition of NMR spectra. Initially, the formation of **29**$_G$ was observed within 5 hours followed by its conversion to **20**$_G$; the observed yields of **20**$_G$ and **29**$_G$ are reported in Supplementary Table 3 below:

Compound 29$_G$: ^{1}H NMR (600 MHz, H_2O/D_2O 9:1 – partial assignment): δ_H 3.50 (s, 2H, (C1)-H), 3.11 (t, J = 6.4 Hz, 2H, (C5)-H), 2.88 (t, J = 6.4 Hz, 2H, (C2)-H).

Compound 20$_G$: ^{1}H NMR (600 MHz, H_2O/D_2O 9:1): δ_H 3.58 (s, 4H, (C2)-H), 2.95 (t, J = 6.4 Hz, 4H, (C3)-H), 2.77 (t, J = 6.4 Hz, 4H, (C4)-H). **^{13}C NMR (150 MHz, H_2O/D_2O 9:1):** δ_C 118.7 (C1), 46.2 (C3), 36.5 (C4), 36.1 (C2). **HRMS (ESI$^+$):** calcd. for $[C_8H_{14}N_4S_2+H]^+$: 231.0733; Observed: 231.0728.

Entry	23a/mM	10/mM	$K_3[Fe(CN)_6]$/mM	BBS/mM	Final pH	Yield(20$_G$+29$_G$)/%	ratio 21$_G$:31$_G$
**1**	100	50	-	-	8.5	45	~1:1
**2**	50	50	-	-	9.3	60	-
**3**	50	100	-	-	9.5	57	-
**4**	50	150	-	-	9.4	51	-
**5**	50	200	-	-	9.5	49	-
**6**	50	50	55	-	6.1	58	17:33
**7**	50	75	100	-	6.6	57	6:19
**8**	50	50	100	500	8.9	60	11:14
**9**	110	50	100	500	8.3	54	91:9
**10**	100	50	100	500	8.9	55	22:3

Table S3: Table showing reactant concentrations, final pH and yield (**20**$_G$ and **29**$_G$ combined) after 24 hours at room temperature and the ratio formed of **20**$_G$ and **29**$_G$ at initial pH 9.5. Yields based on the amount of $RSCH_2CH_2NHCH_2CN$ (combined **20**$_G$ and **29**$_G$) observed after 24 hours.

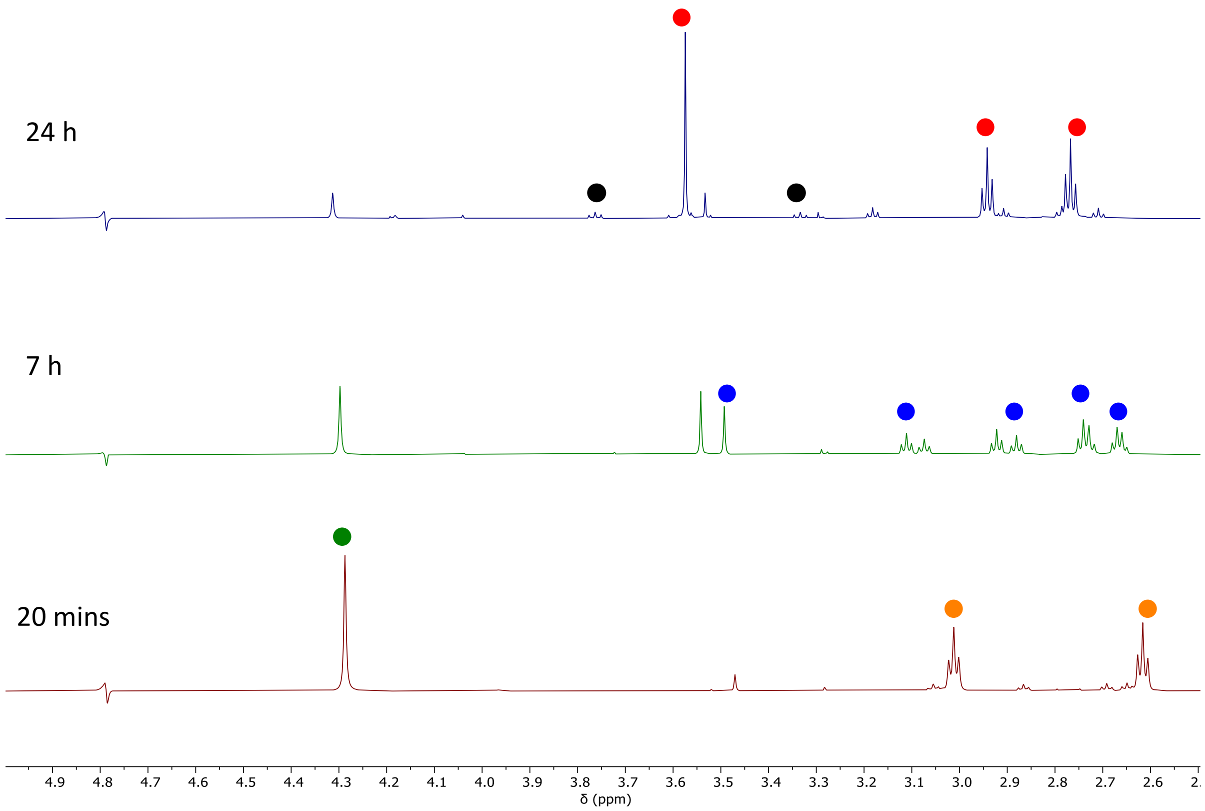

Figure S46: ^{1}H NMR (600 MHz, H_2O/D_2O 9:1, noesygppr1d, 2.5-5.0 ppm) spectra to show the reaction of cystamine (**6**, 50 mM), glycolonitrile (**21a**, 100 mM) and $K_3[Fe(CN)_6]$ (100 mM) at pH 9.5 (borate buffer, 500 mM) and room temperature, which yields **20_G** and **29_G**.

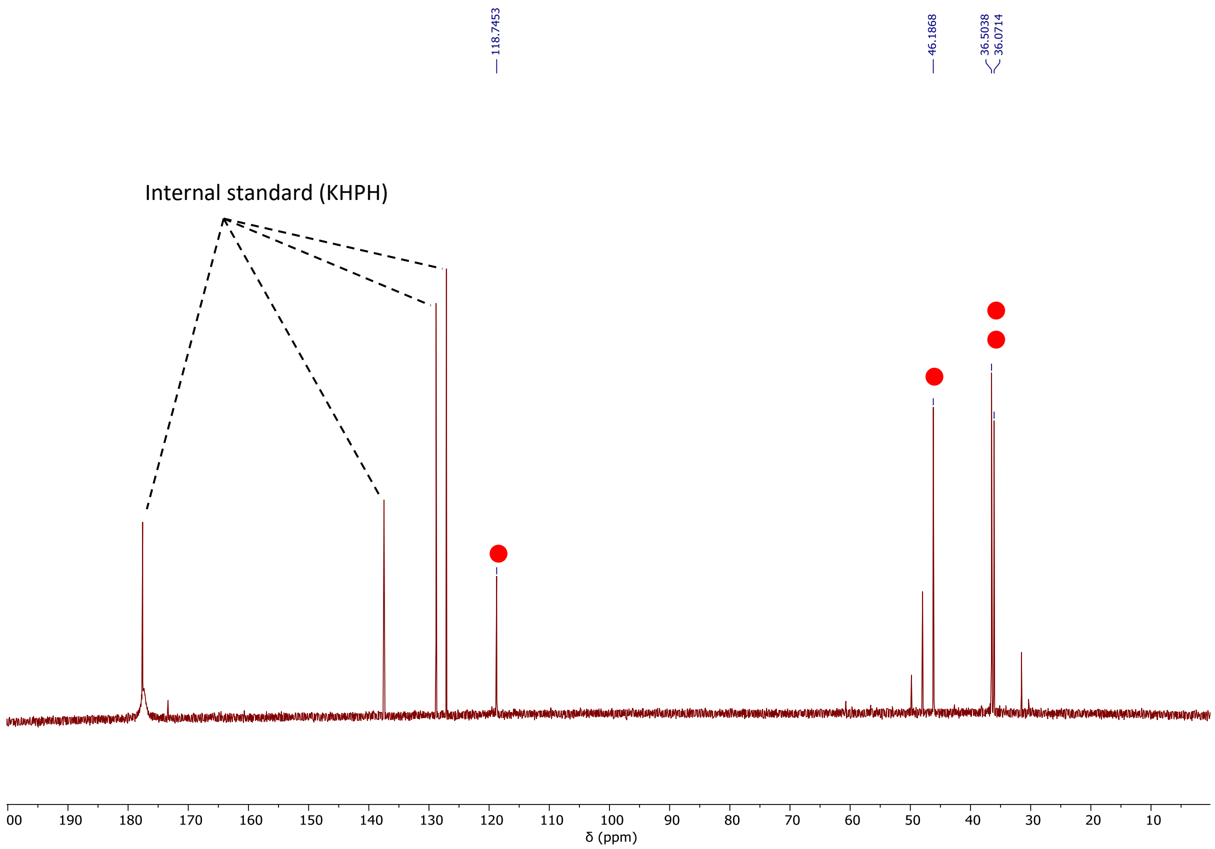

Figure S47: ^{13}C NMR (150 MHz, H_2O/D_2O 9:1, noesygppr1d, 0.0-200 ppm) spectrum to show the reaction of cystamine (**6**, 50 mM), glycolonitrile (**21a**, 110 mM) and $K_3[Fe(CN)_6]$ (100 mM) with KHPH (25 mM; internal standard) at pH 9.5 (borate buffer, 500 mM) and room temperature, which yields **20_G**.

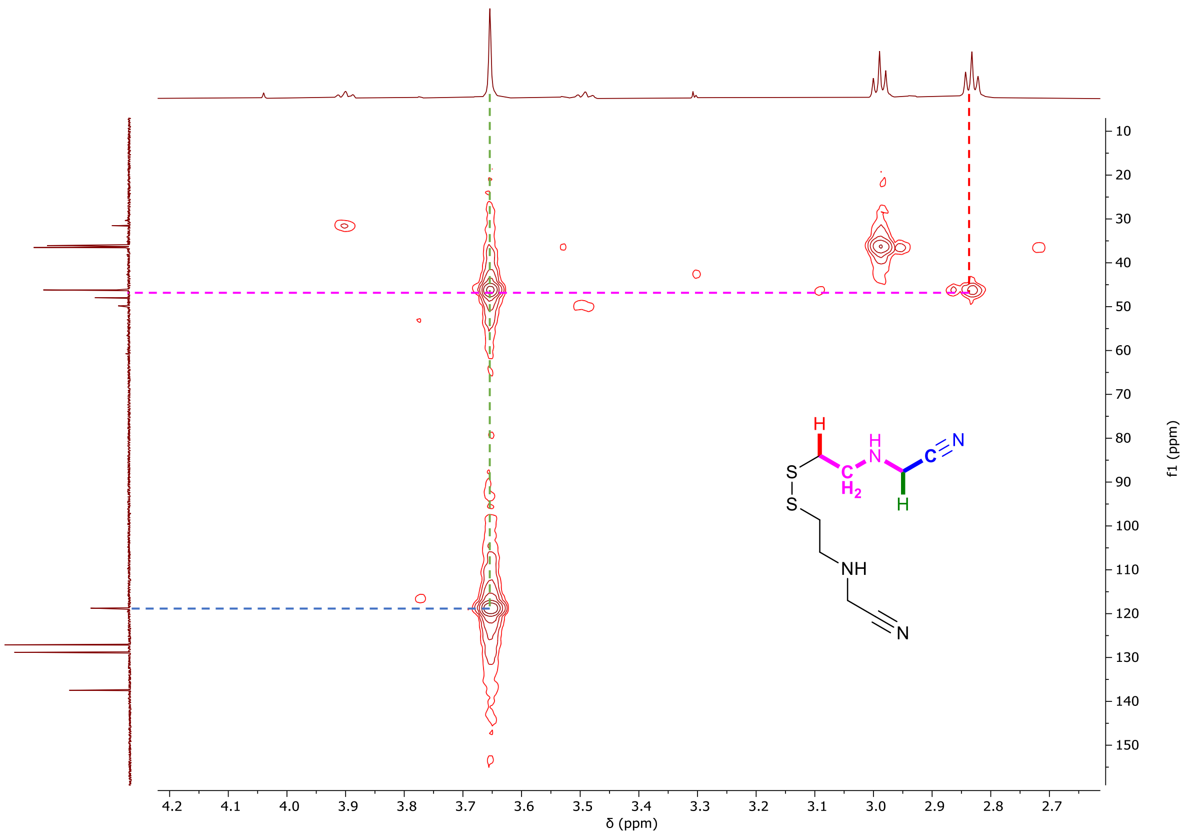

Figure S48: $^{1}H–^{13}C$ HMBC (^{1}H: 600 MHz [2.6-4.2 ppm], ^{13}C: 150 MHz [10-150ppm]) spectrum to show the diagnostic $^{3}J_{CH}$ (CH_2-CN) and $^{2}J_{CH}$ (S-CH_2) coupling to N-CH_2, and $^{2}J_{CH}$ (CH_2-CN) to CN at pH 9.5, that is characteristic of **20_G**.

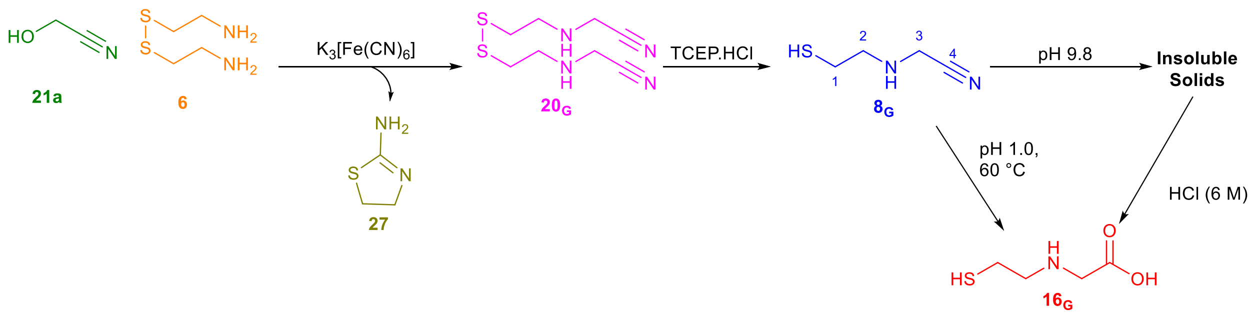

Reaction A: A solution of cystamine **6** (50 mM), glycolonitrile **21a** (100 mM), $K_3(Fe(CN)_6]$ (5 mM) and KHPH (12.5 mM; internal standard) in borate buffer solution (BBS, 5 mL, 200 mM, pH 9.5) was incubated at room temperature. Periodically an aliquot of the reaction mixture (500 µL) was diluted with D_2O (60 µL) and NMR spectra were acquired. After 24 hours the formation of β, β'-disulfide-α, α'-dinitrile **20**$_G$ (66%) was observed. Products from reaction A were used for reactions B and C.

Reaction B: TCEP (500 mM, 75 µL) was added to an aliquot (500 µL) of Reaction A, and the solution was adjusted to pH 9.8 with NaOH (3M). A suspension was observed to form within 1 hour. HCl (750 µL, 12 M) was added to the suspension and the solution was then incubated at 100 °C for 2.5 hours. The solution was cooled to room temperature, and TCEP·HCl (~5 mg) and D_2O (50 µL) were added to an aliquot (200 µL) of the solution. The solution was then adjusted to pH 4.0 with NaOH (6 N), and NMR spectra were acquired. The formation of **16**$_G$ (>95% conversion from **20**$_G$) was observed. The identity of the product (**16**$_G$) was confirmed by spiking with authentic synthetic standard.

Reaction C: TCEP (500 mM, 75 µL) and D_2O (60 µL) was added to an aliquot (500 µL) of Reaction A, and the solution was rapidly adjusted to pH 1.0 with HCl (6M). NMR spectra were periodically acquired. After 24 hours near quantitative conversion (>95%) of **20**$_G$ to **8**$_G$ was observed, but no solids were observed. Additional TCEP·HCl (500 mM, 75 µL) was added, and the solution was then incubated at 60 °C. NMR spectra were periodically acquired, and near quantitative conversion to **16**$_G$ (>95%) was observed after 72 hours. The identity of **16**$_G$ was confirmed by spiking with authentic synthetic standard.

Compound 8$_G$·HCl: ^{1}H NMR (700 MHz, H_2O/D_2O 9:1, pH 1.0): δ_H 2.88 (obs t, 2H, (C1)-H), 3.40 (t, *J* = 6.6 Hz, 2H, (C2)-H), 4.32 (s, 2H, (C3)-H). **^{13}C NMR (175 MHz, H_2O/D_2O 9:1, pH 1.0):** δ_C 20.5 (C1), 35.0 (C3), 50.9 (C2), 113.4 (C4). **HRMS (ESI$^+$):** calcd. for $[C_4H_8N_2S+H]^+$: 117.0481; Observed: 117.0480.

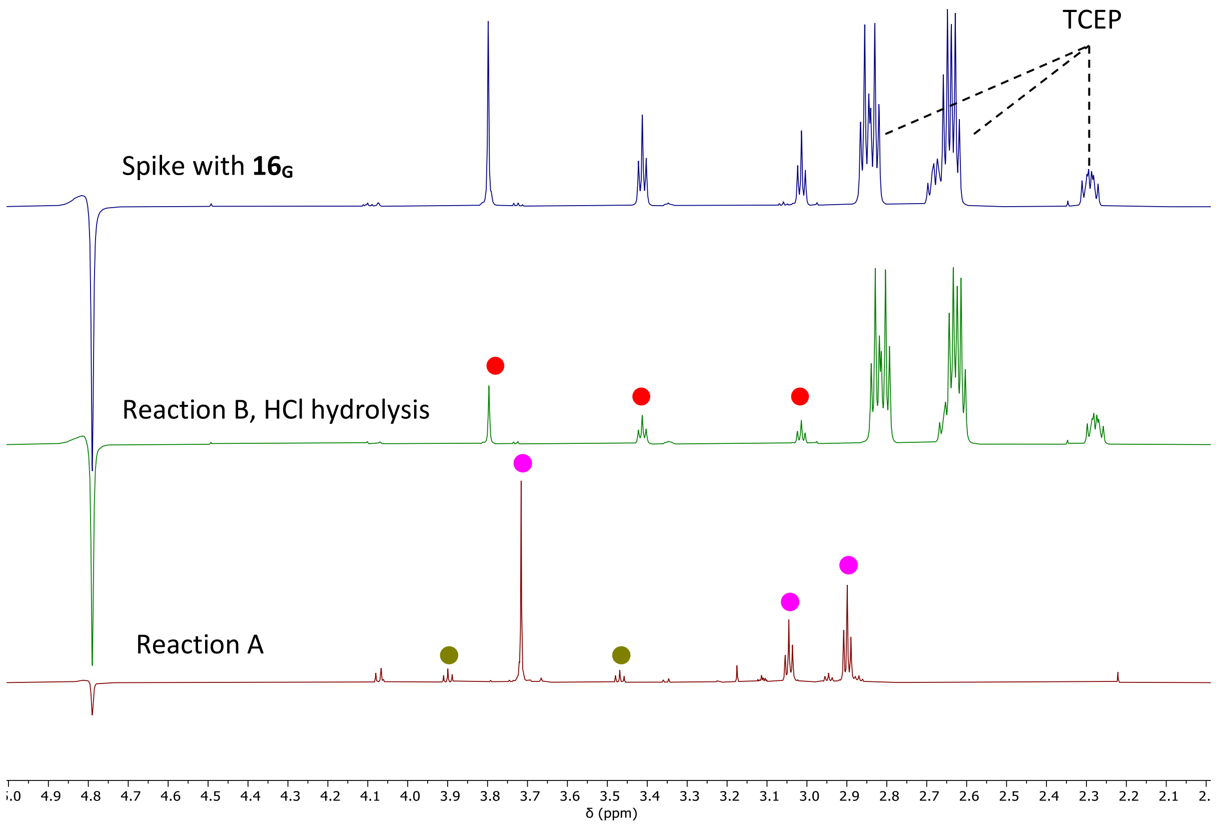

Figure S49: ^{1}H NMR (700 MHz, H_2O/D_2O 9:1, noesygppr1d, 2.0-5.0 ppm) spectra to show the formation of **16$_G$** (identified by spiking with authentic synthetic standard) from HCL (6M) hydrolysis of solids generated from **8$_G$** at pH 9.8, where **8$_G$** was afforded by TCEP reduction of **20$_G$** (Reaction B). **20$_G$** was formed from the reaction of cystamine (**6**, 50 mM), glycolonitrile (**21a**, 100 mM) and $K_3[Fe(CN)_6]$ (5 mM) at pH 9.5 and room temperature (Reaction A).

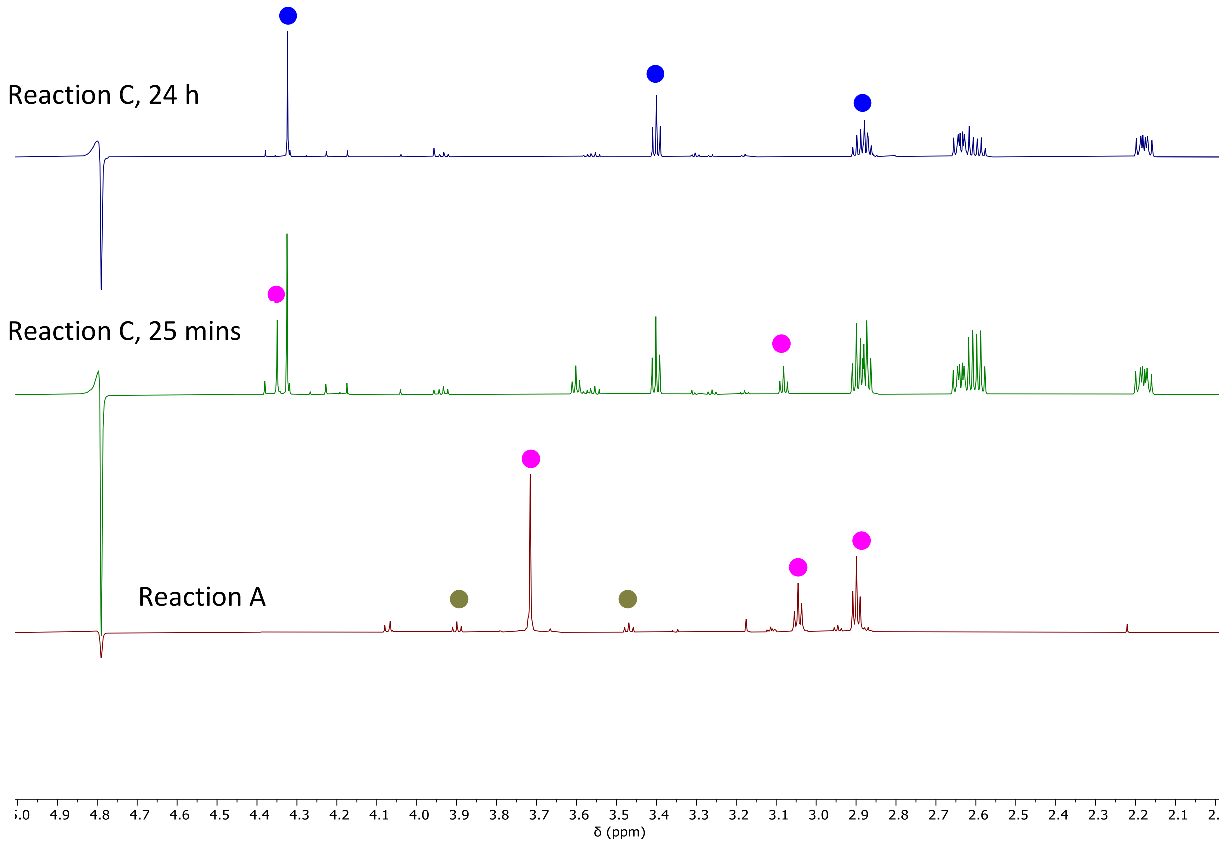

Figure S50: ^{1}H NMR (700 MHz, H_2O/D_2O 9:1, noesygppr1d, 2.0-5.0 ppm) spectra to show the formation of $\mathbf{8_G}$ from $\mathbf{20_G}$ via TCEP reduction at pH 1.0 and at room temperature (Reaction C).$\mathbf{20_G}$ was formed from the reaction of cystamine (**6**, 50 mM), glycolonitrile (**21a**, 100 mM) and $K_3[Fe(CN)_6]$ (5 mM) at pH 9.5 and room temperature (Reaction A).

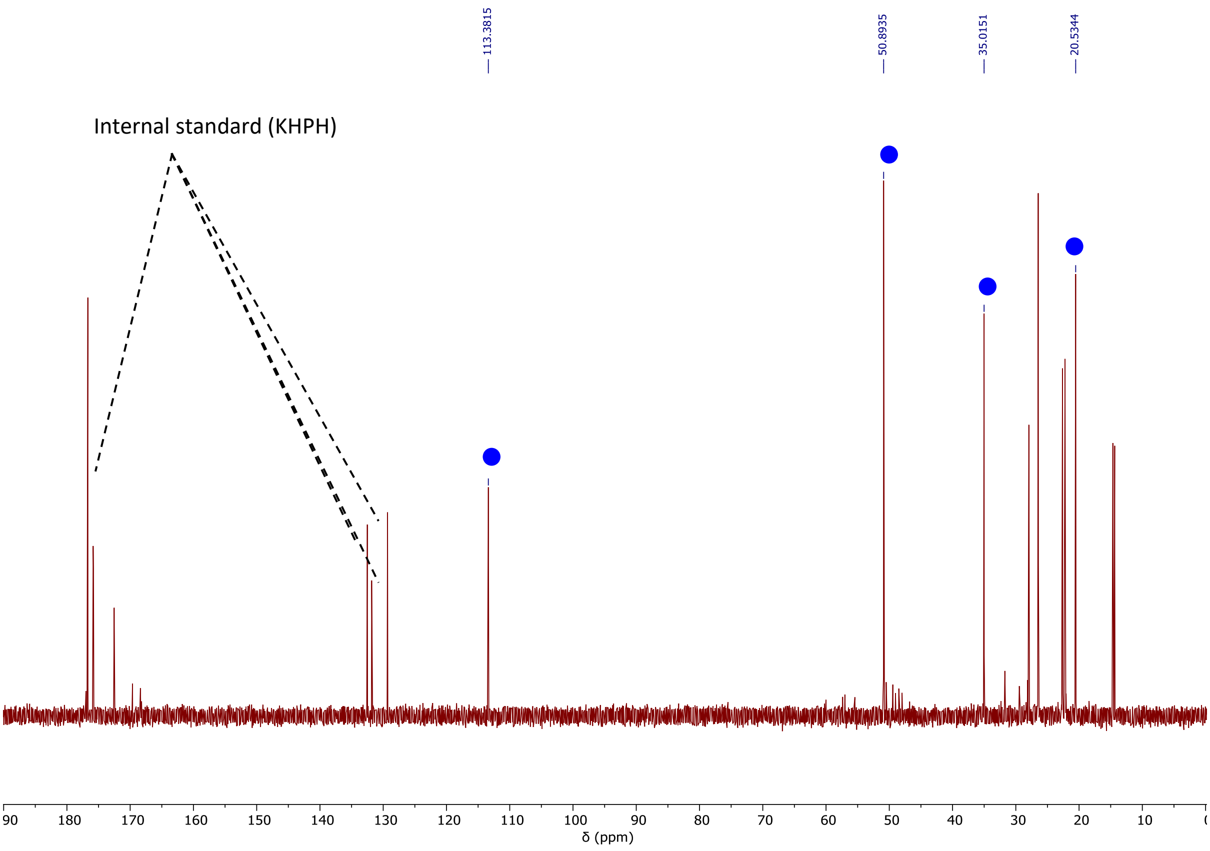

Figure S51: ^{13}C NMR (175 MHz, H_2O/D_2O 9:1, noesygppr1d, 0.0-200 ppm) spectrum to show the formation of $\mathbf{8_G}$ from $\mathbf{20_G}$ via TCEP reduction at pH 1.0 and at room temperature (Reaction C). $\mathbf{20_G}$ was generated from the reaction of cystamine (**6**, 50 mM), glycolonitrile (**21a**, 100 mM) and $K_3[Fe(CN)_6]$ (5 mM) at pH 9.5 and room temperature with KHPH (12.5 mM, internal standard) (Reaction A).

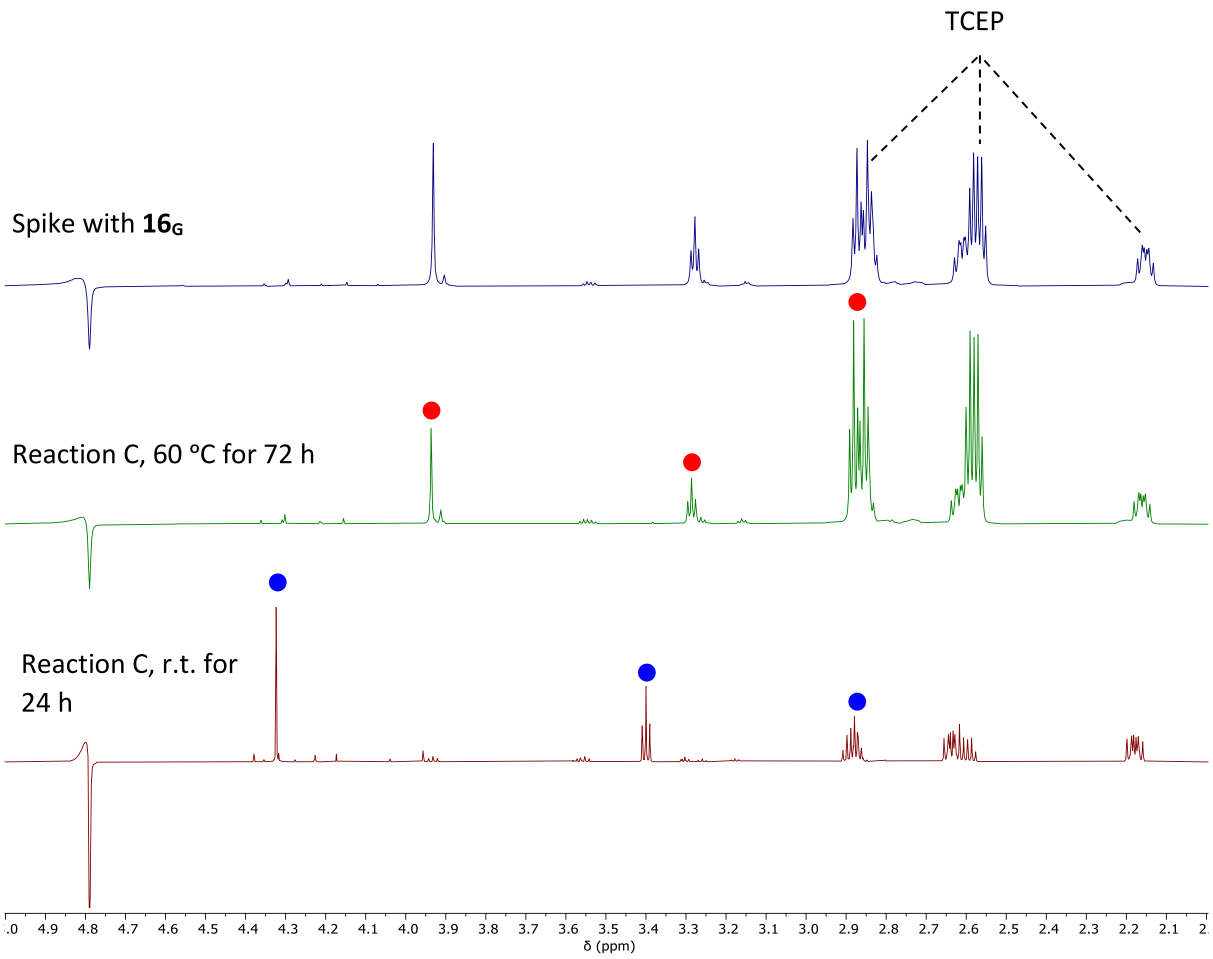

Figure S52: ^{1}H NMR (700 MHz, H_2O/D_2O 9:1, noesygppr1d, 2.0-5.0 ppm) spectra to show the formation of **16_G** (identified by spiking with authentic synthetic standard) from **8_G** (**8_G** obtained from **20_G** via TCEP reduction) at 60 °C and pH 1.0 (Reaction C). Further TCEP added to the reaction after heating for 72 hours at pH 1.0.

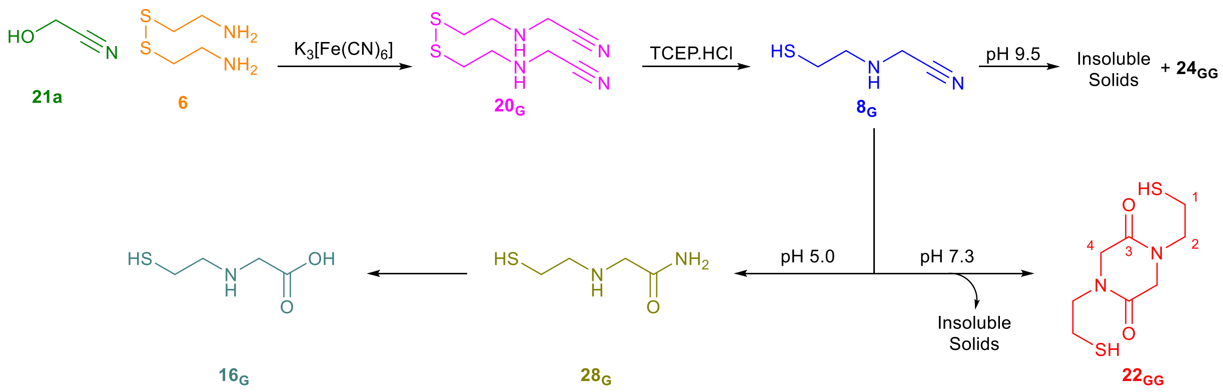

A solution of cystamine **6** (50 mM), glycolonitrile **21a** (100 mM), $K_3[(Fe(CN)_6]$ (50 mM) and MSM (25 mM; internal standard) in H_2O (10 mL) was adjusted to pH 9.5 with NaOH/HCl (1-4 M) and incubated at room temperature. The solution was periodically readjusted to pH 9.5 (after 1–8-hour intervals) with NaOH/HCl (1 M). Periodically an aliquot (500 µL) of the reaction was diluted with D_2O (60 µL), and NMR spectra were acquired. After 18.5 hours, the formation of **20**$_G$ (50%) was observed. The solution was then adjusted to pH 5.0 with HCl (1 M). An aliquot (500 µL) of this solution was diluted with H_2O/D_2O (8:2, 500 µL) containing TCEP (140 mM) and adjusted to pH 5.0-9.5 with NaOH/HCl (1-4 M). NMR spectra were acquired and the formation of **8**$_G$ (>95%) was observed after 5 minutes. The solution was then incubated at room temperature and regular NMR spectra were acquired. At pH 5.0, **8**$_G$ was observed to hydrolyse to furnish amide **28**$_G$ and then acid **16**$_G$ (80% after 7 days), no insoluble solids were observed. The identity of **16**$_G$ was confirmed by spiking with authentic synthetic standard (Supplementary Figure 53). At pH 7.3, **8**$_G$ was observed to yield **22**$_{GG}$ (50%) after 3 days (Supplementary Figure 54-55). At pH 9.5, **8**$_G$ was observed to yield **22**$_{GG}$ (25%) in 7 days (Supplementary Figure 56). Solid precipitate was also observed at pH 7.3-9.5.

Compound 22$_{GG}$**: ^{1}H NMR (600 MHz, H_2O/D_2O 9:1):** δ_H 2.73 (t, *J* = 7.0 Hz, 2H, (C1)-H), 3.55 (t, *J* = 7.0 Hz, 2H, (C2)-H), 4.15 (s, 2H, (C4)-H). **^{13}C NMR (150 MHz, H_2O/D_2O 9:1):** δ_C 21.3 (C1), 49.4 (C2), 50.6 (C4), 166.8 (C3). **HRMS (ESI$^+$):** calcd. for $[C_8H_{14}N_2O_2S_2+H]^+$: 235.0570; Observed: 235.0571.

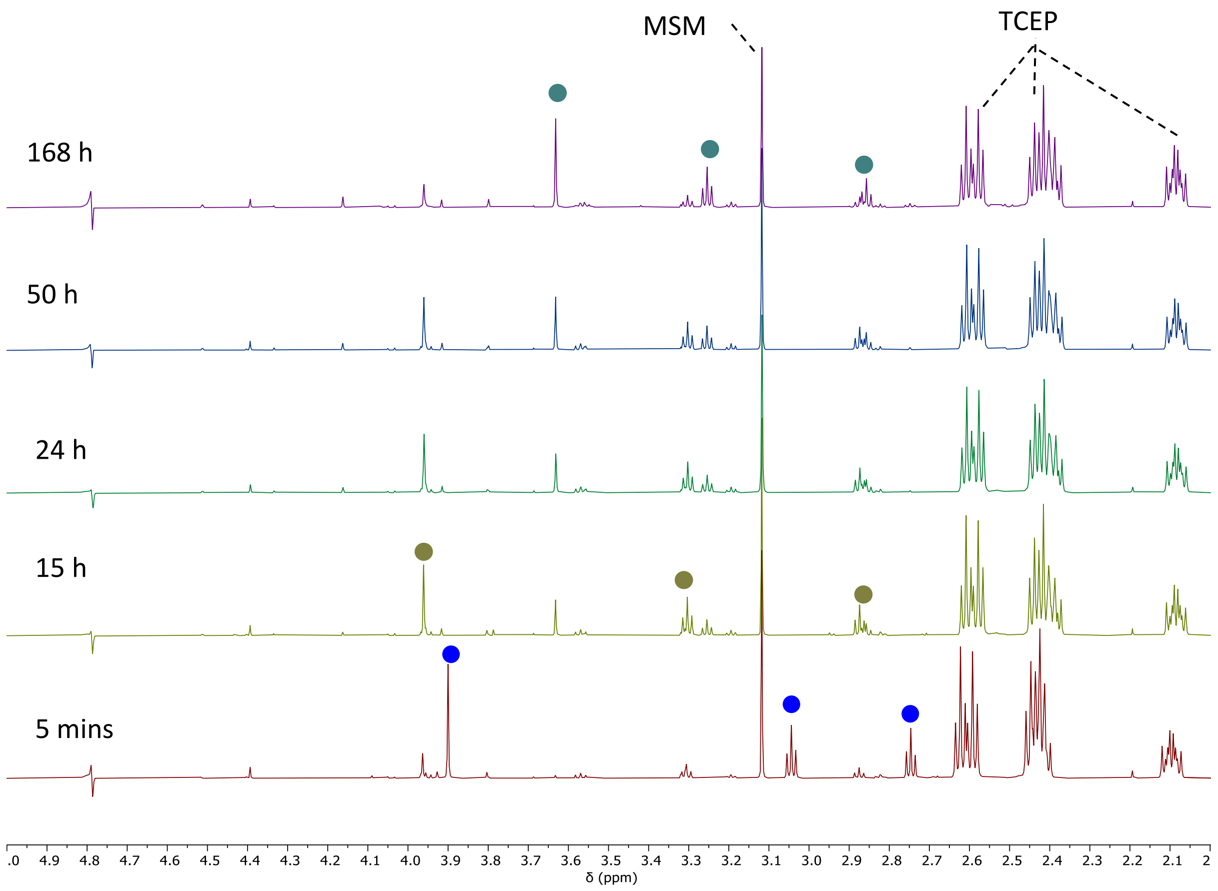

Figure S53: ^{1}H NMR (600 MHz, H_2O/D_2O 9:1, noesygppr1d, 2.0-5.0 ppm) spectra to show the formation of $\mathbf{16_G}$ from $\mathbf{8_G}$ (25 mM) via $\mathbf{28_G}$ ($\mathbf{8_G}$ obtained through the in situ TCEP reduction of $\mathbf{20_G}$) and MSM (12.5 mM, internal standard) at pH 5.0.

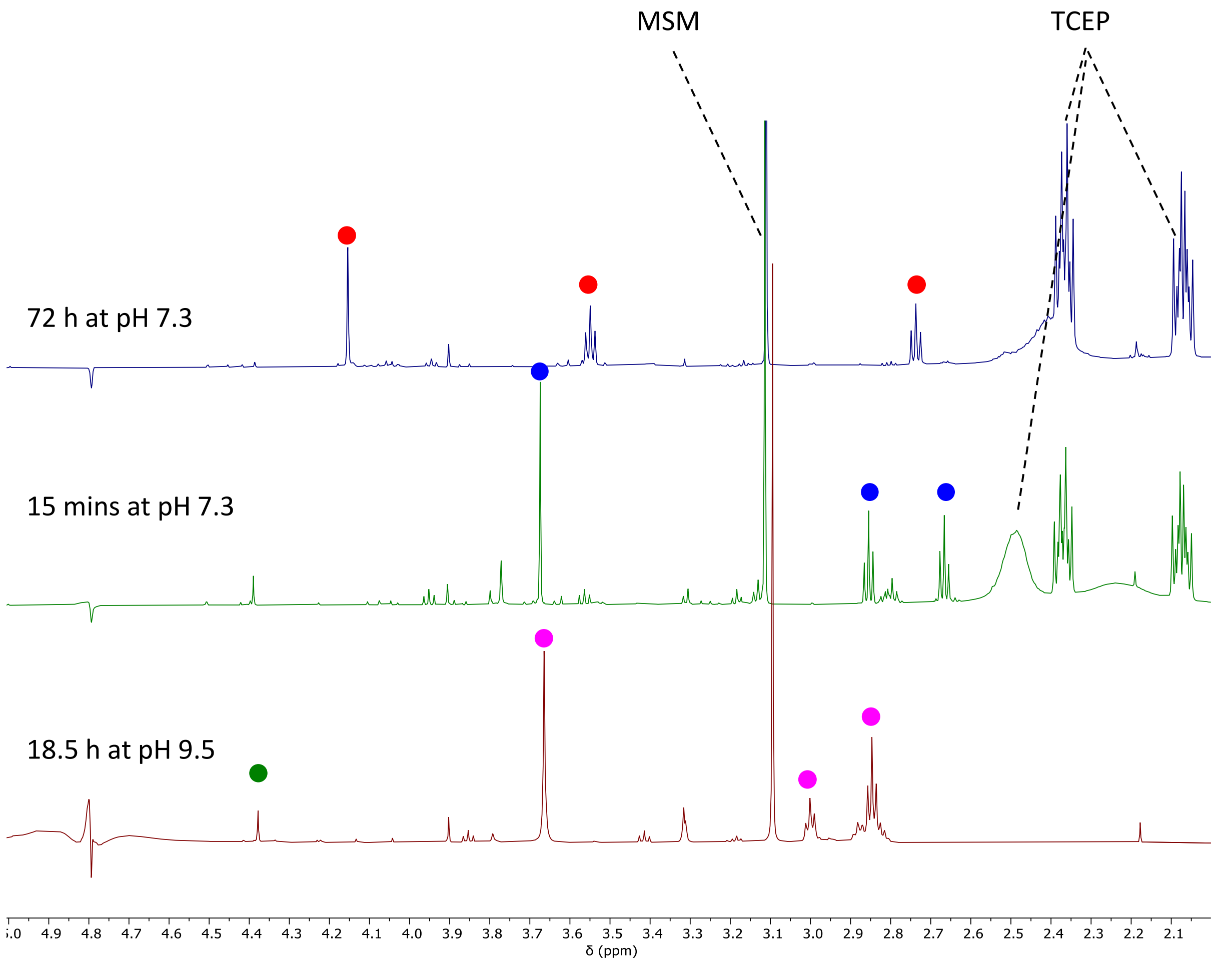

Figure S54: ^{1}H NMR (600 MHz, H_2O/D_2O 9:1, noesygppr1d, 2.0-5.0 ppm) spectra to show the formation of $\mathbf{8_G}$ from TCEP reduction of $\mathbf{20_G}$ ($\mathbf{20_G}$ was obtained from the reaction of cystamine (**6**, 50 mM), glycolonitrile (**21a**, 100 mM) and $K_3[Fe(CN)_6]$ (50 mM) at pH 9.5 with MSM (25 mM, internal standard)) that then dimerises to yield $\mathbf{22_{GG}}$ at pH 7.3.

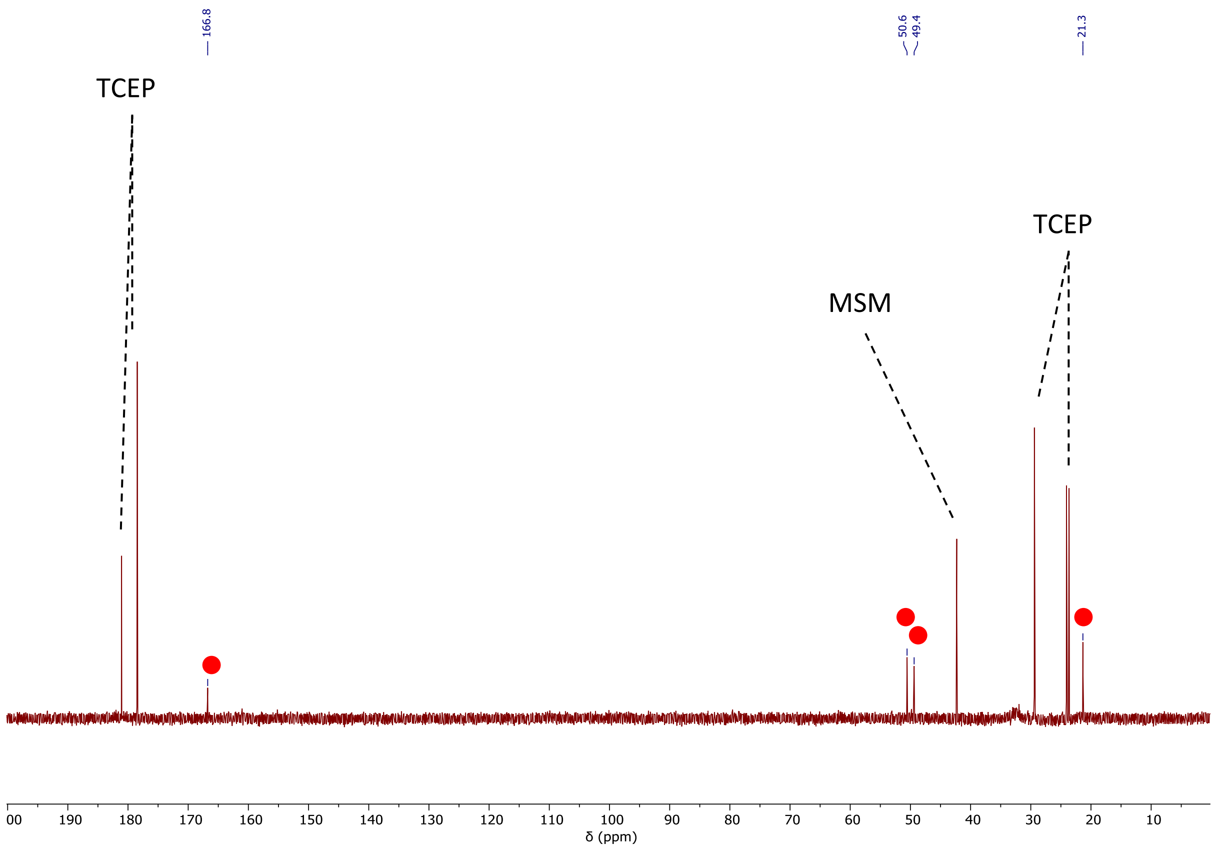

Figure S55: ^{13}C NMR (150 MHz, H_2O/D_2O 9:1, noesygppr1d, 0.0-200 ppm) spectrum to show the formation of $\mathbf{22_{GG}}$ from $\mathbf{8_G}$ (25 mM) ($\mathbf{8_G}$ obtained from TCEP reduction of $\mathbf{20_G}$) with MSM (12.5 mM, internal standard).

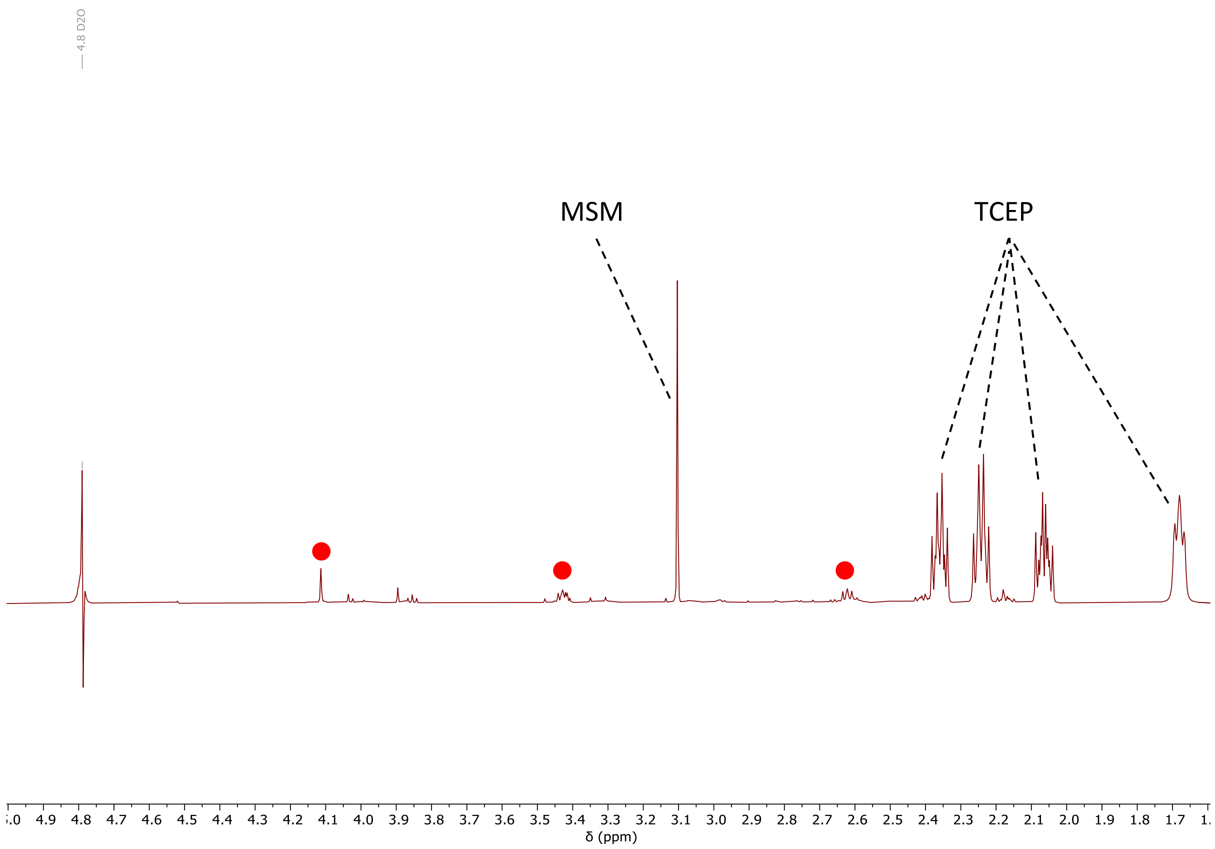

Figure S56: ^{1}H NMR (600 MHz, H_2O/D_2O 9:1, noesygppr1d, 1.6-5.0 ppm) spectrum to show the formation of **22_{GG}** from **8_G** (25 mM) (**8_G** obtained through TCEP reduction of **20_G**) with MSM (12.5 mM, internal standard) at pH 9.5.

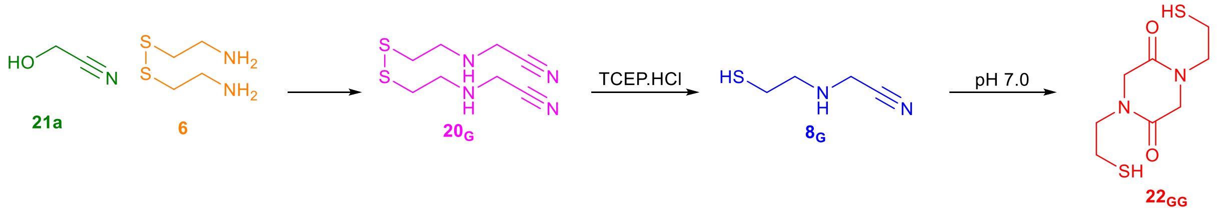

A solution of cystamine **6** (100 mM), glycolonitrile **21a** (200 mM), $K_3(Fe(CN)_6]$ (100 mM) and MSM (50 mM; internal standard) in H_2O (5 mL) was incubated at pH 9.5 and room temperature. The solution was periodically adjusted to pH 9.5 (after 1–8-hour intervals) using HCl/NaOH (1-4 M) and NMR spectra were regularly acquired by combining an aliquot (500 µL) of the solution with D_2O (60 µL). After 28 hours the formation of **20$_G$** (50%) was observed, and the solution was adjusted to pH 7.0 with HCl/NaOH (1–4 M). An aliquot (100–500 µL) of the solution was added to TCEP (10–100 mmol) and D_2O (100 µL), adjusted to pH 7.0 with HCl/NaOH (1–4 M) and volumetrically diluted to 1 mL with H_2O. The resultant solution was then incubated at room temperature and NMR spectra were periodically acquired. In all reactions **8$_G$** (>95%) was observed after 5 mins, and then the formation of cyclic dipeptoid **22$_{GG}$** (65%) was observed after 45 hours.

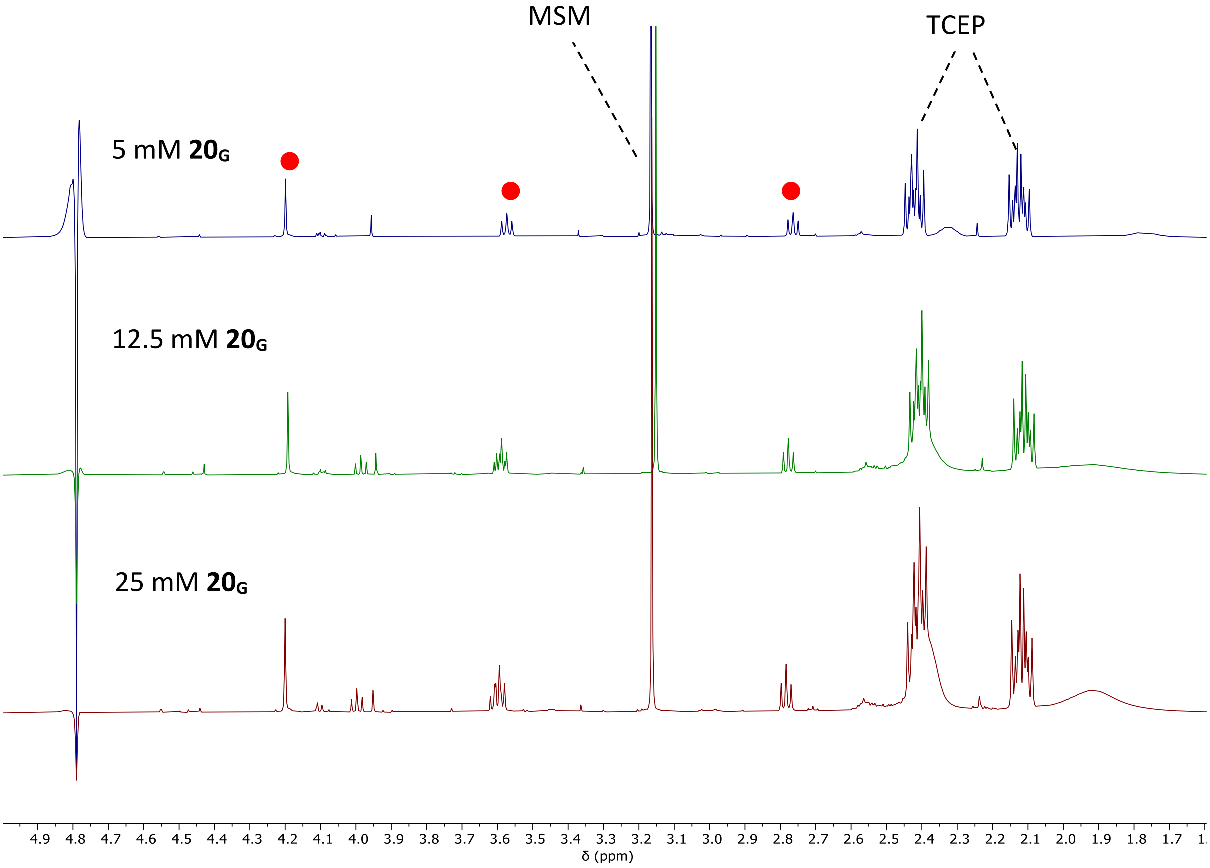

Figure S57: ^{1}H NMR (500 MHz, H_2O/D_2O 9:1, noesygppr1d, 1.6-5.0 ppm) spectra to show the formation of **22$_{GG}$** from **8$_G$** (10 – 50 mM) at pH 7.0 and room temperature (**8$_G$** obtained from **22$_G$** via TCEP reduction).

Synthesis of **8**

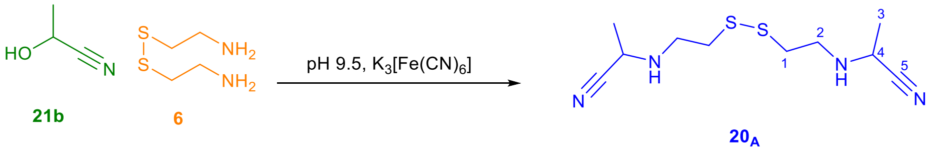

A solution of cystamine **6** (50 mM), lactonitrile **21b** (100 mL), $K_3[Fe(CN)_6]$ (100 mM) and KHPH (25 mM; internal standard) in borate buffer (2 mL, 500 mM, H_2O/D_2O 9:1) was incubated at pH 9.5 and room temperature. After 10 mins NMR spectra were acquired and 71% conversion to **20_A** was observed.

Compound 20_A: ^{1}H NMR (600 MHz, H_2O/D_2O 9:1): δ_H 1.37 (d, *J* = 7.0 Hz, 3H, (C3)-H), 2.75 (m, 2H, (C1)-H), 2.87 (dt, *J* = 12.3, 6.3 Hz, 1H, (C2)-H), 3.05 (m, 1H, (C2)-H), 3.73 (q, *J* = 7.0 Hz, 1H, (C4)-H). **^{13}C NMR (150 MHz, H_2O/D_2O 9:1):** δ_C 18.2 (C3), 36.62 & 36.65 (C1), 44.7 (C4), 45.19 & 45.20 (C2), 121.4 (C5). **HRMS (ESI$^+$):** calcd. for $[C_{10}H_{18}N_4S_2+H]^+$: 259.1046; Observed: 259.1042.

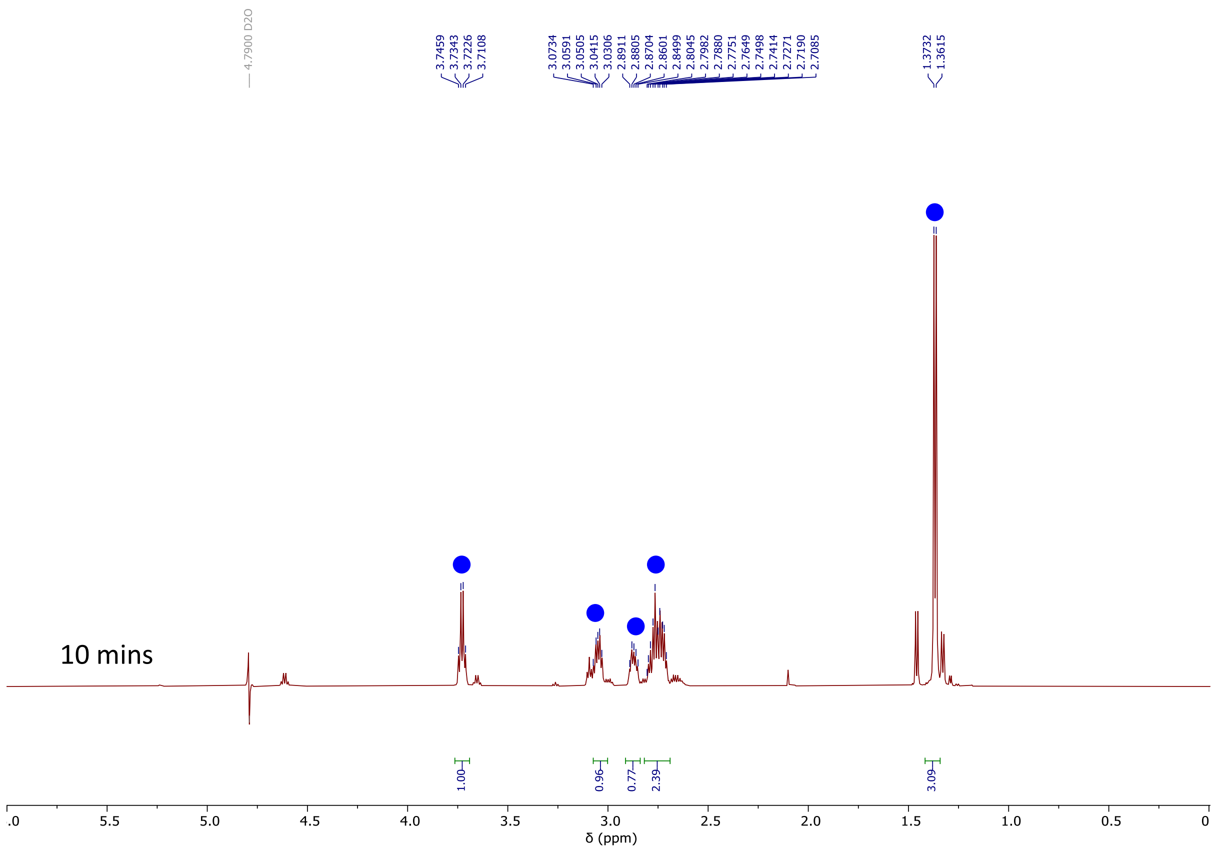

Figure S58: ^{1}H NMR (600 MHz, H_2O/D_2O 9:1, noesygppr1d, 0.0-6.0 ppm) spectrum to show the reaction of cystamine (**6**, 50 mM), lactonitrile (**21b**, 100 mM) and $K_3[Fe(CN)_6]$ (100 mM) at pH 9.5 (borate buffer, 500 mM) and room temperature, which yields **20_A**.

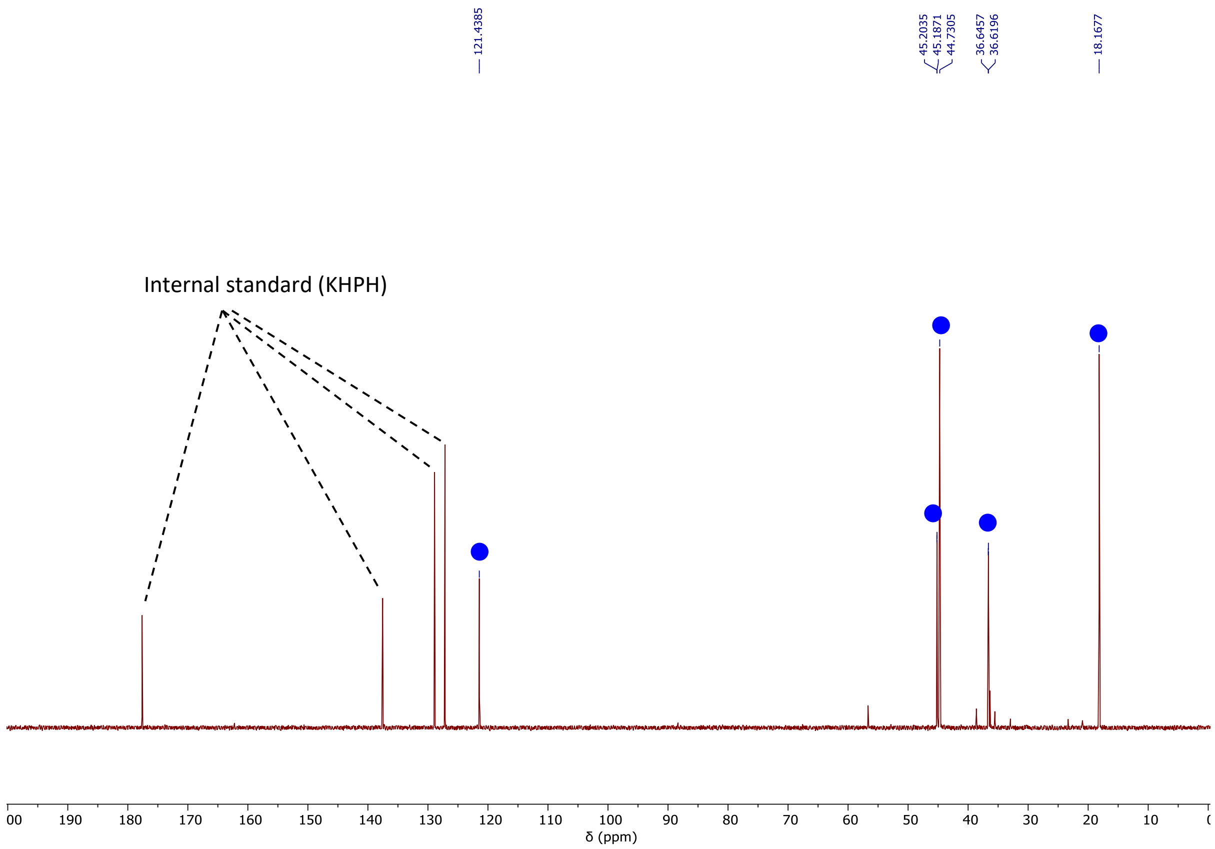

Figure S59: ^{13}C NMR (150 MHz, H_2O/D_2O 9:1, noesygppr1d, 0.0-200 ppm) spectrum to show the reaction of cystamine (**6**, 50 mM), lactonitrile (**21_A**, 100 mM) and $K_3[Fe(CN)_6]$ (100 mM) with KHPH (25 mM; internal standard) at pH 9.5 (borate buffer, 500 mM) and room temperature, which yields **20_A**.

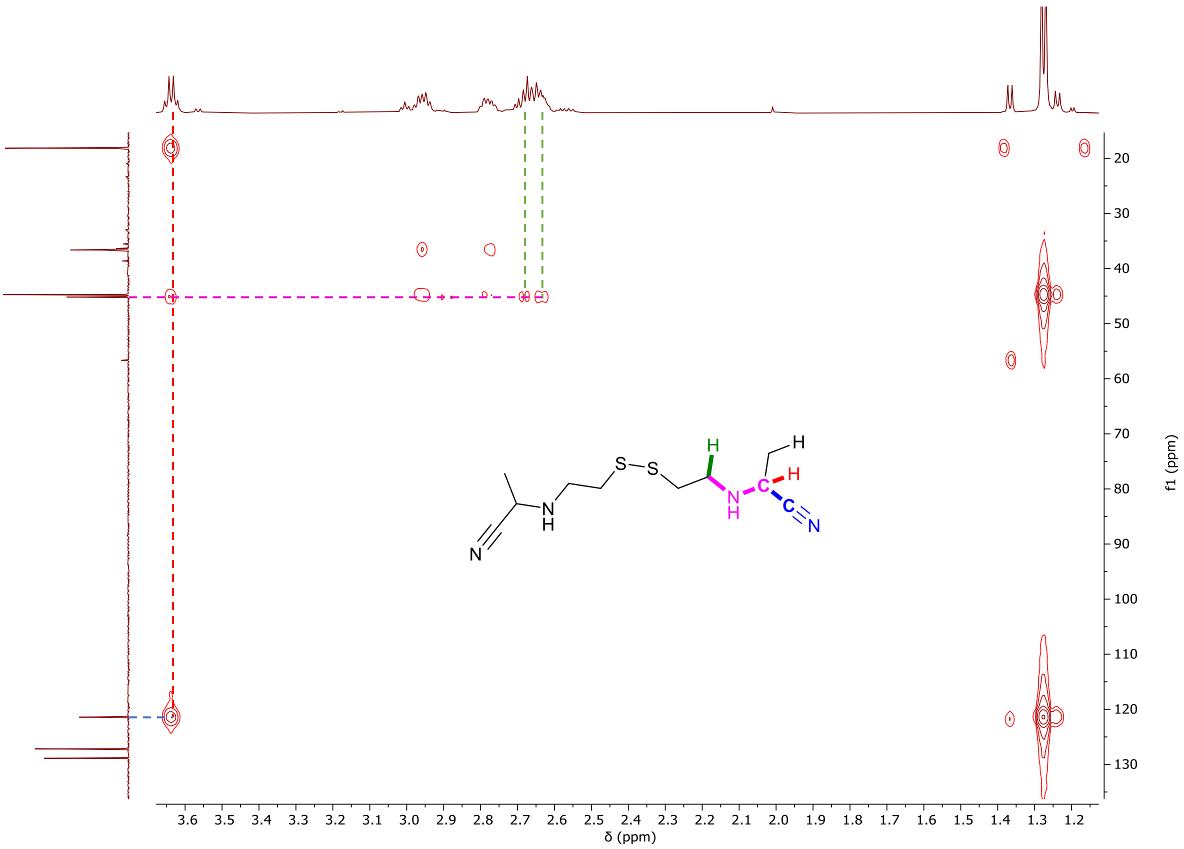

Figure S60: ^{1}H– ^{13}C HMBC (^{1}H: 600 MHz [1.2-3.7 ppm], ^{13}C: 150 MHz [10-140ppm]) spectrum to show the diagnostic $^3J_{CH}$ of *N*-CH_2 to α-carbon and $^2J_{CH}$ coupling of α-CH to the nitrile moiety at pH 9.5, that is characteristic of **20$_A$**.

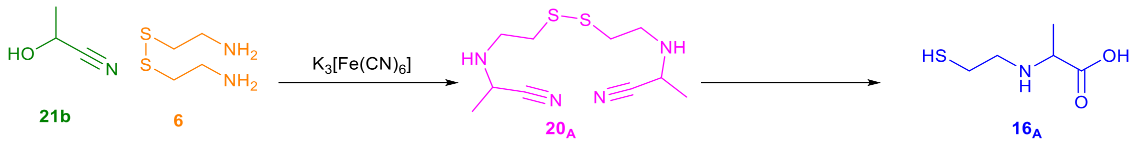

A solution of cystamine **6** (50 mM), lactonitrile **21b** (100 mM), $K_3[Fe(CN)_6]$ (25 mM) and KHPH (25 mM; internal standard) in borate buffer (1 mL, 250 mM) at pH 9.5 were incubated for 15 mins. HCl (500 µL, 12 M) was added to an aliquot (500 µL) of this solution and the resultant solution was incubated at 100 °C for 2 hours before cooling to room temperature. To an aliquot (100 µL) of this solution, D_2O (100 µL), H_2O (500 µL) and a crystal of TCEP·HCl was added. The resultant solution was adjusted to pH 7.0 and NMR spectra were acquired. The formation of $\mathbf{16_A}$ (65%) was observed and the identity of $\mathbf{16_A}$ was confirmed by spiking with authentic synthetic standard.

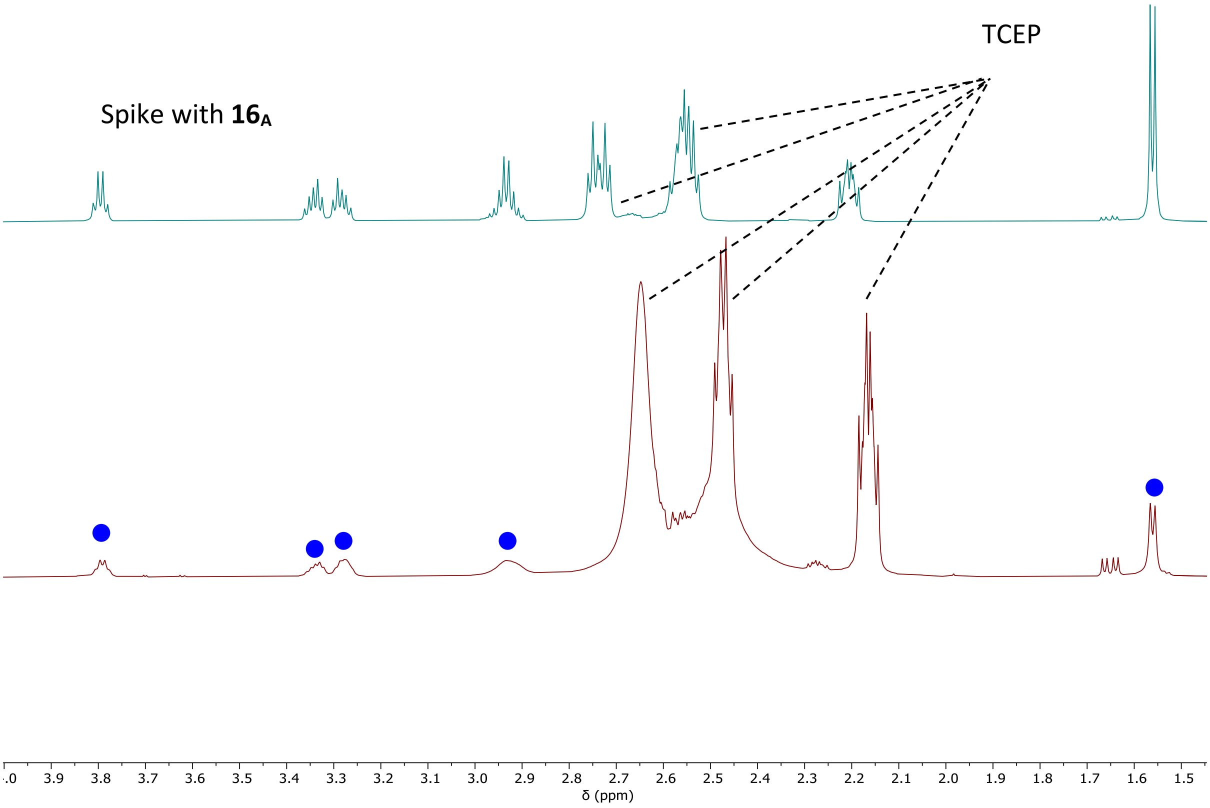

Figure S61: ^1H NMR (600 MHz, H_2O/D_2O 9:1, noesygppr1d, 0.0-6.0 ppm) spectra to show the reaction of cystamine (**6**, 50 mM), lactonitrile (**21b**, 100 mM) and $K_3[Fe(CN)_6]$ (25 mM) at pH 9.5 (borate buffer, 500 mM) at room temperature, which yields $\mathbf{20_A}$. The nitrile moiety was hydrolysed in HCl (6 M) at 100 °C and the disulfide reduced with TCEP to yield $\mathbf{16_A}$, which was identified by spiking with authentic synthetic standard.

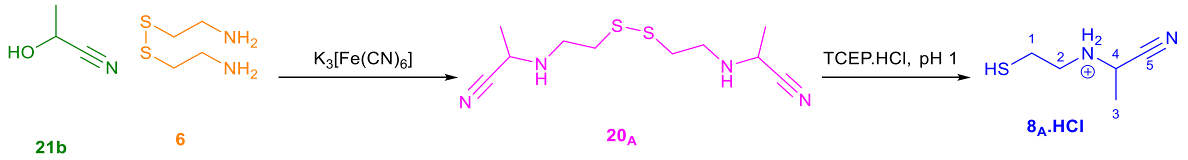

A solution of cystamine **6** (50 mM), lactonitrile **21b** (100 mM), $K_3[Fe(CN)_6]$ (5 mM) and KHPH (12.5 mM; internal standard) in borate buffer (1.5 mL, 200 mM, H_2O/D_2O 9:1) at pH 9.5 were incubated for 1 hour at room temperature. An aliquot (1 mL) of this solution was added to TCEP·HCl (21.5 mg, 0.075 mmol) and the resultant solution was adjusted to pH 1.0. NMR spectra of this aliquot indicated $\mathbf{8_A}$ (85%) had formed.

Compound $\mathbf{8_A}$·HCl: ^{1}H NMR (700 MHz, H_2O/D_2O 9:1): δ_H 1.73 (d, J = 7.1 Hz, 3H, (C3)-H), 2.89 (obs m, 2H, (C1)-H), 3.43 (ddt, J = 25.8, 12.8 & 6.3 Hz, 2H, (C2)-H), 4.66 (q, J = 7.0 Hz, 1H, (C4)-H). **^{13}C NMR (175 MHz, H_2O/D_2O 9:1):** δ_C 13.5 (C3), 18.1 (C1), 42.6 (C4), 47.1 (C2), 113.6 (C5). **HRMS (ESI$^+$):** calcd. for $[C_5H_{10}N_2S+H]^+$: 131.0638; Observed: 131.0635.

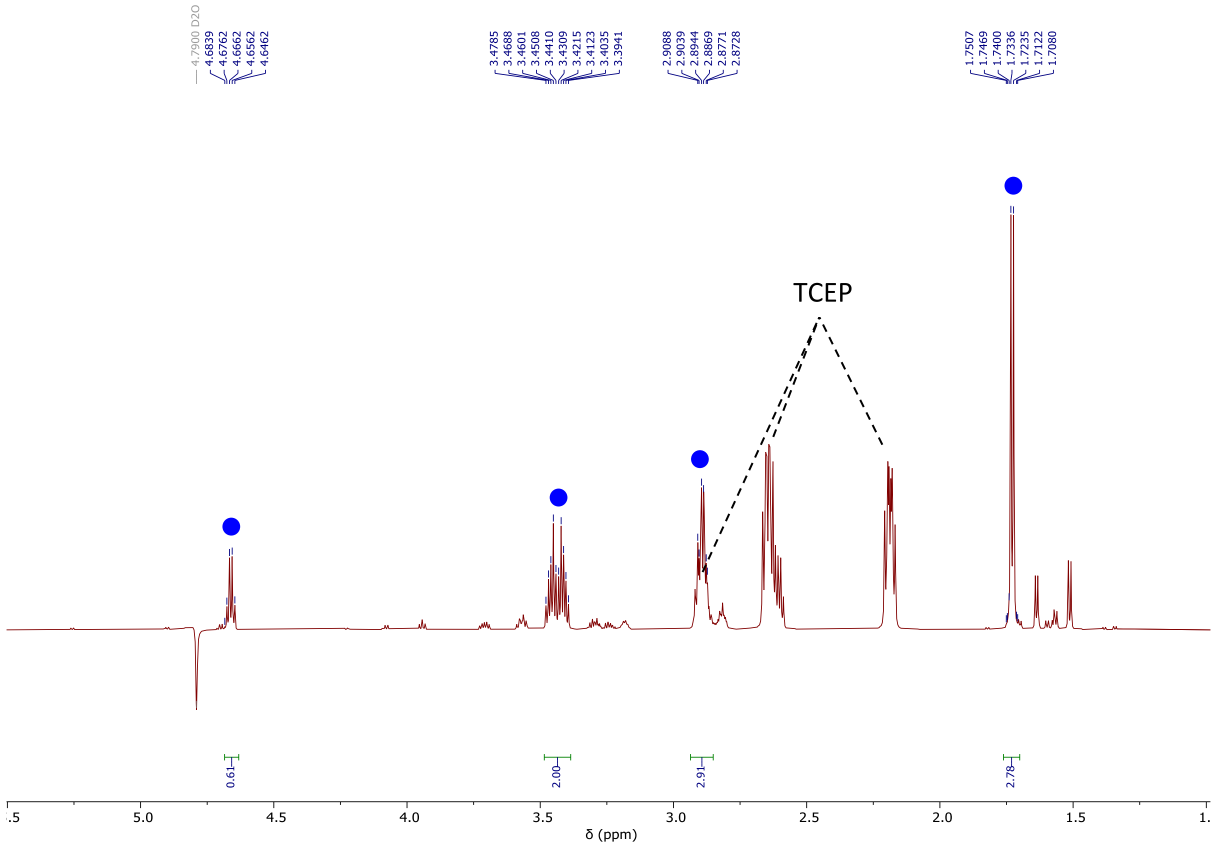

Figure S62: ^{1}H NMR (700 MHz, H_2O/D_2O 9:1, noesygppr1d, 1.0-5.5 ppm) spectrum to show the formation of $\mathbf{8_A}$ after TCEP reduction of $\mathbf{20_A}$ at pH 1.0 ($\mathbf{20_A}$ was synthesised at pH 9.5 from cystamine (**6**, 50 mM), lactonitrile (**21b**, 100 mM) and $K_3[Fe(CN)_6]$ (5 mM)).

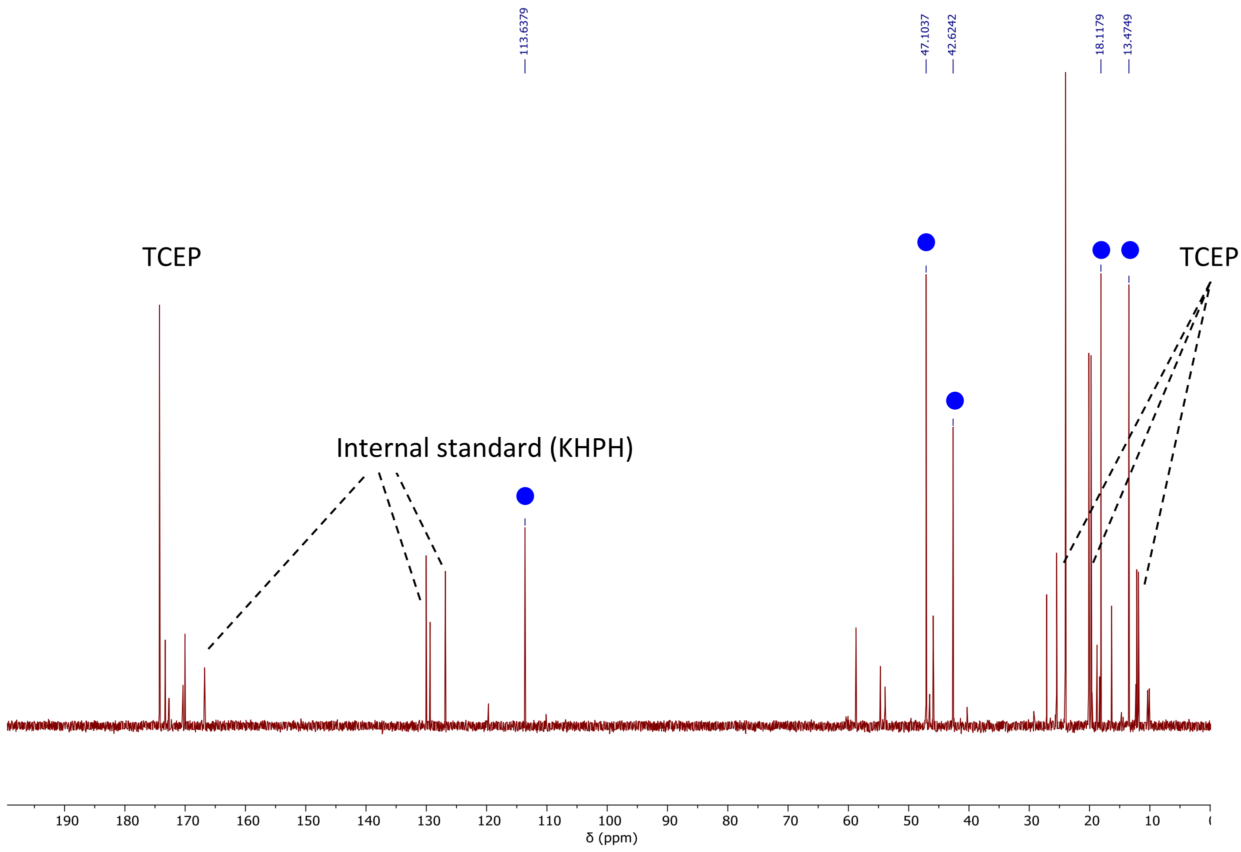

Figure S63: ^{13}C NMR (175 MHz, H_2O/D_2O 9:1, noesygppr1d, 0.0-200 ppm) spectrum to show the formation of $\mathbf{8_A}$ after TCEP reduction of $\mathbf{20_A}$ at pH 1.0 ($\mathbf{20_A}$ formed from cystamine (**6**, 50 mM), lactonitrile (**21b**, 100 mM) and $K_3[Fe(CN)_6]$ (5 mM) at pH 9.5 and room temperature with KHPH (12.5 mM; internal standard)).

Ligation of amidonitriles **2** with β or γ-*N*-thiolkyl amino acids **16**, **17** or **30**:

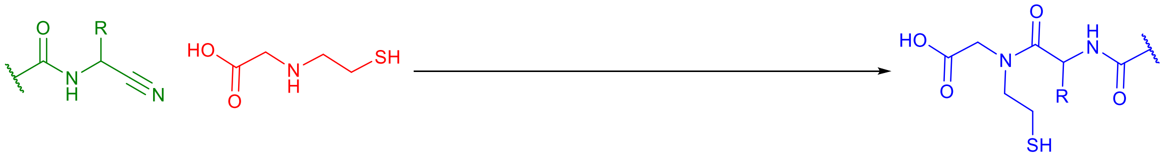

$2_{(AA)m}$ **16** **10**

General Procedure D: A solution of **16** (45–100 mM), **$2_{(AA)m}$** (37.5–100 mM), and MSM (12.5–100 mM; internal standard) or KHPH (25 mM; internal standard) in phosphate buffer (1–3 mL, pH 7, 500 mM) or borate buffer (1–3 mL, pH 9, 500 mM) was incubated at the specified temperature. The reaction was monitored by regular acquisition of NMR spectra and the yields of the ligation products are reported in Supplementary Table 4. Each reaction was monitored for 24 hours at pH 7 or until completion at pH 9. The product was observed to be as a mixture of 2 rotamers, except for **10f** which was observed to be a mixture of four rotamers. **10l** was observed to yield a mixture of two diastereomers, with each diastereomer composed of a mixture of two rotamers.

				pH 7		pH 9				
**Entry**	**2_{AA}**	**Thiol**	**Peptoid**	**Yield/%**	**t/h**	**Yield/%**	**t/h**	**[M+/-]**	**calc. m/z**	**Obs. m/z**
**1**	**2_G**	**16_G**	**10a**	80	24	>95	3	$[C_{16}H_{27}N_4O_8S_2]^+$	467.1265	467.1262
**2**	**2_G**	**17_G**	**24a**	75	24	>95	10	$[C_9H_{17}N_2O_4S]^+$	249.0904	249.0903
**3**	**2_G**	**30_G**	**31a**	82	24	91	24	$[C_{14}H_{18}N_2O_4S]^+$	310.0982	310.0982
**4**	**2_G**	**16_A**	**10b**	65	24	>95	5	$[C_9H_{17}N_2O_4S]^+$	249.0904	249.0903
**5**	**2_G**	**16_V**	**10c**	75	24	>95	5	$[C_{22}H_{39}N_4O_8S_2]^+$	551.2204	551.2187
**6**	**2_A**	**16_G**	**10d**	85	24	>95	10	$[C_9H_{17}N_2O_4S]^+$	249.0904	249.0900
**7**	**2_M**	**16_G**	**10e**	90	24	>95	10	$[C_{11}H_{21}N_2O_4S_2]^+$	309.0937	309.0934
**8**	**2_P**	**16_G**	**10f**	90	24	>95	15	$[C_{11}H_{19}N_2O_4S]^+$	275.1060	275.1056
**9**	**2_S**	**16_G**	**10g**	89	24	>95	10	$[C_9H_{17}N_2O_5S]^+$	265.0853	265.0844
**10**	**2_L**	**16_G**	**10h**	70	24	>95	24	$[C_{12}H_{23}N_2O_4S]^+$	291.1373	291.1371
**11**	**2_R**	**16_G**	**10i**	85	24	>95	10	$[C_{12}H_{24}N_5O_4S]^+$	333.1465	333.1467
**12**	**2_D**	**16_G**	**10j**	75	24	>95	15	$[C_{10}H_{18}O_6N_2S]^+$	293.0802	293.0800
**13**	**2_V**	**16_G**	**10k**	30	24	>95	72	$[C_{11}H_{21}N_2O_4S]^+$	277.1216	277.1211
**14**	**2_I**	**16_G**	**10l**	30	24	>95	96	$[C_{12}H_{21}O_4N_2S]^-$	289.1217	289.1228
**15**	**2_{GG}**	**16_G**	**10m**	75	24	>95	5	$[C_{10}H_{18}N_3O_5S]^+$	292.0962	292.0957
**16**	**2_{GGG}**	**16_G**	**10n**	70	24	>95	10	$[C_{12}H_{21}N_4O_6S]^+$	349.1176	349.1168
**17**	**2_{VG}**	**16_G**	**10o**	65	24	>95	5	$[C_{13}H_{24}N_3O_5S]^+$	334.1431	334.1425
**18**	**2_{DX}**	**16_G**	**8q**	>95	2	-	-	$[C_{20}H_{27}O_8N_6S_2]^-$	543.1326	543.1329
**19**	**2_{EX}**	**16_G**	**8r**	>95	10	-	-	$[C_{22}H_{31}O_8N_6S_2]^-$	573.1796	573.1788

Table S4: Table showing yields and mass spectroscopic data for ligation of *N*-acetyl or peptidyl amidonitrile (**2**, 37.5-50 mM) and mercaptoalkyl-amino acids (37.5-50 mM) in phosphate (pH 7) or borate (pH 9) buffer (500 mM, H_2O/D_2O 9:1) at room temperature.

Ac-Gly-CN (**2$_G$**) Ligation with **16$_G$** to yield **10a**:

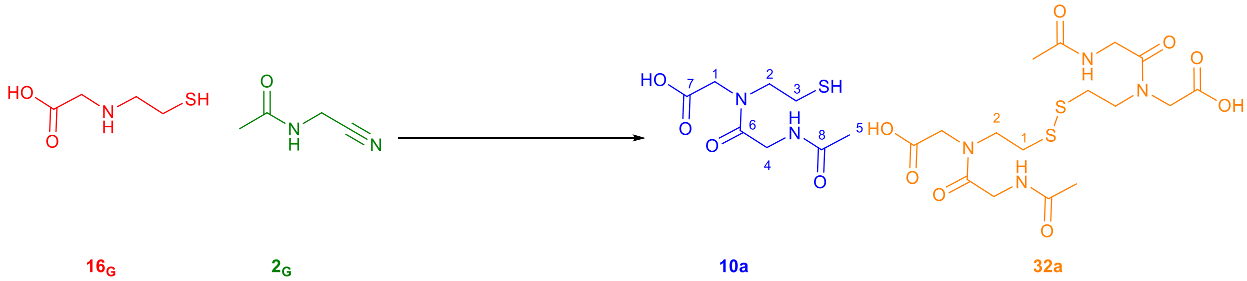

A solution of **16$_G$** (50 - 100 mM), Ac-Gly-CN **2$_G$** (70 - 130 mM) and MSM (25 mM; internal standard) in H_2O/D_2O (9:1, 1 mL) was incubated at pH 7.0 for 24 hours at room temperature. The solution was observed to rise from pH 7.0 to pH 7.8 during this period. After 24 hours, a quantitative consumption of **16$_G$** was observed, corresponding to the formation of **10a** (80-85%) and **32a** (15-20%) as a mixture of two rotamers.

Compound 10a (Rotamer A & B): ^{1}H NMR (700 MHz, H_2O/D_2O 9:1): δ_H 2.04 & 2.05 (2 × s, 3H, (C5)-H), 2.68 (m, 2H, (C3)-H) & 2.77 (t, *J* = 7.0 Hz, 2H, (C3)-H), 3.52 (m, 2H, (C2)-H) & 3.55 (t, *J* = 7.0 Hz, 2H, (C2)-H), 3.90 & 4.00 (2 × s, 2H, (C1)-H), 3.98 (app d, *J* = 4.6 Hz, (C4)-H) & 4.26 (app d, *J* = 4.0 Hz, 2H, (C4)-H).

Compound 32a: ^{1}H NMR (700 MHz, H_2O/D_2O 9:1 – partial assignment): δ_H 2.90 (m, 2H, (C1)-H), 3.68 (m, *J* = 17.0, 9.7, 7.2, 4.0 Hz, 2H, (C2)-H).

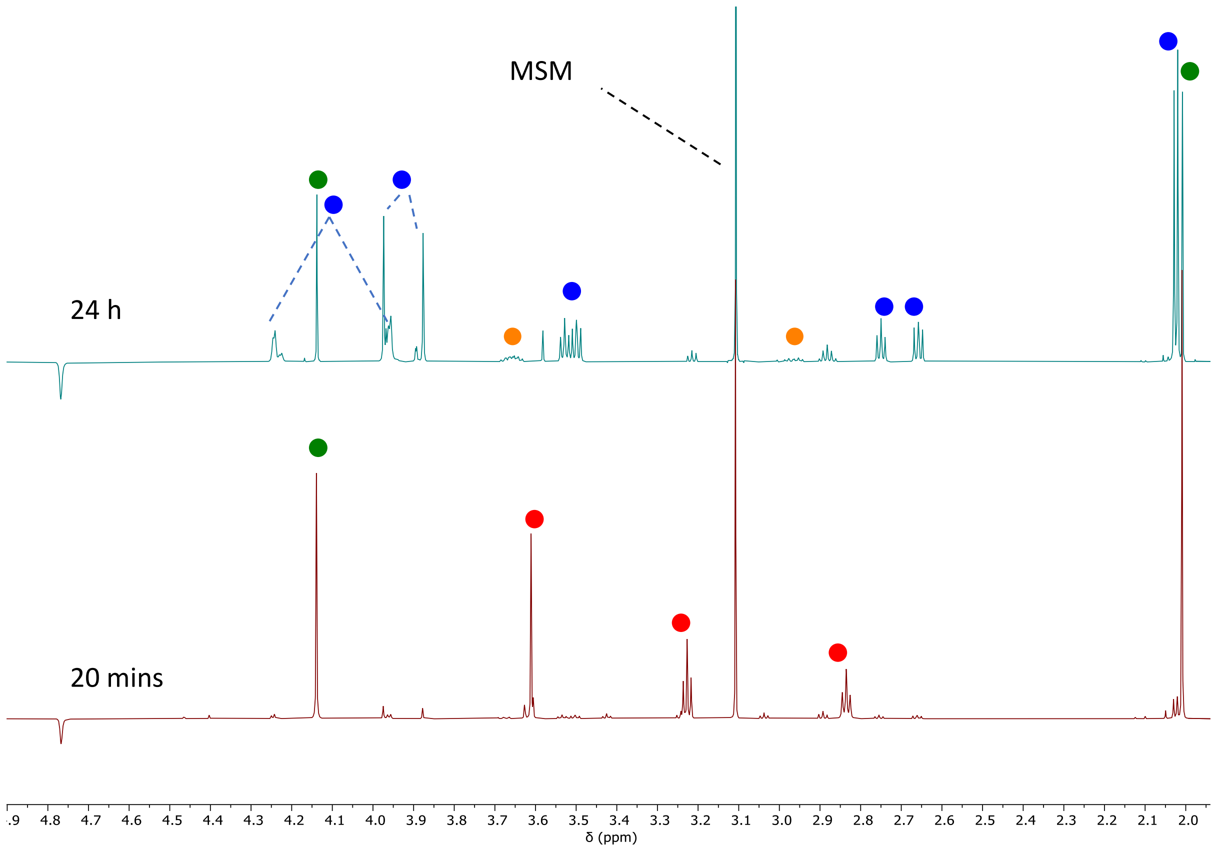

Figure S64: ^{1}H NMR (700 MHz, H_2O/D_2O 9:1, noesygppr1d, 1.9-4.9 ppm) spectra to show the reaction of $\mathbf{16_G}$ (50 mM) and Ac-Gly-CN ($\mathbf{2_G}$, 70 mM) with MSM (25 mM; internal standard) at pH 7.0 and room temperature, which yields **10a** and **32a**.

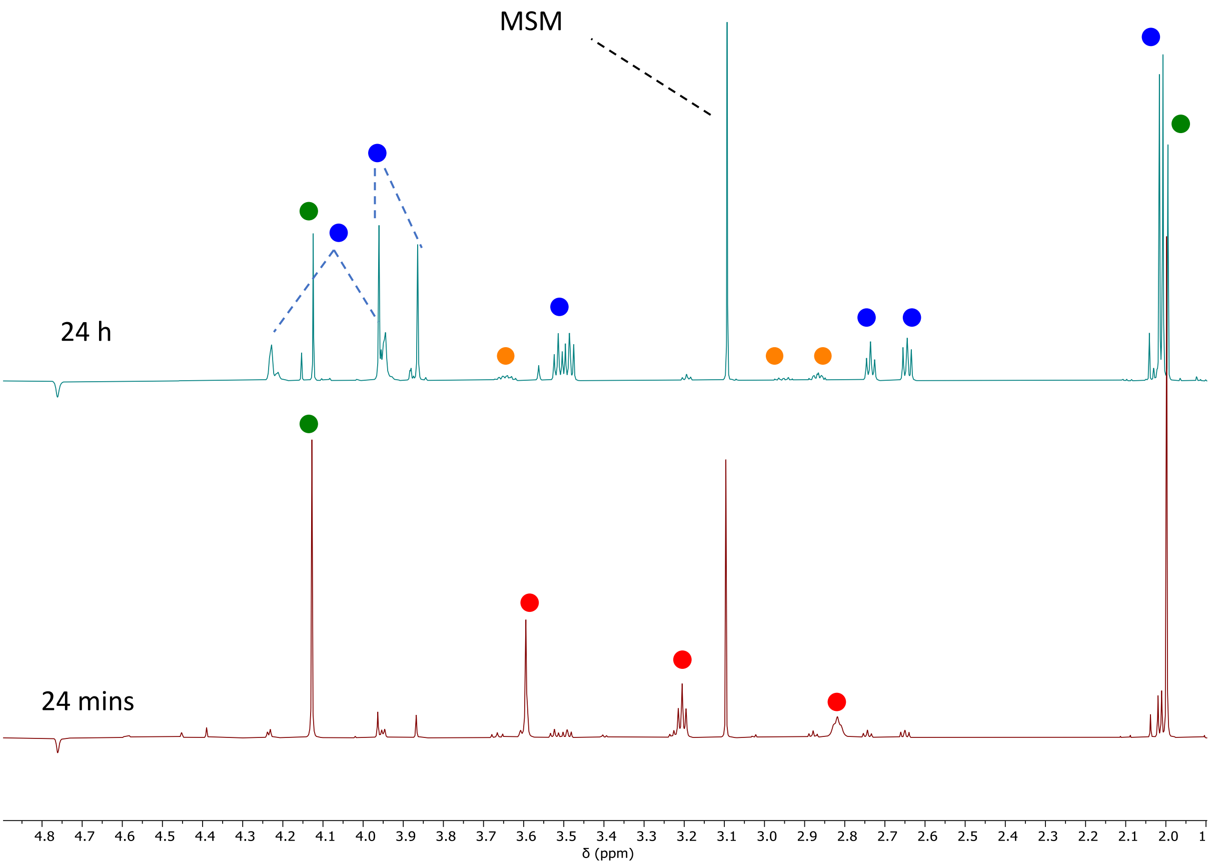

Figure S65: ^{1}H NMR (700 MHz, H_2O/D_2O 9:1, noesygppr1d, 1.9-4.9 ppm) spectra to show the reaction of **16_G** (100 mM) and Ac-Gly-CN (**2_G**, 130 mM) with MSM (25 mM; internal standard) at pH 7 and room temperature, which yields **10a** and **32a**.

A solution of **16_G** (53 mM), Ac-Gly-CN **2_G** (50 mM), TCEP (10 mM) and MSM (25 mM; internal standard) in H_2O/D_2O (9:1, 1 mL) was incubated at pH 7.0 for 7 days at room temperature. The pH of the solution was observed to drop from 7.0 to 6.0. After 24 hours, the reaction yielded **10a** (92%) in excellent yield. Upon further monitoring the reaction for 7 days, **10a** was observed in near quantitative yield (>95%).

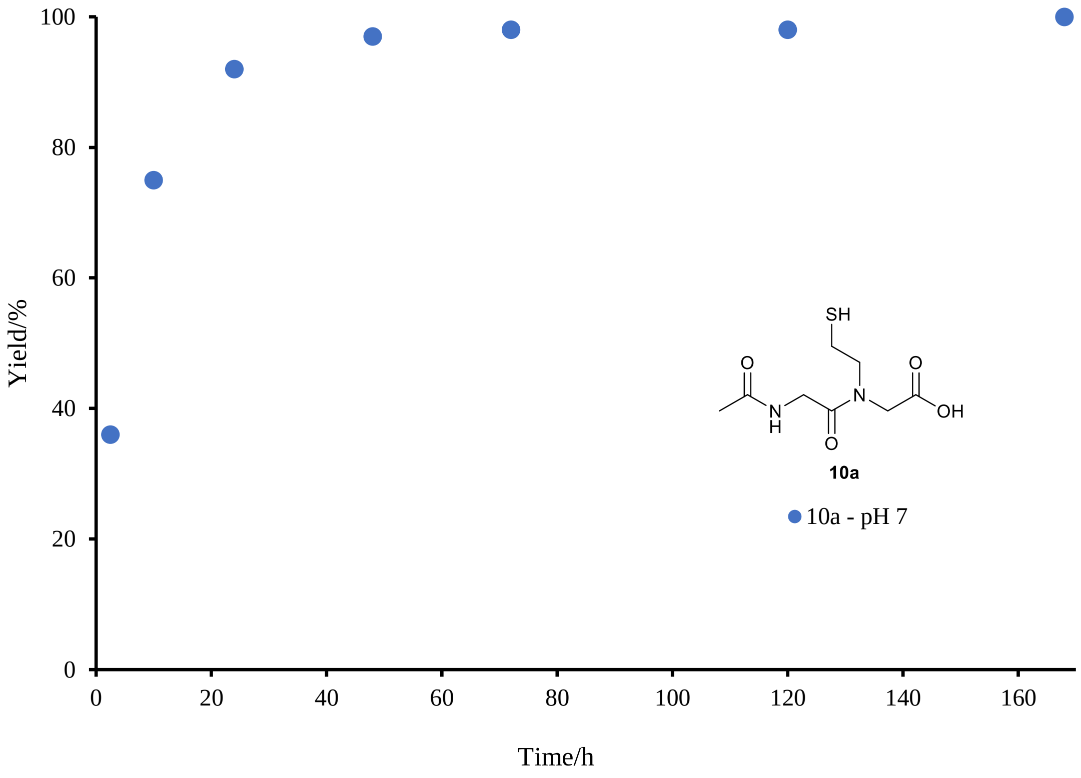

Figure S66: Graph showing the yield of formation of **10a** from **16_G** (53 mM), **2_G** (50 mM) and TCEP (10 mM) at pH 7.0 and room temperature over a period of 7 days.

A solution of **16$_G$** (100 mM), Ac-Gly-CN **2$_G$** (100 mM) and MSM (50 mM; internal standard) in phosphate buffer (1 mL, pH 7, 500 mM in H_2O/D_2O (9:1)) was incubated for 24 hours at room temperature. The reaction was then analysed by NMR spectroscopy and observed to yield **10a** (84%) (see Supplementary Figure 67-69).

Compound 10a: (Rotamer A & B): ^{1}H NMR (700 MHz, H_2O/D_2O 9:1): δ_H 2.04 & 2.05 (2 × s, 3H, (C5)-H), 2.68 & 2.77 (2 × t, *J* = 7.0 Hz, 2H, (C3)-H), 3.52 & 3.55 (2 × t, *J* = 7.0 Hz, 2H, (C2)-H), 3.90 & 4.00 (2 × s, 2H, (C1)-H), 3.98 (app d, *J* = 4.6 Hz, 2H, (C4)-H) & 4.26 (app d, *J* = 4.0 Hz, 2H, (C4)-H). **^{13}C NMR (175 MHz, H_2O/D_2O 9:1):** δ_C 21.7 (C5), 22.4 & 22.5 (C5 & 2 × C3), 41.7 & 41.2 (2 × C4), 50.6 & 52.2 (2 × C1), 51.5 (2 × C2), 171.2 & 171.6 (2 × C6), 175.1 & 175.2 (2 × C8), 176.2 & 176.7 (2 × C7).

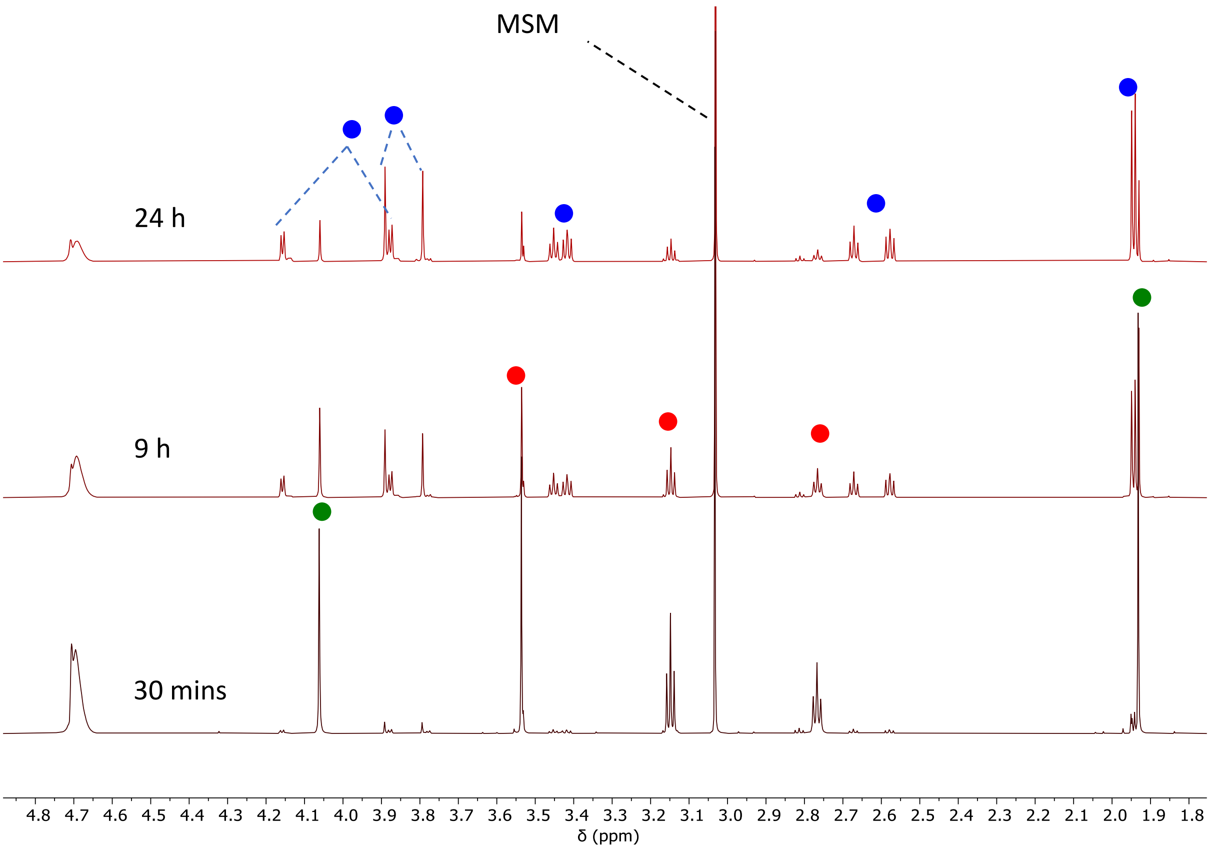

Figure S67: ^{1}H NMR (700 MHz, H_2O/D_2O 9:1, noesygppr1d, 1.8-4.9 ppm) spectra to show the reaction of **16$_G$** (100 mM) and Ac-Gly-CN (**2$_G$**, 100 mM) with MSM (50 mM; internal standard) in phosphate buffer (pH 7, 500 mM) at room temperature, which yields **10a**.

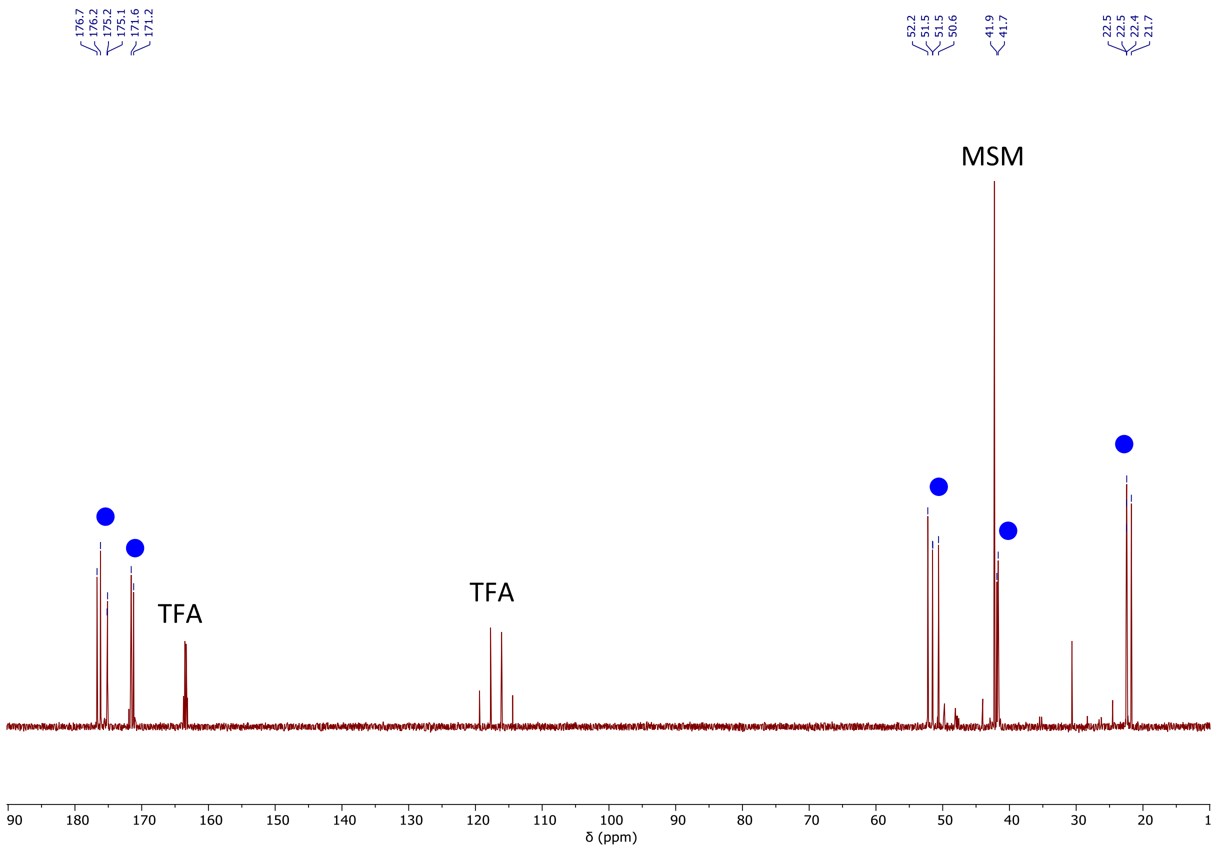

Figure S68: ^{1}H NMR (175 MHz, H_2O/D_2O 9:1, noesygppr1d, 10-190 ppm) spectrum to show the reaction of $\mathbf{16_G}$ (200 mM) and Ac-Gly-CN ($\mathbf{2_G}$, 200 mM) with MSM (100 mM; internal standard) at pH 7 and room temperature, which yields **10a**.

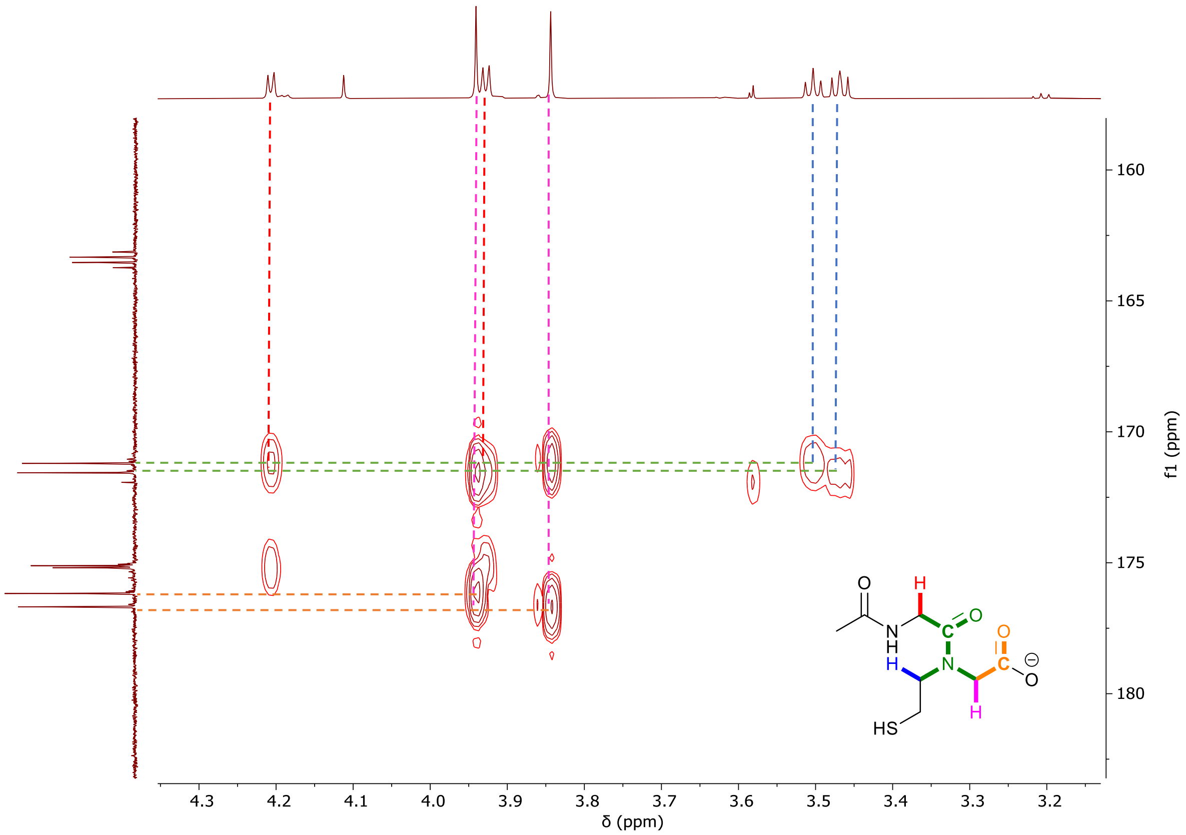

Figure S69: ^{1}H– ^{13}C HMBC (^{1}H: 700 MHz [3.1-4.3 ppm], ^{13}C: 175 MHz [155-185 ppm]) spectrum to show the diagnostic $^3J_{CH}$ and $^2J_{CH}$ coupling of H_1, H_2 and H_4 to C_6 and H_1 to C_7 at pH 7, that is characteristic of dipeptoid **10a**.

A solution of **16$_G$** (50 mM), Ac-Gly-CN **2$_G$** (50 mM) and MSM (50 mM; internal standard) in phosphate buffer (pH 7, 500 mM in H_2O/D_2O (9:1), 1 mL) was incubated for 24 hours at room temperature. The reaction was then analysed by NMR spectroscopy and observed to yield **10a** (80%) (see Supplementary Figure 70).

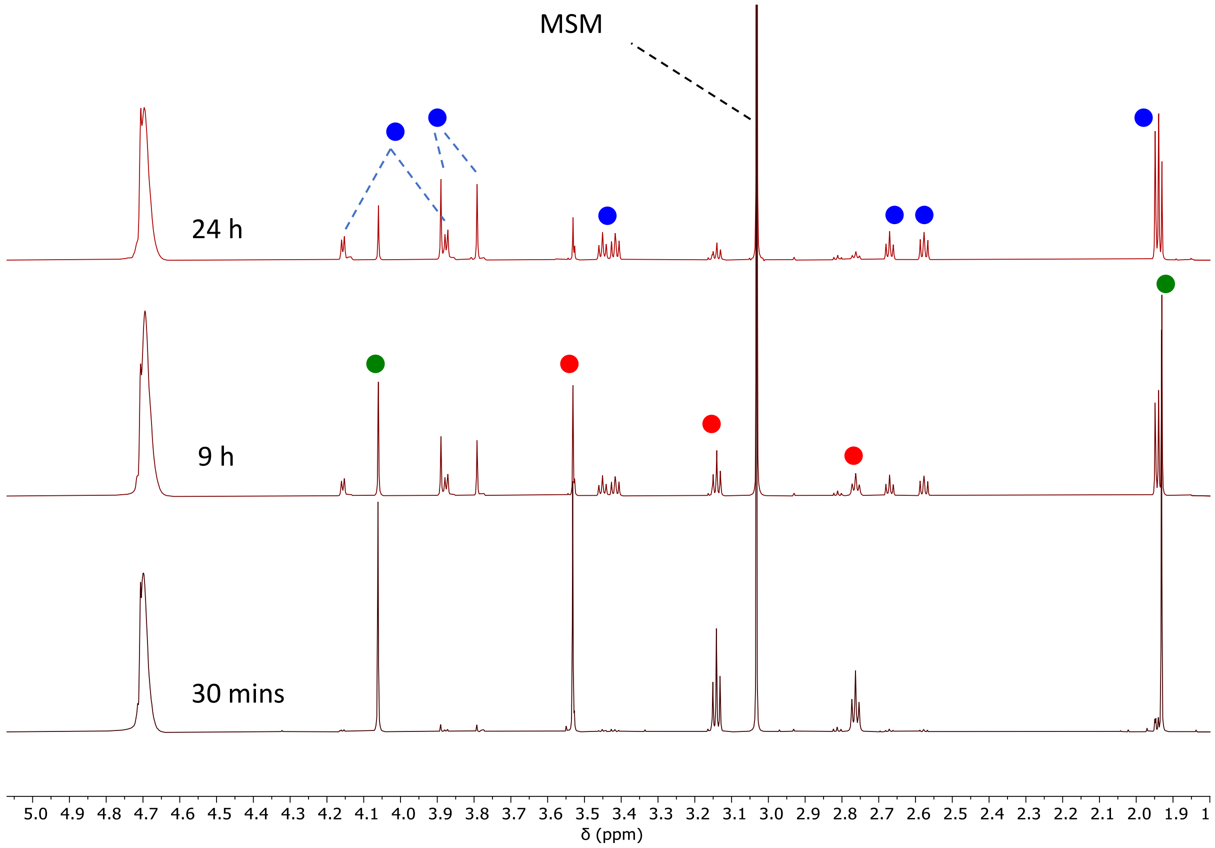

Figure S70: ^{1}H NMR (700 MHz, H_2O/D_2O 9:1, noesygppr1d, 1.8-5.1 ppm) spectra to show the reaction of **16$_G$** (50 mM) and Ac-Gly-CN (**2$_G$**, 50 mM) with MSM (50 mM; internal standard) in phosphate buffer (pH 7, 500 mM) at room temperature, which yields **10a**.

A solution of **16$_G$** (100 mM), Ac-Gly-CN **2$_G$** (100 mM) and MSM (50 mM) in phosphate buffer (2 mL, pH 7, 500 mM in H_2O/D_2O (9:1)) was incubated for 15 hours at 25, 40 or 60 °C. The reaction was then analysed by NMR spectroscopy and observed to yield **10a** in 79%, 90% and 91% yield, respectively (see Supplementary Figure 71-73). Addition of TCEP (~1 equiv.) led to no change in the product spectrum, meanwhile the addition of $K_3[Fe(CN)_6]$ (~1 equiv.) was observed to transform the product **10a** into oxidised disulfide **32a**.

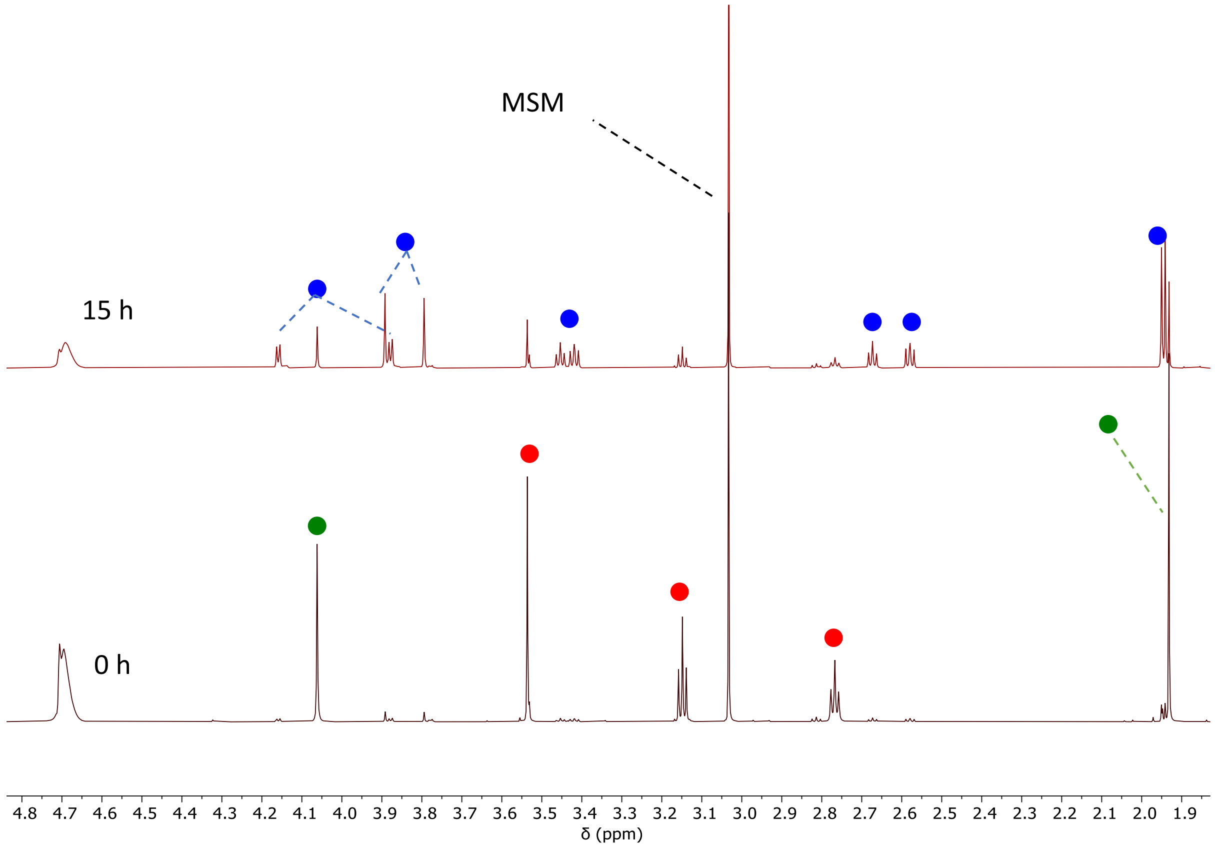

Figure S71: ^1H NMR (700 MHz, H_2O/D_2O 9:1, noesygppr1d, 1.9-4.8 ppm) spectra to show the reaction of **16$_G$** (100 mM) and Ac-Gly-CN (**2$_G$**, 100 mM) with MSM (50 mM; internal standard) in phosphate buffer (pH 7, 500 mM) at 25°C, which yields **10a**.

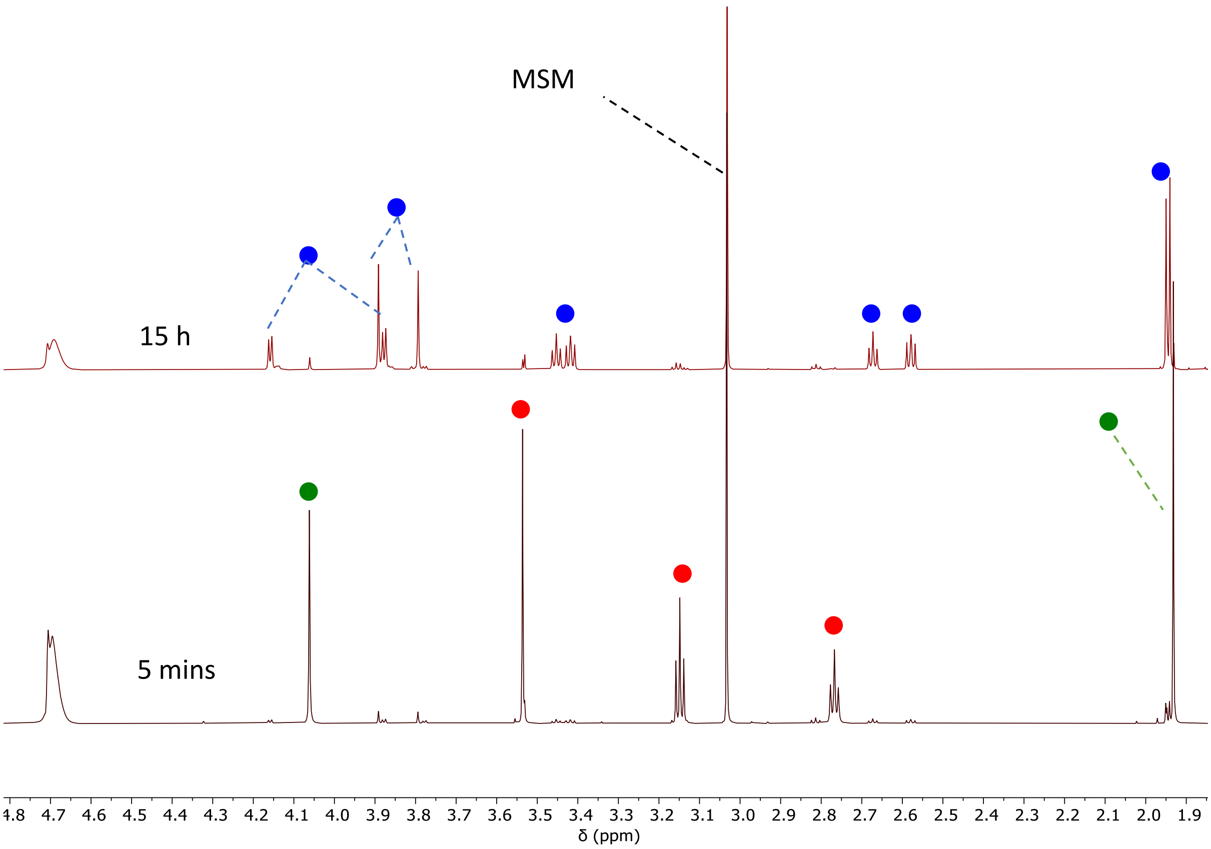

Figure S72: ^{1}H NMR (700 MHz, H_2O/D_2O 9:1, noesygppr1d, 1.9-4.8 ppm) spectra to show the reaction of **16**$_{\mathbf{G}}$ (100 mM) and Ac-Gly-CN (**2**$_{\mathbf{G}}$, 100 mM) with MSM (50 mM, internal standard) in phosphate buffer (pH 7, 500 mM) at 40 °C, which yields **10a**.

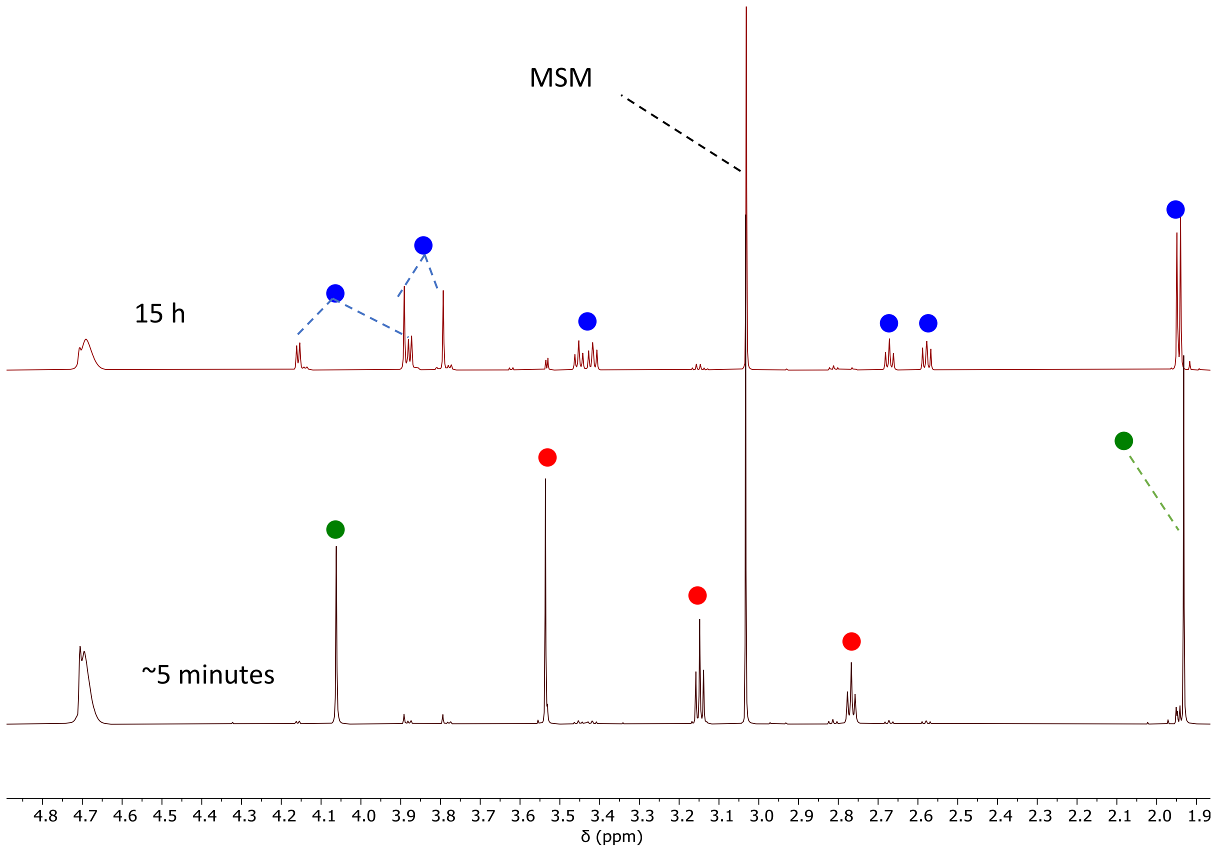

Figure S73: ^{1}H NMR (700 MHz, H_2O/D_2O 9:1, noesygppr1d, 1.9-4.9 ppm) spectra to show the reaction of $\mathbf{16_G}$ (100 mM) and Ac-Gly-CN ($\mathbf{2_G}$, 100 mM) with MSM (50 mM; internal standard) in phosphate buffer (pH 7, 500 mM) at 60°C, which yields **10a**.

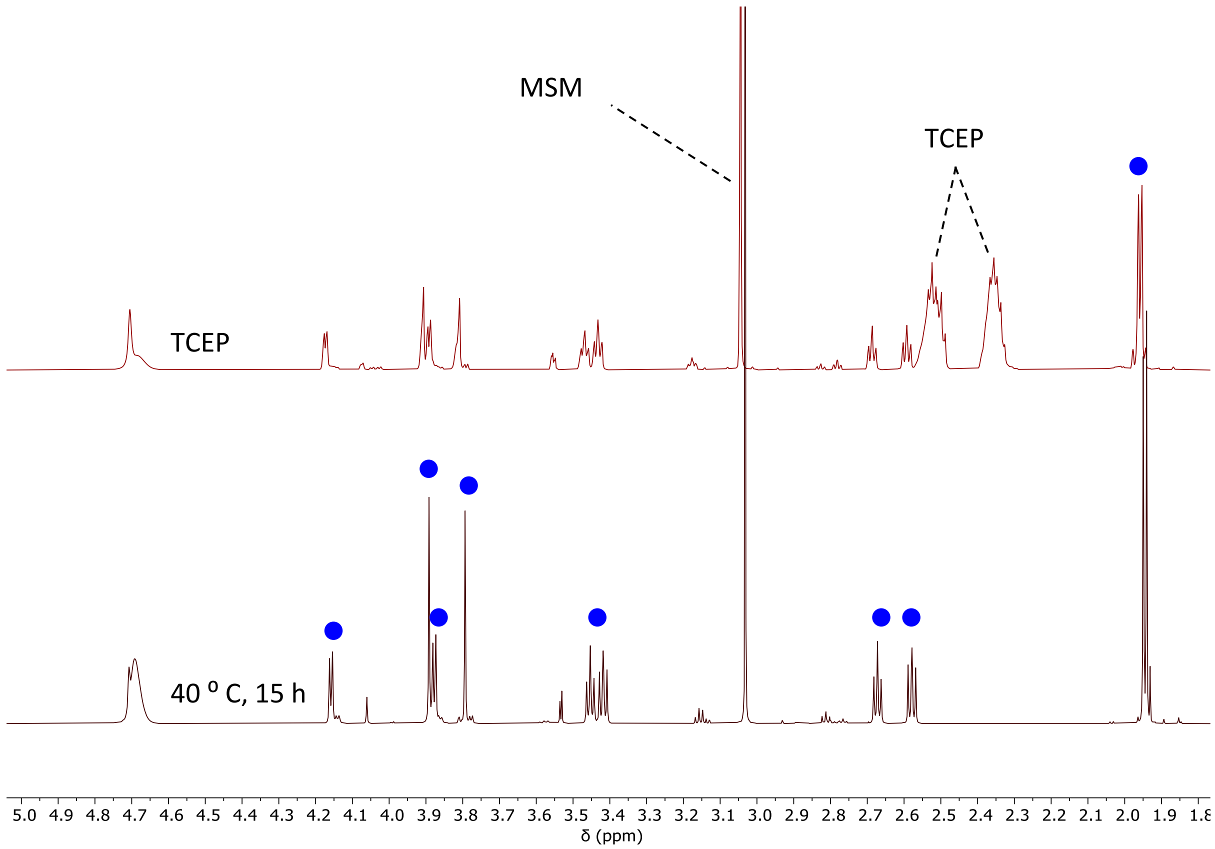

Figure S74: ^{1}H NMR (700 MHz, H_2O/D_2O 9:1, noesygppr1d, 1.9-4.9 ppm) spectra to show the effect of TCEP on **10a** (formed at 40 °C).

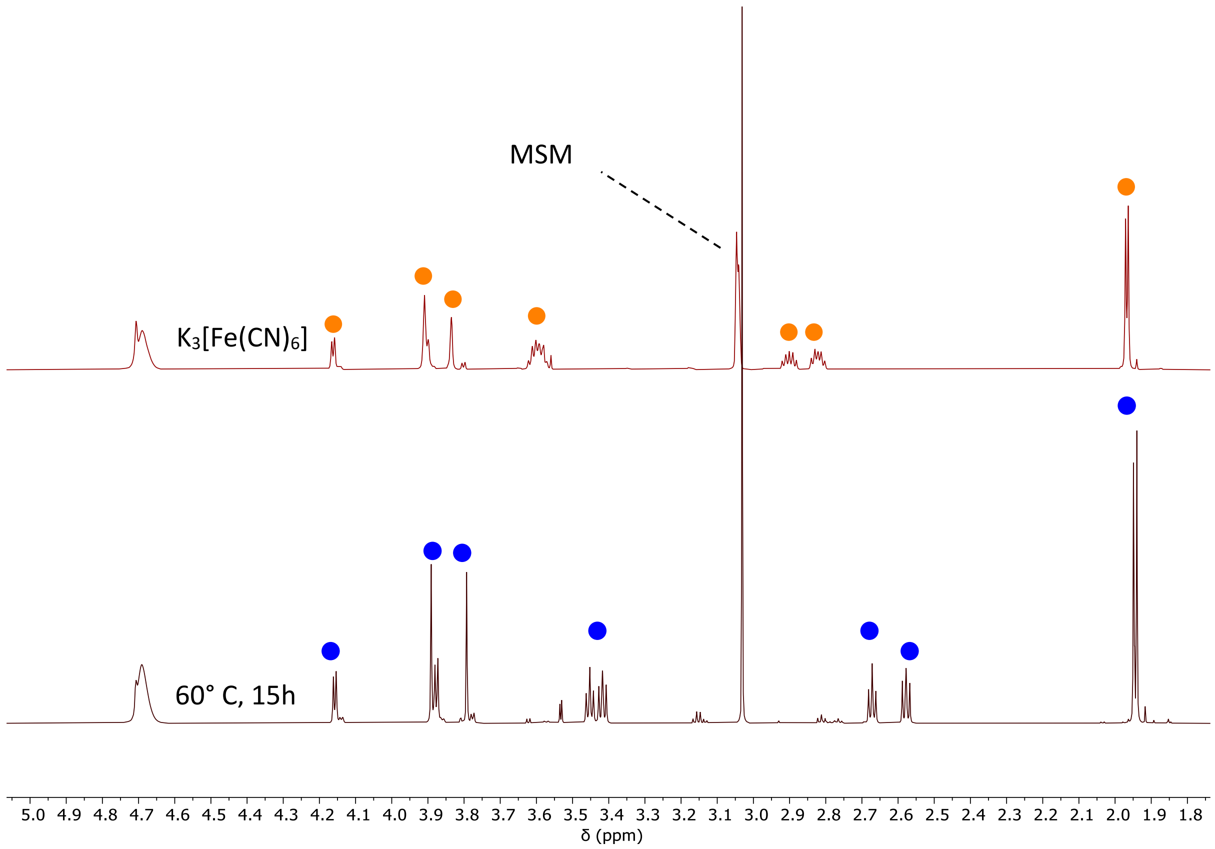

Figure S75: ^{1}H NMR (700 MHz, H_2O/D_2O 9:1, noesygppr1d, 1.8-5.0 ppm) spectra to show $K_3[Fe(CN)_6]$ oxidation of **10a** (formed at 60 °C) to disulfide **32a**.

A solution of **16$_G$** (100 mM), Ac-Gly-CN **2$_G$** (110 mM) and MSM (50 mM; internal standard) in borate buffer (1 mL, pH 9, 500 mM in H_2O/D_2O (9:1)) was incubated for 3 hours at room temperature. The reaction was then analysed by NMR spectroscopy and observed to yield **10a** in near quantitative yield (>95%) (see Supplementary Figure 76).

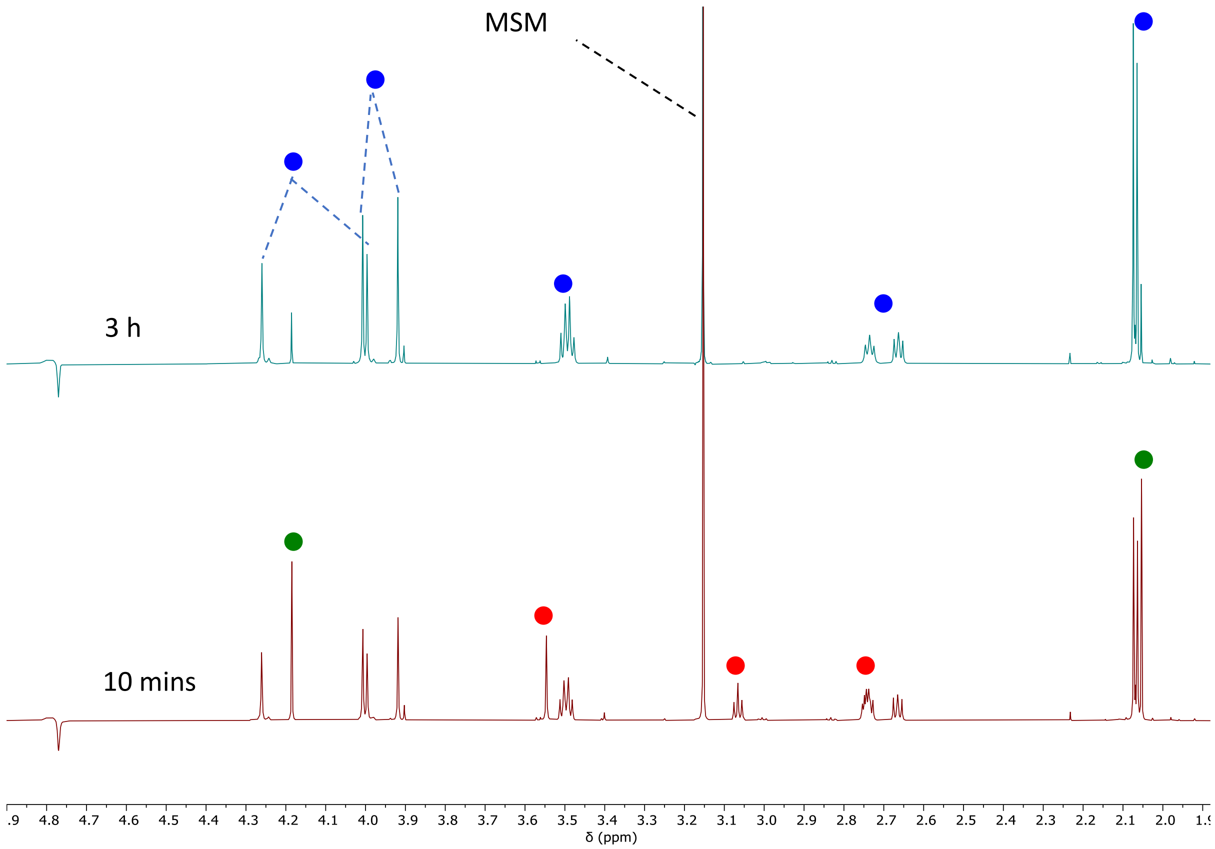

Figure S76: ^{1}H NMR (700 MHz, H_2O/D_2O 9:1, noesygppr1d, 1.9-4.9 ppm) spectra to show the reaction of **16$_G$** (100 mM), Ac-Gly-CN (**2$_G$**, 110 mM) and MSM (50 mM; internal standard) in borate buffer (pH 9, 500 mM) at room temperature, which yields **10a**.

A solution of **16$_G$** (53 mM), Ac-Gly-CN **2$_G$** (50 mM), TCEP (10 mM) and MSM (25 mM) in H_2O/D_2O (9:1, 1 mL) was incubated at pH 9.0 for 10 hours at room temperature. The solution was observed to rise from pH 9.0 to pH 9.4. After 10 hours, **10a** was observed in near quantitative yield (>95%).

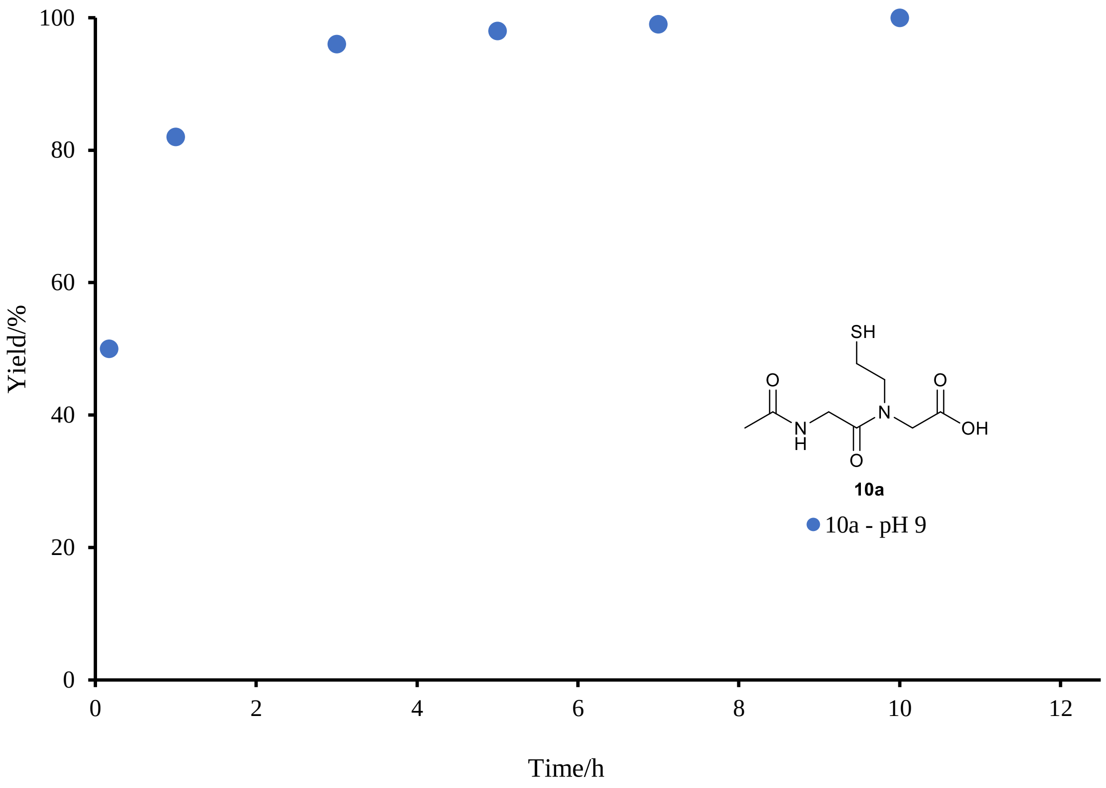

Figure S77: Graph showing the yield of formation of **10a** from **16$_G$** (53 mM), **2$_G$** (50 mM) and TCEP (10 mM) at pH 9.0 and room temperature over a period of 10 hours.

A solution of **16**$_G$ (100 mM), Ac-Gly-CN **2**$_G$ (115 mM) and MSM (25 mM; internal standard) in acetate buffer (1 mL, pH 5, 500 mM in H_2O/D_2O (9:1)) was incubated for 24 hours at room temperature. The reaction was then analysed by NMR spectroscopy and observed to yield **10a** in 7% yield. The reaction was further incubated for 7 days at room temperature and the yield of **10a**, determined by NMR spectroscopy, was observed to increase to 27% (see Supplementary Figure 78).

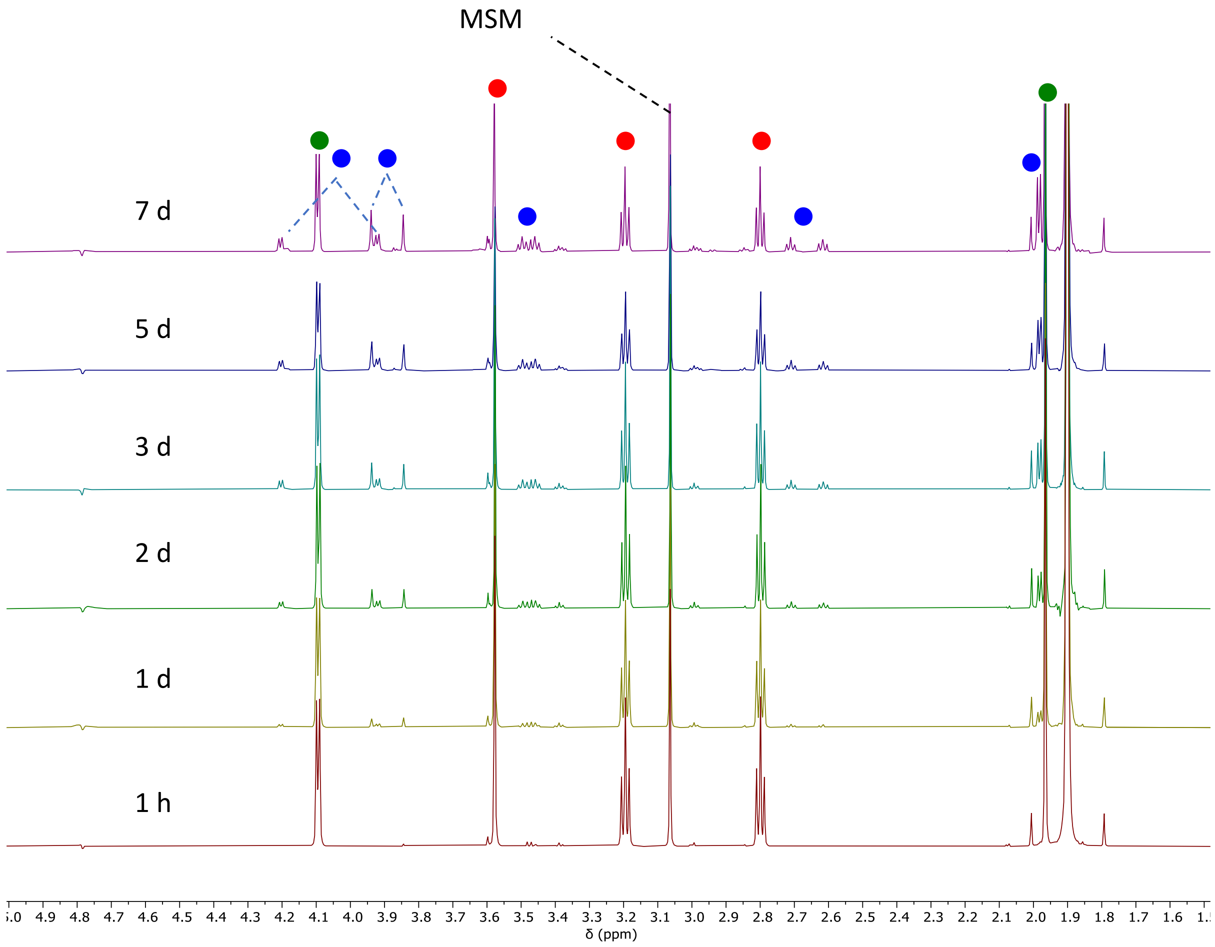

Figure S78: ^{1}H NMR (600 MHz, H_2O/D_2O 9:1, noesygppr1d, 1.5-5.0 ppm) spectra to show the reaction of **16**$_G$ (100 mM) and Ac-Gly-CN (**2**$_G$, 115 mM) with MSM (25 mM; internal standard) in acetate buffer (pH 5, 500 mM) at room temperature, which yields **10a**.

A solution of **16**$_G$ (53 mM), Ac-Gly-CN **2**$_G$ (50 mM), TCEP (10 mM) and MSM (25 mM) in H_2O/D_2O (9:1, 1 mL) was incubated at pH 9.0 for 15 days at room temperature. The solution was observed to drop from pH 5.0 to pH 4.7. After 15 days, **10a** was observed in 27% yield.

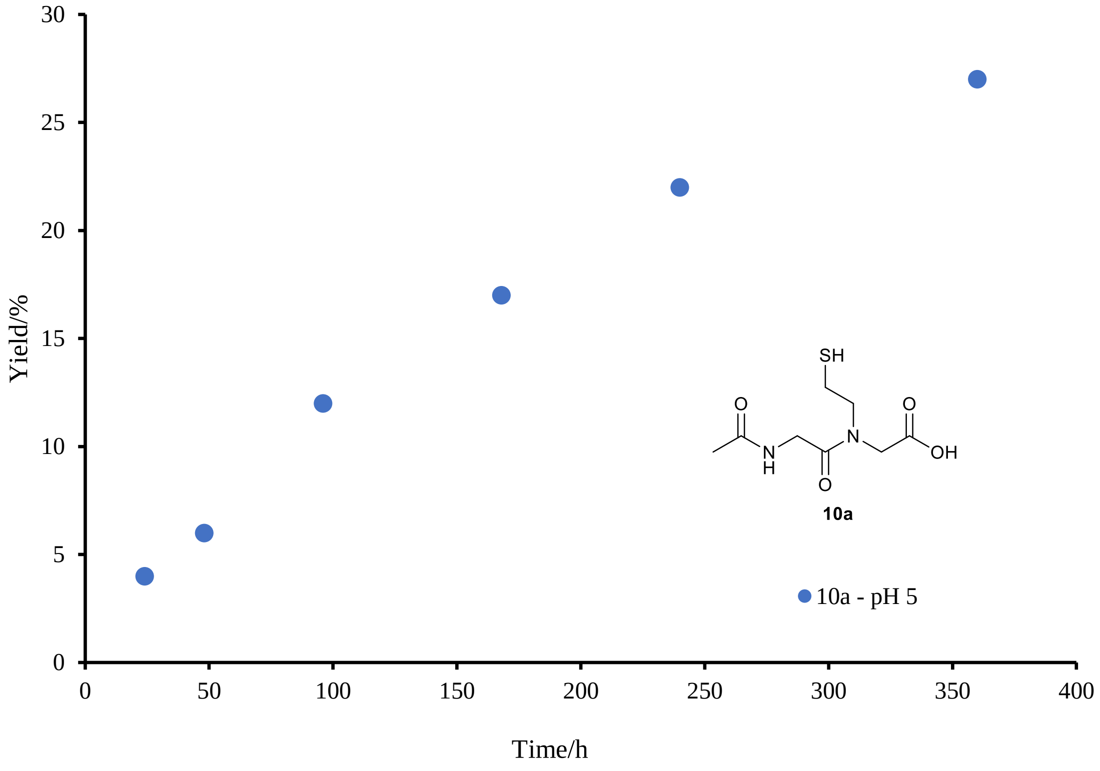

Figure S79: Graph showing the yield of formation of **10a** from **16**$_G$ (53 mM), **2**$_G$ (50 mM) and TCEP (10 mM) at pH 5.0 and room temperature over a period of 15 days.

Intermolecular CPL with **2**$_G$ and **33**$_G$ to yield **34**$_{GG}$:

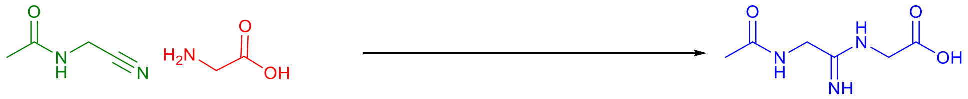

2$_G$ **33**$_G$ **34**$_{GG}$

A solution of Glycine **33**$_G$ (50 mM), Ac-Gly-CN **2**$_G$ (50 mM), β-mercapto propanoic acid (MPA, 50 mM) and MSM (12.5 mM; internal standard) in H_2O/D_2O (9:1, 2 mL) was incubated at pH 7.0 or 8.5 at room temperature for 7 days. The solutions were observed to rise from pH 7.0 to 7.8 and from pH 8.5 to 9.0, respectively. After 7 days **34**$_{GG}$ was observed in 92% (pH 8.5) and 67% (pH 7.0) yield.

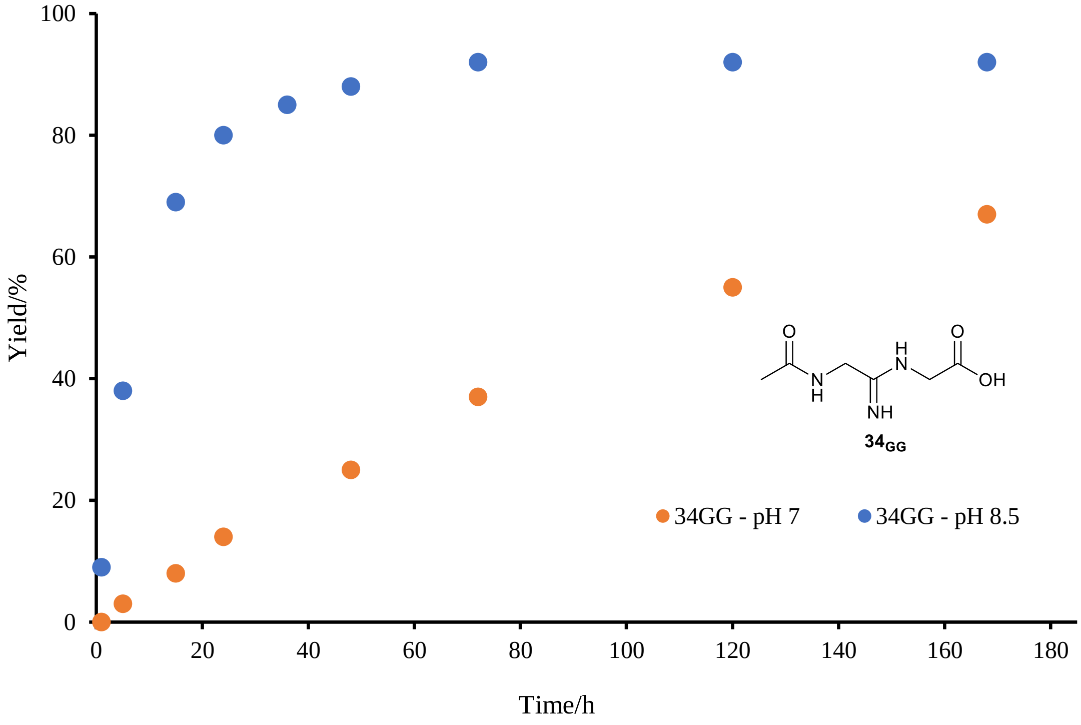

Figure S80: Graph to show the ligation yield of **34**$_{GG}$ over time from glycine (**33**$_G$, 50 mM), **2**$_G$ (50 mM) and MPA (50 mM) at pH 7.0-8.5 and at room temperature.

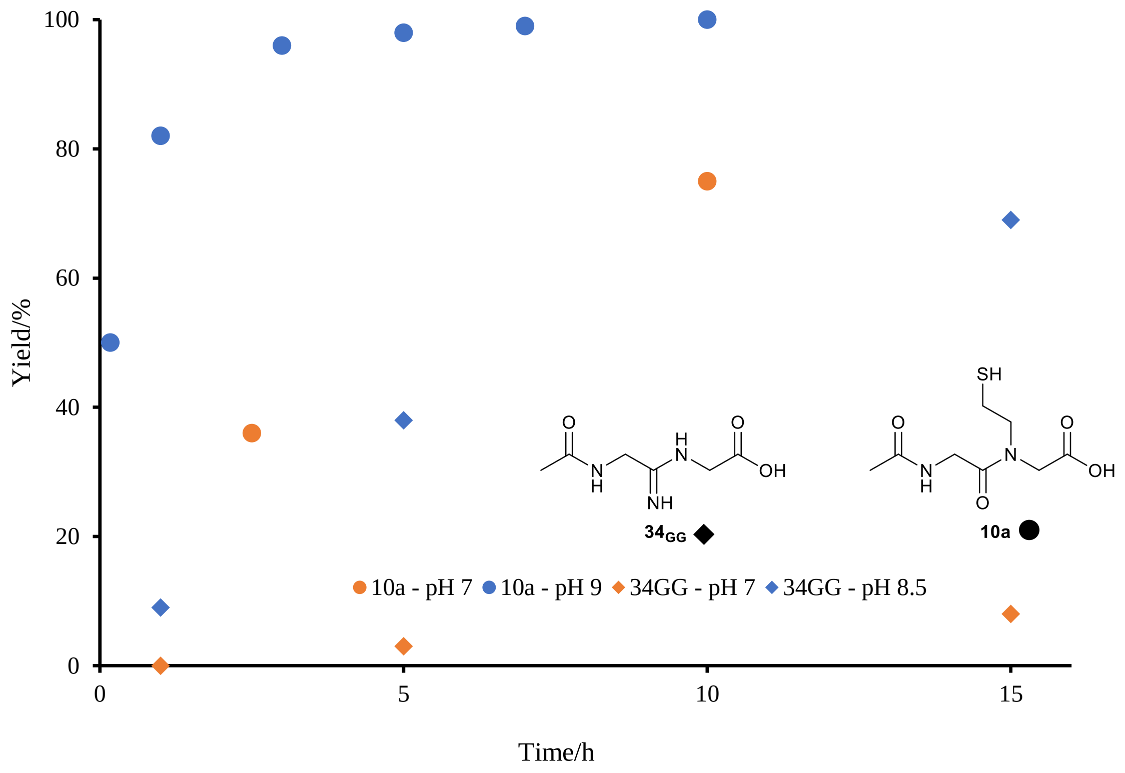

Figure S81: The enhanced rate of intramolecular and intermolecular thiol catalysis nitrile ligation. Intramolecular: The formation of peptoid (**10a**; circles) from Ac-Gly-CN (**2**$_{G}$, 53 mM) and *N*-thioethyl-glycine (**16**$_{G}$, 50 mM) with TCEP (10 mM) at room temperature and pH 9 (blue circles) or pH 7 (orange circles). **Intermolecular:** The formation of peptide-amidine **34**$_{GG}$ (diamonds) from glycine (**33**$_{G}$, 50 mM) and Ac-Gly-CN (**2**$_{G}$, 50 mM) with β-mercapto propanoic acid (MPA, 50 mM) at room temperature and pH 8.5 (blue diamonds) or pH 7 (orange diamonds).

32_G ligation of Ac-Gly-CN **2_G** to yield **33a**:

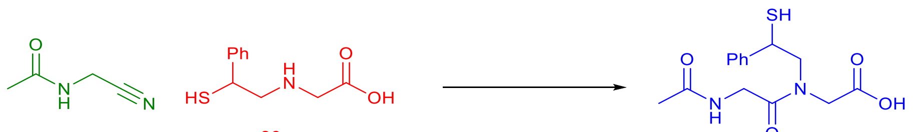

2_G **30_G** **31a**

A solution of **30_G** (25 mM), Ac-Gly-CN **2_G** (25 mM) and MSM (12.5 mM; internal standard) in phosphate or borate buffer (1 mL, pH 7 or 9 respectively, 500 mM in H_2O/D_2O (9:1)) was incubated for 24 hours at room temperature. The reaction was then analysed by NMR spectroscopy: at pH 7, **31a** (82%) was observed; and at pH 9, **31a** (91%) was observed (see Supplementary Figure 82-83).

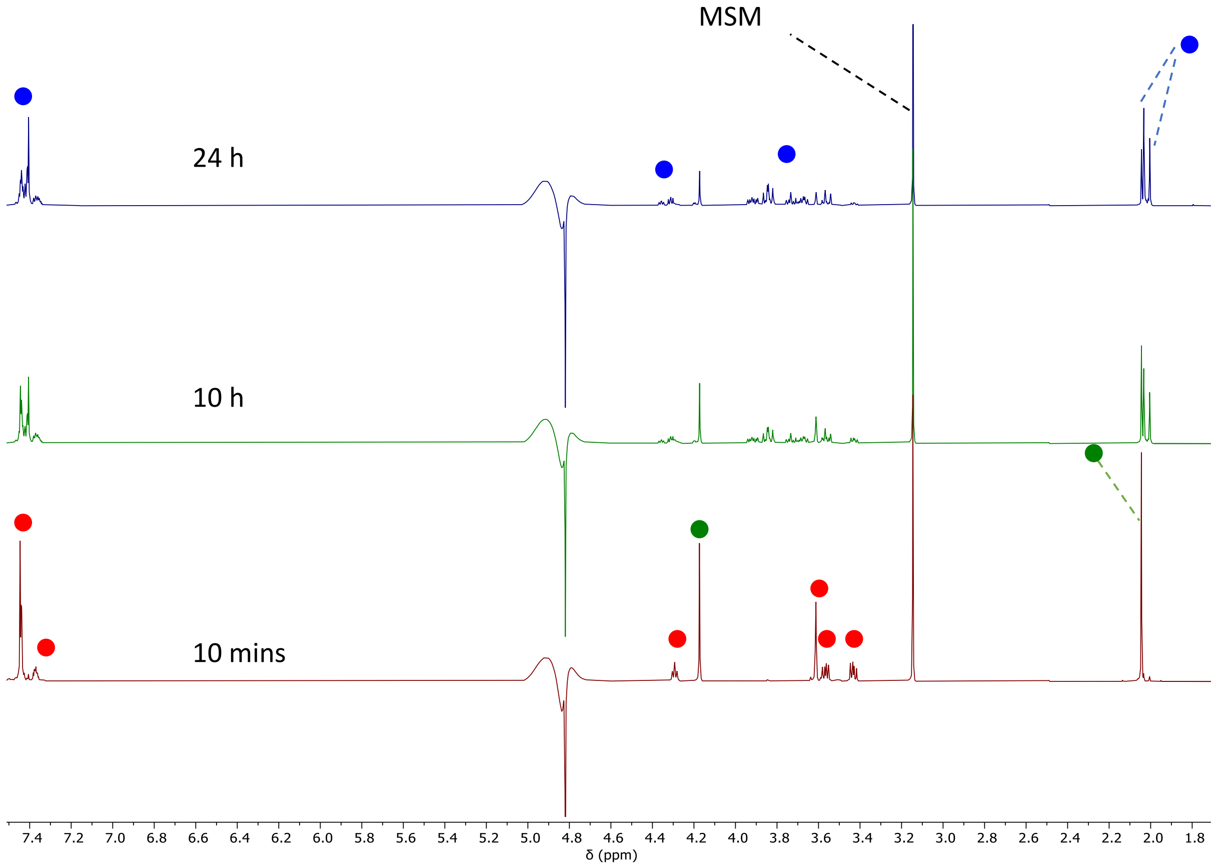

Figure S82: ^1H NMR (700 MHz, H_2O/D_2O 9:1, noesygppr1d, 1.7-7.5 ppm) spectra to show the reaction of **30_G** (25 mM) and **2_G** (25 mM) with MSM (12.5 mM; internal standard) in phosphate buffer (pH 7, 500 mM) at room temperature, which yields **31a**.

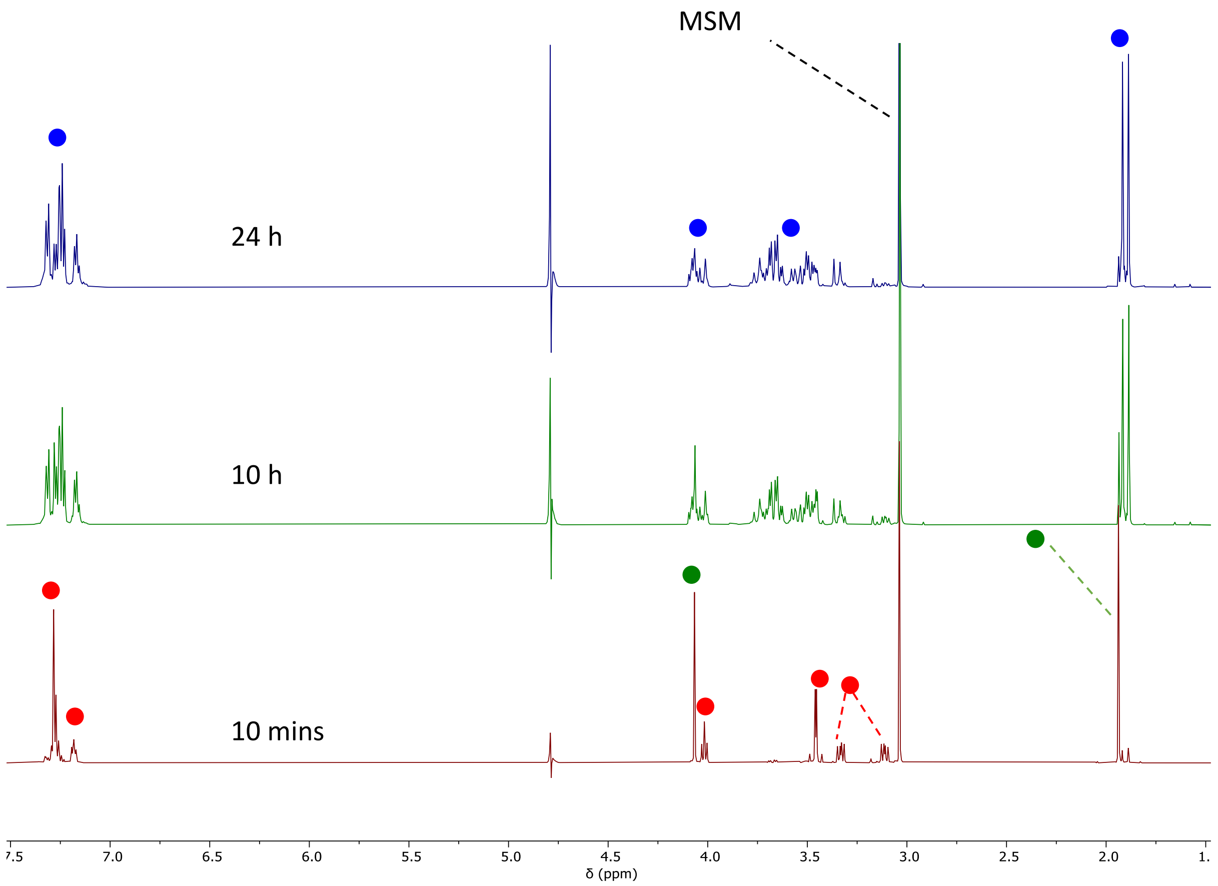

Figure S83: ^{1}H NMR (600 MHz, H_2O/D_2O 9:1, noesygppr1d, 1.5-7.5 ppm) spectra to show the reaction of $\mathbf{30_G}$ (25 mM) and $\mathbf{2_G}$ (25 mM) with MSM (12.5 mM; internal standard) in borate buffer (pH 9, 500 mM) at room temperature, which yields **31a**.

Selective ligation of **2_G** with **16_G** in presence of **17_G**:

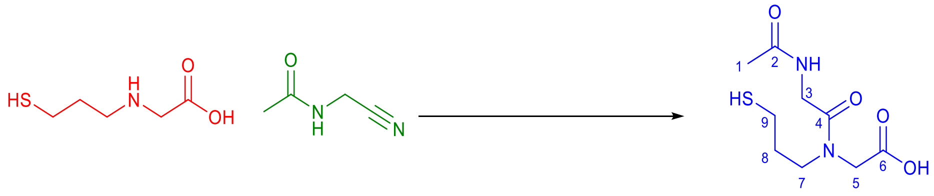

17_G 2_G **24a**

A solution of **17_G** (37.5 mM), Ac-Gly-CN **2_G** (50 mM), TCEP (50 mM) and MSM (25 mM; internal standard) in phosphate or borate buffer (1 mL, pH 7 or 9 respectively, 500 mM, H_2O/D_2O (9:1)) was incubated at room temperature. The reaction was monitored by NMR spectroscopy and observed to yield: at pH 7, peptoid **24a** (75%) after 2 days; and at pH 9, peptoid **24a** (>95%) in near-quantitative yield after 10 hours (see Supplementary Figures 84-86).

Compound 24a (Rotamer A & B): ^{1}H NMR (600 MHz, H_2O/D_2O 9:1, pH 9): δ_H 1.83 & 1.93 (2 × m, 2H, (C8)-H), 2.07 & 2.08 (2 × s, 3H, (C1)-H), 2.53 (t, J = 7.1 Hz, 2H, (C9)-H) & 2.59 (t, J = 6.9 Hz, 2H, (C9)-H), 3.47 (overlapping t, J = 7.1 Hz, 2H, (C7)-H), 3.92 & 3.97 (2 × s, 2H, (C5)-H), 3.98 & 4.26 (2 × s, 2H, (C3)-H).

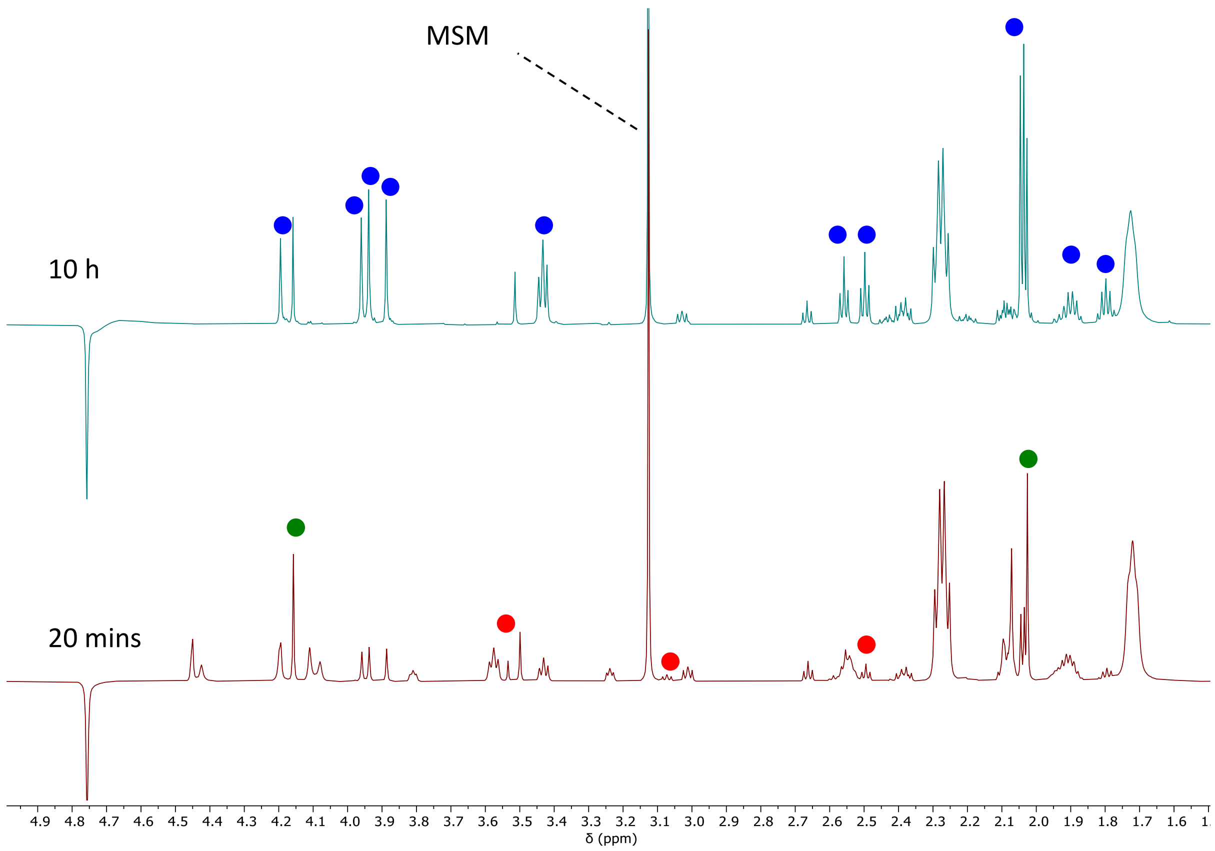

Figure S84: ^{1}H NMR (600 MHz, H_2O/D_2O 9:1, noesygppr1d, 1.5-5.0 ppm) spectra to show the reaction of **17**$_{\mathbf{G}}$ (37.5 mM), Ac-Gly-CN (**2**$_{\mathbf{G}}$, 50 mM) and TCEP (50 mM) with MSM (25 mM; internal standard) in borate buffer (pH 9, 500 mM) at room temperature, which yields peptoid **24a**.

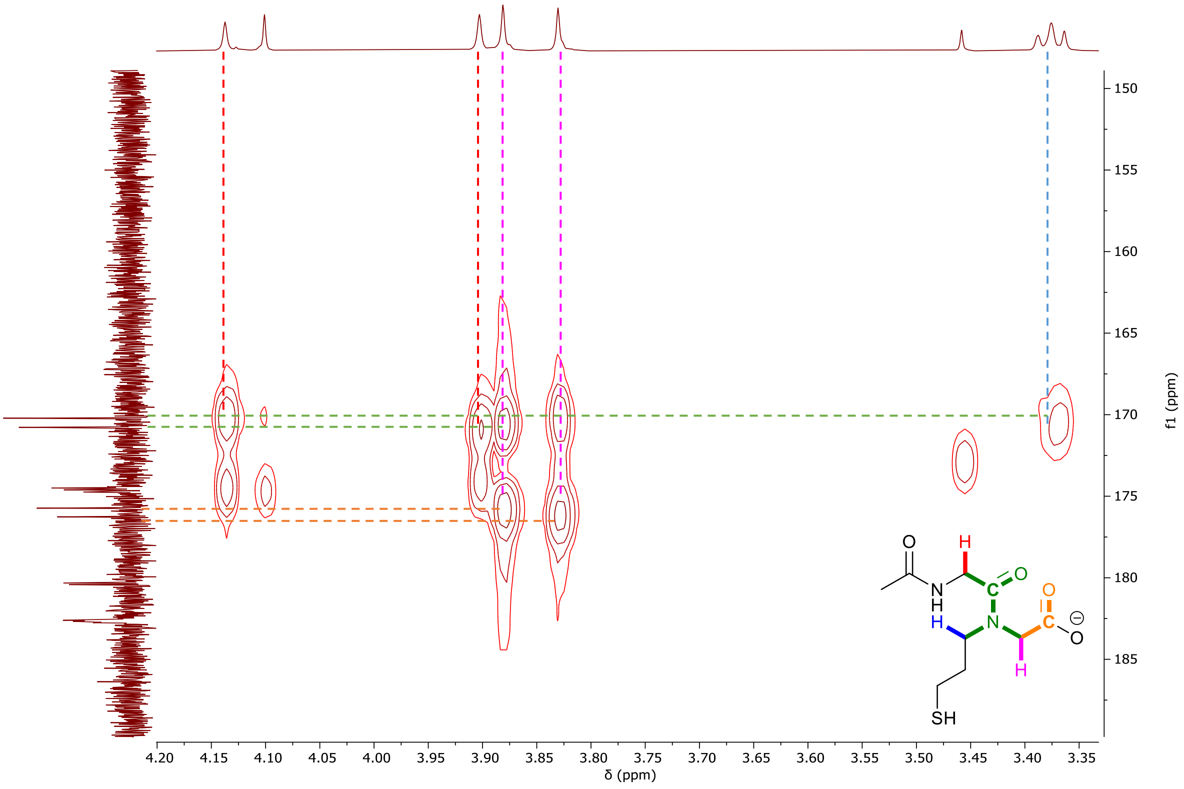

Figure S85: ^{1}H– ^{13}C HMBC (^{1}H: 600 MHz [3.3-4.2 ppm], ^{13}C: 150 MHz [150-190 ppm]) spectrum to show the diagnostic $^{3}J_{CH}$ and $^{2}J_{CH}$ coupling of α-Gly, α-Gly and *N*-CH_2 to tertiary amide carbon at pH 9, that is characteristic of peptoid **24a**.

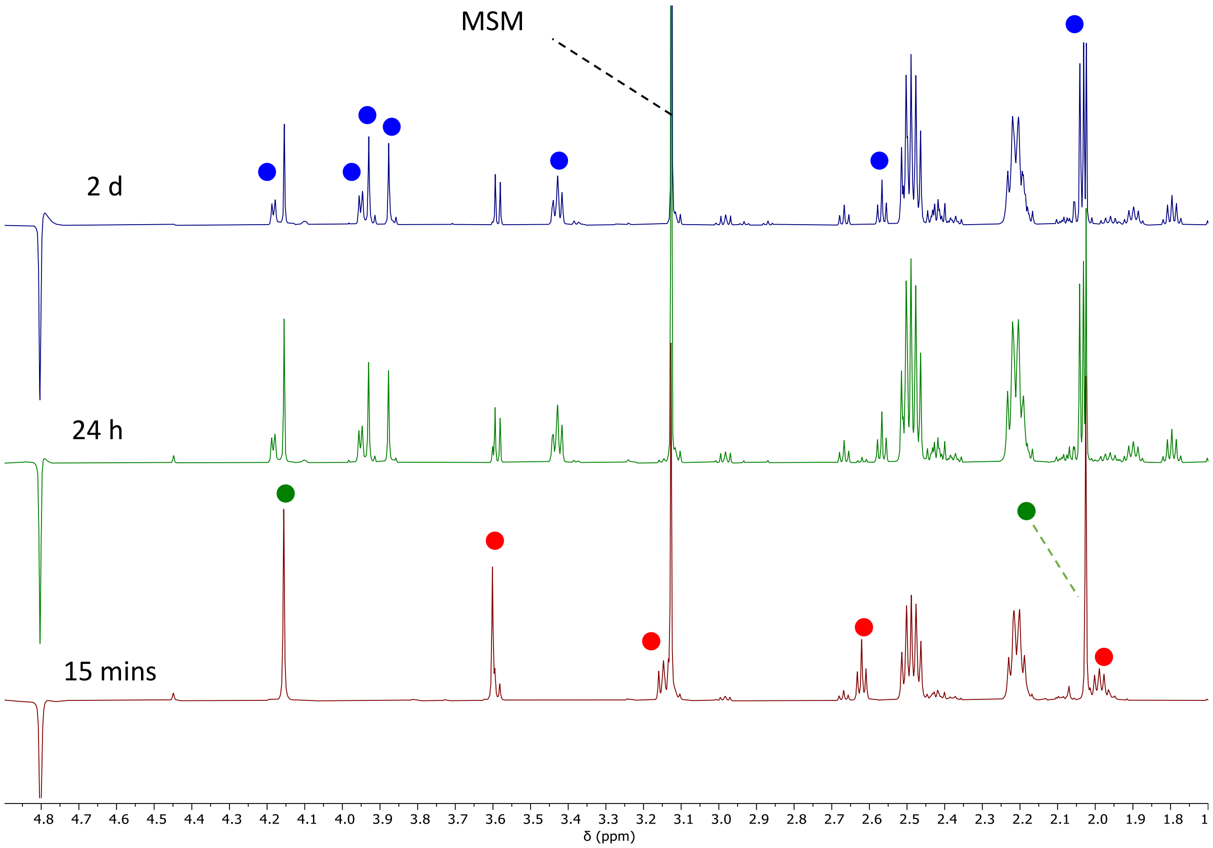

Figure S86: ^{1}H NMR (600 MHz, H_2O/D_2O 9:1, noesygppr1d, 1.7-4.9 ppm) spectra to show the reaction of **17**$_{G}$ (37.5 mM), Ac-Gly-CN (**2**$_{G}$, 50 mM) and TCEP (50 mM) with MSM (25 mM; internal standard) in phosphate buffer (pH 7, 500 mM) at room temperature, which yields peptoid **24a**.

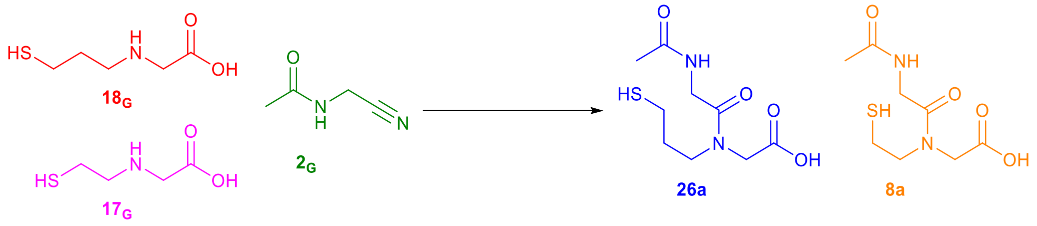

A solution of **16G** (50 mM), **17G** (50-100 mM), Ac-Gly-CN **2G** (50 mM), TCEP (50 mM) and MSM (25 mM; internal standard) in phosphate buffer (1 mL, pH 7, 500 mM, H_2O/D_2O (9:1)) was incubated for 24 hours at room temperature. The reaction was then analysed by NMR spectroscopy and observed to yield: **10a/24a** (77:23) when equimolar **17G** (50 mM) and **16G** (50 mM) were reacted; and **10a/24a** (7:3) when **17G** (100 mM) and **16G** (50 mM) were reacted (see Supplementary Figure 87-88).

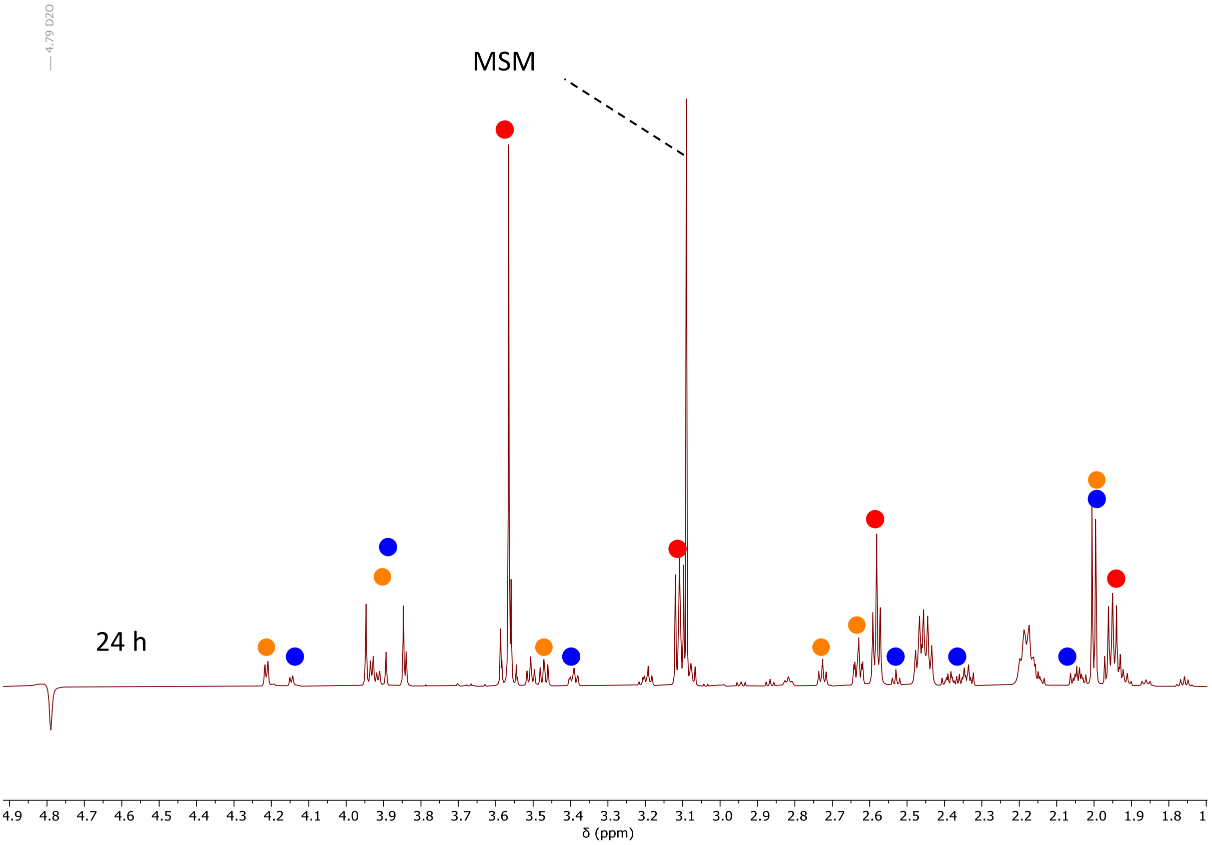

Figure S87: ^{1}H NMR (700 MHz, H_2O/D_2O 9:1, noesygppr1d, 1.7-4.9 ppm) spectrum to show the reaction of **17G** (100 mM), **16G** (50 mM), Ac-Gly-CN (**2G**, 50 mM) and TCEP (50 mM) with MSM (25 mM; internal standard) in phosphate buffer (pH 7, 500 mM) at room temperature, which yielded a mixture of **10a** and **24a**.

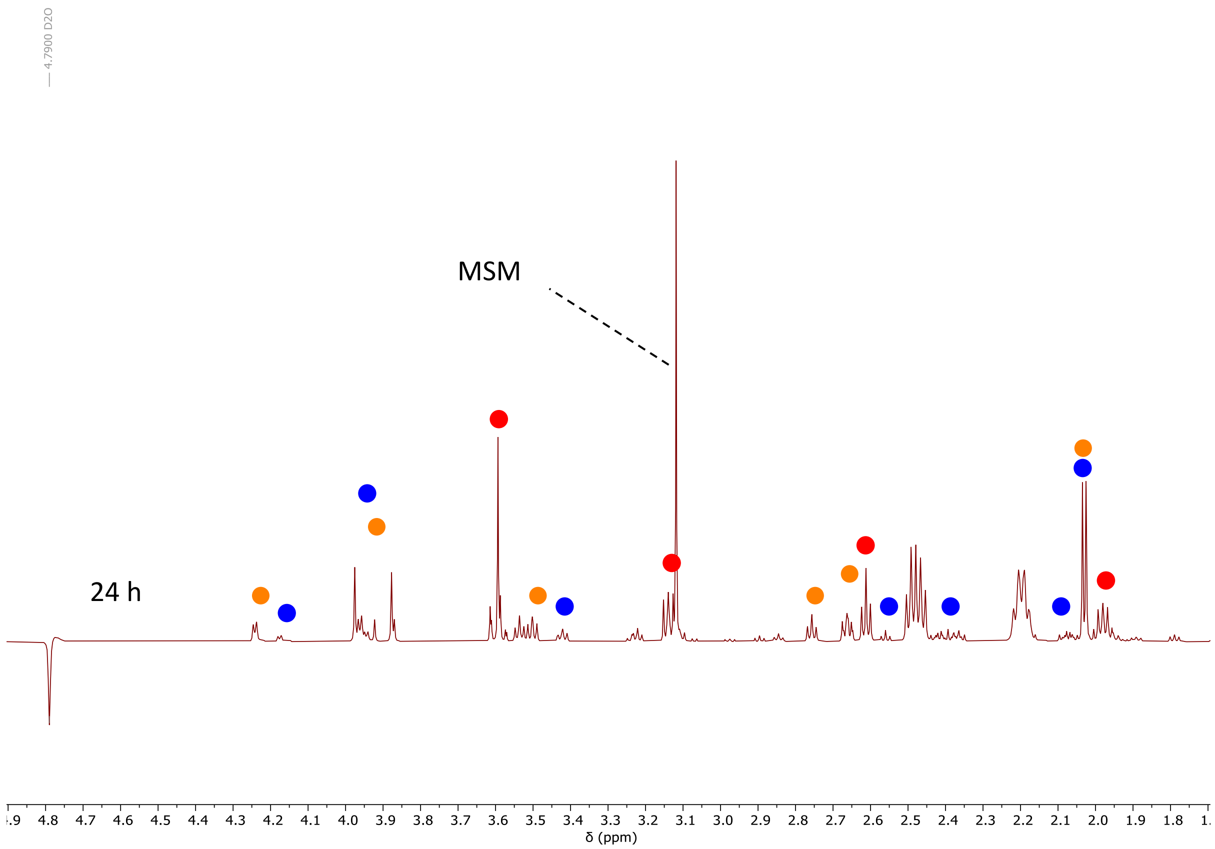

Figure S88: ^{1}H NMR (600 MHz, H_2O/D_2O 9:1, noesygppr1d, 1.7-4.9 ppm) spectrum to show the reaction of $\mathbf{17_G}$ (50 mM), $\mathbf{16_G}$ (50 mM), Ac-Gly-CN ($\mathbf{2_G}$, 50 mM) and TCEP (50 mM) with MSM (25 mM; internal standard) in phosphate buffer (pH 7, 500 mM) at room temperature, which yielded a mixture of **10a** and **24a**.

Ac-Gly-CN (2_G) Ligation with 16_A to yield **10b**:

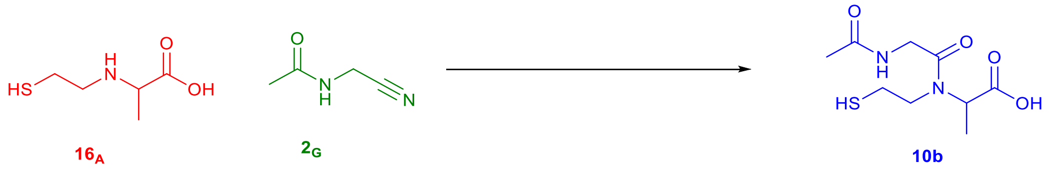

A solution of **16**$_A$ (50 mM), Ac-Gly-CN **2**$_G$ (50 mM) and MSM (6.25 mM; internal standard) in phosphate buffer (2 mL, pH 7, 500 mM in H_2O/D_2O (9:1)) was incubated for 3 days at room temperature. The reaction was then analysed by NMR spectroscopy and observed to yielded **10b** (70%) after 3 days (see Supplementary Figure 89-90).

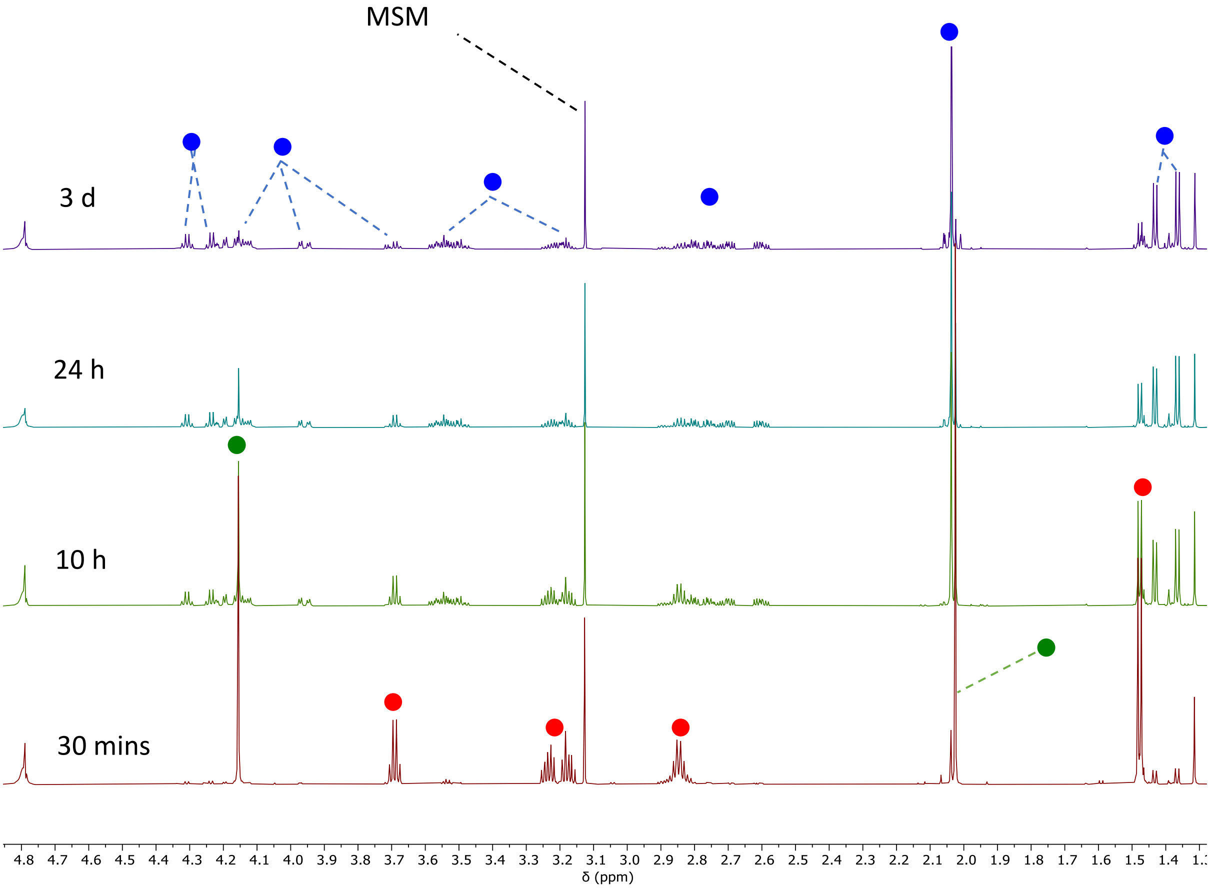

Figure S89: ^{1}H NMR (700 MHz, H_2O/D_2O 9:1, noesygppr1d, 1.3-4.8 ppm) spectra to show the reaction of **16**$_A$ (50 mM) and Ac-Gly-CN (**2**$_G$, 50 mM) with MSM (6.25 mM; internal standard) in phosphate buffer (pH 7, 500 mM) at room temperature, which yields **10b**.

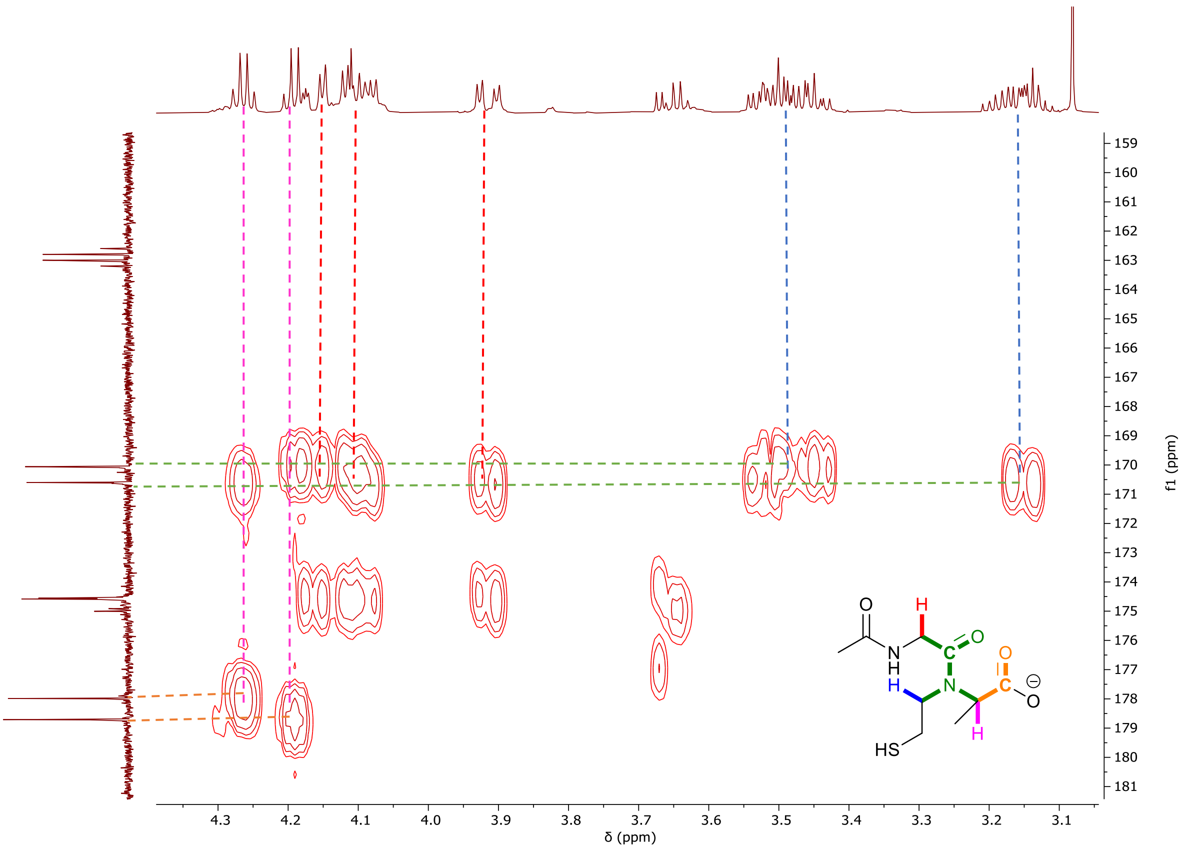

Figure S90: ^{1}H– ^{13}C HMBC (^{1}H: 700 MHz [3.1-4.4 ppm], ^{13}C: 175 MHz [159-181ppm]) spectrum to show the diagnostic $^3J_{CH}$ and $^2J_{CH}$ coupling of α-Gly, α-Ala and *N*-CH_2 to tertiary amide carbon at pH 7, that is characteristic of the dipeptoid **10b**.

A solution of **16**$_{\mathbf{A}}$ (43 mM), Ac-Gly-CN **2**$_{\mathbf{G}}$ (50 mM), TCEP (10 mM) and KHPH (25 mM; internal standard) in borate buffer (1 mL, pH 9, 500 mM in H_2O/D_2O (9:1)) was incubated for 5 hours at room temperature. The reaction was then analysed by NMR spectroscopy and observed to yield **10b** (>95%) in near quantitative yield.

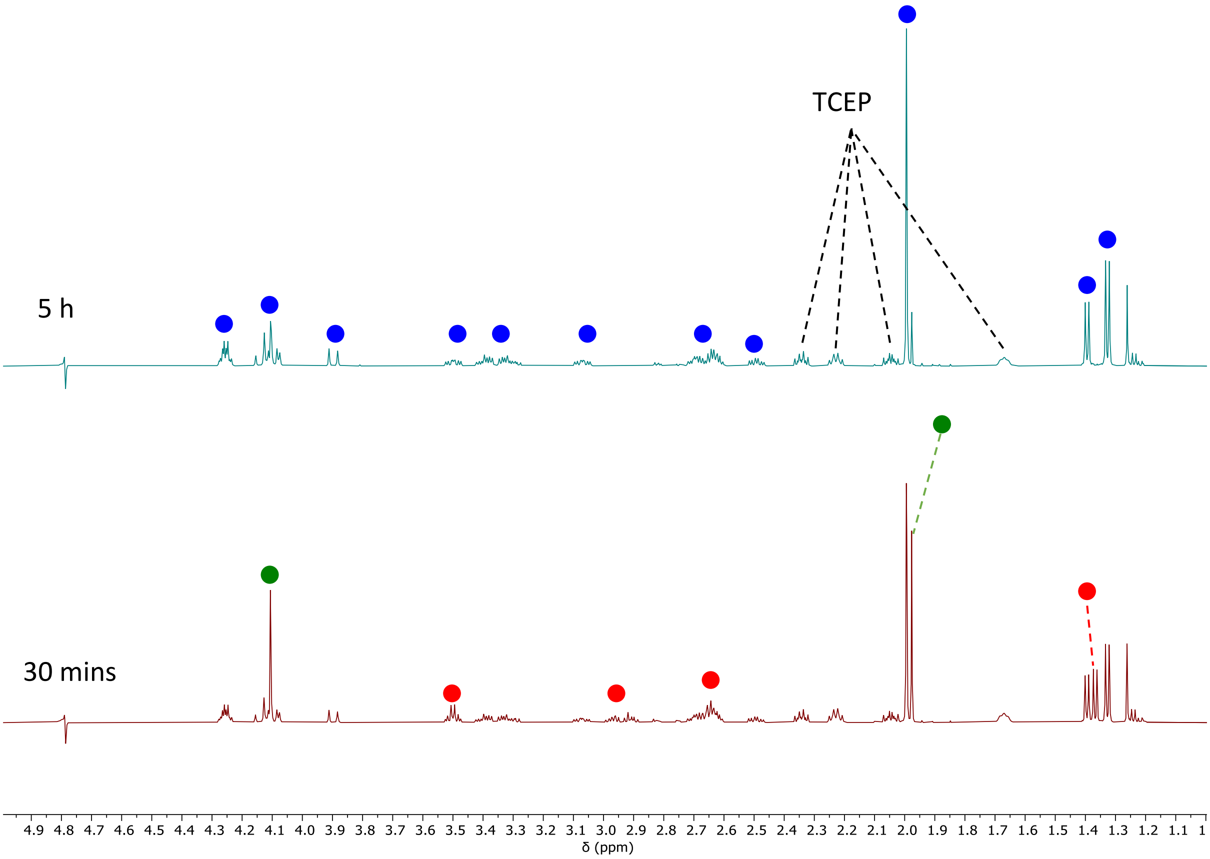

Figure S91: ^{1}H NMR (600 MHz, H_2O/D_2O 9:1, noesygppr1d, 1.0-5.0 ppm) spectra to show the reaction of **16**$_{\mathbf{A}}$ (43 mM) and Ac-Gly-CN (**2**$_{\mathbf{G}}$, 50 mM) in borate buffer (pH 9, 500 mM) at room temperature, which yields **10b**.

Ac-Gly-CN (2_G) Ligation with 16_V to yield **10c**:

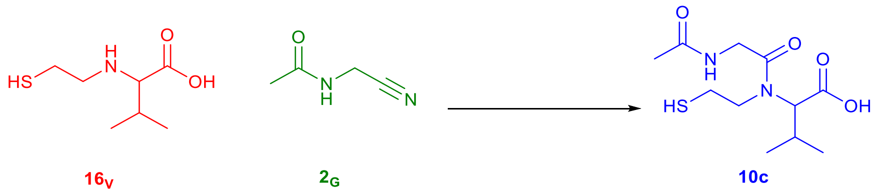

A solution of Ac-Gly-CN 2_G (50 mM), 16_V (37.5 mM), TCEP (50 mM) and KHPH (12.5 mM; internal standard) in phosphate buffer (1 mL, pH 7, 500 mM in H_2O/D_2O (9:1)) was incubated for 24 hours at room temperature. The reaction was monitored by regular acquisition of NMR spectra. After 24 hours, **10c** (75%) was observed.

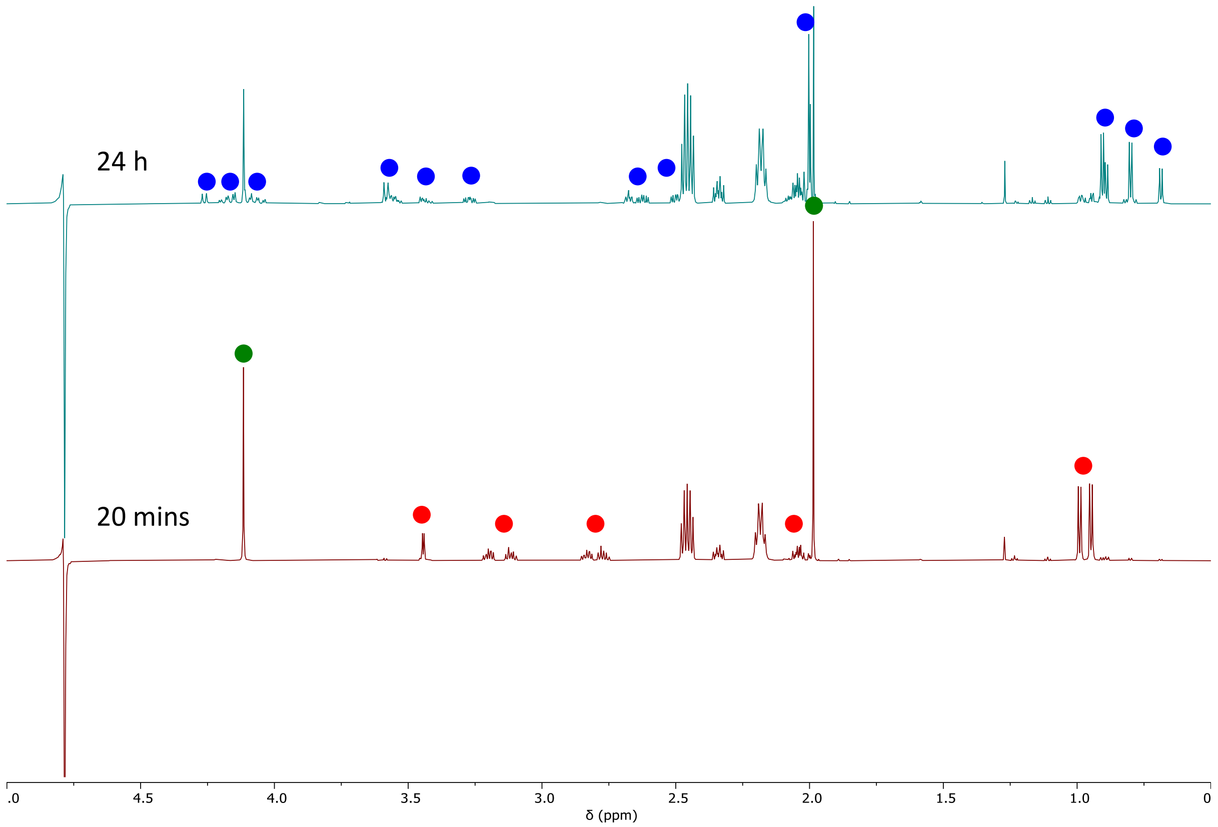

Figure S92: ^{1}H NMR (700 MHz, H_2O/D_2O 9:1, noesygppr1d, 0.0-5.0 ppm) spectra to show the reaction of 16_V (37.5 mM), Ac-Gly-CN (2_G, 50 mM) and TCEP (50 mM) in phosphate buffer (pH 7, 500 mM) at room temperature, which yields **10c**.

A solution of **16**$_{\mathbf{V}}$ (37.5 mM), Ac-Gly-CN **2**$_{\mathbf{G}}$ (50 mM), TCEP (50 mM) and KHPH (12.5 mM; internal standard) in borate buffer (1 mL, pH 9, 500 mM in H_2O/D_2O (9:1)) was incubated for 5 hours at room temperature. The reaction was then analysed by NMR spectroscopy and observed to yield **10c** (>95%) in near quantitative yield.

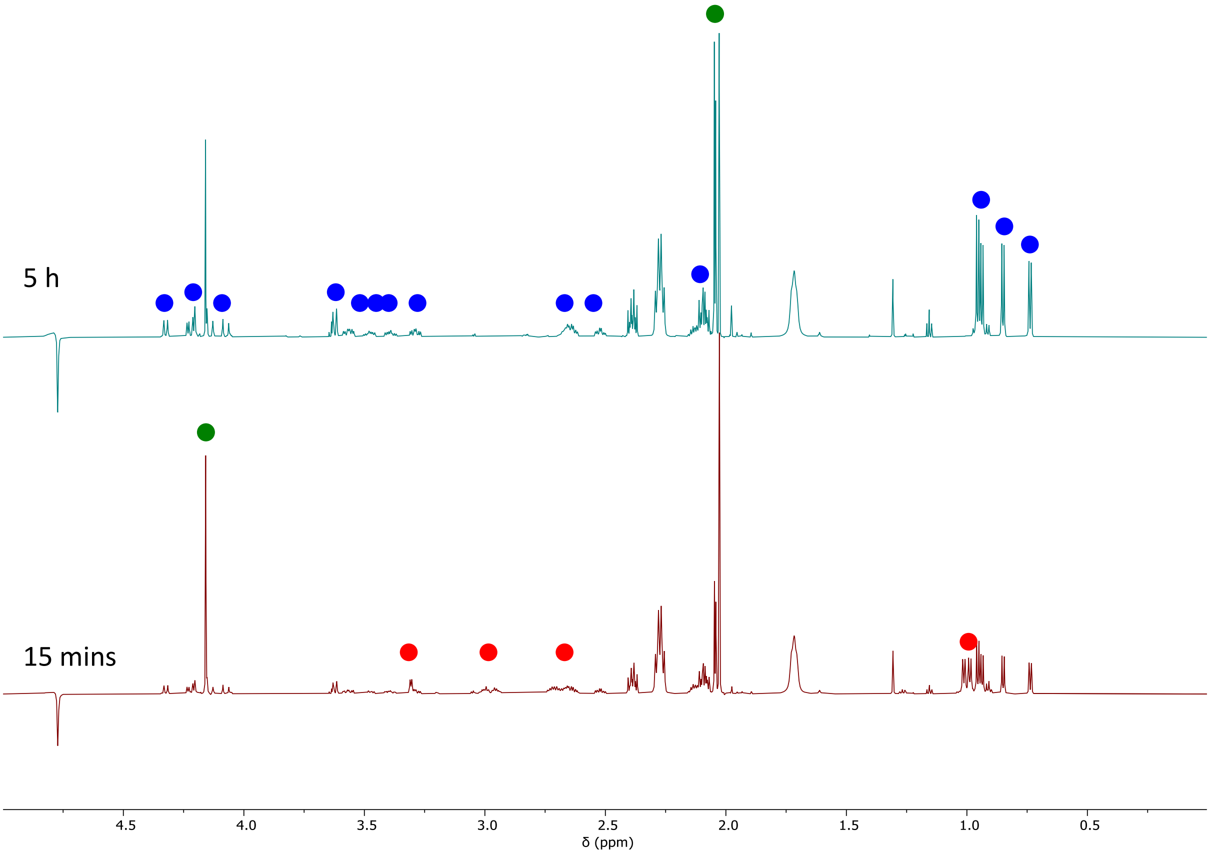

Figure S93: ^{1}H NMR (700 MHz, H_2O/D_2O 9:1, noesygppr1d, 0.0-5.0 ppm) spectra to show the reaction of **16**$_{\mathbf{V}}$ (37.5 mM), Ac-Gly-CN (**2**$_{\mathbf{G}}$, 50 mM) and TCEP (50 mM) in borate buffer (pH 9, 500 mM) at room temperature, which yields **10c**.

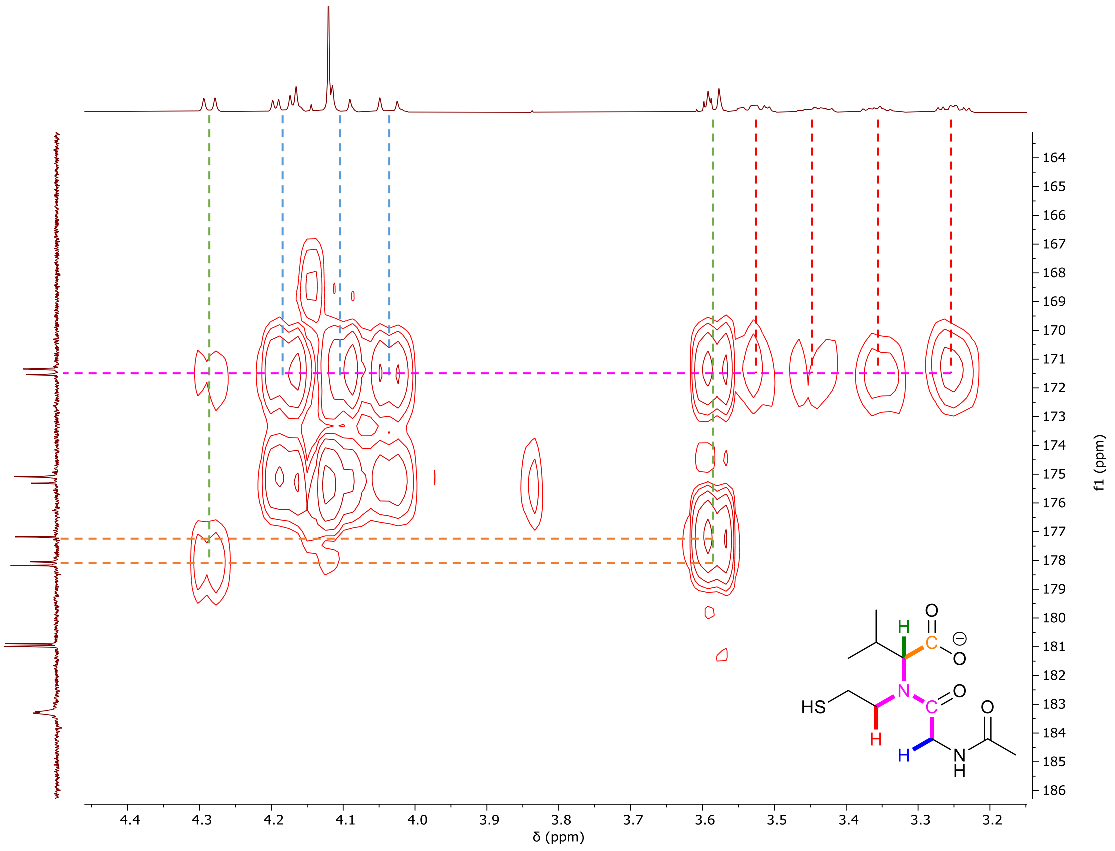

Figure S94: ^1H– ^{13}C HMBC (^1H: 700 MHz [3.0-4.6 ppm], ^{13}C: 175 MHz [167-188 ppm]) spectrum to show the diagnostic $^3J_{CH}$ and $^2J_{CH}$ coupling of α-Gly, α-Val and *N*-CH_2 to tertiary amide carbon at pH 7, that is characteristic of the dipeptoid **10c**.

$\mathbf{16_G}$ ligation of $\mathbf{2_{(AA)m}}$ to yield **10**:

Ac-Ala-CN ($\mathbf{2_A}$):

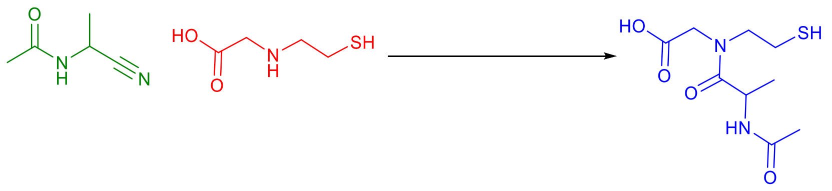

$\mathbf{2_A}$ $\mathbf{16_G}$ **10d**

Following **General procedure D** (pH 7), **10d** was observed in 70% yield.

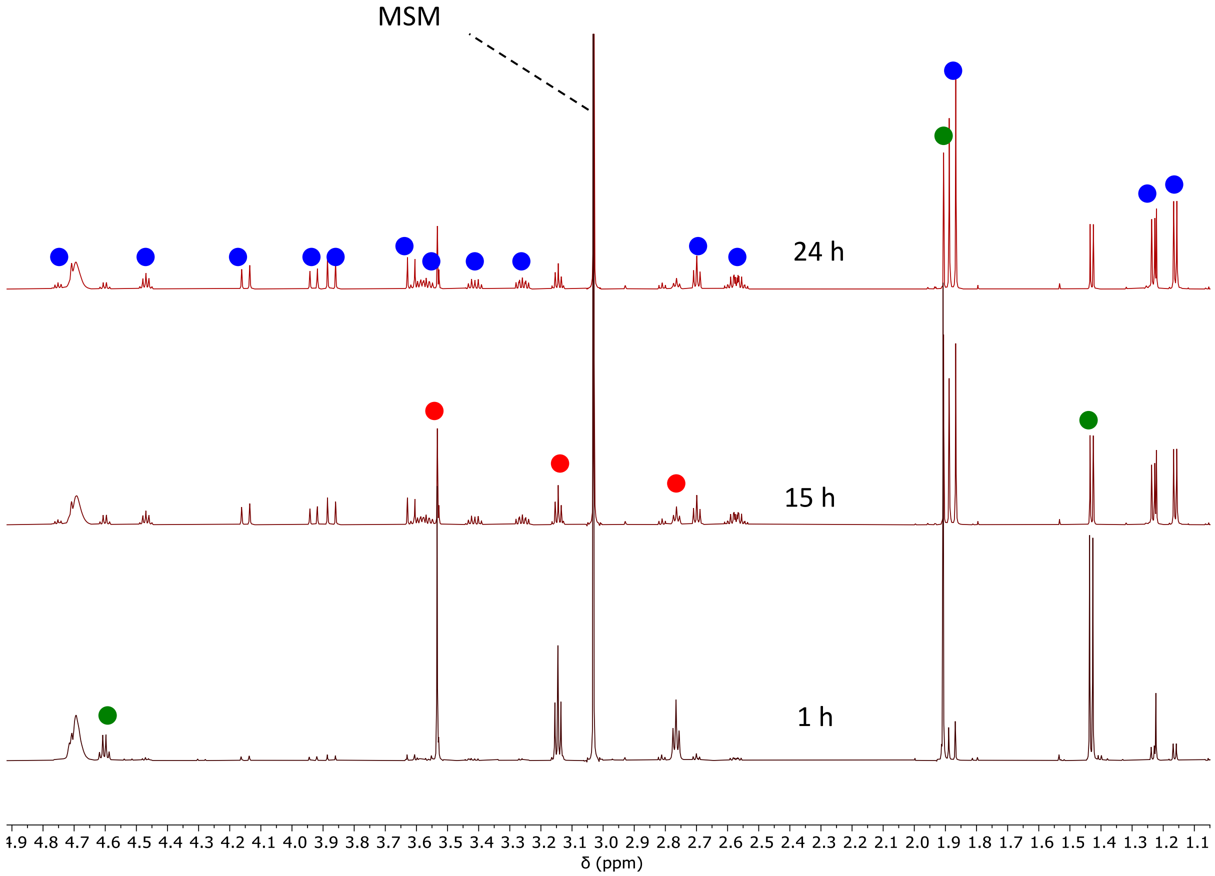

Figure S95: ^{1}H NMR (700 MHz, H_2O/D_2O 9:1, noesygppr1d, 1.1-4.9 ppm) spectra to show the reaction of $\mathbf{16_G}$ (100 mM) and $\mathbf{2_A}$ (100 mM) with MSM (100 mM; internal standard) in phosphate buffer (pH 7, 500 mM) at room temperature, which yields **10d**.

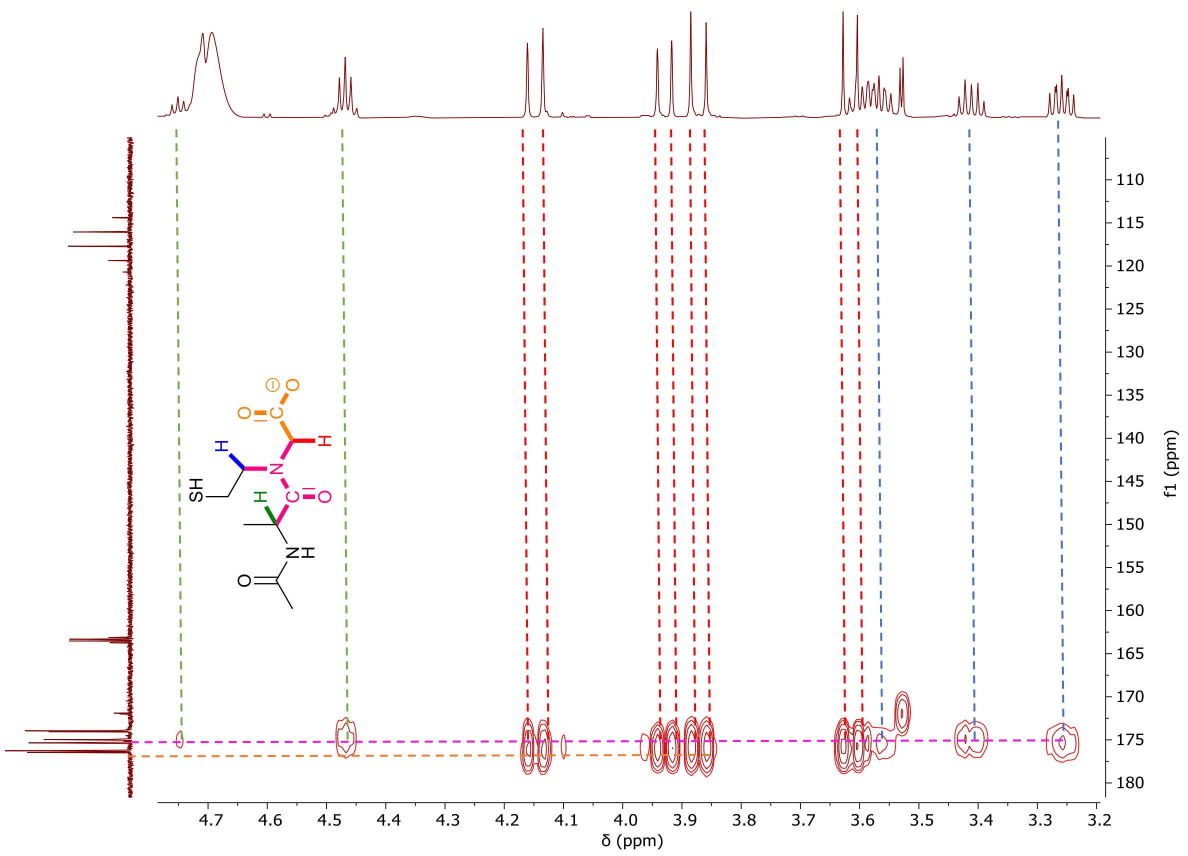

Figure S96: ^{1}H– ^{13}C HMBC (^{1}H: 700 MHz [3.2-4.8 ppm], ^{13}C: 175 MHz [100-180 ppm]) spectrum to show the diagnostic $^{3}J_{CH}$ and $^{2}J_{CH}$ coupling of α-Gly, α-Ala and *N*-CH_2 to tertiary amide carbon at pH 7, that is characteristic of **10d**.

Following **General procedure D** (pH 7), **10d** was observed in 85% yield.

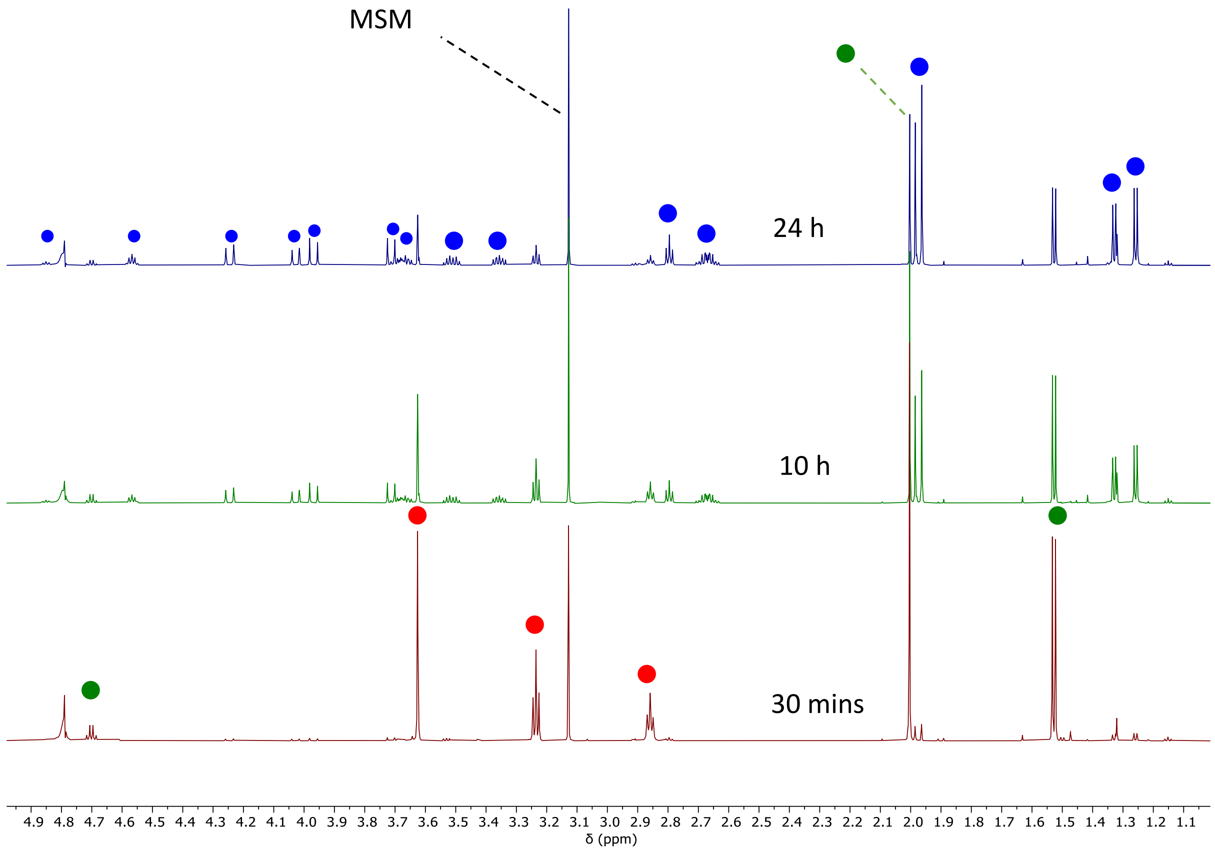

Figure S97: ^{1}H NMR (700 MHz, H_2O/D_2O 9:1, noesygppr1d, 1.1-5.0 ppm) spectra to show the reaction of **16**$_G$ (50 mM) and **2**$_A$ (50 mM) with MSM (12.5 mM; internal standard) in phosphate buffer (pH 7, 500 mM) at room temperature, which yields **10d**.

Following **General procedure D** (pH 9), **10d** was observed in near quantitative yield (>95%) in 10 h.

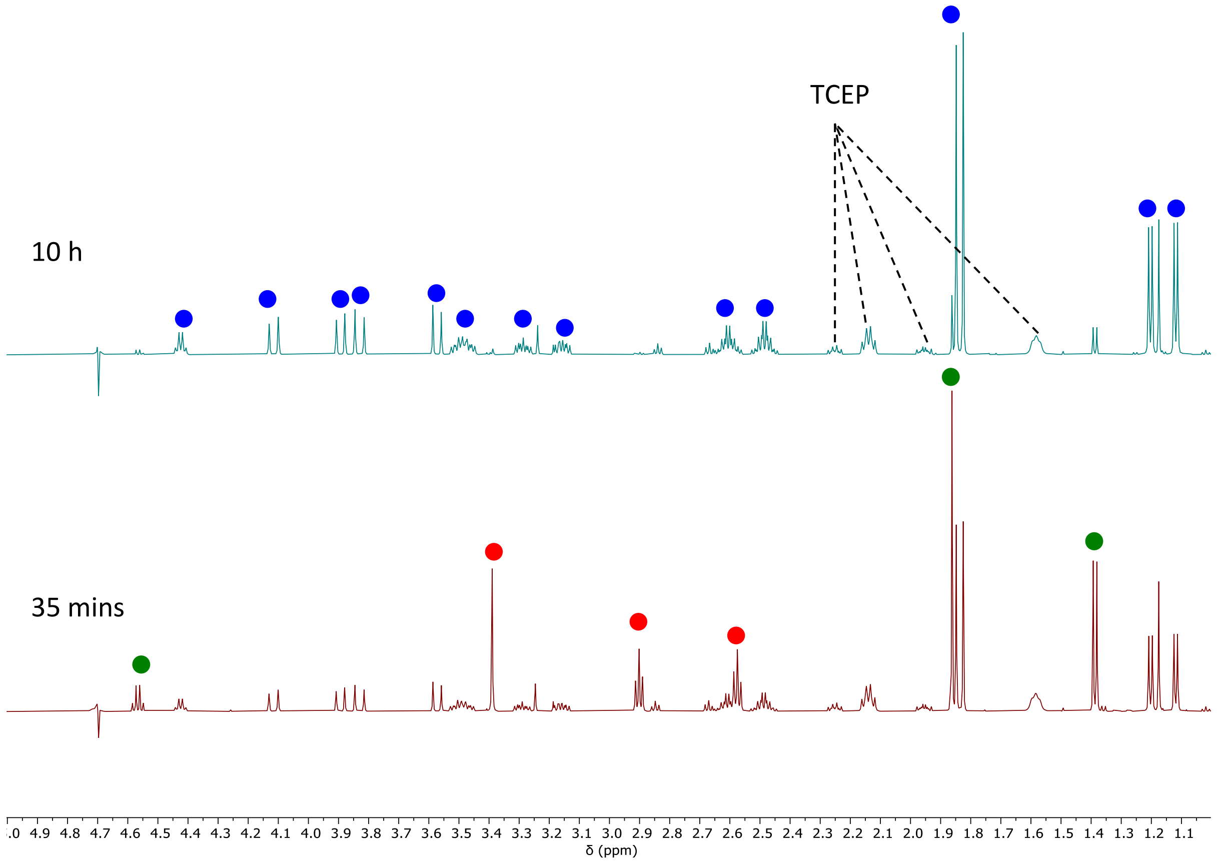

Figure S98: ^{1}H NMR (600 MHz, H_2O/D_2O 9:1, noesygppr1d, 1.0-5.0 ppm) spectra to show the reaction of **16**$_G$ (45 mM), Ac-Ala-CN (**2**$_A$, 50 mM) and TCEP (10 mM) in borate buffer (pH 9, 500 mM) at room temperature, which yields **10d**.

Ac-Met-CN (**2**$_M$):

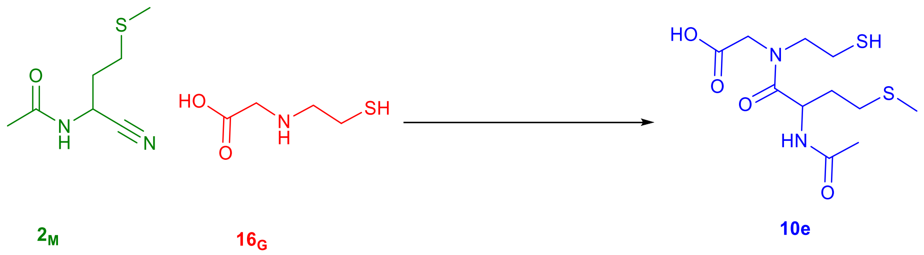

Following **General procedure D** (pH 7), **10e** was observed in 90% yield.

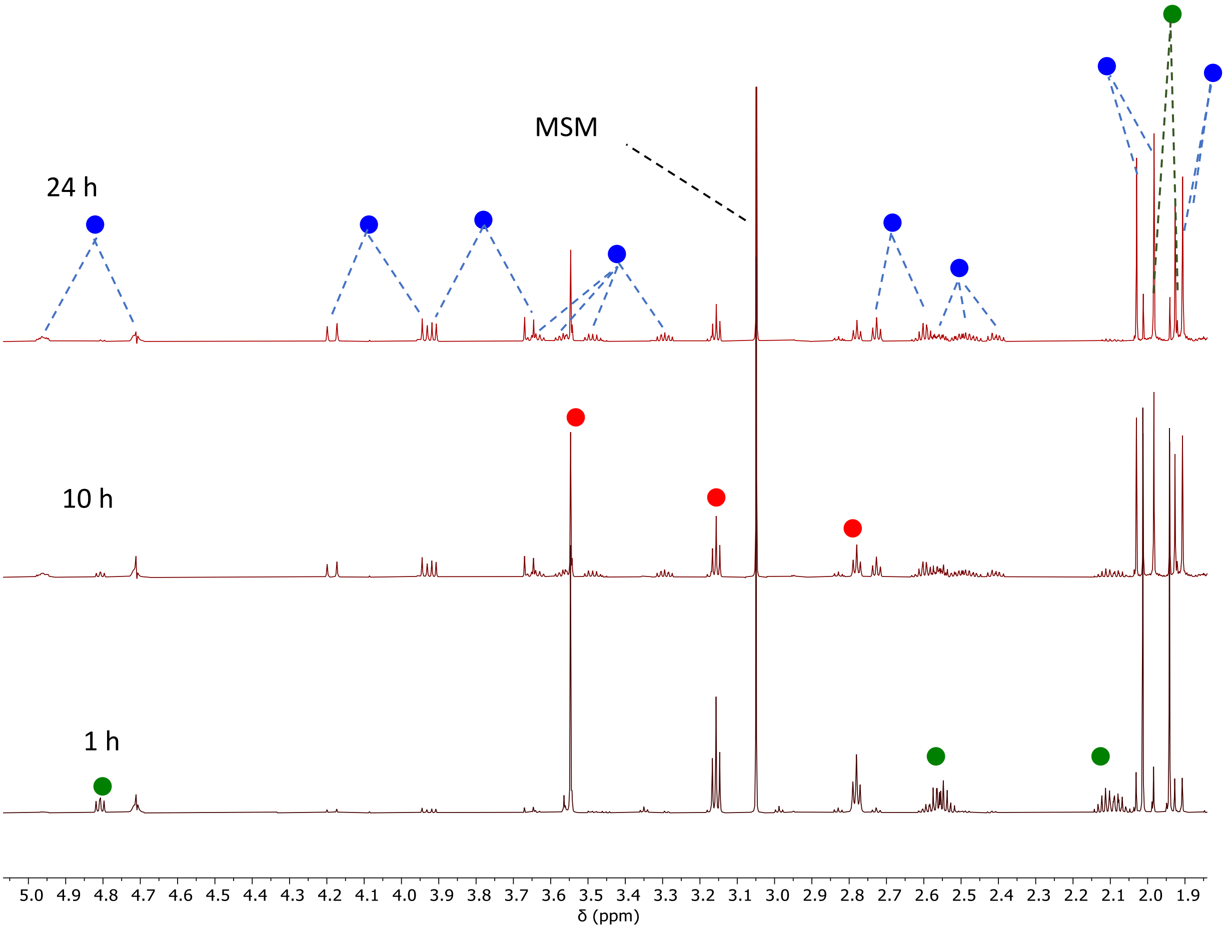

Figure S99: ^{1}H NMR (700 MHz, H_2O/D_2O 9:1, noesygppr1d, 1.9-5.0 ppm) spectra to show the reaction of **16**$_G$ (50 mM) and **2**$_M$ (50 mM) with MSM (25 mM; internal standard) in phosphate buffer (pH 7, 500 mM) at room temperature, which yields **10e**.

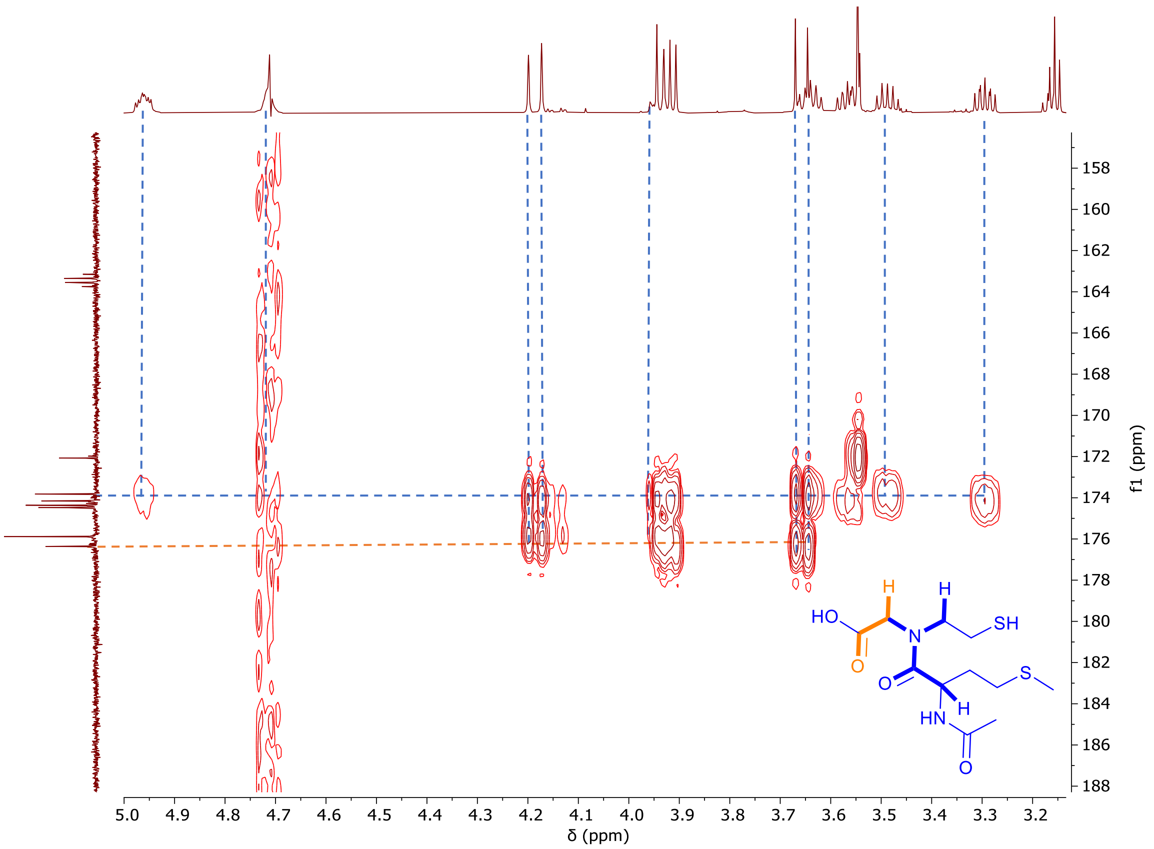

Figure S100: ^{1}H– ^{13}C HMBC (^{1}H: 700 MHz [3.2-5.0 ppm], ^{13}C: 175 MHz [155-190 ppm]) spectrum to show the diagnostic $^{3}J_{CH}$ and $^{2}J_{CH}$ coupling of α-Gly, α-Met and *N*-CH_2 to tertiary amide carbon at pH 7, that is characteristic of **10e**.

Following **General procedure D** (pH 9), **10e** was observed in near quantitative yield (>95%) in 10 h.

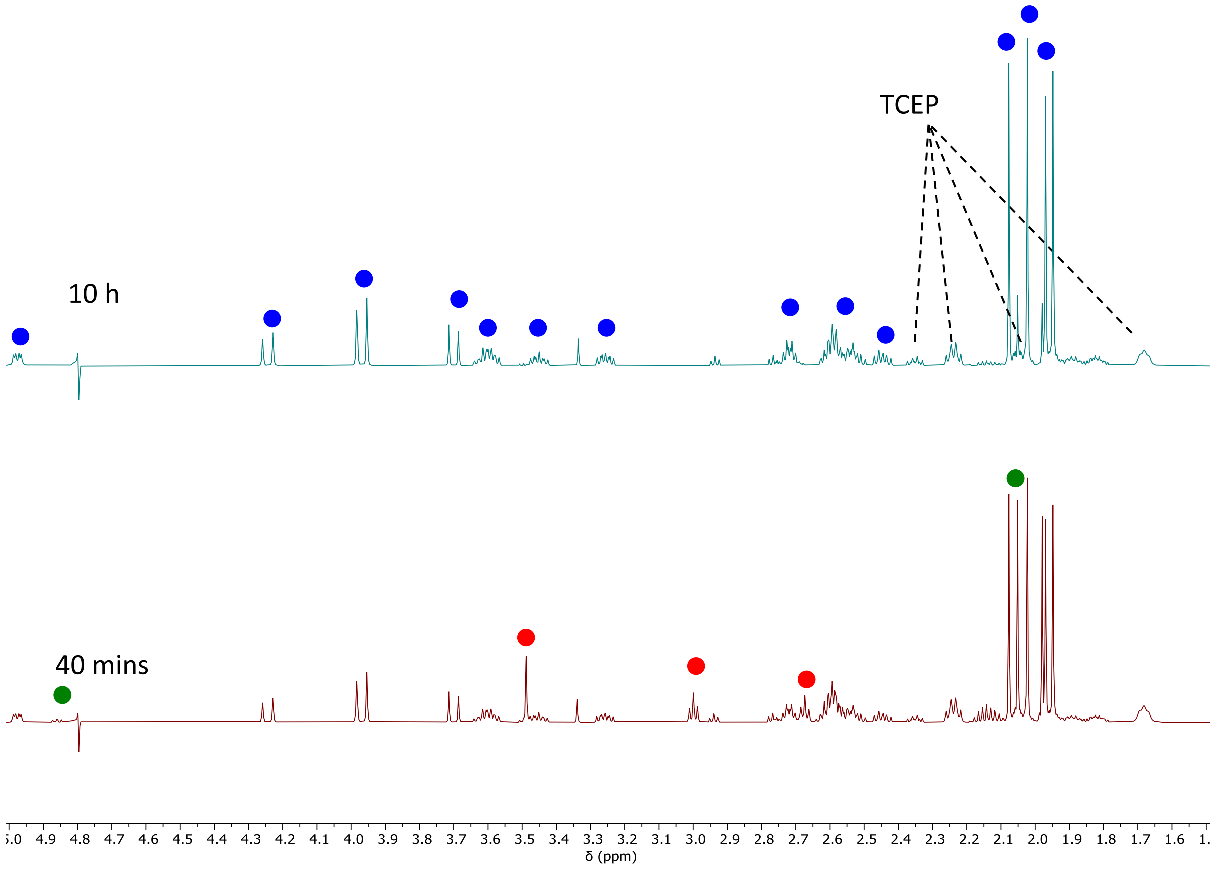

Figure S101: ^{1}H NMR (600 MHz, H_2O/D_2O 9:1, noesygppr1d, 1.5-5.0 ppm) spectra to show the reaction of **16$_G$** (45 mM), Ac-Met-CN (**2$_M$**, 50 mM) and TCEP (10 mM) in borate buffer (pH 9, 500 mM) at room temperature, which yields **10e**.

Ac-Pro-CN ($\mathbf{2_P}$):

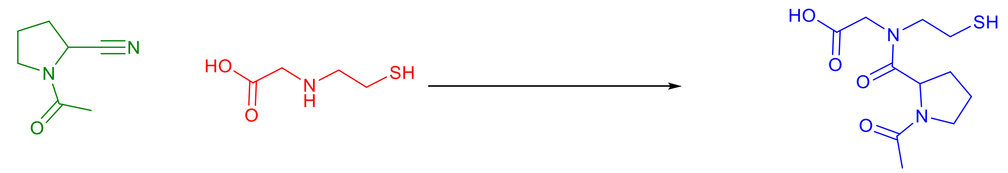

$\mathbf{2_P}$ $\mathbf{16_G}$ **10f**

Following **General procedure D** (pH 7), **10f** was observed in 90% yield.

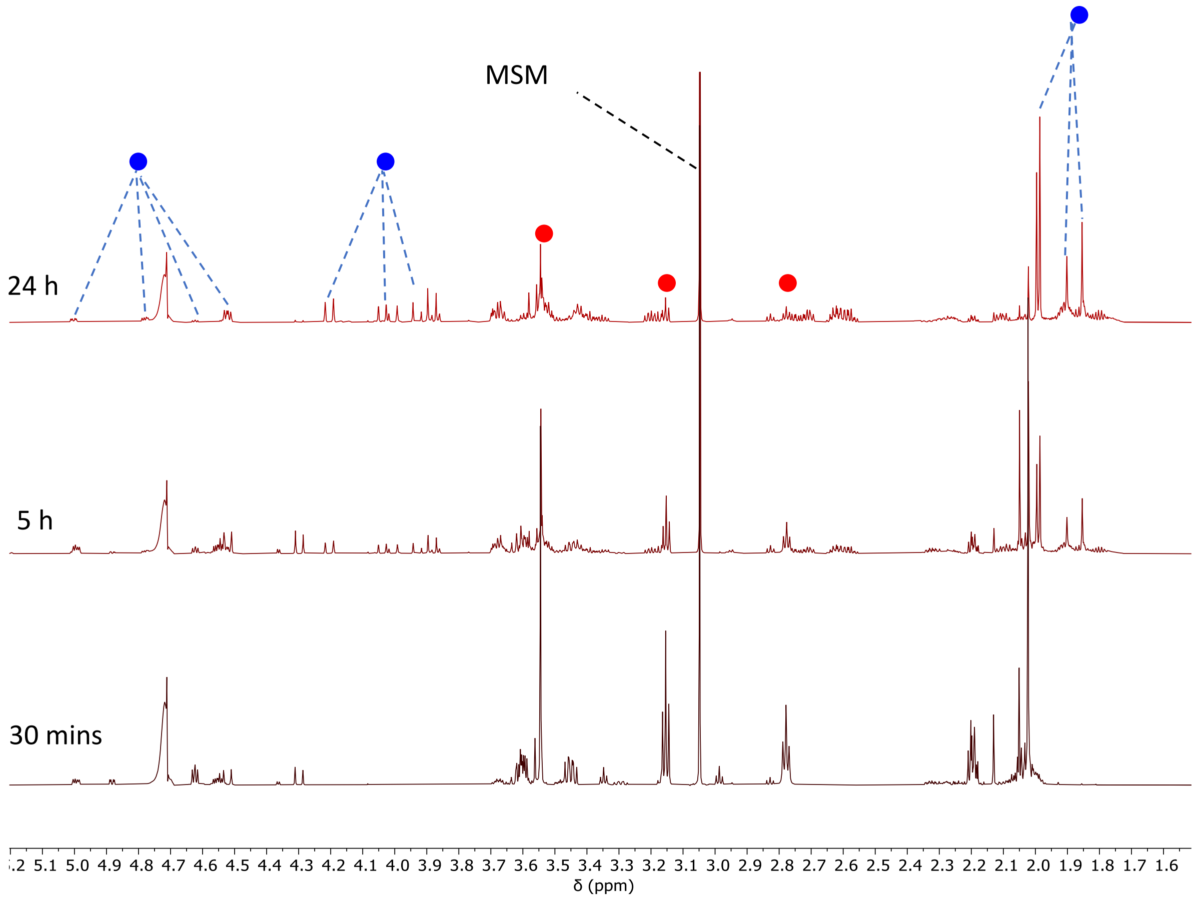

Figure S102: ^{1}H NMR (700 MHz, H_2O/D_2O 9:1, noesygppr1d, 1.6-5.2 ppm) spectra to show the reaction of $\mathbf{16_G}$ (50 mM) and Ac-Pro-CN ($\mathbf{2_P}$, 50 mM) with MSM (12.5 mM; internal standard) in phosphate buffer (pH 7, 500 mM) at room temperature, which yields **10f**.

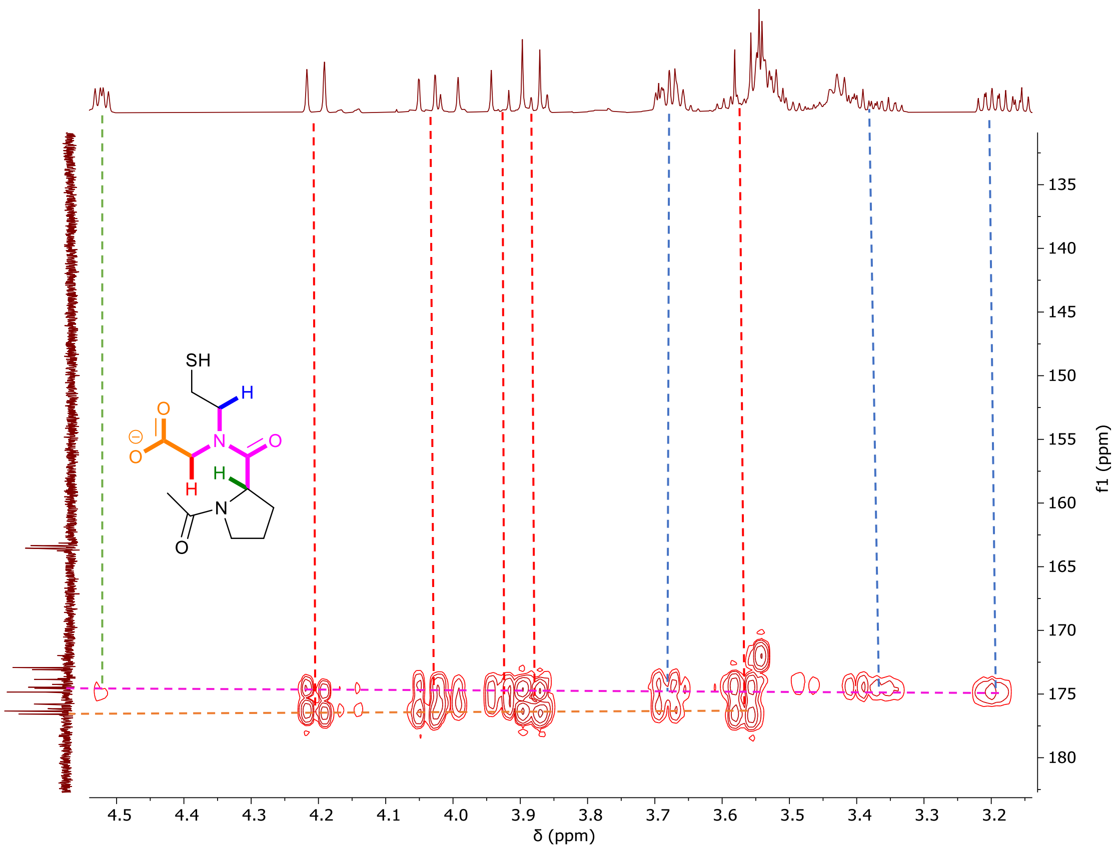

Figure S103: ^{1}H– ^{13}C HMBC (^{1}H: 700 MHz [3.2-4.6 ppm], ^{13}C: 175 MHz [130-180 ppm]) spectrum to show the diagnostic $^{3}J_{CH}$ and $^{2}J_{CH}$ coupling of α-Gly, α-Pro and *N*-CH_2 to tertiary amide carbon at pH 7, that is characteristic of **10f**.

Following **General procedure D** (pH 9), **10f** was observed in near quantitative yield (>95%) in 15 h.

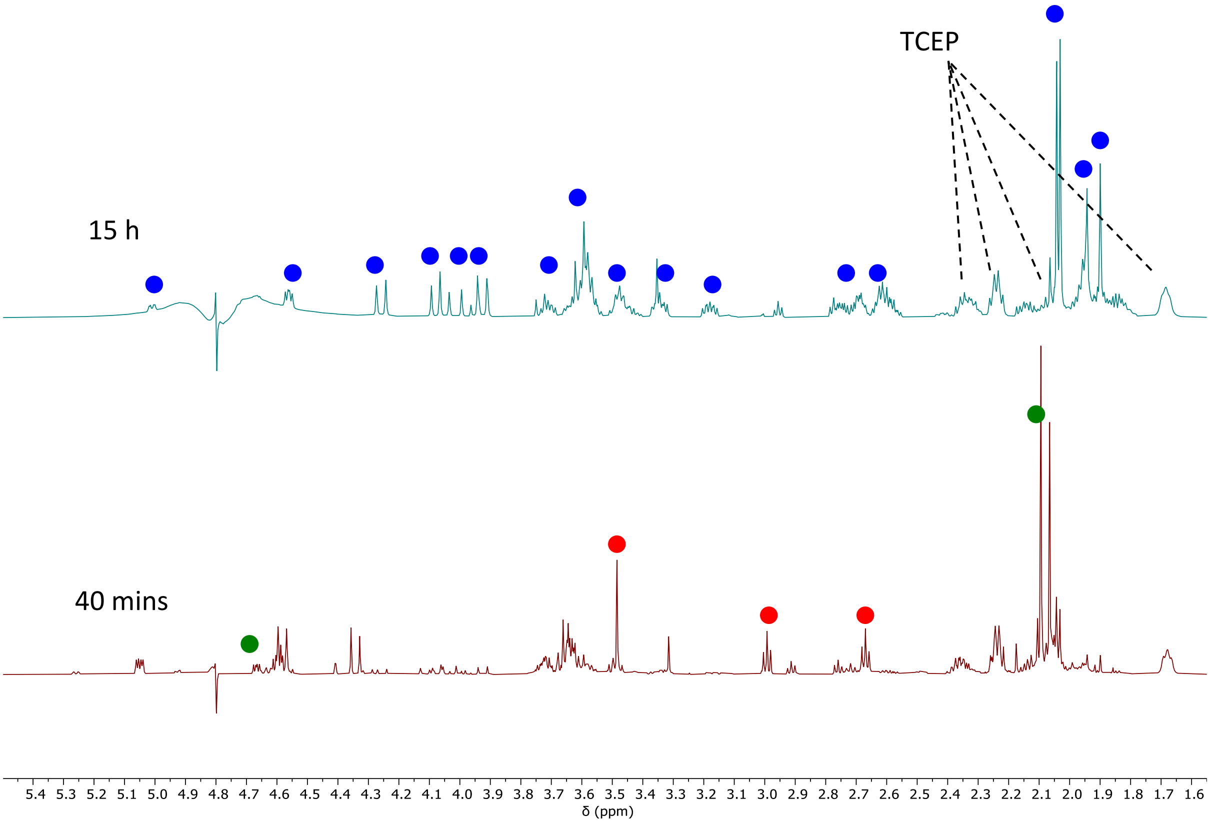

Figure S104: ^{1}H NMR (600 MHz, H_2O/D_2O 9:1, noesygppr1d, 1.5-5.5 ppm) spectra to show the reaction of **16$_G$** (45 mM), Ac-Pro-CN (**2$_P$**, 50 mM) and TCEP (10 mM) in borate buffer (pH 9, 500 mM) at room temperature, which yields **10f**.

Ac-Ser-CN (**2**$_S$):

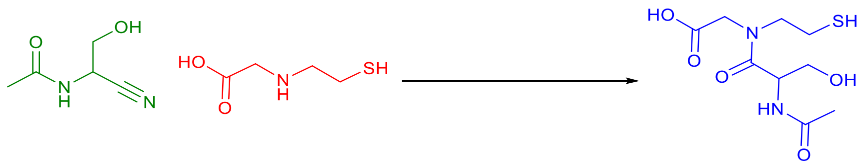

2$_S$ **17**$_G$ **8g**

Following **General procedure D** (pH 7), **10g** was observed in 89% yield.

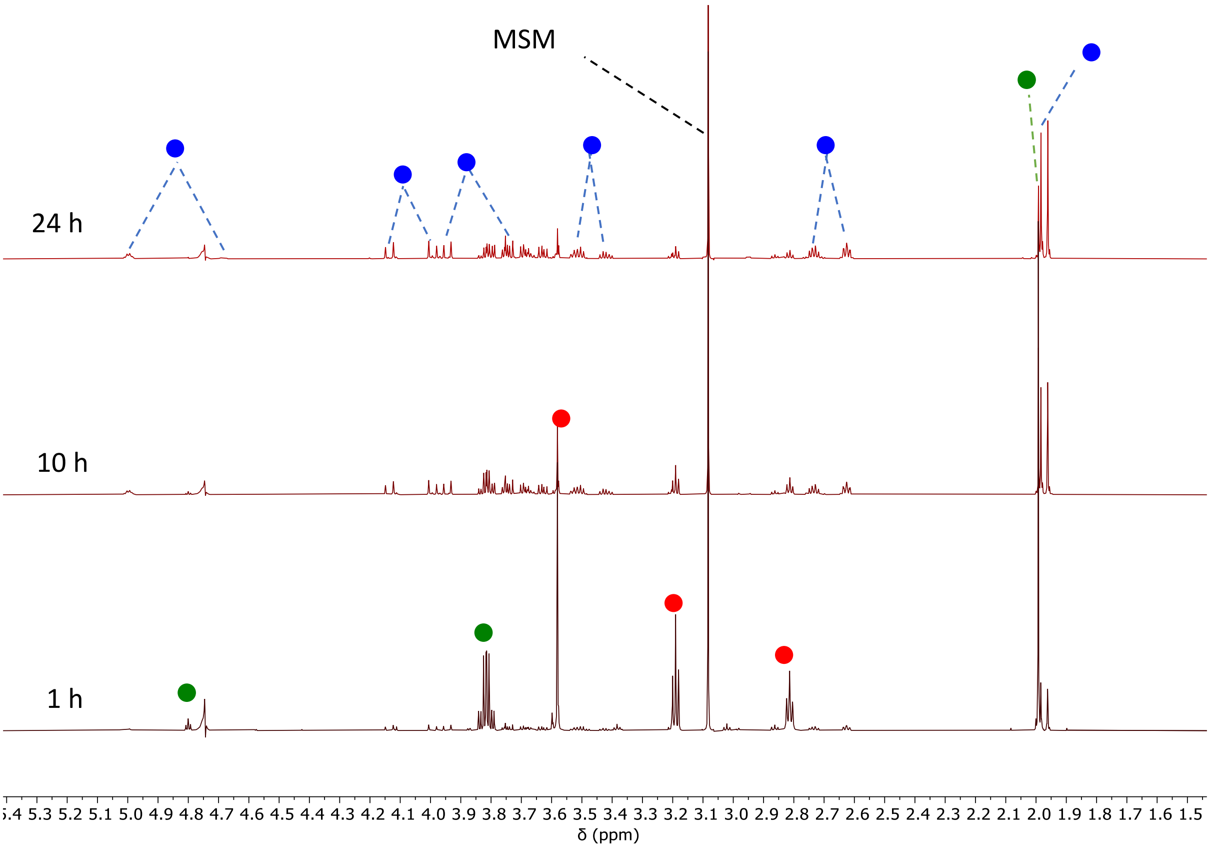

Figure S105: ^{1}H NMR (700 MHz, H_2O/D_2O 9:1, noesygppr1d, 1.5-5.4 ppm) spectra to show the reaction of **16**$_G$ (50 mM) and Ac-Ser-CN (**2**$_S$,50 mM) with MSM (25 mM, internal standard) in phosphate buffer (pH 7, 500 mM) at room temperature, which yields **10g**.

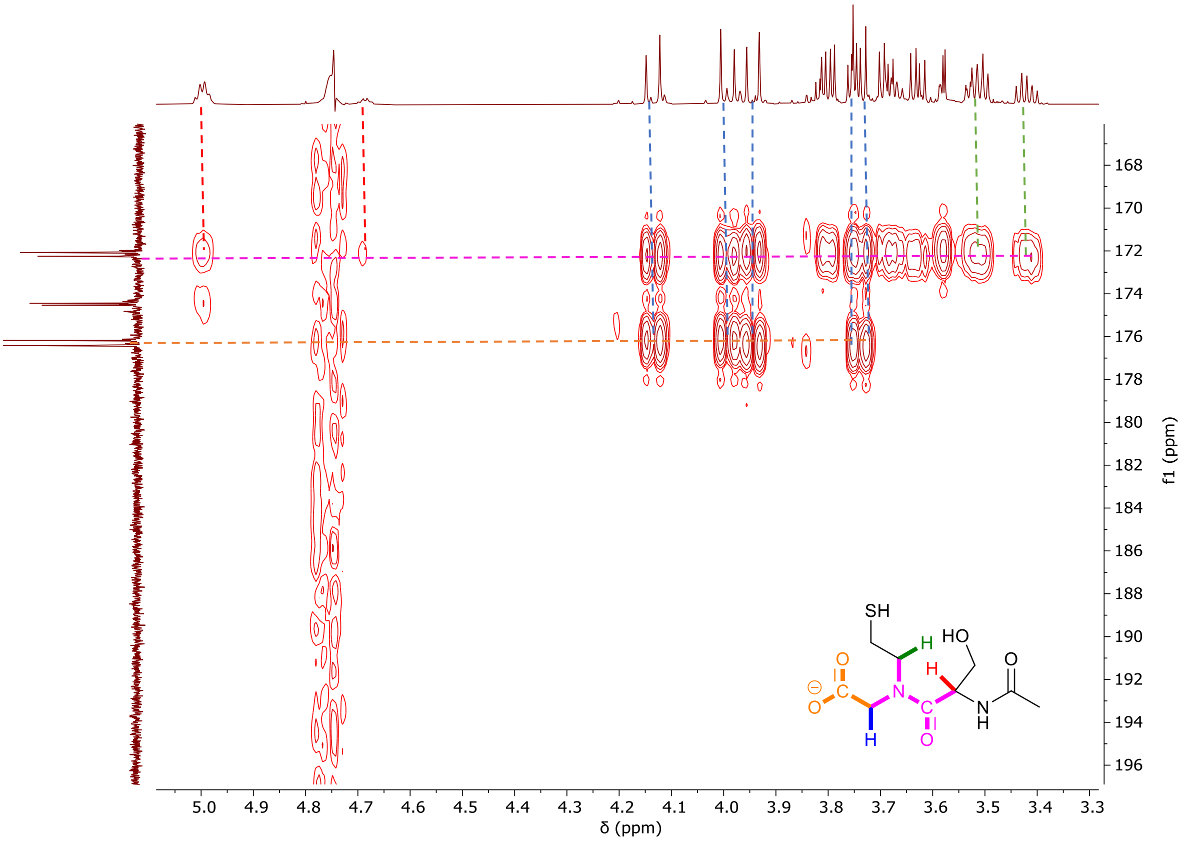

Figure S106: ^{1}H– ^{13}C HMBC (^{1}H: 700 MHz [3.3-5.1 ppm], ^{13}C: 175 MHz [165-195 ppm]) spectrum to show the diagnostic $^3J_{CH}$ and $^2J_{CH}$ coupling of α-Gly, α-Ser and *N*-CH_2 to tertiary amide carbon at pH 7, that is characteristic of **10g**.

Following **General procedure D** (pH 9), **10g** was observed in near quantitative yield (>95%) in 10 h.

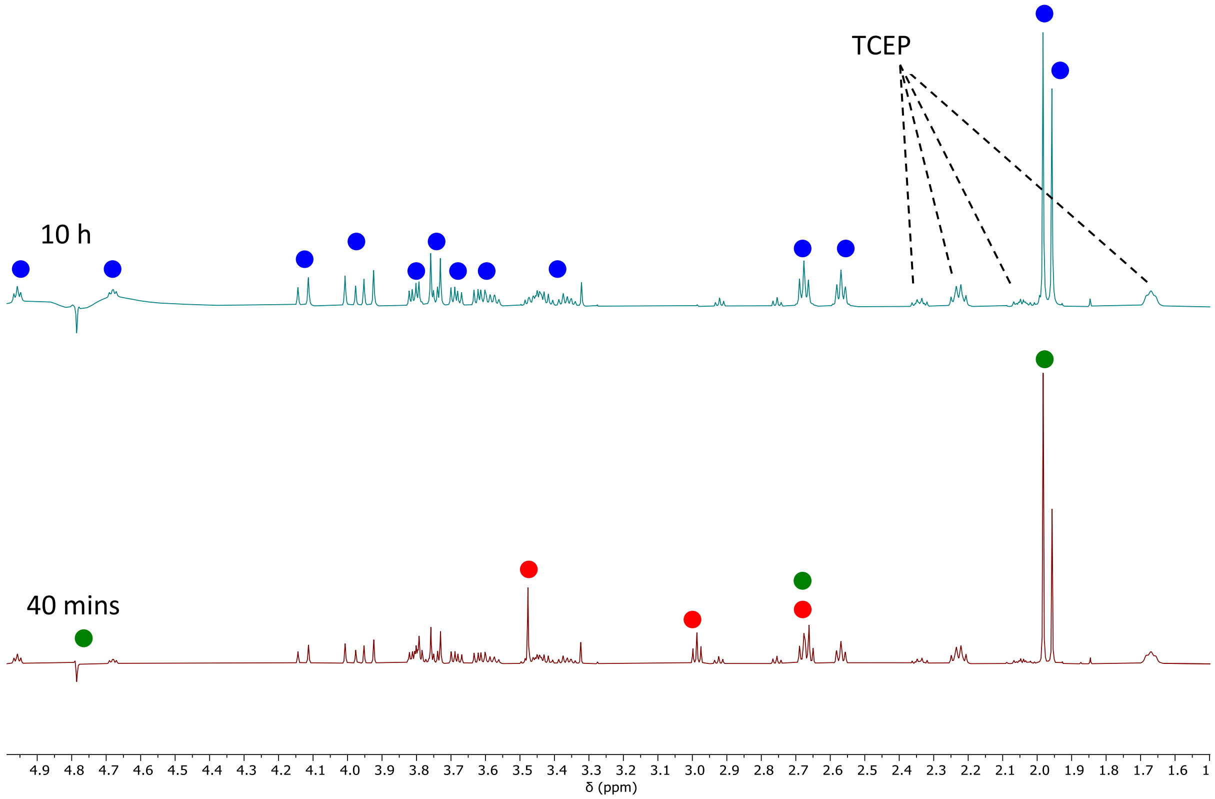

Figure S107: ^{1}H NMR (600 MHz, H_2O/D_2O 9:1, noesygppr1d, 1.5-5.5 ppm) spectra to show the reaction of **16**$_G$ (45 mM), Ac-Ser-CN (**2**$_S$, 50 mM) and TCEP (10 mM) in borate buffer (pH 9, 500 mM) at room temperature, which yields **10g**.

Ac-Leu-CN (**2$_L$**):

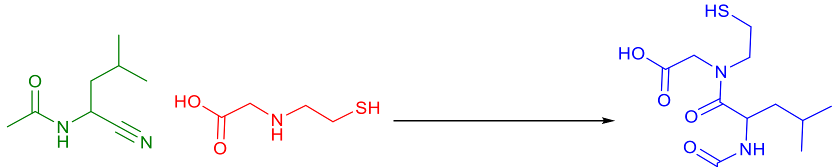

2$_L$ **16$_G$** **10h**

Following **General procedure D** (pH 7), **10h** was observed in 80% yield.

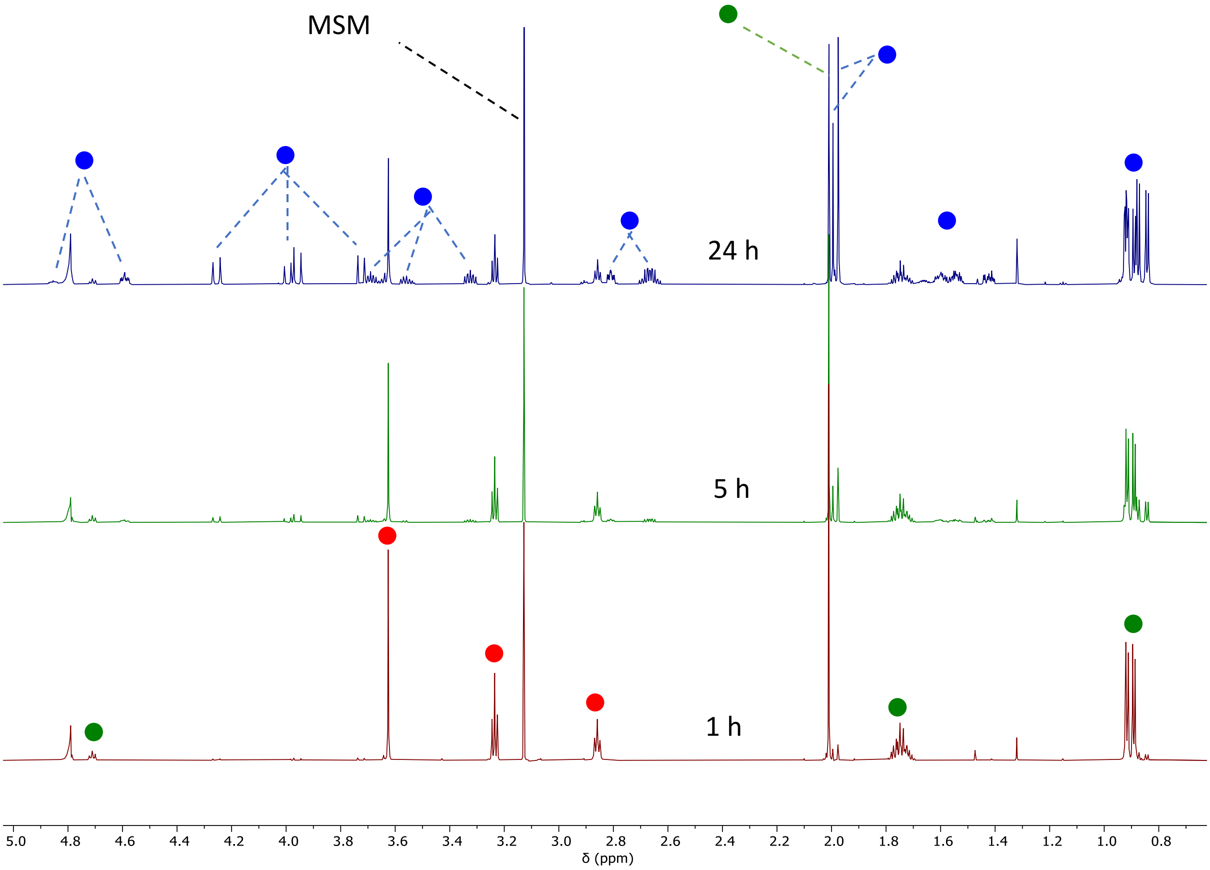

Figure S108: ^{1}H NMR (700 MHz, H_2O/D_2O 9:1, noesygppr1d, 0.6-5.0 ppm) spectra to show the reaction of **16$_G$** (50 mM) and Ac-Leu-CN (**2$_L$**,50 mM) with MSM (12.5 mM, internal standard) in phosphate buffer (pH 7, 500 mM) at room temperature, which yields **10h**.

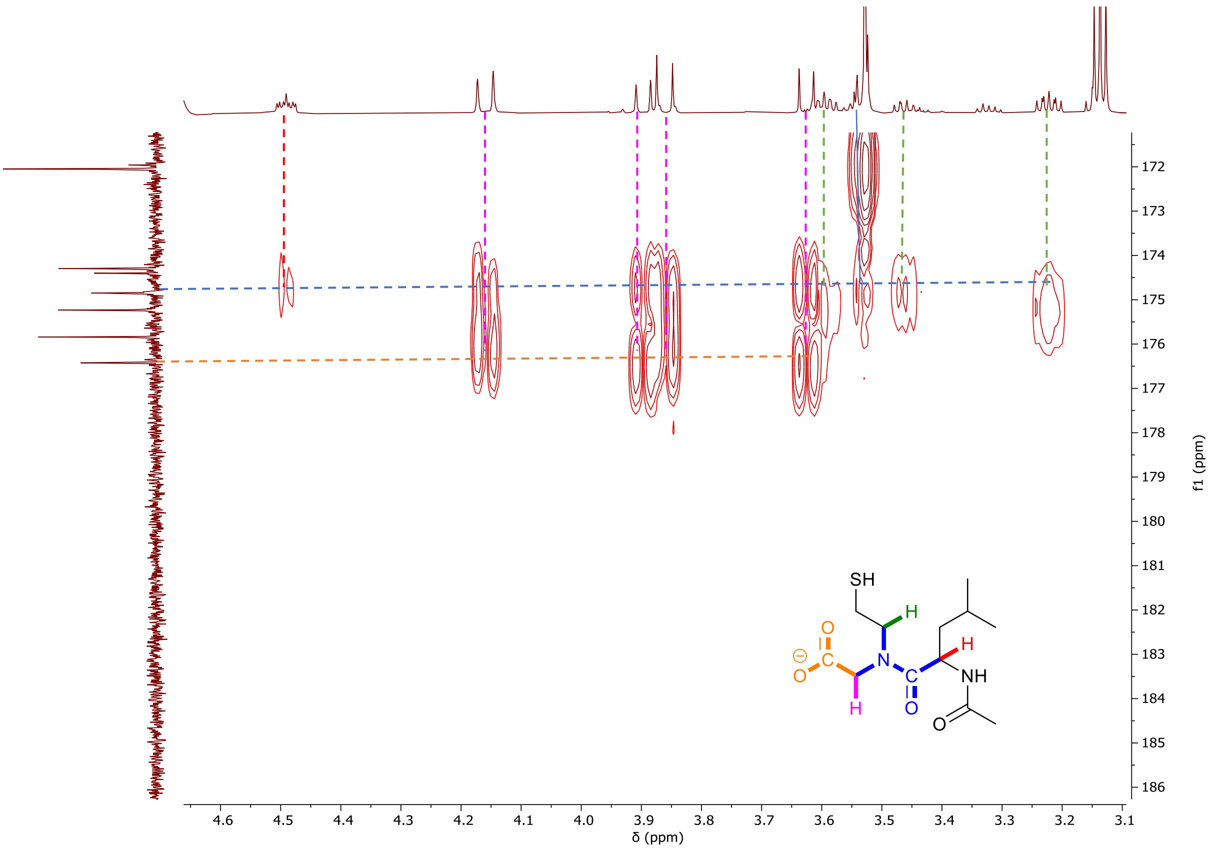

Figure S109: ^{1}H– ^{13}C HMBC (^{1}H: 700 MHz [3.1-4.6 ppm], ^{13}C: 175 MHz [170-185 ppm]) spectrum to show the diagnostic $^3J_{CH}$ and $^2J_{CH}$ coupling of α-Gly, α-Leu and *N*-CH$_2$ to tertiary amide carbon at pH 7, that is characteristic of **10h**.

Following **General procedure D** (pH 9), **10h** was observed in near quantitative yield (>95%) in 24 h.

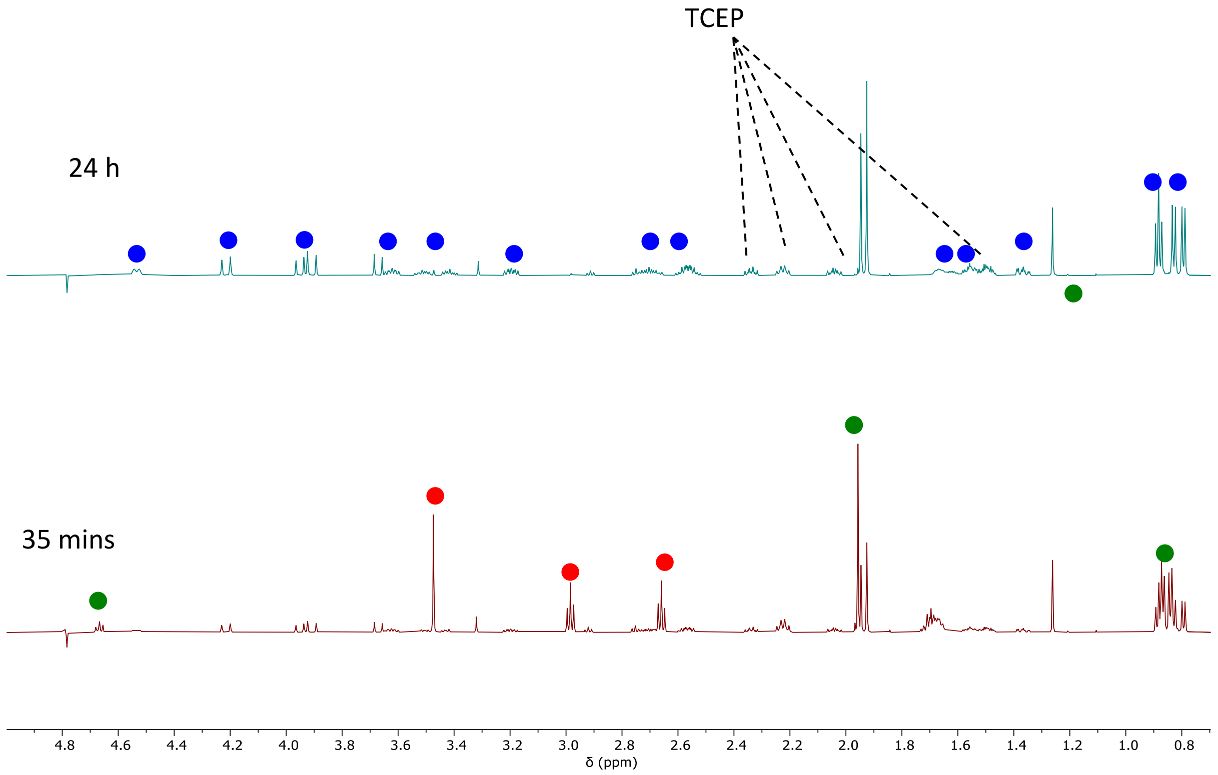

Figure S110: ^{1}H NMR (600 MHz, H_2O/D_2O 9:1, noesygppr1d, 0.7-5.0 ppm) spectra to show the reaction of $\mathbf{16_G}$ (45 mM), Ac-Leu-CN ($\mathbf{2_L}$, 45 mM) and TCEP (10 mM) in borate buffer (pH 9, 500 mM) at room temperature, which yields **10h**.

Ac-Arg-CN ($\mathbf{2_R}$):

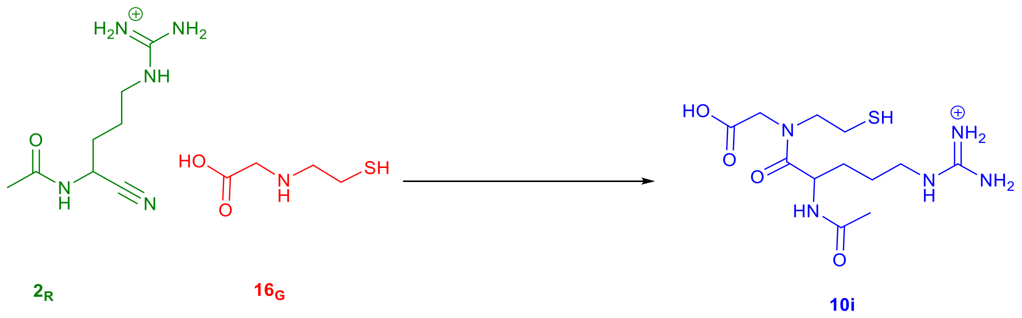

Following **General procedure D** (pH 7), **10i** was observed in 85% yield.

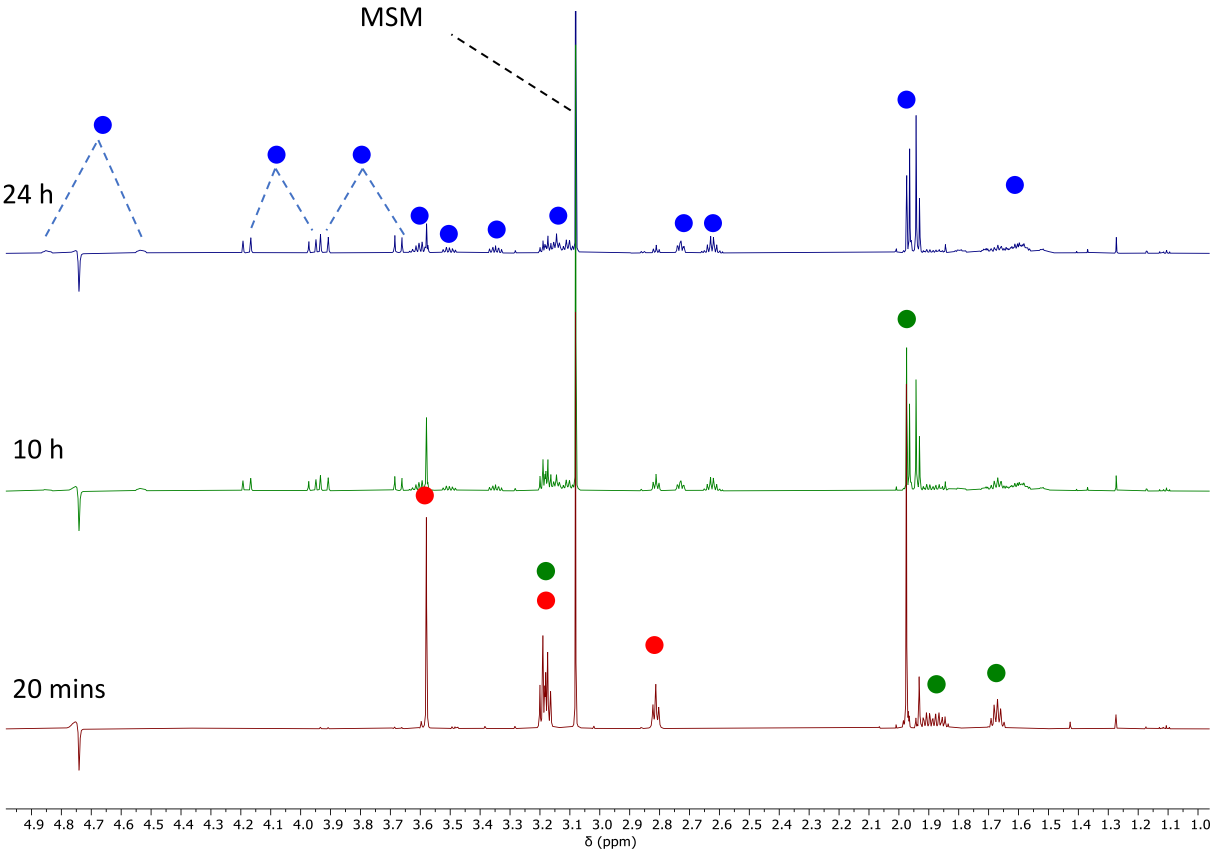

Figure S111: ^{1}H NMR (700 MHz, H_2O/D_2O 9:1, noesygppr1d, 1.0-5.0 ppm) spectra to show the reaction of $\mathbf{16_G}$ (50 mM) and Ac-Arg-CN ($\mathbf{2_R}$, 50 mM) with MSM (25 mM; internal standard) in phosphate buffer (pH 7, 500 mM) at room temperature, which yields **10i**.

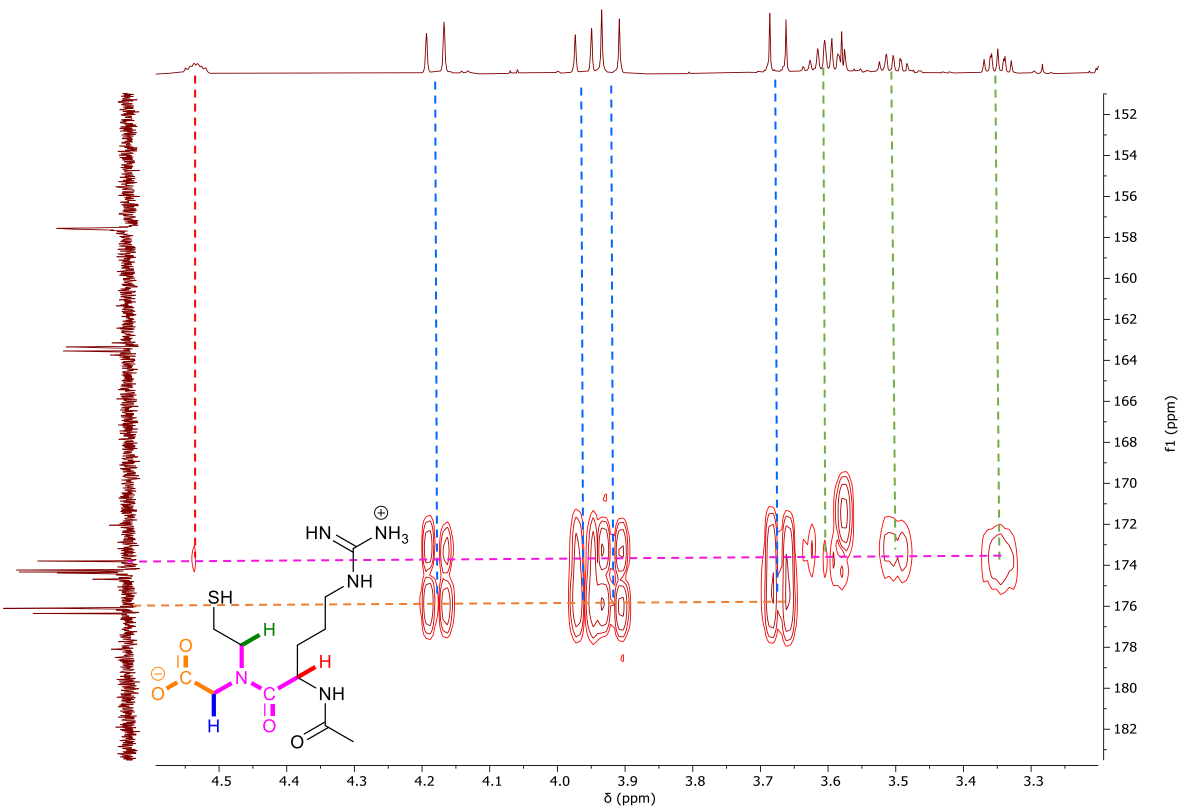

Figure S112: ^{1}H– ^{13}C HMBC (^{1}H: 700 MHz [3.2-4.6 ppm], ^{13}C: 175 MHz [150-185 ppm]) spectrum to show the diagnostic $^{3}J_{CH}$ and $^{2}J_{CH}$ coupling of α-Gly, α-Arg and *N*-CH$_2$ to tertiary amide carbon at pH 7, that is characteristic of **10i**.

Following **General procedure D** (pH 9), **10i** was observed in near quantitative yield (>95%) in 10 h.

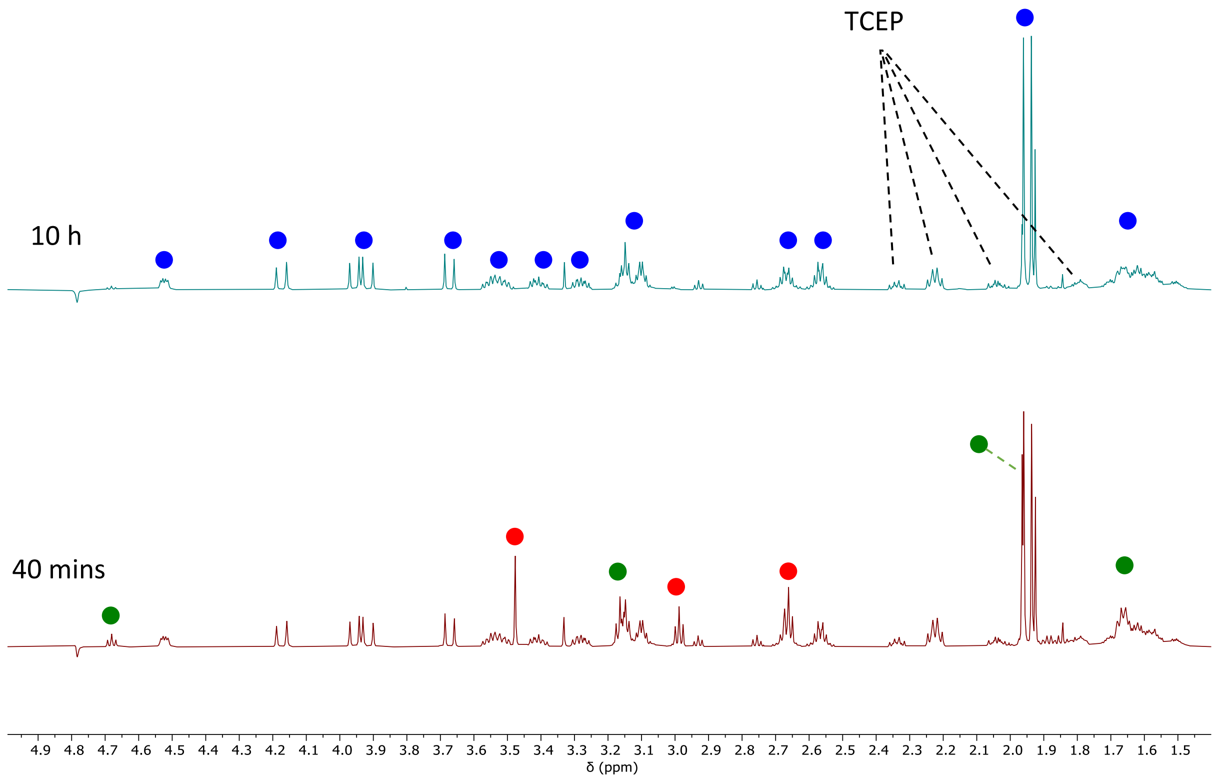

Figure S113: ^{1}H NMR (600 MHz, H_2O/D_2O 9:1, noesygppr1d, 1.4-5.0 ppm) spectra to show the reaction of **16**$_{\mathbf{G}}$ (45 mM), Ac-Arg-CN (**2**$_{\mathbf{R}}$, 50 mM) and TCEP (10 mM) in borate buffer (pH 9, 500 mM) at room temperature, which yields **10i**.

Ac-Asp-CN (**2**$_D$):

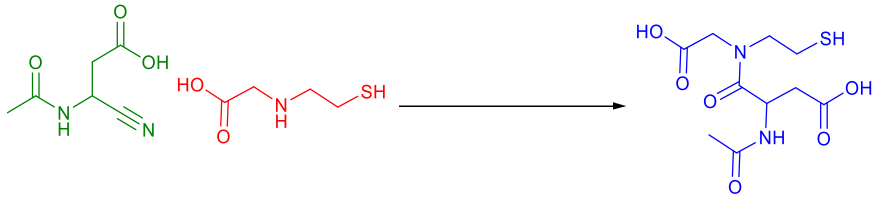

Following **General procedure D** (pH 7), **10j** was observed in 75% yield.

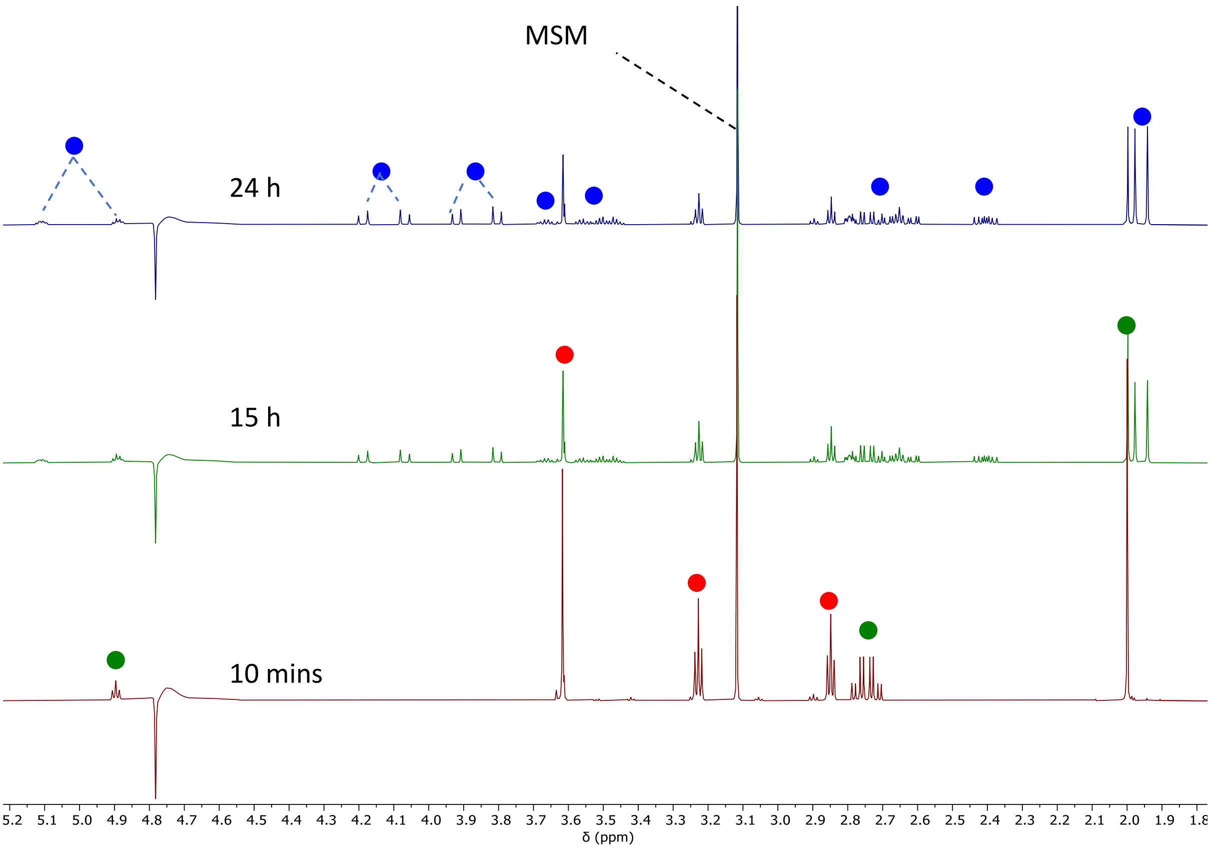

Figure S114: ^{1}H NMR (700 MHz, H_2O/D_2O 9:1, noesygppr1d, 1.8-5.2 ppm) spectra to show the reaction of **16**$_G$ (50 mM) and Ac-Asp-CN (**2**$_D$, 50 mM) with MSM (25 mM; internal standard) in phosphate buffer (pH 7, 500 mM) at room temperature, which yields **10j**.

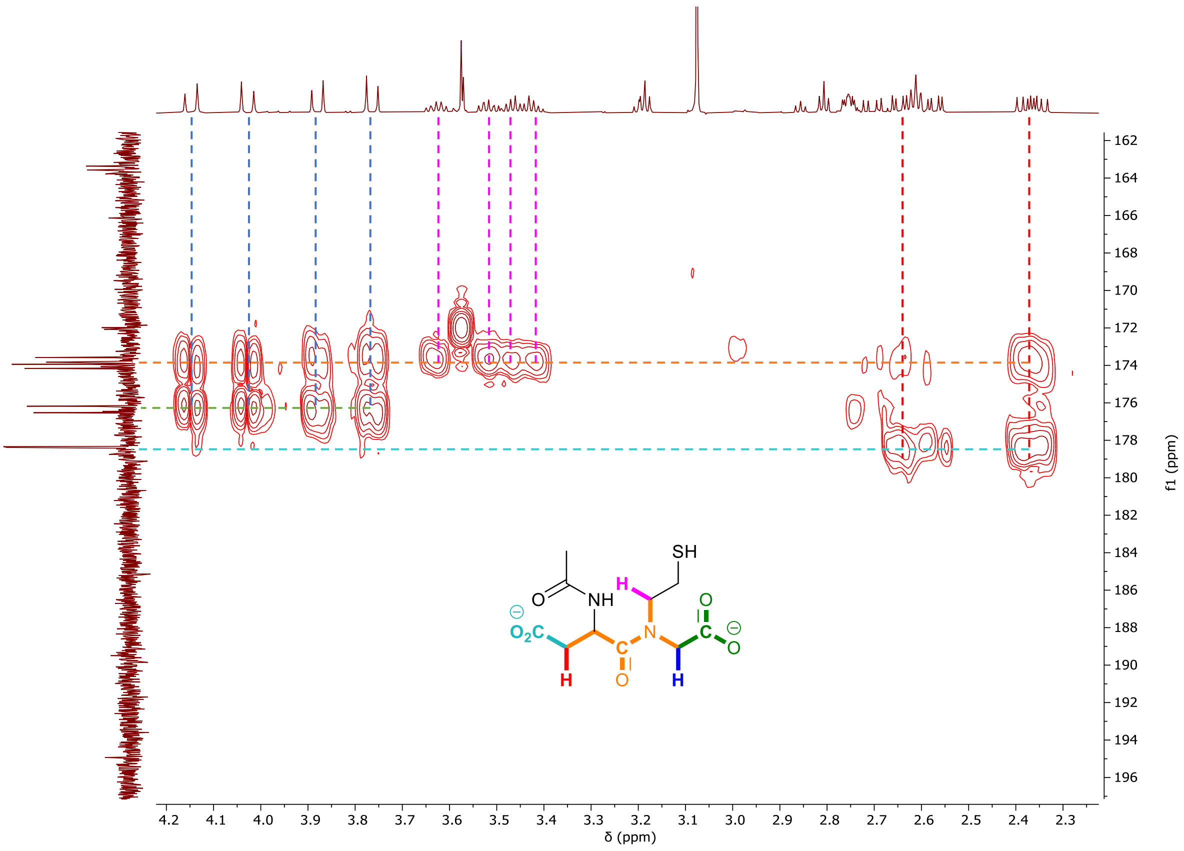

Figure S115: ^{1}H– ^{13}C HMBC (^{1}H: 700 MHz [2.2-4.2 ppm], ^{13}C: 175 MHz [160-195 ppm]) spectrum to show the diagnostic $^{3}J_{CH}$ and $^{2}J_{CH}$ coupling of α-Gly, α-Asp and *N*-CH_2 to tertiary amide carbon at pH 7, that is characteristic of **10j**.

Following **General procedure D** (pH 9), **10j** was observed in near quantitative yield (>95%) in 15 h.

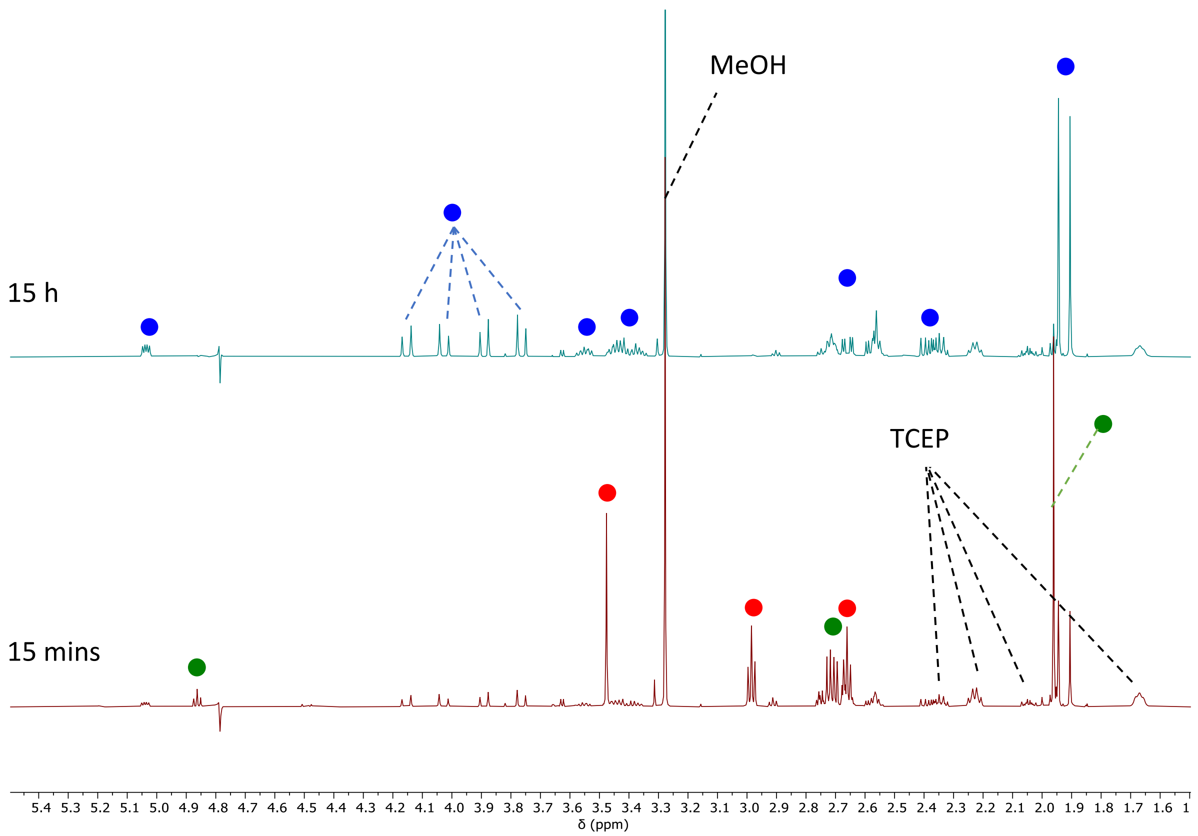

Figure S116: ^{1}H NMR (600 MHz, H_2O/D_2O 9:1, noesygppr1d, 1.5-5.5 ppm) spectra to show the reaction of **16$_G$** (45 mM), Ac-Asp-CN (**2$_D$**, 50 mM) and TCEP (10 mM) in borate buffer (pH 9, 500 mM) at room temperature, which yields **10j**.

Ac-Val-CN ($\mathbf{2_V}$):

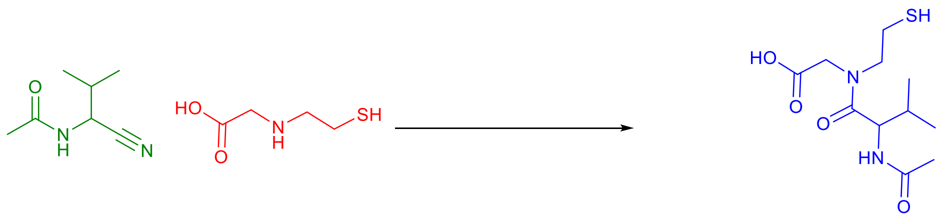

$\mathbf{2_V}$ $\mathbf{16_G}$ **10k**

Following **General procedure D** (pH 7), **10k** was observed in 30% yield, but incubating the reaction upto 4 days yielded **10k** in upto 60% yield. Heating the reaction yielded **10k** in upto 90% yield, summarised below in Supplementary Table 5:

Entry	T/ºC	t/d	Yield/%
**1**	r.t.	1	30
**2**	r.t.	2	45
**3**	r.t.	3	55
**4**	r.t.	4	60
**5**	30	1	50
**6**	40	1	70
**7**	50	1	85
**8**	60	1	90

Table S5: Table showing the ligation yield for $\mathbf{2_V}$ (50 mM) and $\mathbf{16_G}$ (50 mM) in phosphate buffer (pH 7, 500 mM) at various temperatures to yield **10k**.

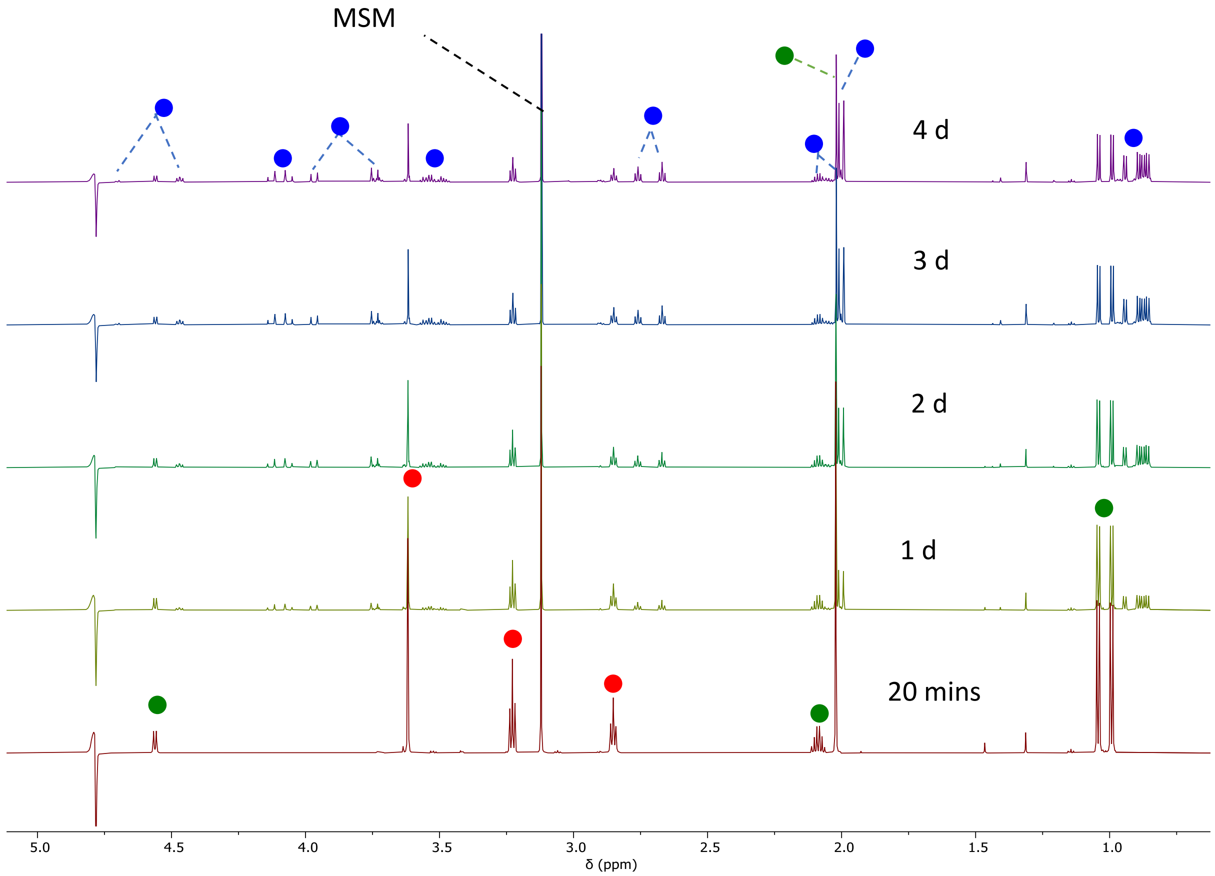

Figure S117: ^{1}H NMR (700 MHz, H_2O/D_2O 9:1, noesygppr1d, 0.0-5.2 ppm) spectra to show the reaction of **16_G** (50 mM) and Ac-Val-CN (**2_V**, 50 mM) with MSM (25 mM; internal standard) in phosphate buffer (pH 7, 500 mM) at room temperature, which yields **10k**.

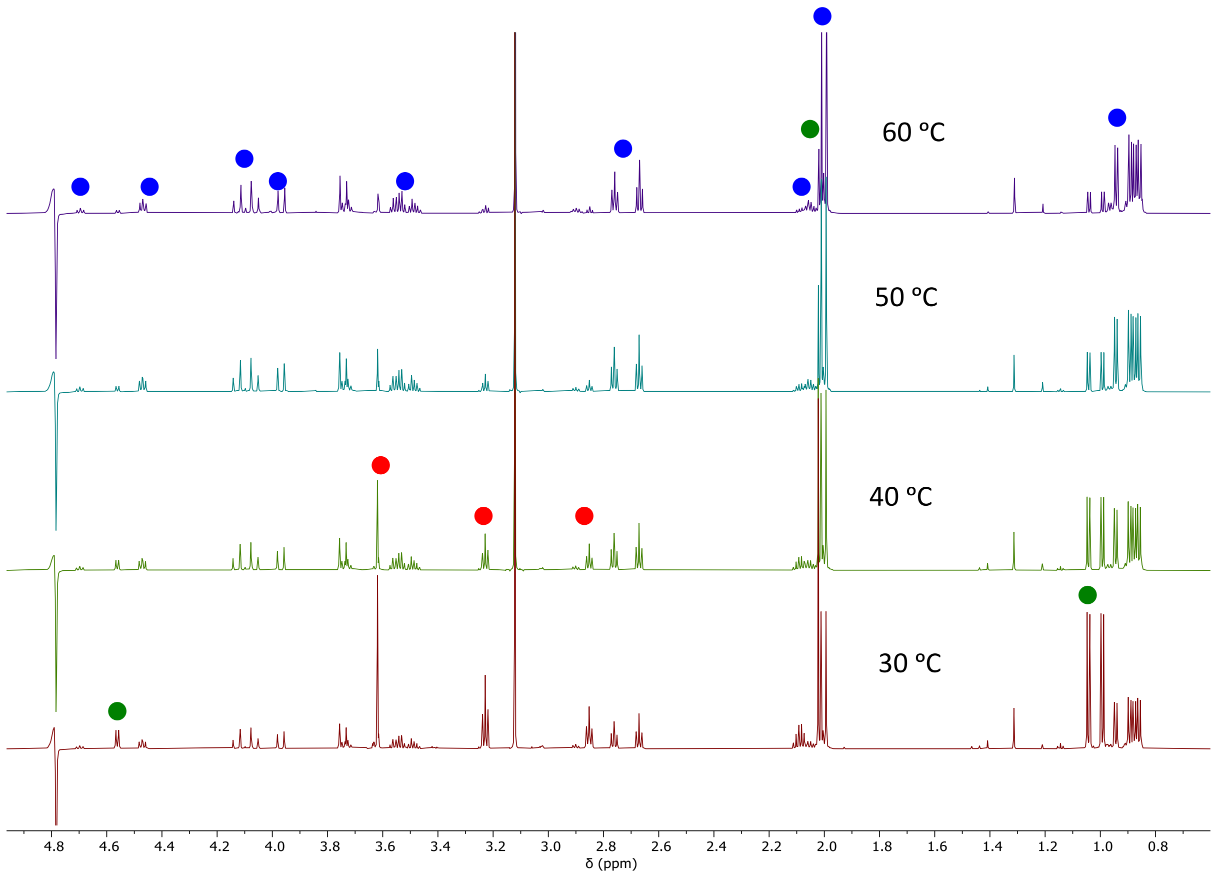

Figure S118: ^{1}H NMR (700 MHz, H_2O/D_2O 9:1, noesygppr1d, 0.6-5.0 ppm) spectra to show the reaction of **16**$_{G}$ (50 mM) and Ac-Val-CN (**2**$_{V}$, 50 mM) with MSM (25 mM; internal standard) in phosphate buffer (pH 7, 500 mM) at 30-60 ^{o}C, which yields **10k**.

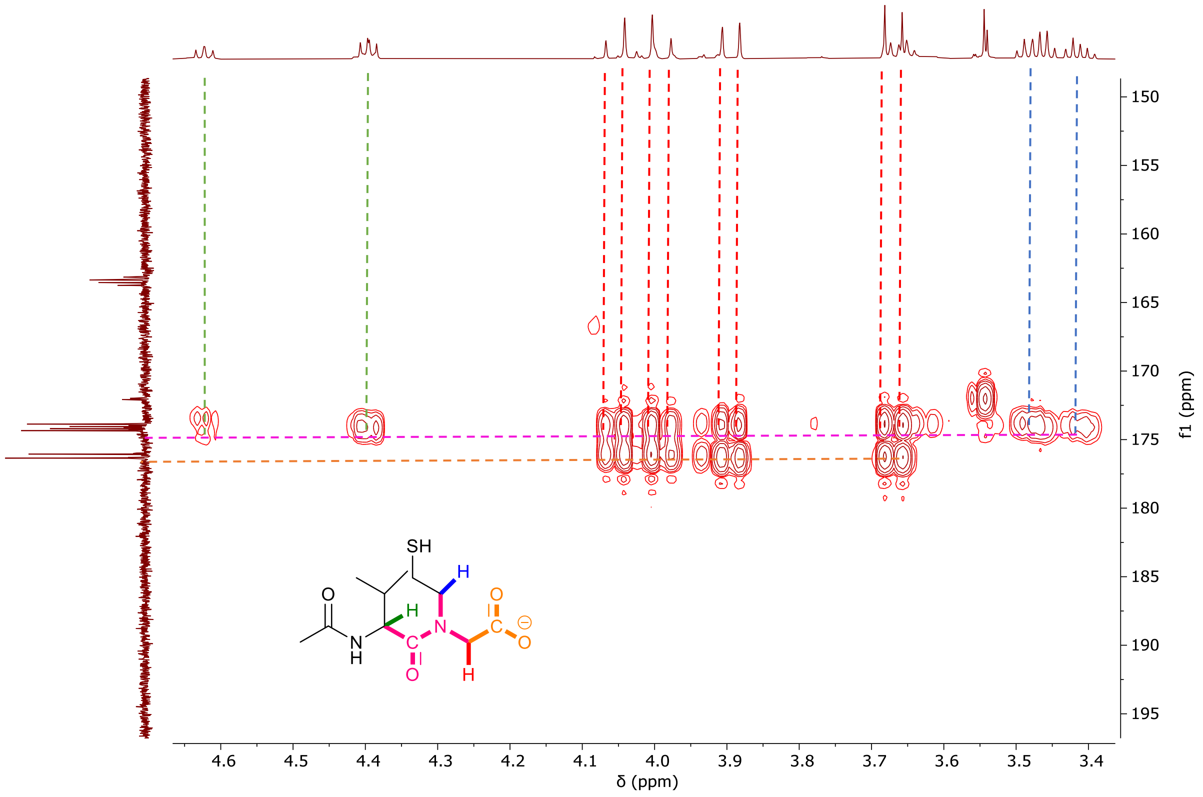

Figure S119: ^{1}H– ^{13}C HMBC (^{1}H: 700 MHz [3.4-4.7 ppm], ^{13}C: 175 MHz [150-195 ppm]) spectrum to show the diagnostic $^{3}J_{CH}$ and $^{2}J_{CH}$ coupling of α-Gly, α-Val and *N*-CH_2 to tertiary amide carbon at pH 7, that is characteristic of **10k**.

Following **General procedure D** (pH 9), **10k** was observed in near quantitative yield (>95%) in 72 h.

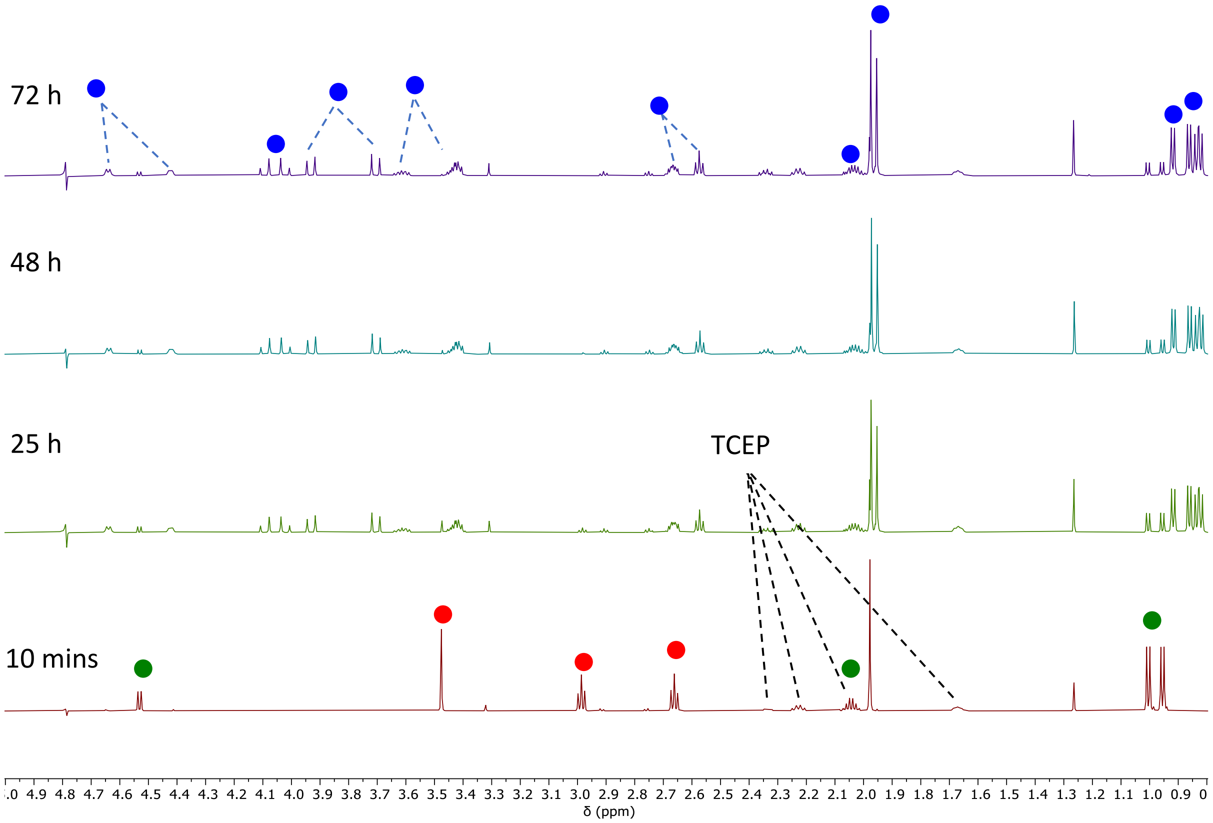

Figure S120: ^1H NMR (600 MHz, H_2O/D_2O 9:1, noesygppr1d, 0.8-5.0 ppm) spectra to show the reaction of **16**$_G$ (45 mM), Ac-Val-CN (**2**$_V$, 50 mM) and TCEP (10 mM) in borate buffer (pH 9, 500 mM) at room temperature, which yields **10k**.

.

Ac-Ile-CN (**2**$_I$)

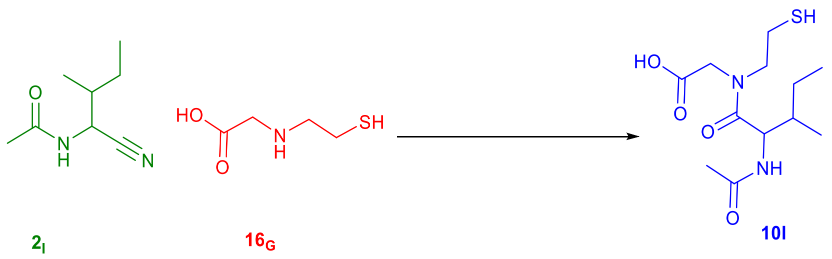

Following **General procedure D** (pH 7), **10l** was observed in 30-61% yield in 1-7 days at room temperature, and 75% yield in 24 hours at 60 °C.

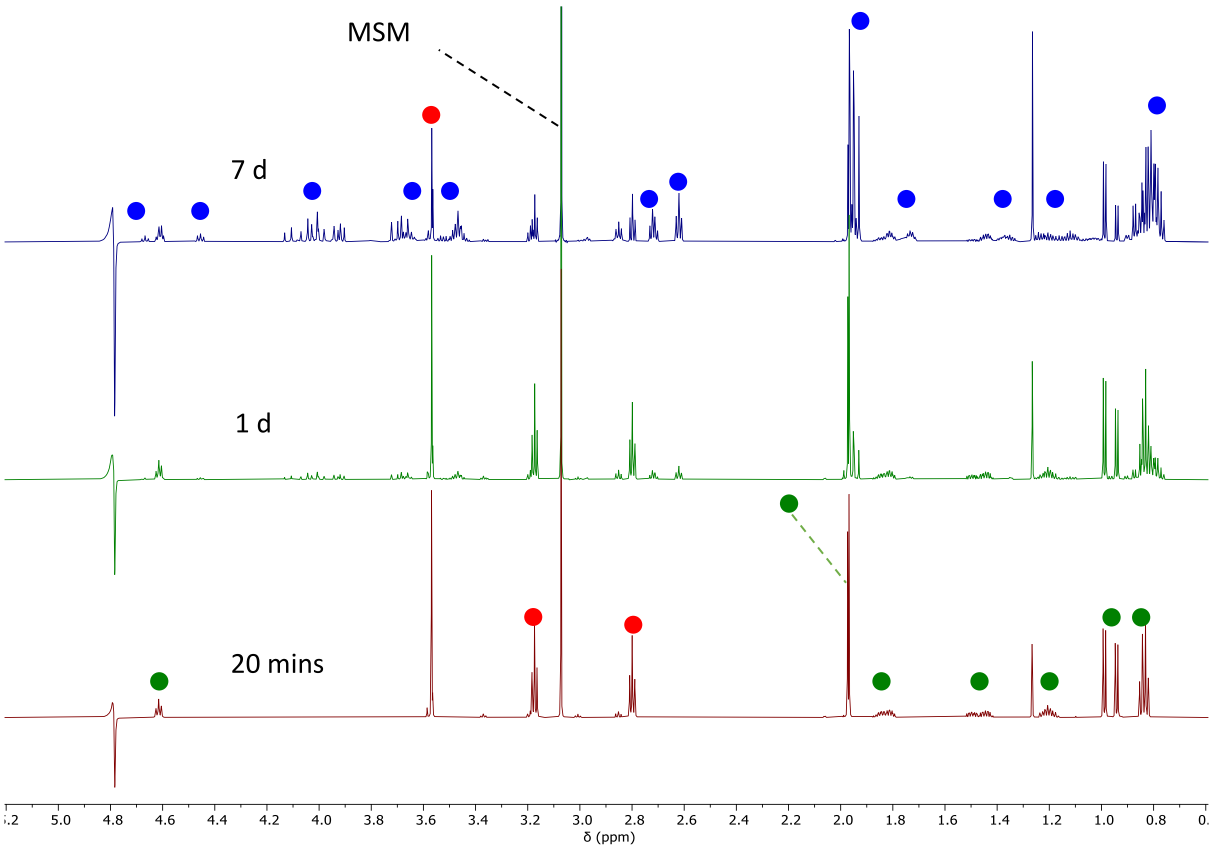

Figure S121 : ^{1}H NMR (700 MHz, H_2O/D_2O 9:1, noesygppr1d, 0.6-5.2 ppm) spectra to show the reaction of **16**$_G$ (50 mM) and Ac-Ile-CN (**2**$_I$, 50 mM) with MSM (25 mM, internal standard) in phosphate buffer (pH 7, 500 mM) at room temperature, which yields **10l**.

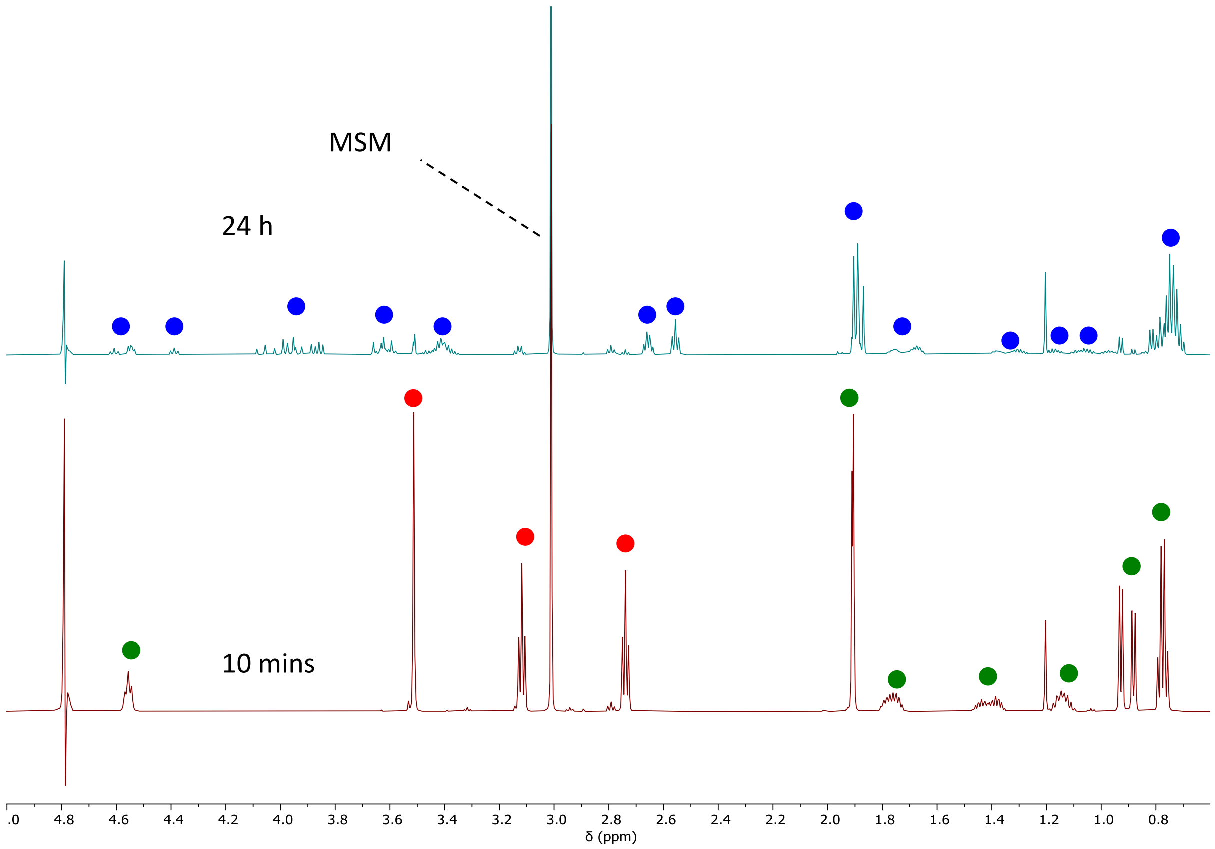

Figure S122: ^{1}H NMR (700 MHz, H_2O/D_2O 9:1, noesygppr1d, 0.6-5.2 ppm) spectra to show the reaction of **16**$_{\mathbf{G}}$ (50 mM) and Ac-Ile-CN (**2**$_{\mathbf{I}}$, 50 mM) with MSM (25 mM, internal standard) in phosphate buffer (pH 7, 500 mM) at 60 °C, which yields **10I**.

Following **General procedure D** (pH 9), **10l** was observed in near quantitative yield (>95%) in 96 h.

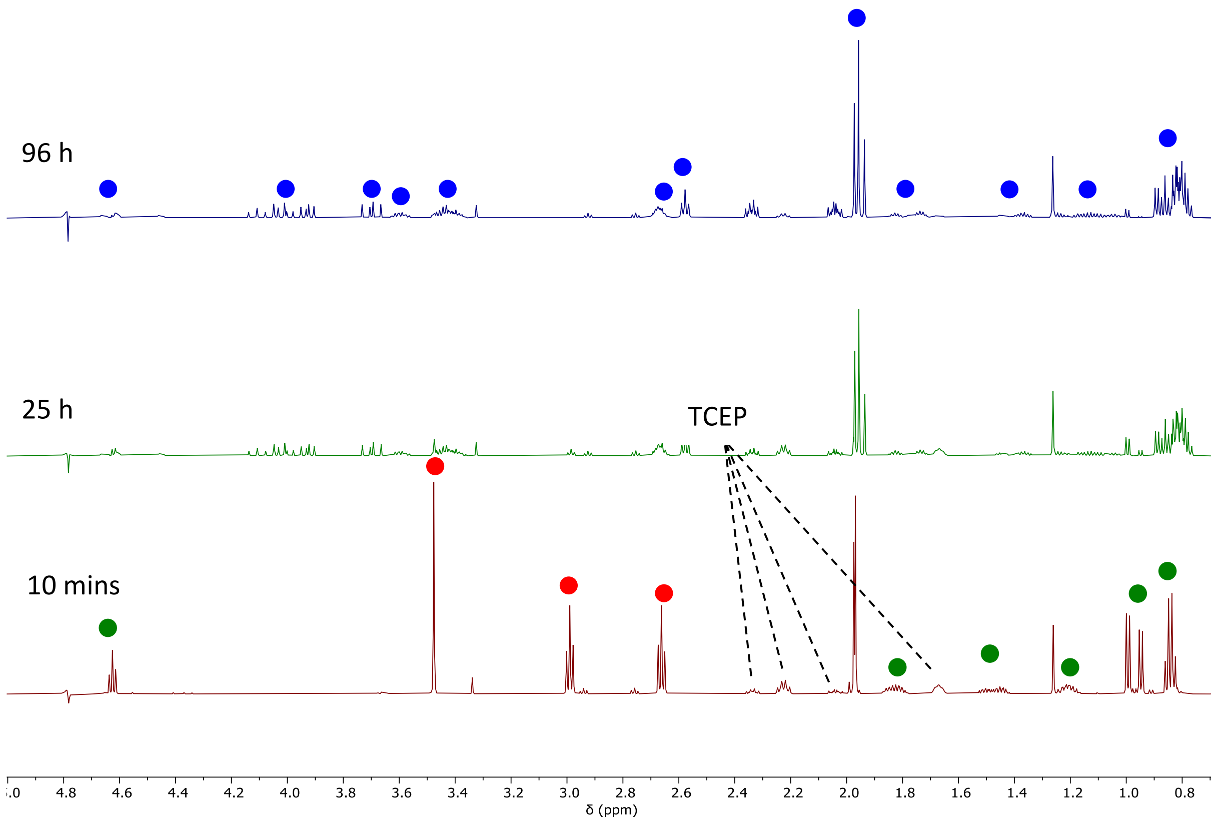

Figure S123: ^{1}H NMR (600 MHz, H_2O/D_2O 9:1, noesygppr1d, 0.8-5.0 ppm) spectra to show the reaction of **16$_G$** (45 mM), Ac-Ile-CN (**2$_I$**, 50 mM) and TCEP (10 mM) in borate buffer (pH 9, 500 mM) at room temperature, which yields **10l**.

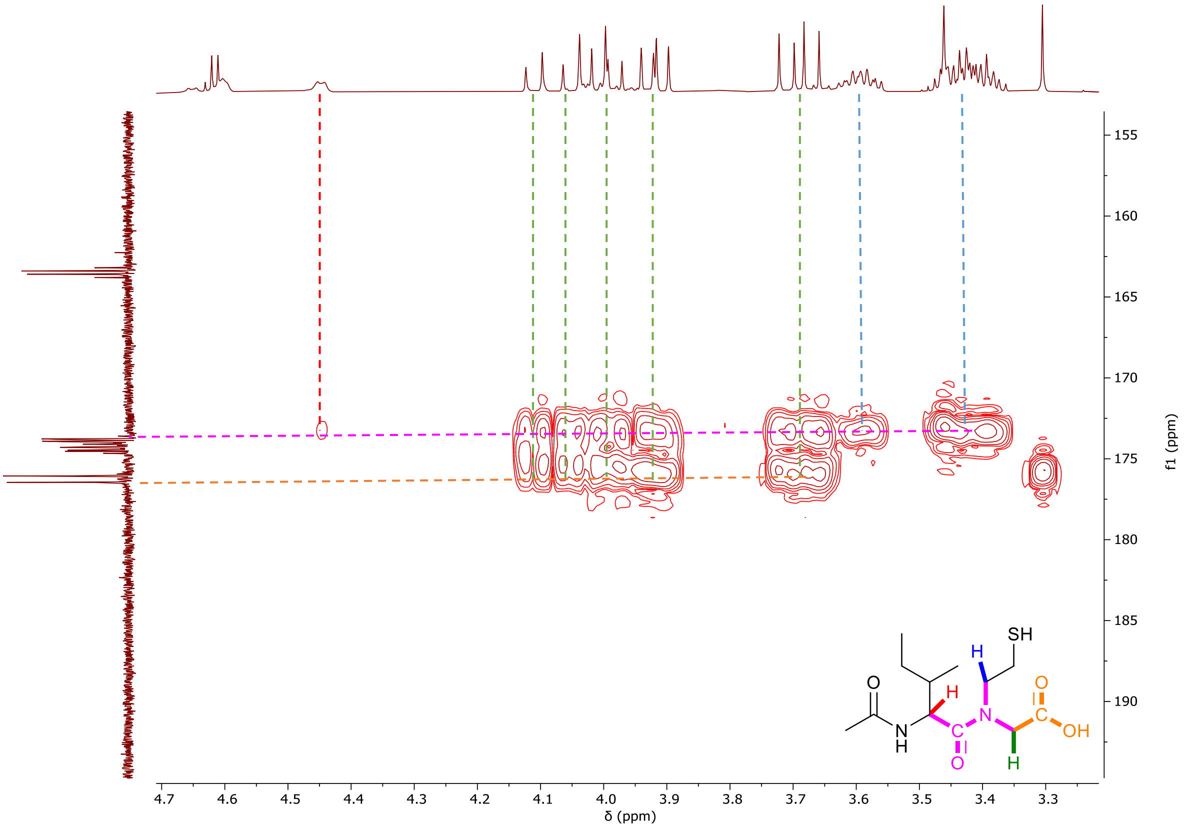

Figure S124: ^{1}H– ^{13}C HMBC (^{1}H: 700 MHz [3.2-4.7 ppm], ^{13}C: 175 MHz [155-195 ppm]) spectrum to show the diagnostic $^{3}J_{CH}$ and $^{2}J_{CH}$ coupling of α-Gly, α-iLe and *N*-CH_2 to tertiary amide carbon at pH 9, that is characteristic of **10l**.

Ac-Gly-Gly-CN ($\mathbf{2_{GG}}$):

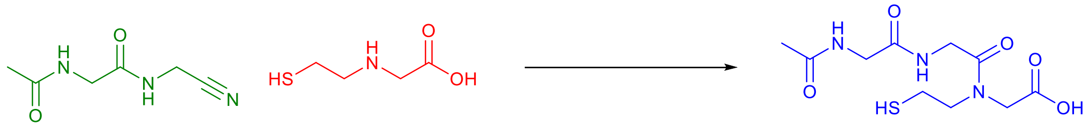

Following **General procedure D** (pH 7), **10m** was observed in 75% yield.

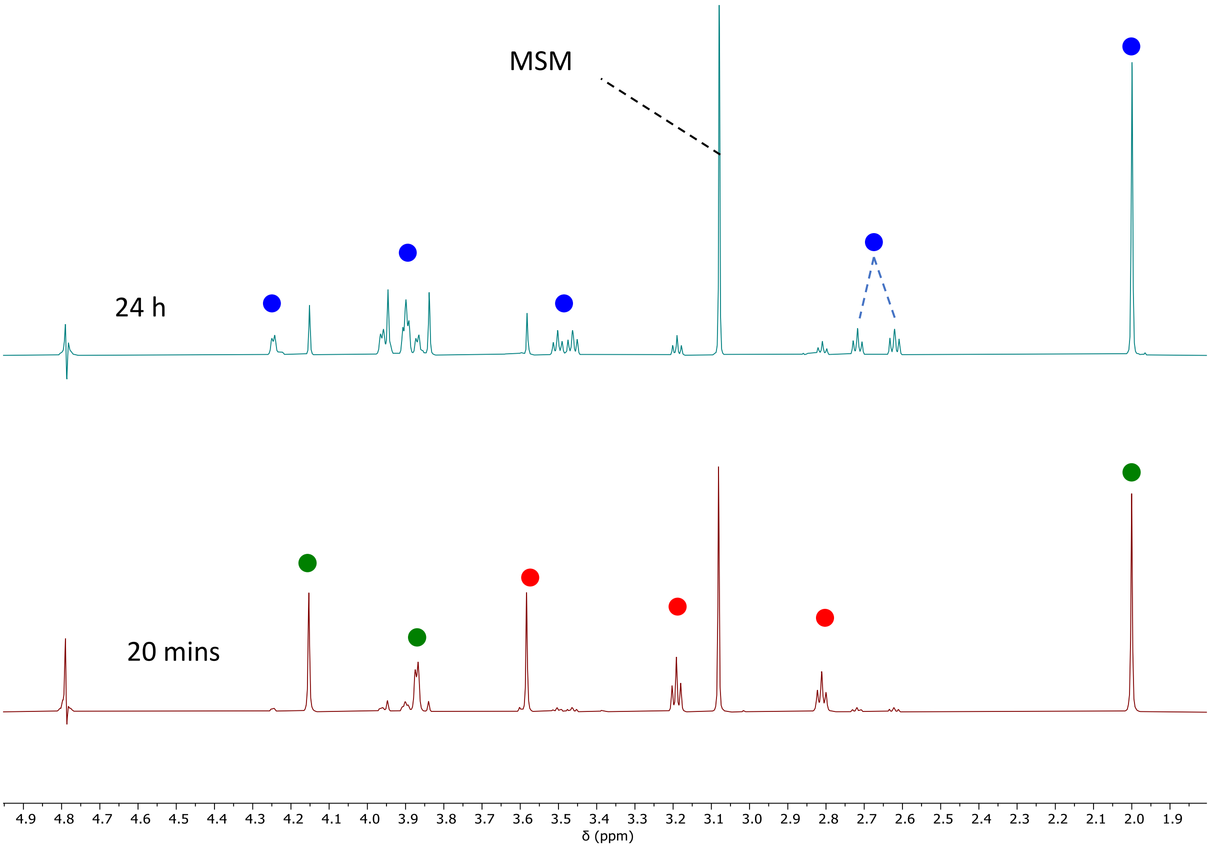

Figure S125: ^{1}H NMR (700 MHz, H_2O/D_2O 9:1, noesygppr1d, 1.8-4.9 ppm) spectra to show the reaction of $\mathbf{16_G}$ (50 mM) and $\mathbf{2_{GG}}$ (50 mM) with MSM (25 mM; internal standard) in phosphate buffer (pH 7, 500 mM) at room temperature, which yields **10m**.

Following **General procedure D** (pH 9), **10m** was observed in near quantitative yield (>95%) in 5 h.

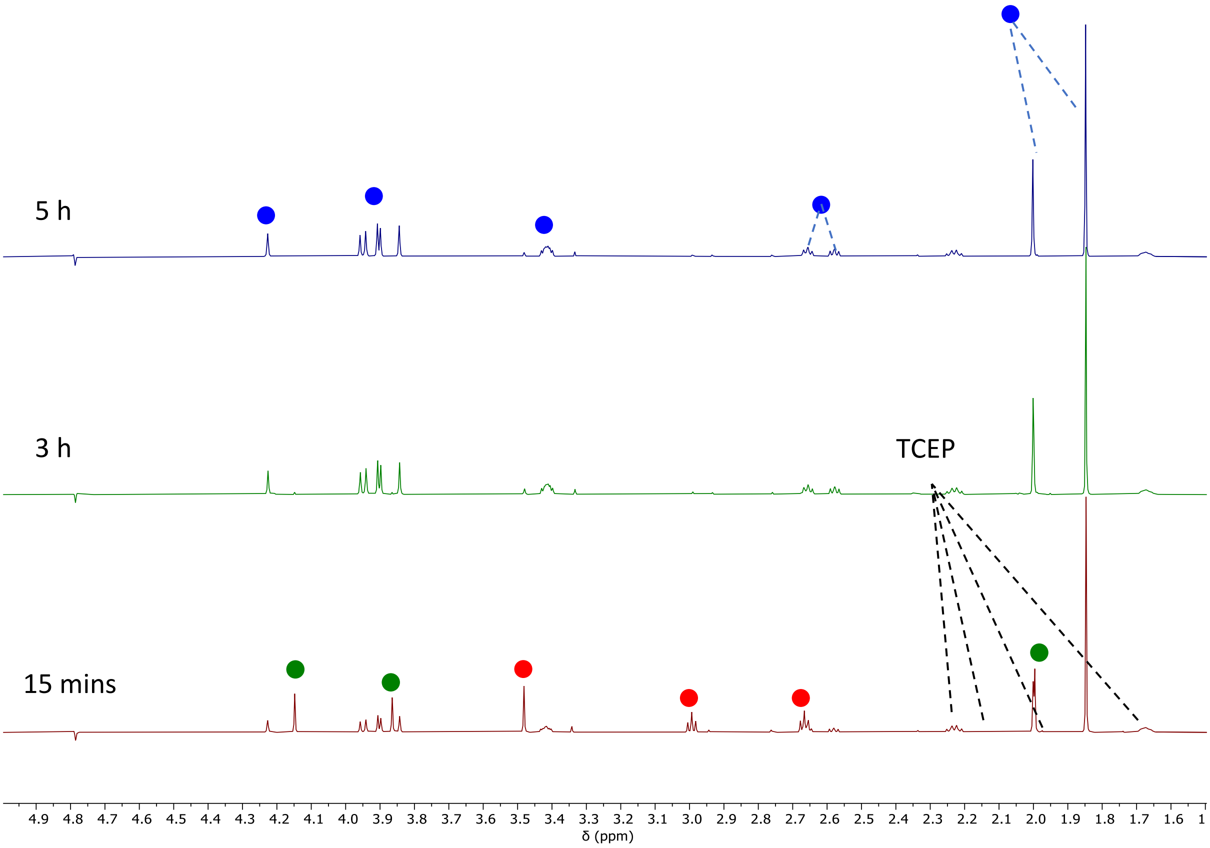

Figure S126: ^{1}H NMR (600 MHz, H_2O/D_2O 9:1, noesygppr1d, 0.8-5.0 ppm) spectra to show the reaction of **16**$_G$ (45 mM), **2**$_{GG}$ (40 mM) and TCEP (10 mM) in borate buffer (pH 9, 500 mM) at room temperature, which yields **10m**.

Ac-Gly-Gly-Gly-CN ($\mathbf{2_{GGG}}$):

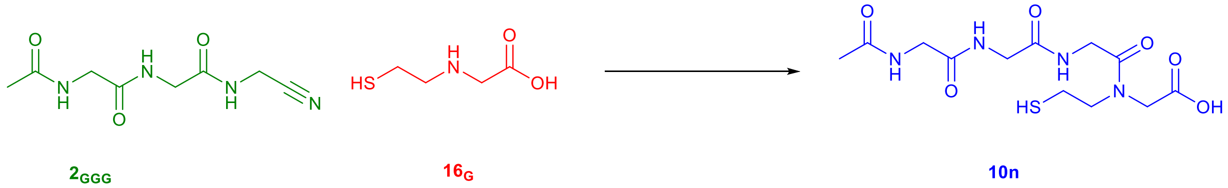

Following **General procedure D** (pH 7), **10n** was observed in 70% yield

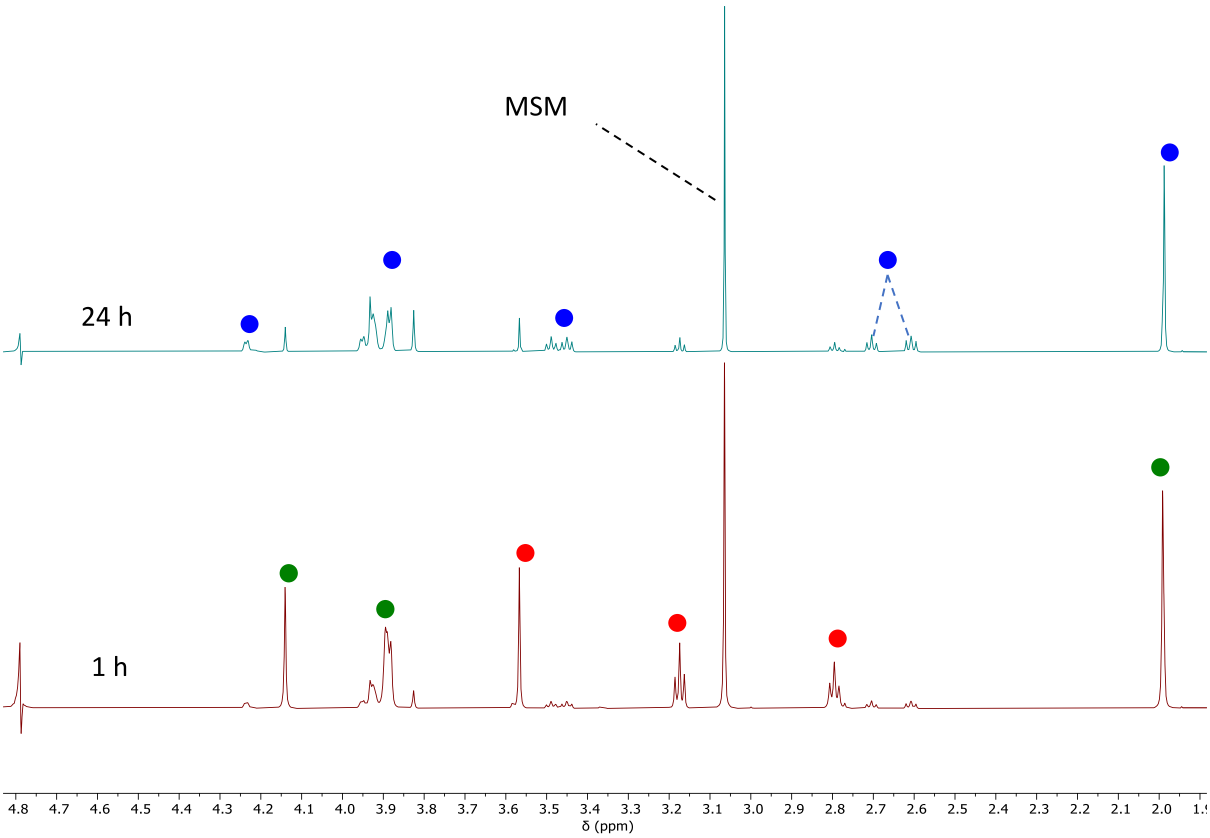

Figure S127: ^{1}H NMR (700 MHz, H_2O/D_2O 9:1, noesygppr1d, 1.9-4.8 ppm) spectra to show the reaction of $\mathbf{16_G}$ (50 mM) and $\mathbf{2_{GGG}}$ (50 mM) with MSM (25 mM; internal standard) in phosphate buffer (pH 7, 500 mM) at room temperature, which yields **10n**.

Following **General procedure D** (pH 9), **10n** was observed in near quantitative yield (>95%) in 10 h.

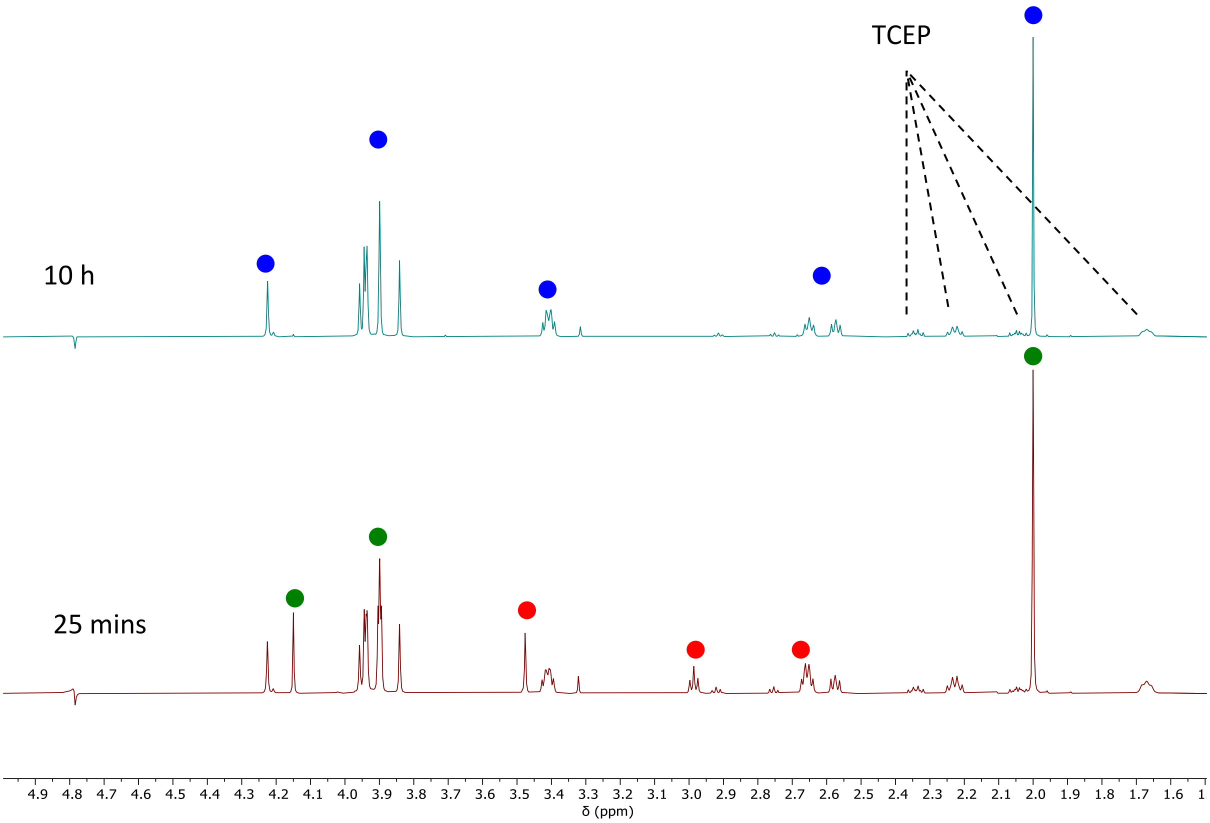

Figure S128: ^{1}H NMR (600 MHz, H_2O/D_2O 9:1, noesygppr1d, 1.5-5.0 ppm) spectra to show the reaction of $\mathbf{16_G}$ (45 mM), $\mathbf{2_{GGG}}$ (45 mM) and TCEP (10 mM) in borate buffer (pH 9, 500 mM) at room temperature, which yields **10n**.

Ac-Val-Gly-CN ($\mathbf{2_{VG}}$):

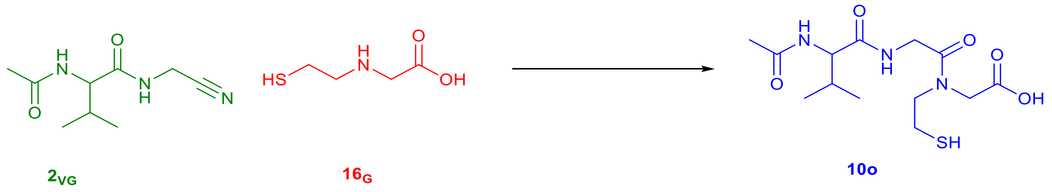

Following **General procedure D** (pH 7), **10o** was observed in 65% yield.

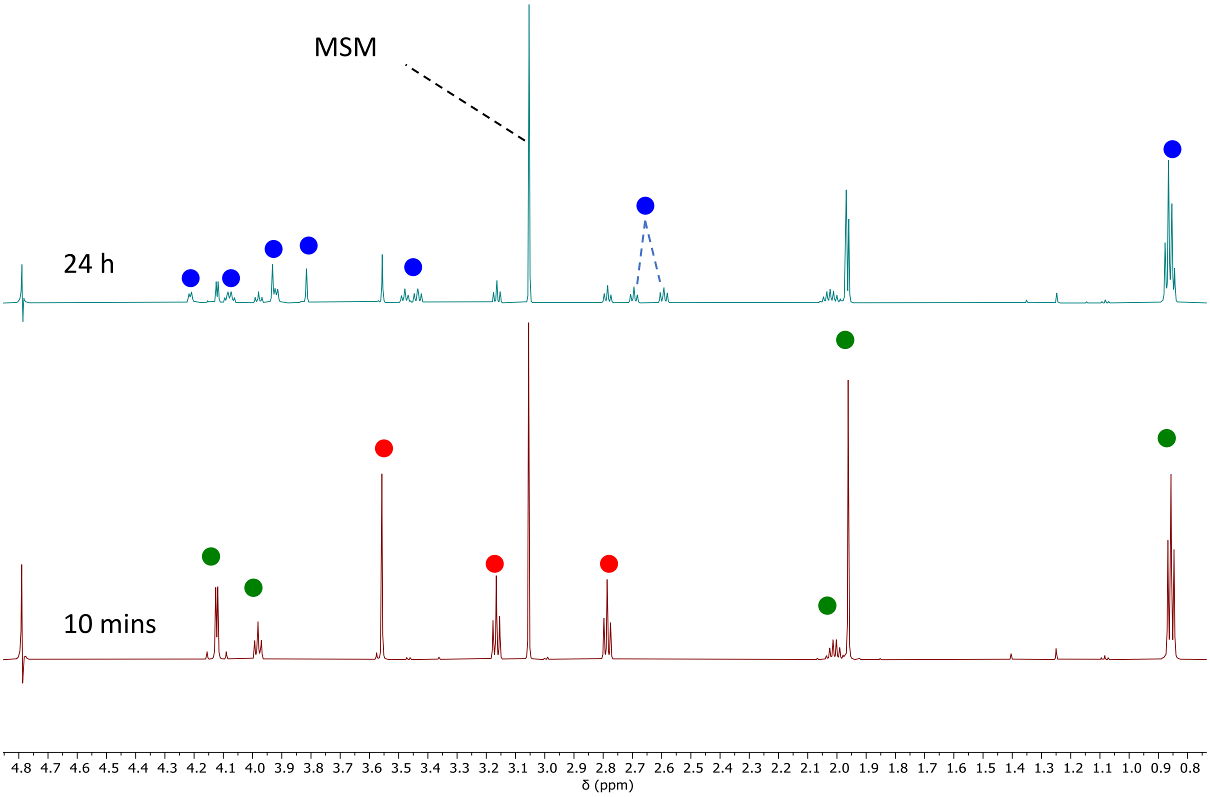

Figure S129: ^{1}H NMR (700 MHz, H_2O/D_2O 9:1, noesygppr1d, 0.7-4.8 ppm) spectra to show the reaction of $\mathbf{16_G}$ (50 mM) and $\mathbf{2_{VG}}$ (50 mM) with MSM (25 mM; internal standard) in phosphate buffer (pH 7, 500 mM) at room temperature, which yields **10o**.

Following **General procedure D** (pH 9), **10o** was observed in near quantitative yield (>95%) in 5 h.

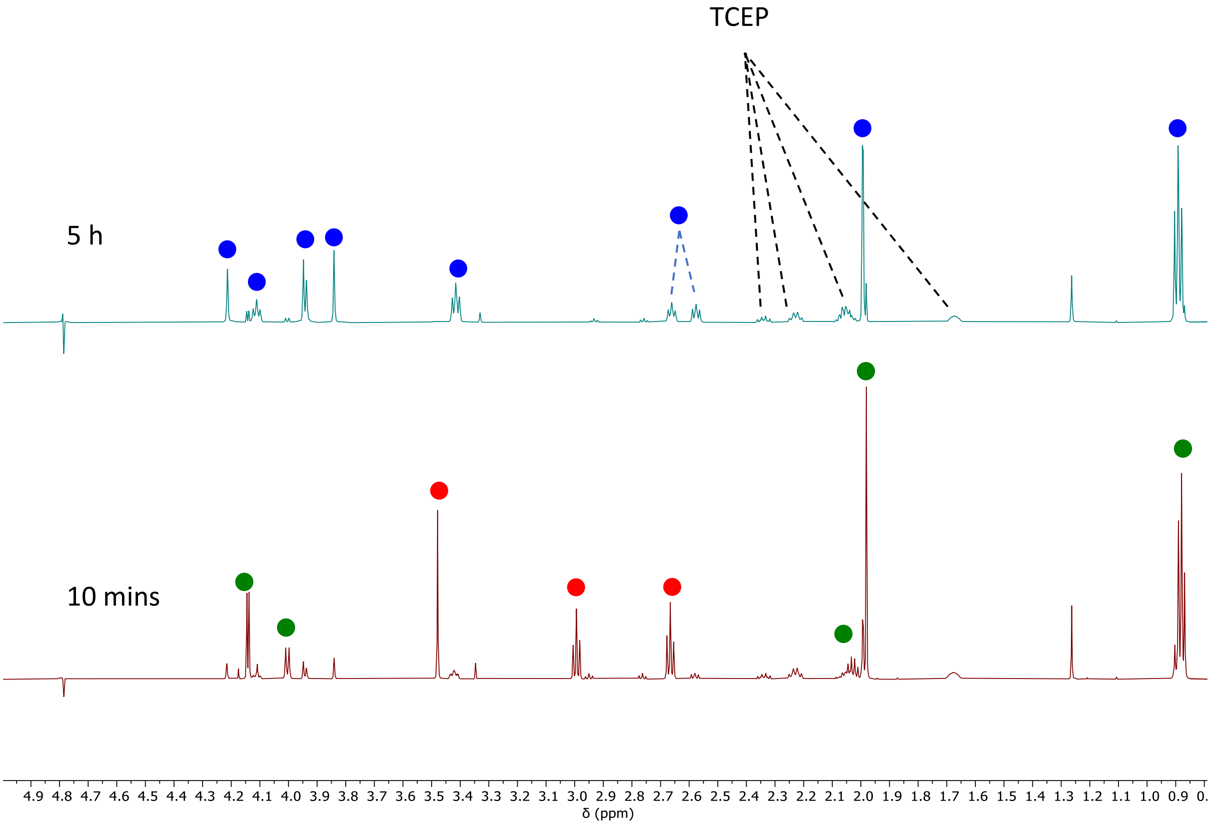

Figure S130: ^{1}H NMR (600 MHz, H_2O/D_2O 9:1, noesygppr1d, 0.8-5.0 ppm) spectra to show the reaction of $\mathbf{16_G}$ (45 mM), $\mathbf{2_{VG}}$ (50 mM) and TCEP (10 mM) in borate buffer (pH 9, 500 mM) at room temperature, which yields **10o**.

16_G selective ligation of α-amidonitriles:

$2_{βA}$ and **2_G** competition for **16_G**:

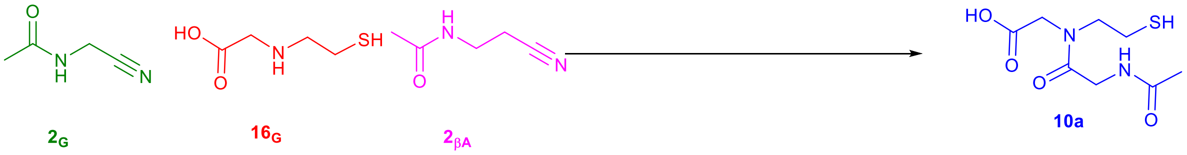

A solution of **16_G** (100 mM), Ac-Gly-CN **2_G** (100 mM), Ac-β-Ala-CN **$2_{βA}$** (100 mM) and MSM (50 mM; internal stanard) in phosphate buffer (pH 7, 500 mM in H_2O/D_2O (9:1), 1 mL) was incubated for 24 hours at room temperature. The reaction was then analysed by NMR spectroscopy and observed to yield **10a** selectively in 87% yield.

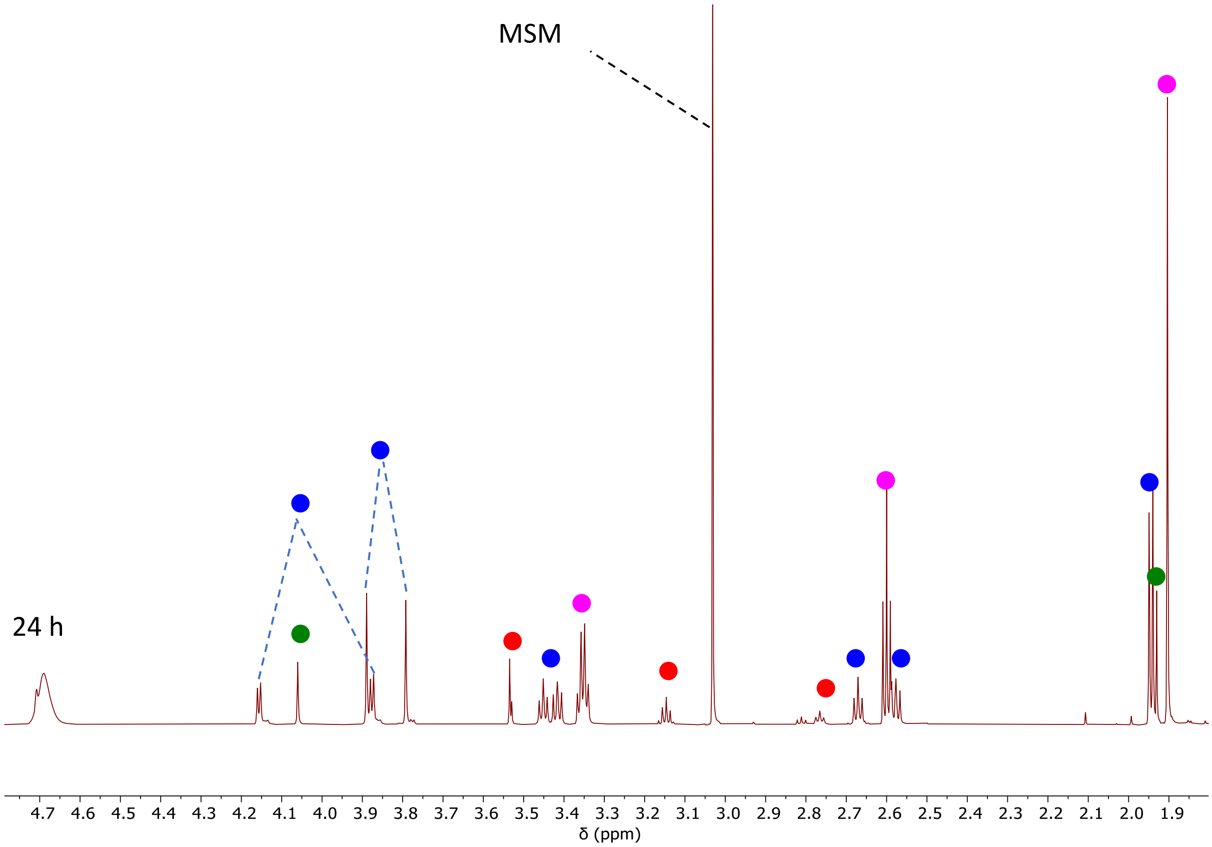

Figure S131: ^{1}H NMR (700 MHz, H_2O/D_2O 9:1, noesygppr1d, 1.8-4.8 ppm) spectrum to show the competition reaction of **16_G** (100 mM), **2_G** (100 mM) and **$2_{βA}$** (100 mM) with MSM (50 mM; internal standard) in phosphate buffer (pH 7, 500 mM) at room temperature to selectively yield **10a**.

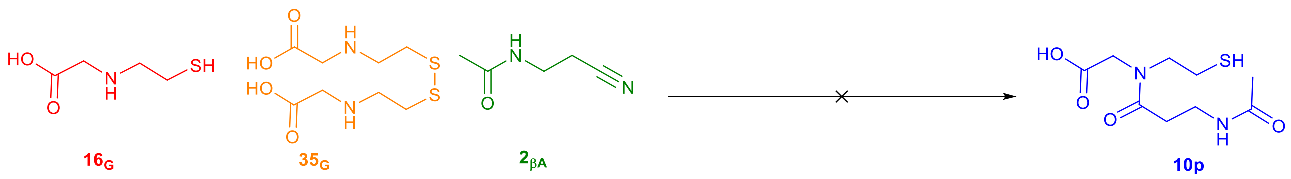

A solution of **16_G** (50 mM), Ac-β-Ala-CN **$2_{βA}$** (50 mM) and MSM (25 mM; internal standard) in phosphate buffer (pH 7, 500 mM in H_2O/D_2O (9:1), 1 mL) was incubated for 7 days at room temperature. The reaction was then analysed by NMR spectroscopy and observed not to yield **10p**, but instead the oxidation of **16_G** to **35_G** was observed.

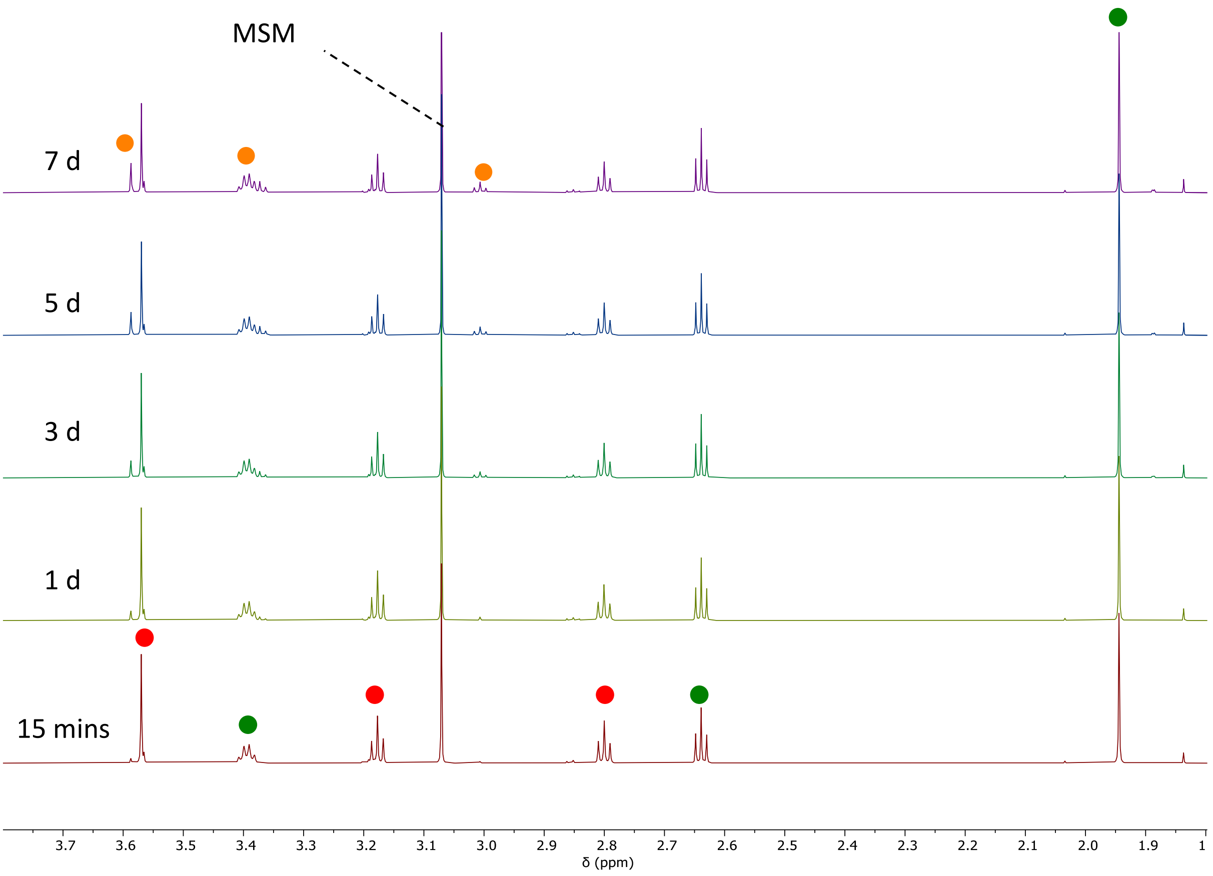

Figure S132: ^{1}H NMR (700 MHz, H_2O/D_2O 9:1, noesygppr1d, 1.8-3.8 ppm) spectra to show the reaction of **16_G** (50 mM) and **$2_{βA}$** (50 mM) with MSM (25 mM; internal standard) in phosphate buffer (pH 7, 500 mM) at room temperature, which illustrates the stability of **$2_{βA}$** under these conditions.

2_{Aib} and **2_G** competition for **16_G**:

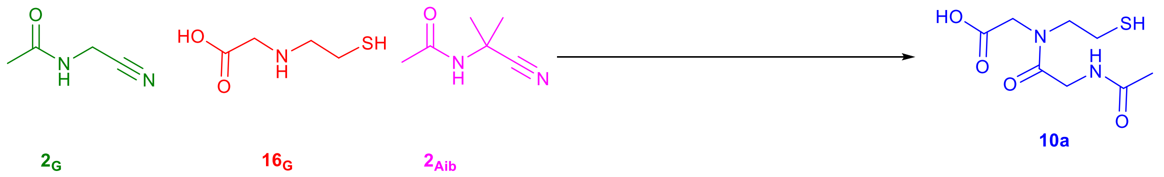

A solution of **16_G** (100 mM), Ac-Gly-CN **2_G** (100 mM), Ac-Aib-CN **2_{Aib}** (100 mM) and MSM (50 mM; internal standard) in phosphate buffer (pH 7, 500 mM in H_2O/D_2O (9:1), 1 mL) was incubated for 24 hours at room temperature. The reaction was then analysed by NMR spectroscopy and observed to yield **10a** selectively in 74% yield.

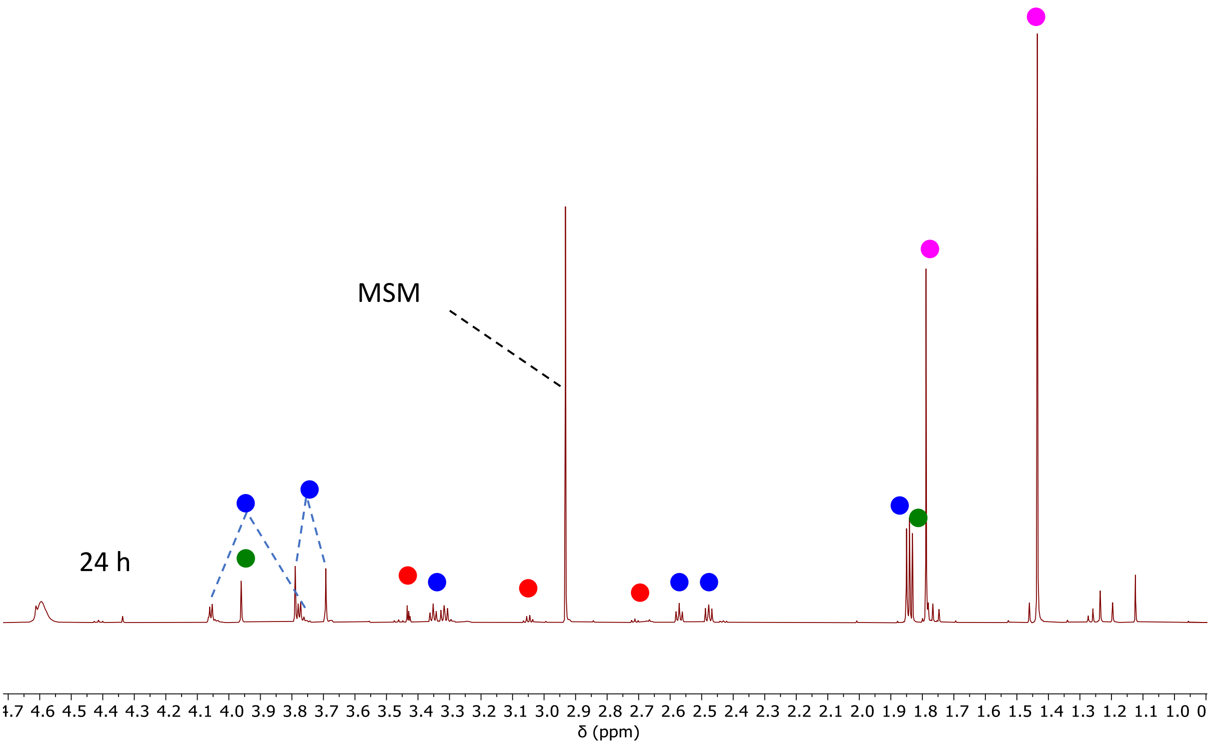

Figure S133: ^{1}H NMR (700 MHz, H_2O/D_2O 9:1, noesygppr1d, 0.9-4.7 ppm) spectrum to show the competition reaction of **16_G** (100 mM), **2_G** (100 mM) and **2_{Aib}** (100 mM) with MSM (50 mM; internal standard) in phosphate buffer (pH 7, 500 mM) at room temperature to selectively yield **10a**.

Selective $\mathbf{2_{DX}}$ ligation with $\mathbf{16_G}$:

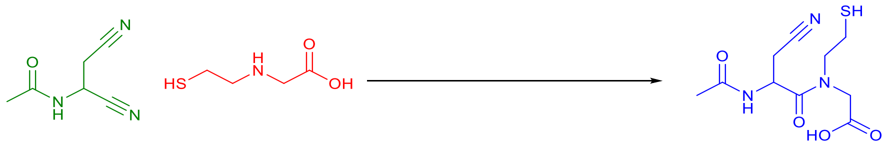

A solution of $\mathbf{16_G}$ (100 mM), Ac-Asx-CN $\mathbf{2_{DX}}$ (100 mM) and MSM (50 mM; internal standard) in phosphate buffer (pH 7, 500 mM in H_2O/D_2O (9:1), 1 mL) was incubated for 1 hour at room temperature. The reaction was then analysed by NMR spectroscopy and observed to yield **10q** selectively in near quantitative yield (>95%).

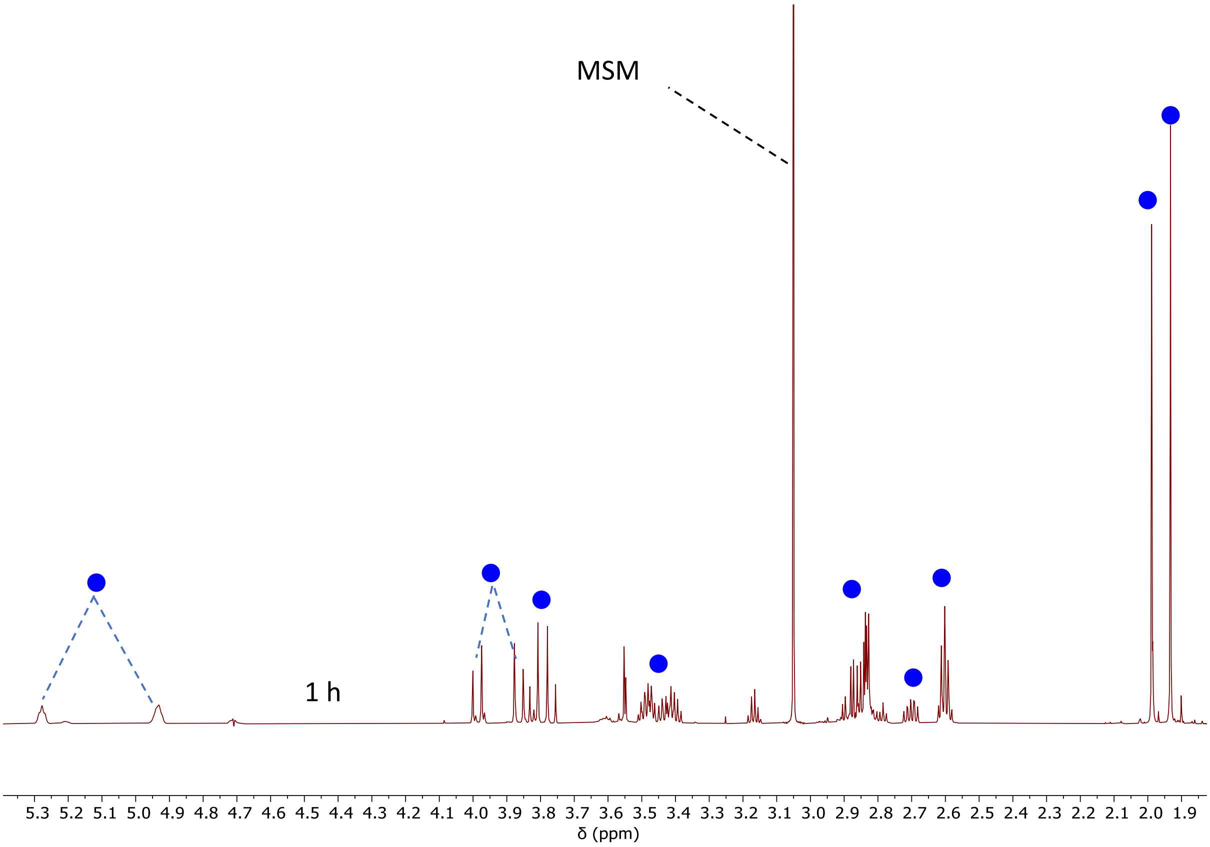

Figure S134: ^1H NMR (700 MHz, H_2O/D_2O 9:1, noesygppr1d, 1.8-5.4 ppm) spectrum to show the reaction of $\mathbf{16_G}$ (100 mM) and $\mathbf{2_{DX}}$ (100 mM) with MSM (50 mM; internal standard) in phosphate buffer (pH 7, 500 mM) at room temperature, which selectively yields **10q**.

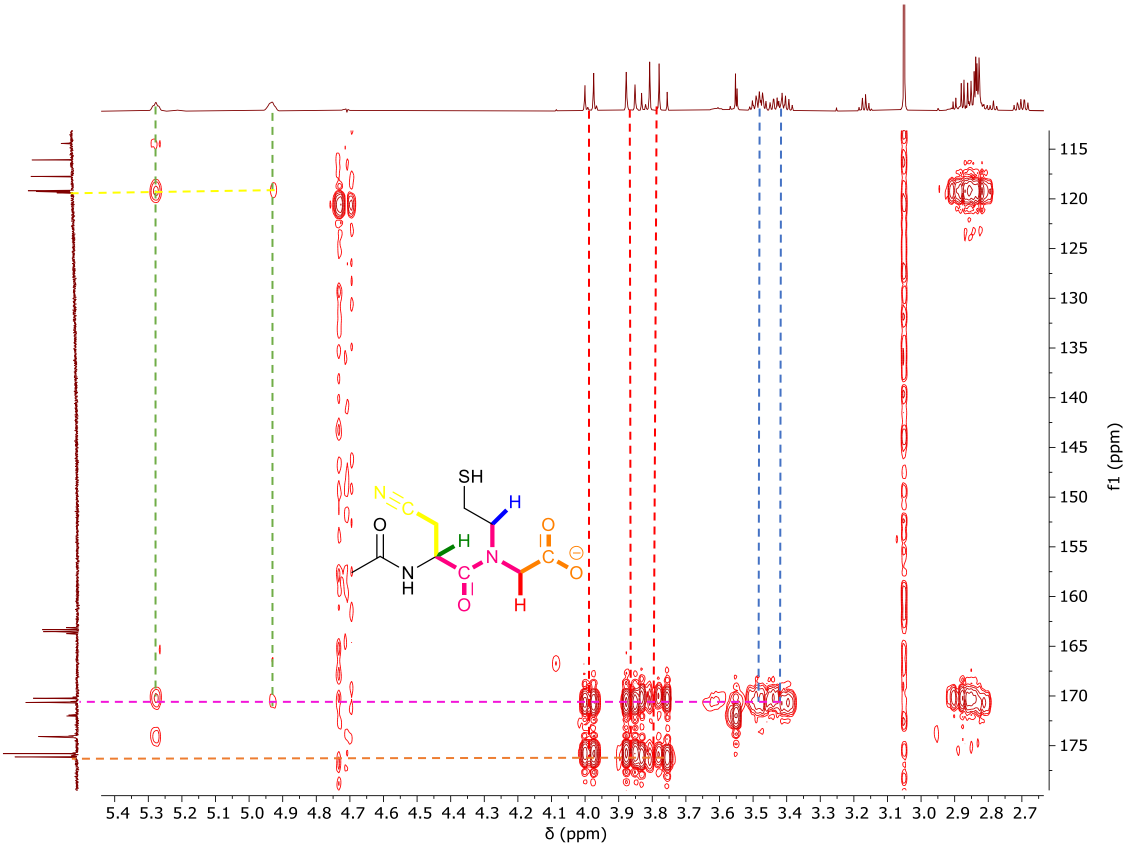

Figure S135: ^{1}H– ^{13}C HMBC (^{1}H: 700 MHz [2.7-5.4 ppm], ^{13}C: 175 MHz [115-180ppm]) spectrum to show the diagnostic $^3J_{CH}$ and $^2J_{CH}$ coupling of α-Gly, α-Asx and *N*-CH_2 to tertiary amide carbon, and the diagnostic $^3J_{CH}$ coupling of α-Asx to β-CN carbon at pH 7, that is characteristic of the dipeptoid **10q** formation.

A solution of **16$_G$** (200 mM), Ac-Asx-CN **2$_{DX}$** (100 mM) and MSM (50 mM; internal standard) in phosphate buffer (pH 7, 500 mM in H_2O/D_2O (9:1), 1 mL) was incubated for 2 days at room temperature. The reaction was then analysed by NMR spectroscopy and observed to yield **10q** selectively in near quantitative yield (>95%).

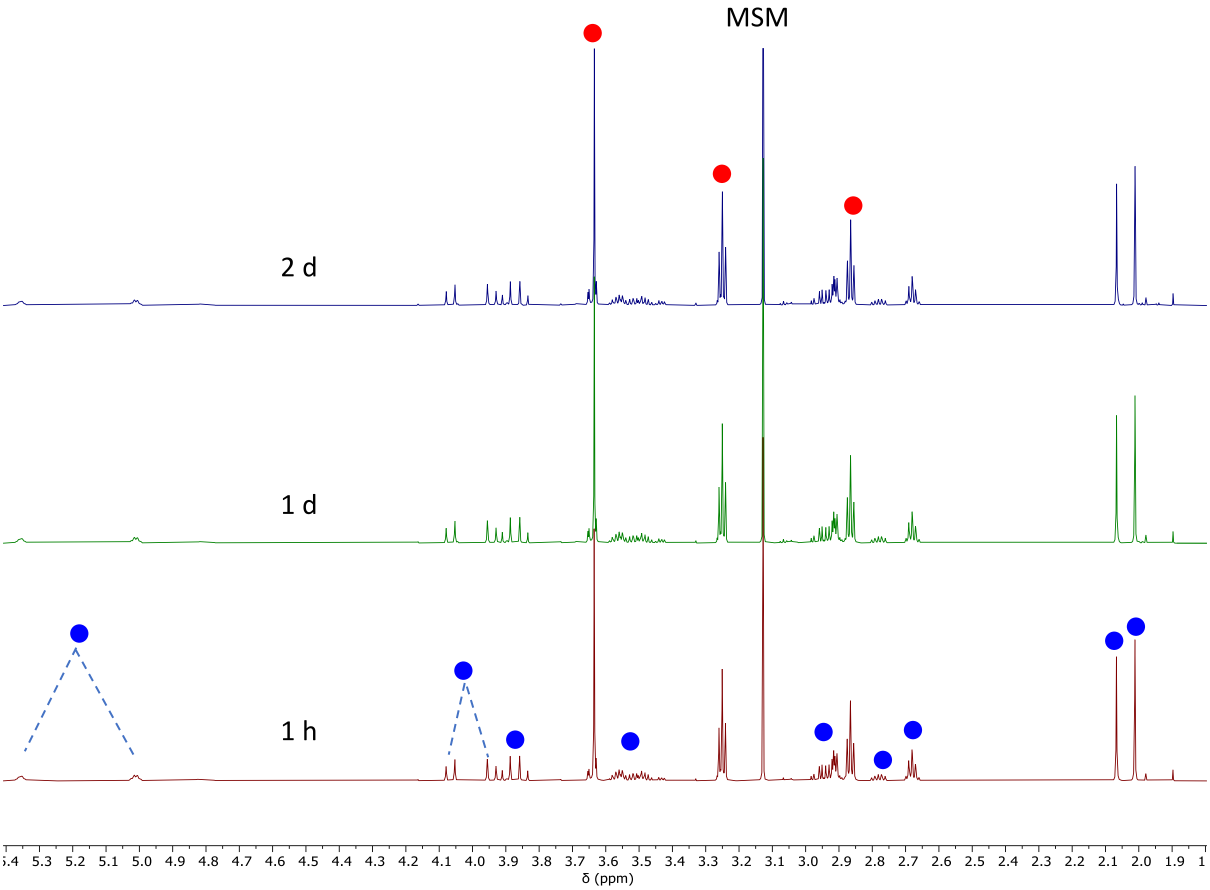

Figure S136: ^{1}H NMR (700 MHz, H_2O/D_2O 9:1, noesygppr1d, 1.8-5.4 ppm) spectra to show the reaction of **16$_G$** (200 mM) and **2$_{DX}$** (100 mM) with MSM (50 mM; internal standard) in phosphate buffer (pH 7, 500 mM) at room temperature to selectively yield **10q**. The reaction was monitored for 2 days and the second equivalent of **16$_G$** was observed not to attack the β-CN.

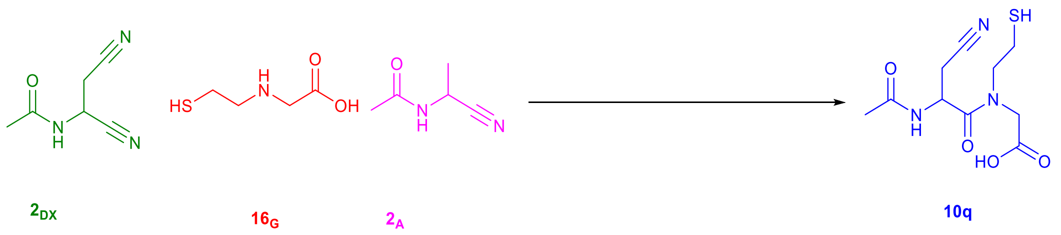

A solution of **16_G** (50 mM), Ac-Asx-CN **2_{DX}** (50 mM), Ac-Ala-CN **2_A** (50 mM) and MSM (25 mM; internal standard) in phosphate buffer (pH 7, 500 mM in H_2O/D_2O (9:1), 1 mL) was incubated for 1.5 hours at room temperature. The reaction was then analysed by NMR spectroscopy and observed to yield **10q** selectively in near quantitative yield (>95%).

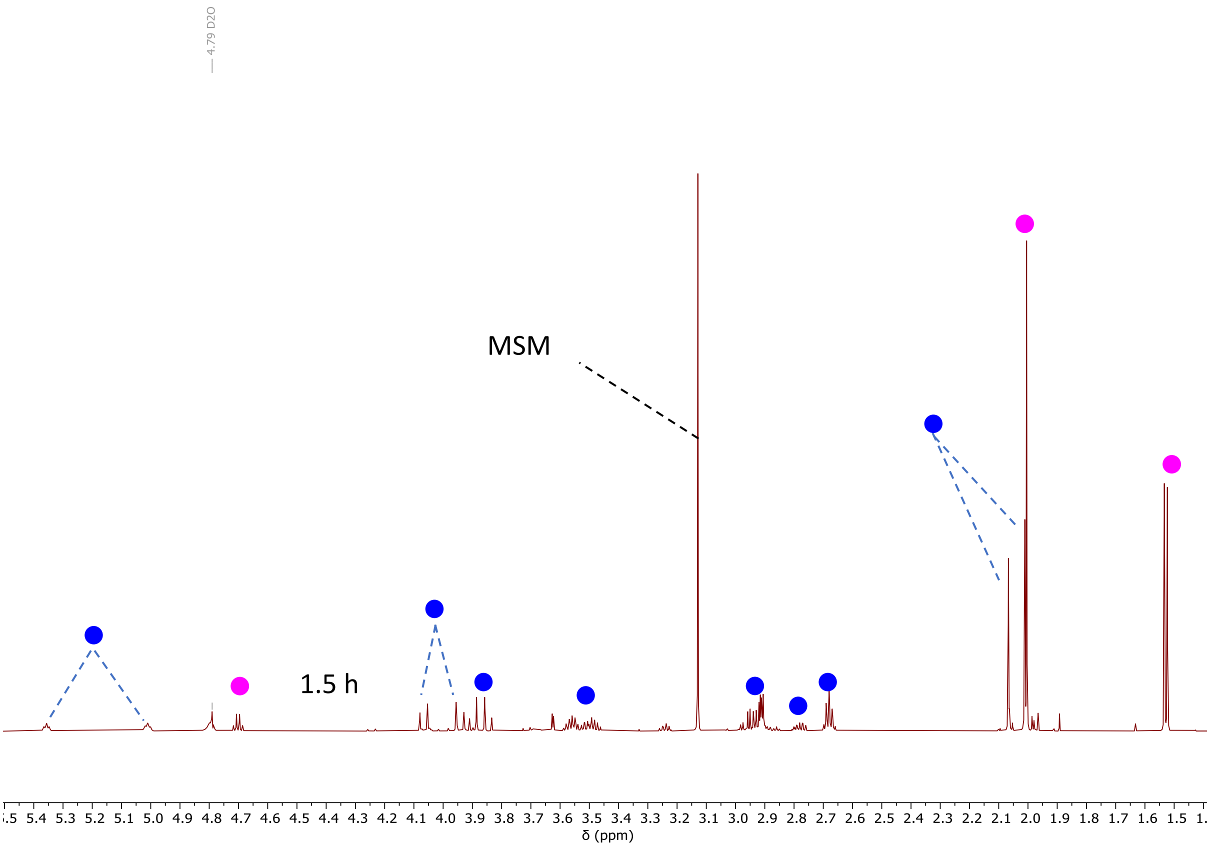

Figure S137: ^{1}H NMR (700 MHz, H_2O/D_2O 9:1, noesygppr1d, 1.4-5.5 ppm) spectrum to show the competition reaction of **16_G** (50 mM), **2_{DX}** (50 mM) and **2_A** (50 mM) with MSM (25 mM; internal standard) in phosphate buffer (pH 7, 500 mM) at room temperature to selectively yield **10q**.

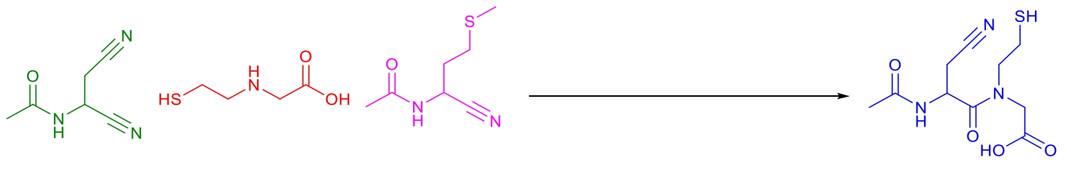

A solution of **16$_G$** (50 mM), Ac-Asx-CN **2$_{DX}$** (50 mM), Ac-Met-CN **2$_M$** (50 mM) and MSM (25 mM; internal standard) in phosphate buffer (1 mL, pH 7, 500 mM in H_2O/D_2O (9:1)) was incubated for 1.5 hours at room temperature. The reaction was then analysed by NMR spectroscopy and observed to yield **10q** selectively in near quantitative yield (>95%).

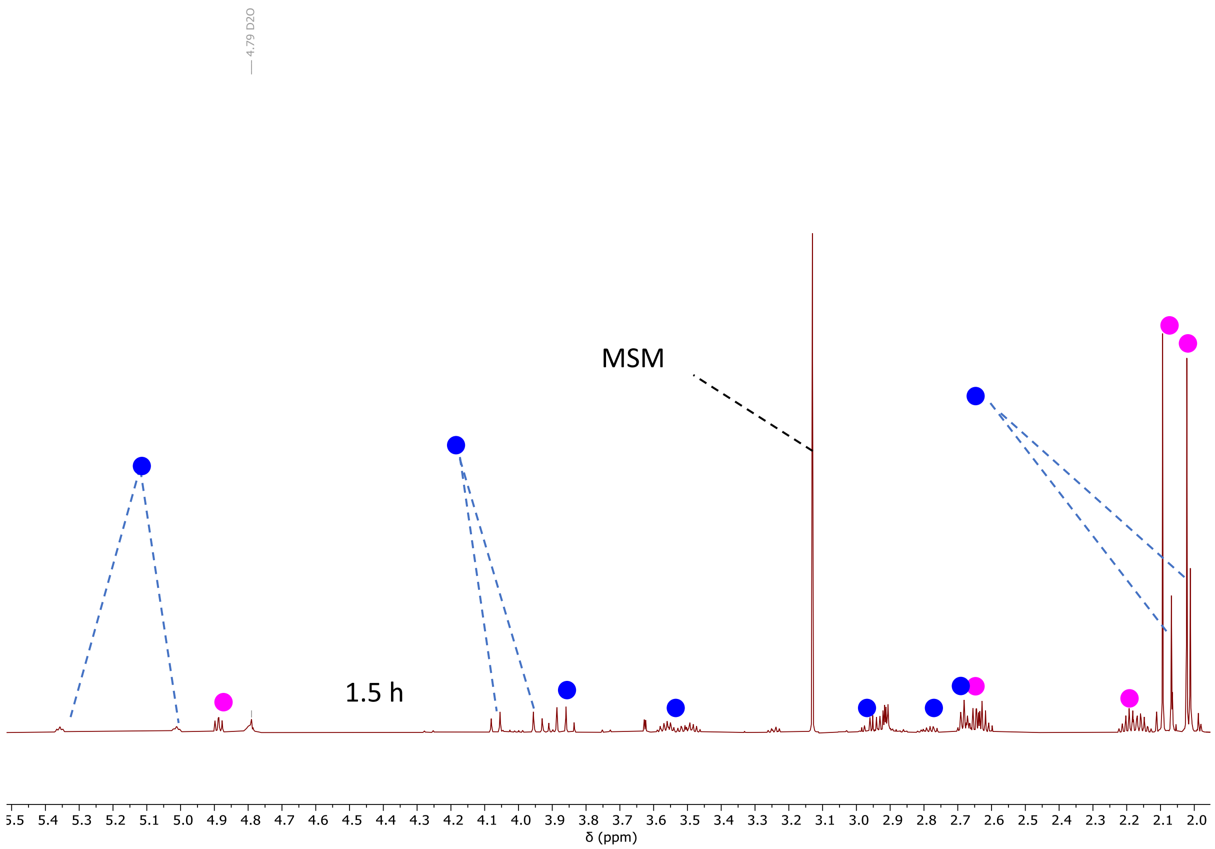

Figure S138: ^{1}H NMR (700 MHz, H_2O/D_2O 9:1, noesygppr1d, 2.0-5.5 ppm) spectrum to show the competition reaction of **16$_G$** (50 mM), **2$_{DX}$** (50 mM) and **2$_M$** (50 mM) with MSM (25 mM; internal standard) in phosphate buffer (pH 7, 500 mM) at room temperature to selectively yield **10q**.

Selectibe $\mathbf{2_{EX}}$ ligation with $\mathbf{16_G}$:

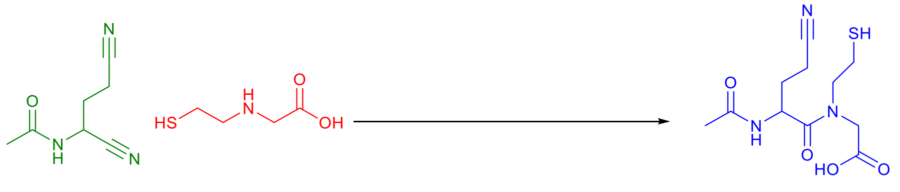

A solution of $\mathbf{16_G}$ (50 mM), Ac-Glx-CN $\mathbf{2_{EX}}$ (50 mM) and MSM (25 mM; internal standard) in phosphate buffer (1 mL, pH 7, 500 mM in H_2O/D_2O (9:1)) was incubated for 10 hours at room temperature. The reaction was then analysed by NMR spectroscopy and observed to yield **10r** selectively in near quantitative yield (>95%).

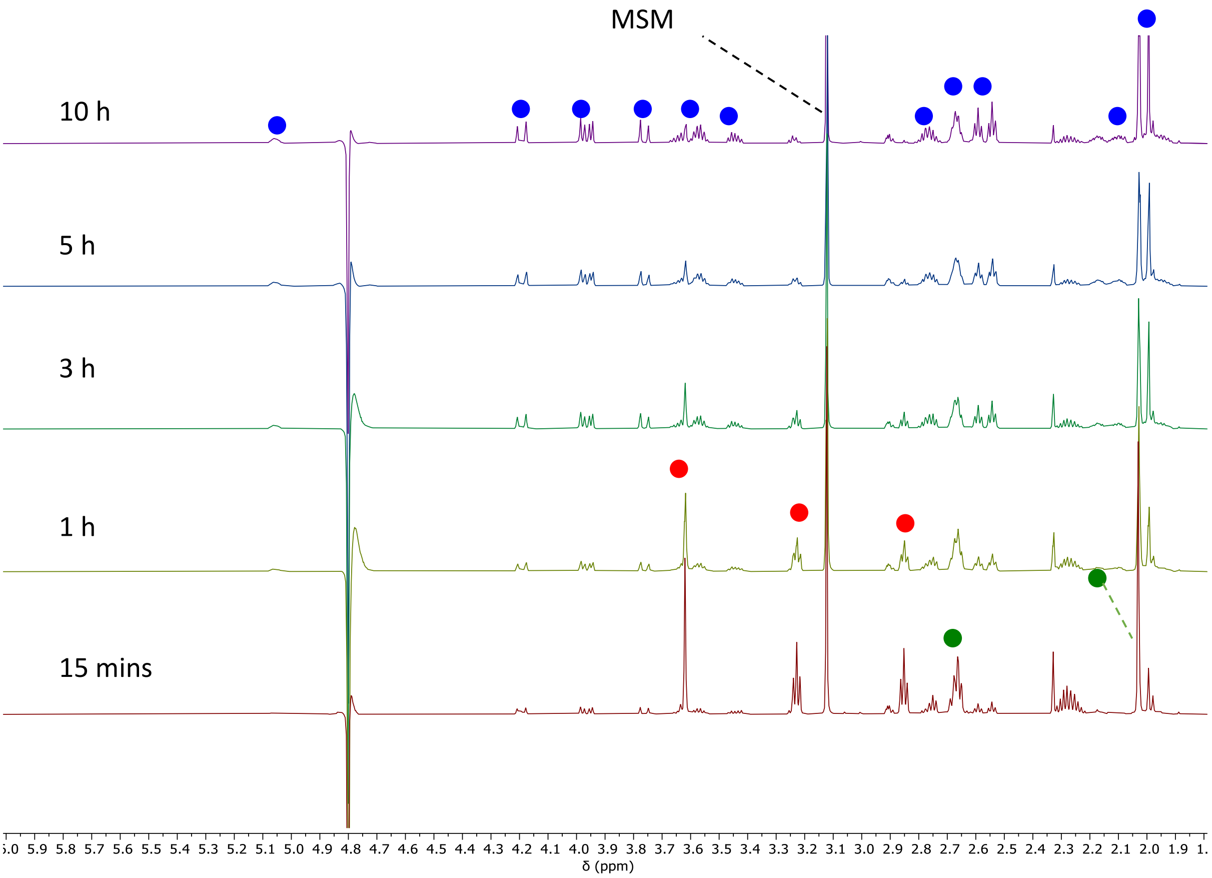

Figure S139: ^{1}H NMR (600 MHz, H_2O/D_2O 9:1, noesygppr1d, 1.8-6.0 ppm) spectra to show the reaction of $\mathbf{16_G}$ (50 mM) and $\mathbf{2_{EX}}$ (50 mM) with MSM (25 mM; internal standard) in phosphate buffer (pH 7, 500 mM) at room temperature to selectively yield **10r**.

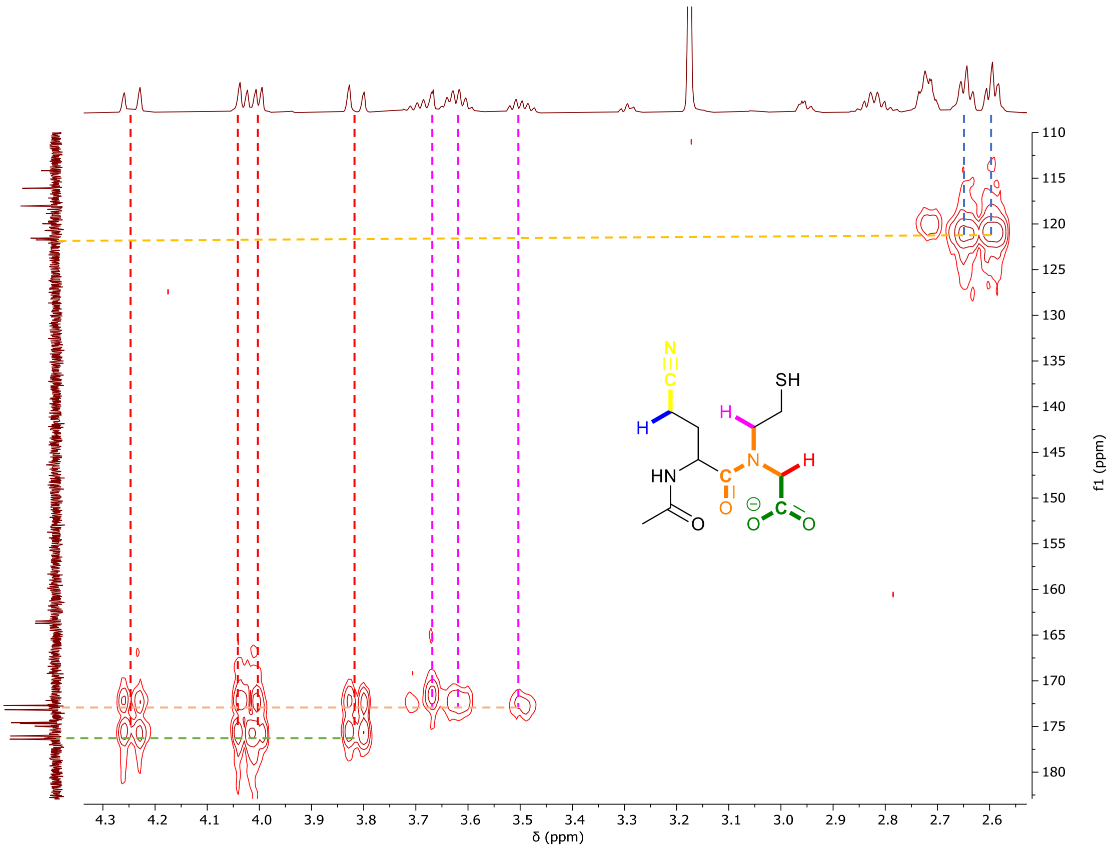

Figure S140: ^{1}H– ^{13}C HMBC (^{1}H: 600 MHz [2.5-4.3 ppm], ^{13}C: 150 MHz [110-185 ppm]) spectrum to show the diagnostic $^{3}J_{CH}$ and $^{2}J_{CH}$ coupling of α-Gly and *N*-CH_2 to tertiary amide carbon and the diagnostic $^{2}J_{CH}$ coupling of γ-Glx to γ-CN carbon at pH 7, that is characteristic of **10r**.

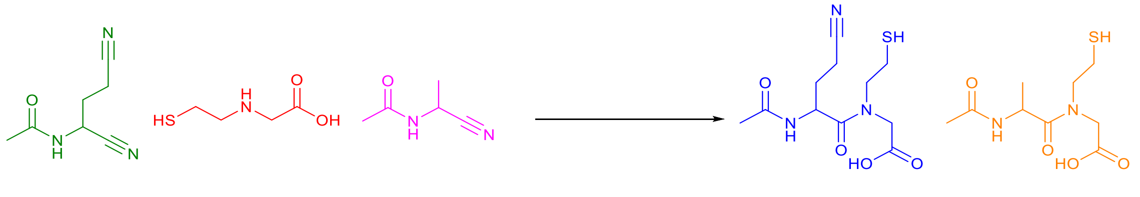

2_{EX} 16_G 2_A 10r 10d

A solution of **16$_G$** (50 mM), Ac-Glx-CN **2$_{EX}$** (50 mM), Ac-Ala-CN **2$_A$** (50 mM) and MSM (25 mM; internal standard) in phosphate buffer (1 mL, pH 7, 500 mM in H_2O/D_2O (9:1)) was incubated for 24 hours at room temperature. The reaction was then analysed by NMR spectroscopy and observed to yield mixture of **10r** (80%) and **10d** (20%).

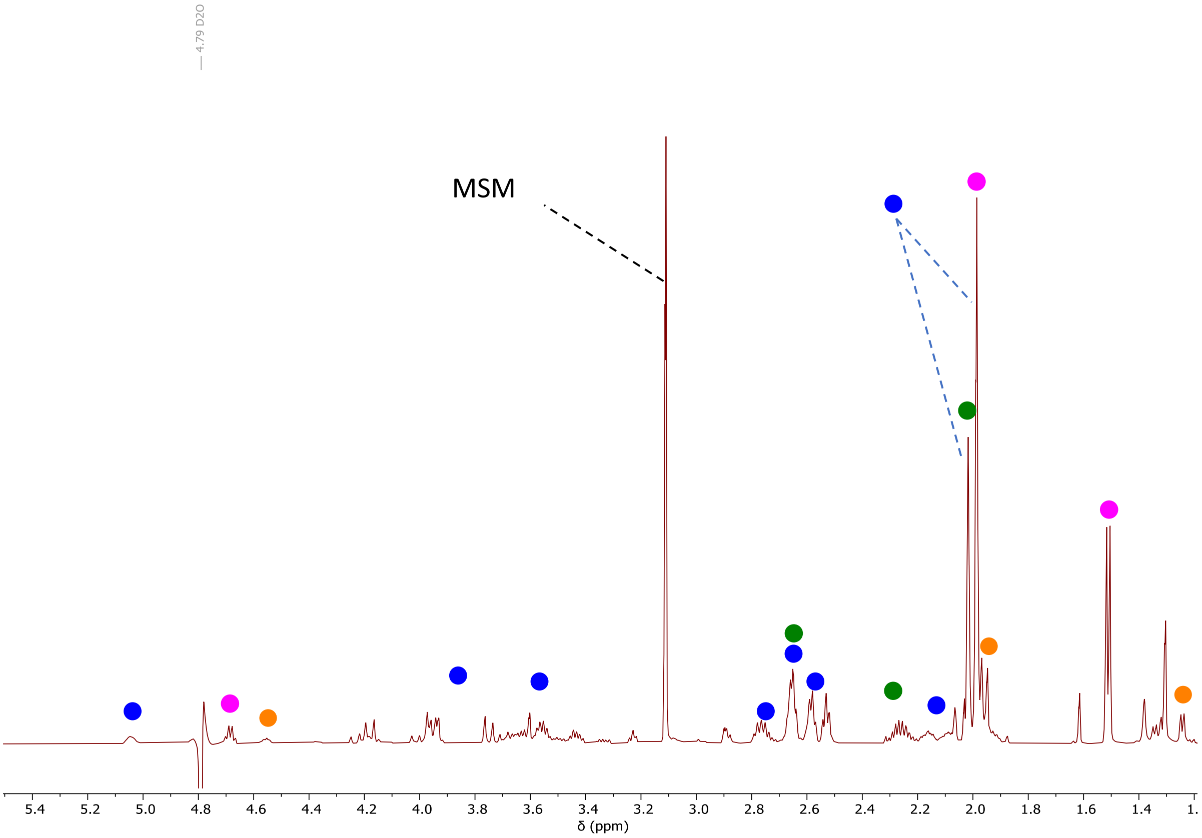

Figure S141: ^1H NMR (600 MHz, H_2O/D_2O 9:1, noesygppr1d, 1.3-5.4 ppm) spectrum to show the competition reaction of **16$_G$** (50 mM), **2$_{EX}$** (50 mM) and **2$_A$** (50 mM) with MSM (25 mM; internal standard) in phosphate buffer (pH 7, 500 mM) at room temperature to yield a mixture of **10r** and **10d**.

Reductive fragmentation:

Reductive fragmentation of **31a** to yield **11_{GG}**:

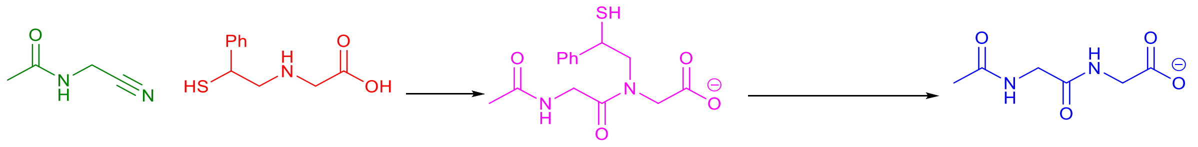

2_G **30_G** **31a** **11_{GG}**

A solution of **30_G** (25 mM) and **2_G** (25 mM) was incubated at pH 9.2 in H_2O (2 mL) at room temperature for 48 hours to yield **31a**. TCEP (20 mM) and **31a** (0.5 mM) in H_2O were then incubated at pH 8.5 and 55 °C for 23 hours. The solution was then lyophilised. The white lyophilizate was dissolved in H_2O/D_2O (9:1, 2 mL) with MSM (12.5 mM) and NMR spectra were acquired. Peptide **11_{GG}** (60% yield over 2 steps) was observed. The identity of **11_{GG}** was confirmed by spiking with authentic synthetic standard.[1,2]

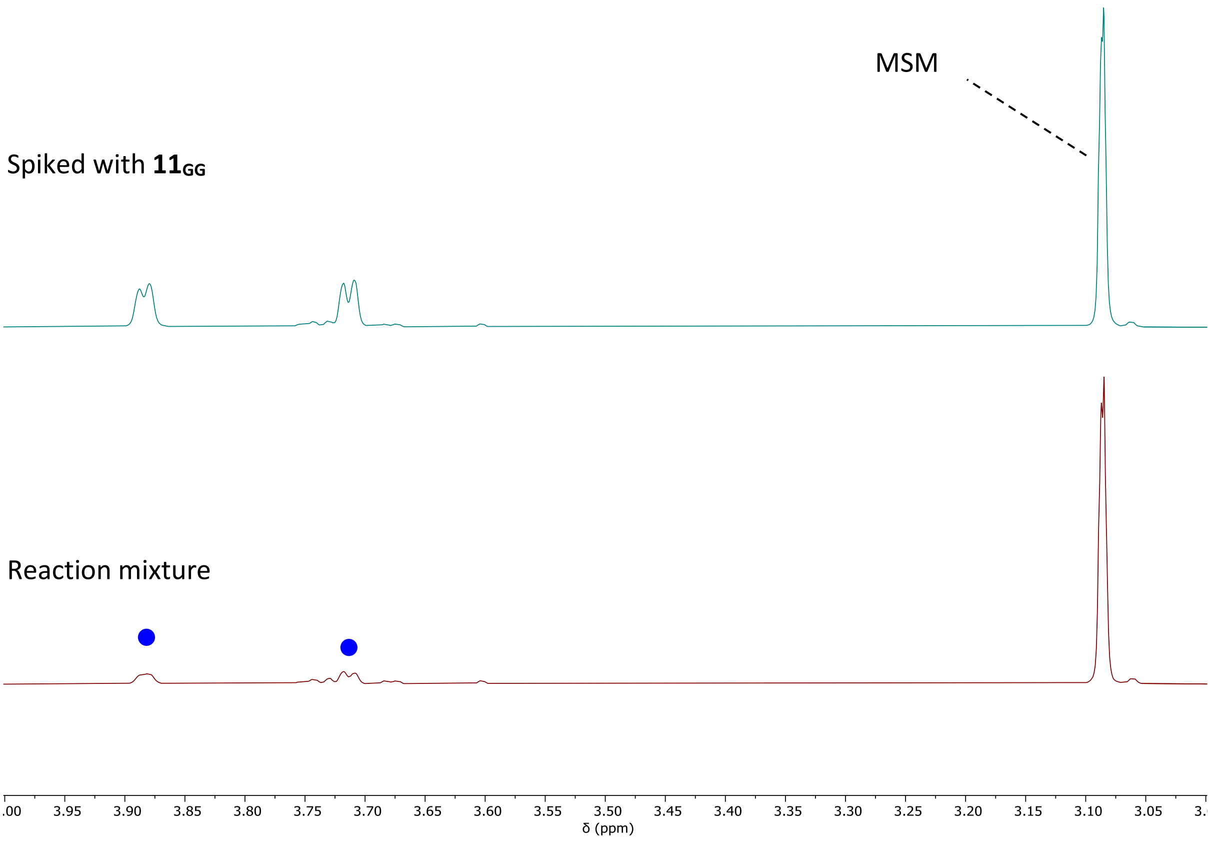

Figure S142: ^{1}H NMR (600 MHz, H_2O, noesygppr1d, 3.0-4.0 ppm) spectra to show the reaction of **31a** (0.5 mM) and TCEP (20 mM) at pH 8.5 and 50 ^oC for 23 hours, which was observed to yield peptide **11_{GG}** (Ac-Gly-Gly-OH).

Reductive fragmentation of **10a** to yield **11$_{GG}$**:

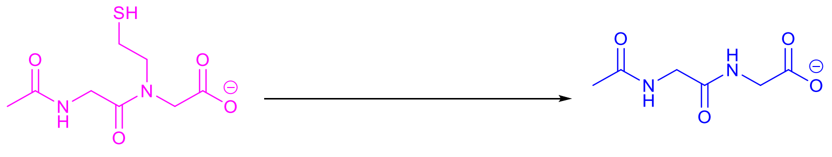

10a **11$_{GG}$**

Peptoid **10a** (0.5 mM) and TCEP (100 mM) in H_2O (25 mL) were incubated at pH 8.5 and 50 °C for 22 hours. The solution was then lyophilised. The white lyophilizate and MSM (12.5 mM) were dissolved in H_2O/D_2O (2 mL, 9:1). The solution was analysed by NMR spectroscopy and peptide **11$_{GG}$** (70%) was observed. The identity of **11$_{GG}$** was confirmed by spiking with authentic synthetic standard.[1,2]

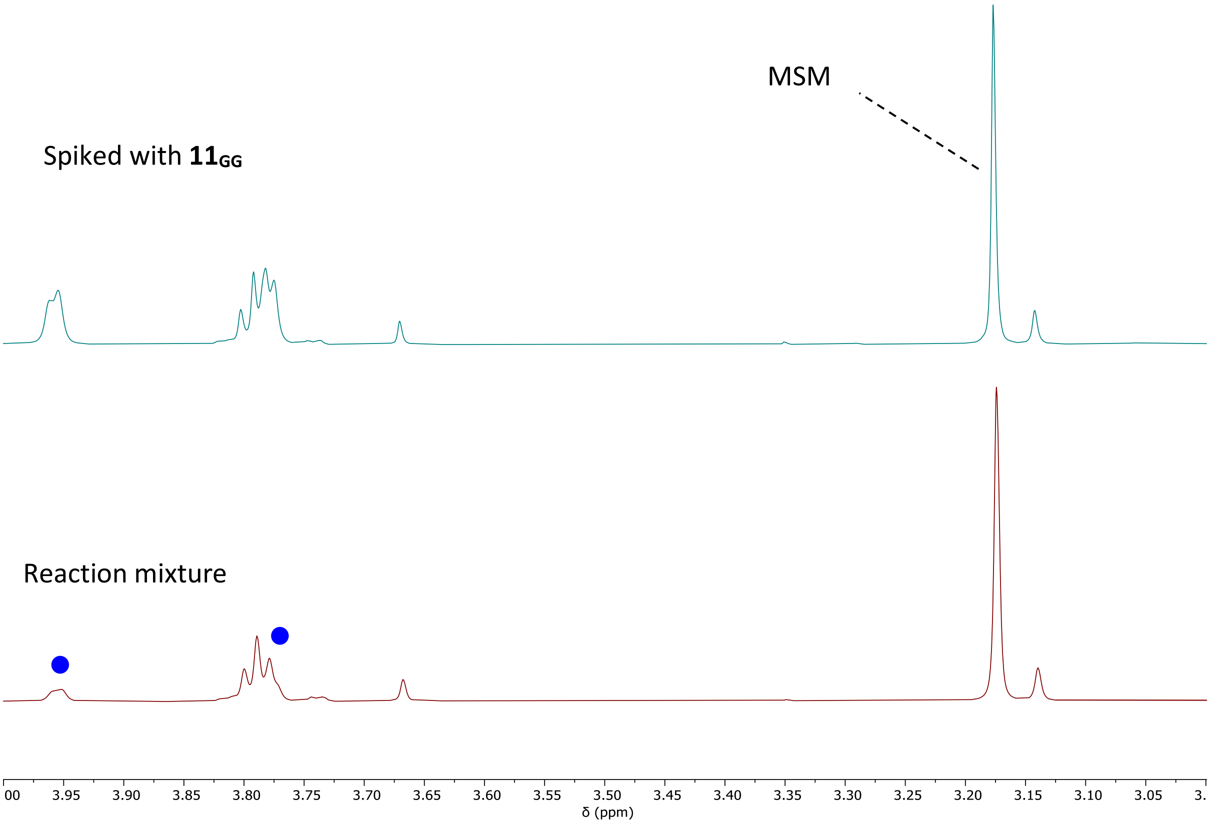

Figure S143: ^{1}H NMR (600 MHz, H_2O/D_2O 9:1, noesygppr1d, 3.0-4.0 ppm) spectra to show the reaction of **10a** (0.5 mM) and TCEP (100 mM) at pH 8.5 and 50 °C for 22 hours, which was observed to yield peptide **11$_{GG}$** (Ac-Gly-Gly-OH).

Preparative synthesis:

Synthesis of 16_{AA}:

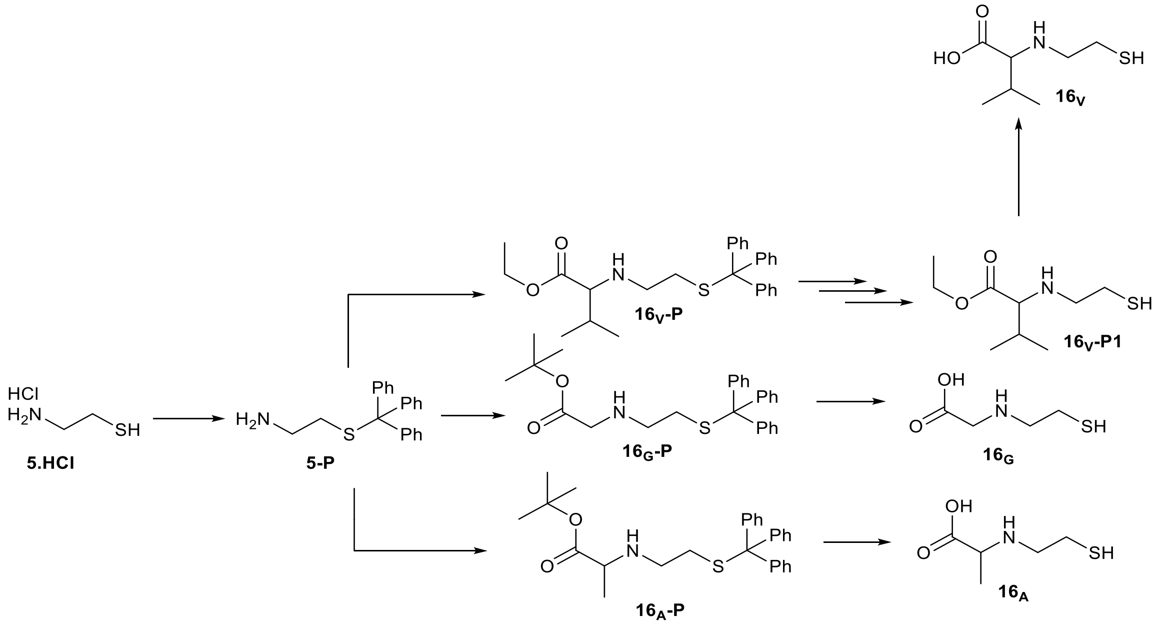

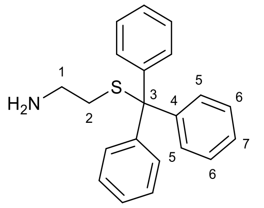

Synthesis of S-trityl cysteamine **5-P**: To cysteamine hydrochloride **5**.HCl (2.55 g, 33.05 mmol, 1 equiv.) in trifluoroacetic acid (30 mL) was added trityl chloride (9.21 g, 33.05 mmol, 1 equiv.). Upon complete addition the solution, which went blood-red, was left to stir at room temperature for 3 hours. The reaction mixture was concentrated *in vacuo* before the addition of EtOAc (50 mL). The organics were separated and then washed with NaOH (4 × 20 mL, 3 M), H_2O (20 mL), $NaHCO_3$ (2 × 20 mL, sat. aqueous solution) and brine (3 × 20 mL). The organics were dried over $MgSO_4$ and concentrated in vacuo to yield **5-P** as a pale-yellow solid (10.07 g, 95%). **5-P** was used without further purifications.[3]

Compound 5-P: ^{1}H NMR (400 MHz, CDCl$_3$): δ_H 1.63 (broad s, 2H, N-H), 2.34 (t, *J* = 4.0 Hz, 2H, (C2)-H), 2.58 (t, *J* = 4.0 Hz, 2H, (C1)-H), 7.21 (m, 3H, (C7)-H), 7.29 (m, 6H, (C6)-H), 7.435 (m, 6H, (C5)-H). **^{13}C NMR (100 MHz, CDCl$_3$):** δ_C 36.1 (C2), 41.1 (C1), 66.7 (C3), 126.7 (C7), 128.0 (C6), 129.7 (C5), 145.0 (C4). **IR (solid):** 3054.12, 3018.15, 2924.94, 1592.69, 1485.09, 1440.78, 1180.26, 1077.27, 1032.45, 849.88, 741.35, 696.89, 674.11, 624.14, 505.91. **HRMS (ESI$^+$):** Calcd. for $[C_{21}H_{21}NS+H]^+$ = 320.1467; observed 320.1467.

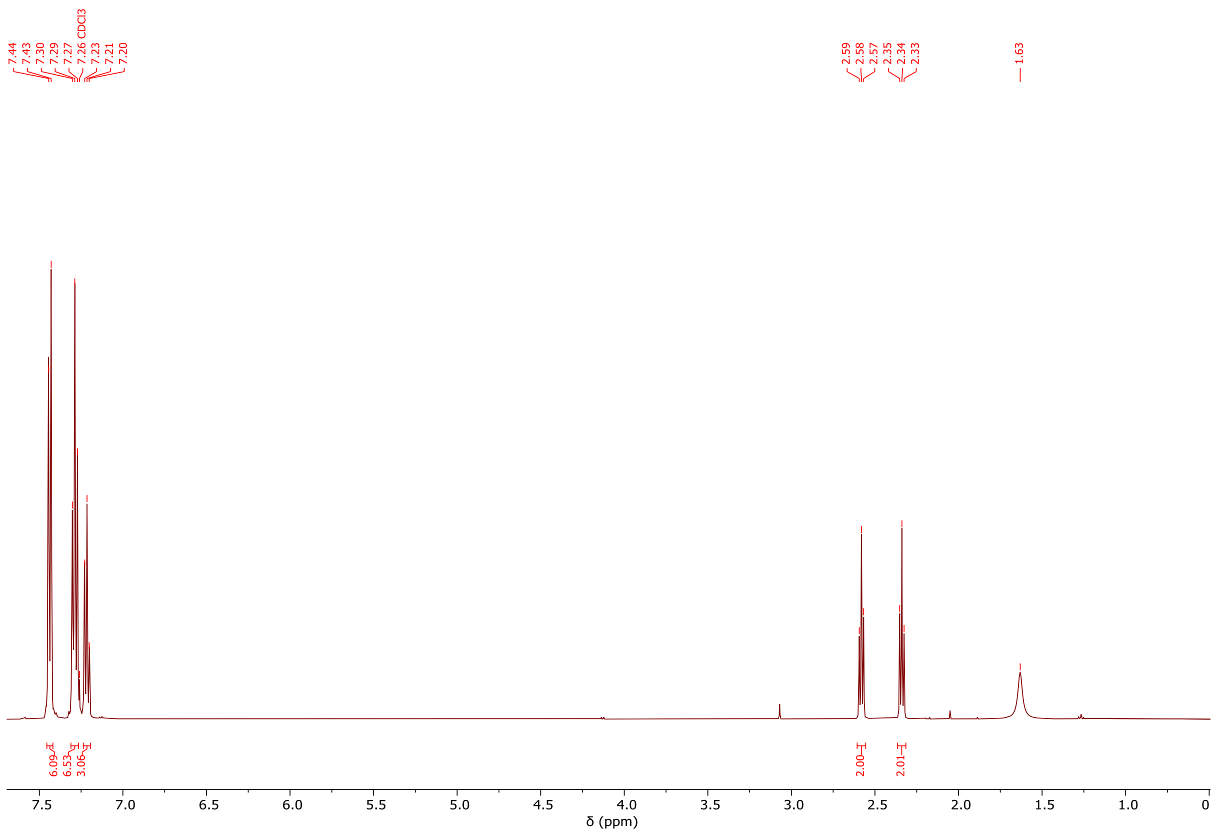

Figure S144: ^{1}H NMR (400 Hz, $CDCl_3$, 0.0-7.8 ppm) spectrum to show *S*-trityl cysteamine (**5-P**).

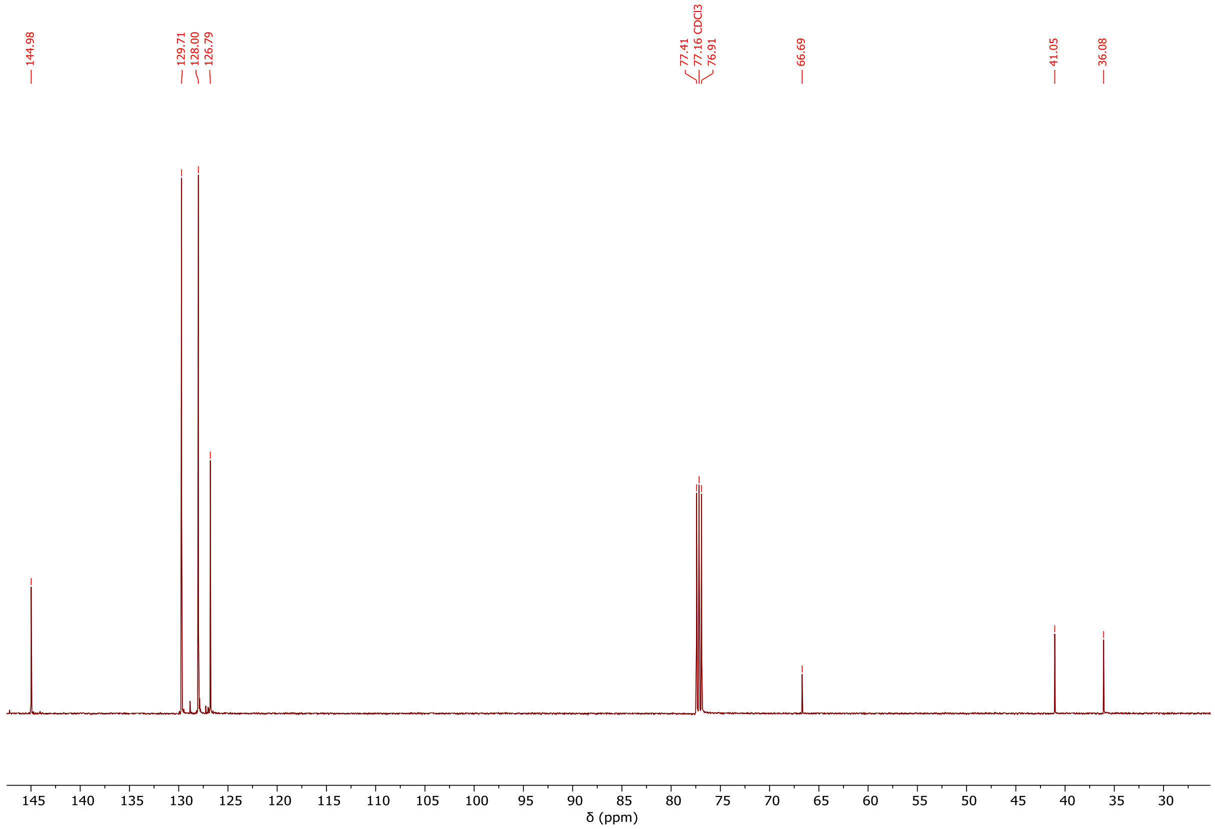

Figure S145: ^{13}C NMR (100 Hz, $CDCl_3$, 25-145 ppm) spectrum to show *S*-trityl cysteamine (**5-P**).

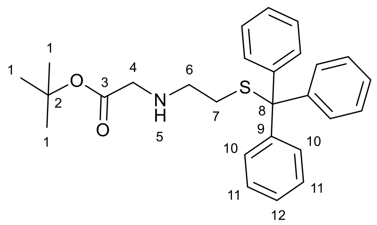

Synthesis of **16_G-P**: A solution of t-butyl bromoacetate (1.43 mL, 9.68 mmol, 1 equiv.) in anhydrous tetrahydrofuran (12.5 mL) was added dropwise to a stirred solution of *S*-trityl cysteamine **5-P** (3.09 g, 9.68 mmol, 1 equiv.) and NEt_3 (2.70 mL, 19.36 mmol, 2 equiv.) in anhydrous tetrahydrofuran (12.5 mL) over 30 mins. The solution was then stirred at room temperature and monitored by TLC. After 4 hours, the reaction was judged to have reached completion. The reaction was concentrated *in vacuo* before purification with flash column chromatography (SiO_2; eluting with a gradient of 40-60 petroleum ether/EtOAc/NEt_3 100:00:1 → 75:25:1) to afford **16_G-P** (2.82 g, 6.50 mmol, 67%) as a viscous, colourless oil.

Compound 16_G-P: Rf (Hexane/EtOAc/NEt_3 75:25:1) = 0.19. **^{1}H NMR (400 MHz, $CDCl_3$):** δ_H 1.43 (s, 9H, (C1)-H), 1.78 (broad s, 1H, (C5)-H), 2.33 (t, *J* = 8.0 Hz, 2H, (C7)-H), 2.54 (t, *J* = 8.0 Hz, 2H, (C6)-H), 3.14 (s, 2H, (C4)-H), 7.19 (m, 3H, (C12)-H), 7.26 (m, 6H, (C11)-H), 7.41 (m, 6H, (C10)-H). **^{13}C NMR (150 MHz, $CDCl_3$):** δ_C 28.2 (C1), 32.3 (C7), 48.1 (C6), 51.4 (C4), 66.7 (C8), 81.4 (C2), 126.8 (C12), 128.0 (C11), 129.7 (C10), 145.0 (C9), 171.5 (C3). **IR (oil):** 3063.90, 2977.33, 2924.38, 1724.26, 1593.84, 1487.53, 1441.38, 1366.24, 1228.28, 1144.16, 1000.86, 923.43, 846.45, 764.86, 741.32, 696.37, 675.76, 623.29, 506.98. **HRMS (ESI$^+$):** Calcd. for $[C_{27}H_{31}NO_2S+H]^+$ = 434.2148; observed 434.2141

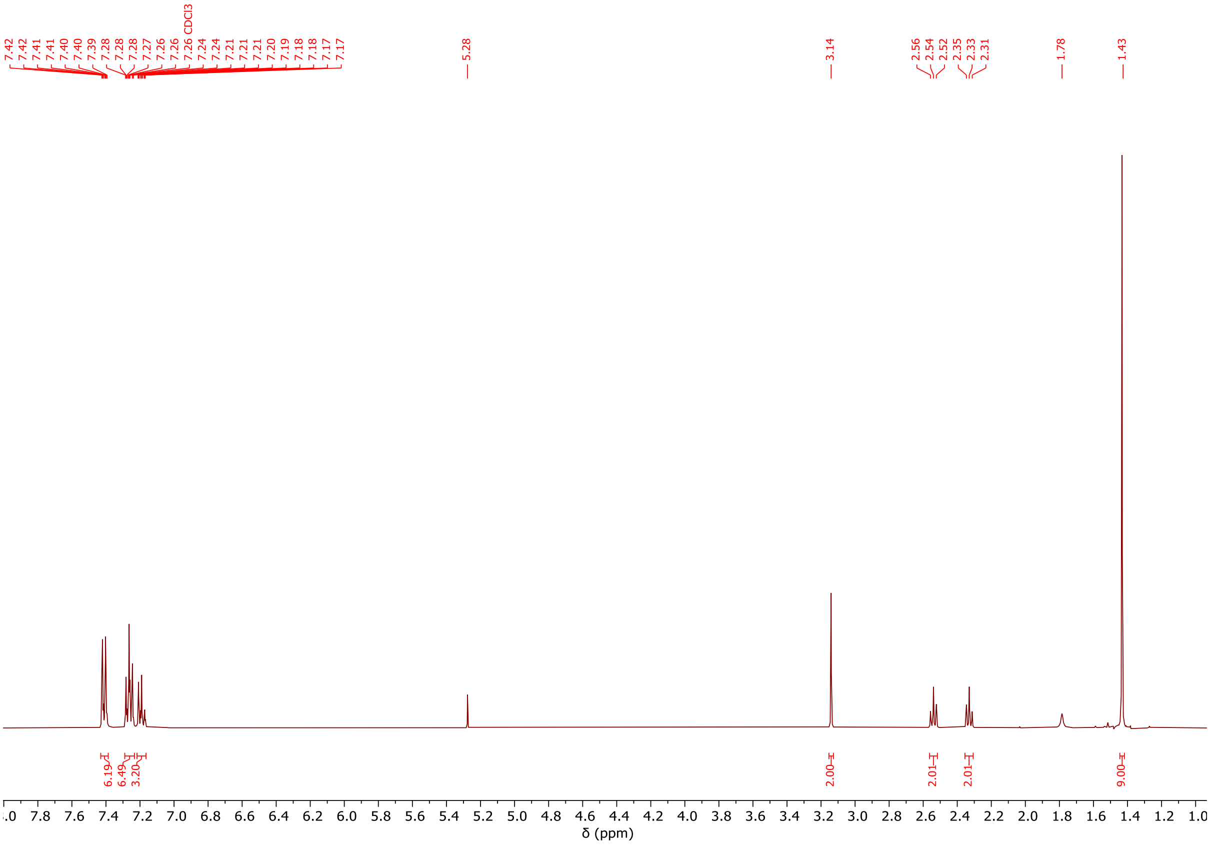

Figure S146: ^{1}H NMR (400 Hz, $CDCl_3$, 1.0-8.0 ppm) spectrum to show **16_G-P**.

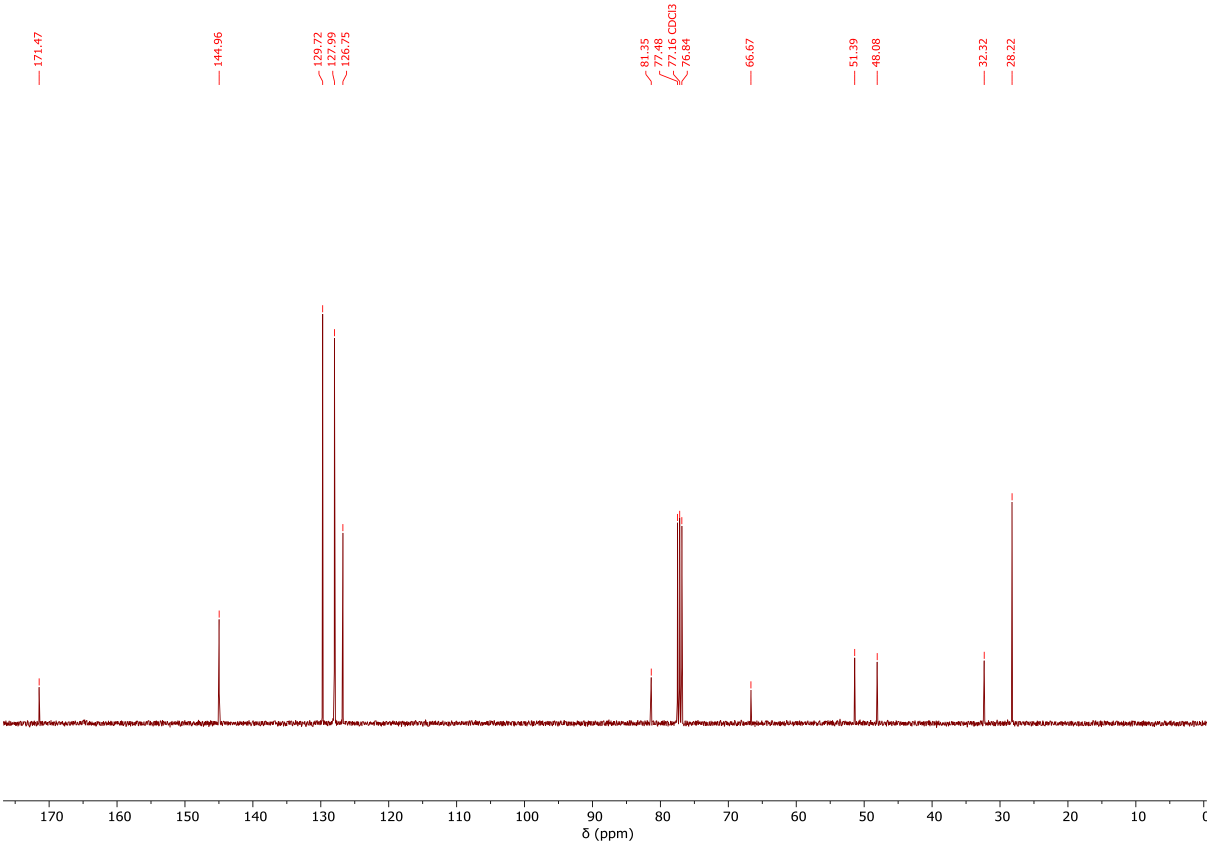

Figure S147: ^{13}C NMR (150 Hz, $CDCl_3$, 0.0-175 ppm) spectrum to show **16_G-P**.

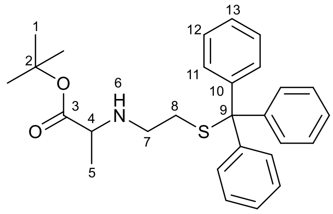

Synthesis of **16_A-P**: A solution of racemic *tert*-butyl-2-bromopropanoate (0.87 mL, 5.22 mmol, 1 equiv.) in anhydrous tetrahydrofuran (12.5 mL) was added dropwise to a stirred solution of *S*-trityl cysteamine **5-P** (2.00 g, 6.26 mmol, 1.2 equiv.) and NEt_3 (1.45 mL, 10.43 mmol, 2 equiv.) in anhydrous tetrahydrofuran (12.5 mL). The resultant solution was then heated at 50°C for 24 hours. After cooling to room temperature, the suspension was filtered and concentrated *in vacuo* before purification with flash column chromatography (SiO_2; n-hexane/diethyl ether: 100:00 → 50:50) to yield **16_A-P** as a viscous, pale-yellow oil (1.55 g, 3.46 mmol, 66%).

Compound 16_A-P: Rf (40-60 petroleum ether/diethyl ether 8:2) = 0.22. **^{1}H NMR (700 MHz, MeOD-d4):** δ_H 1.14 (d, *J* = 7.0 Hz, 3H, (C5)-H), 1.44 (s, 9H, (C1)-H), 2.31-2.44 (m, 4H, (C7)-H & (C8)-H), 3.01 (q, *J* = 7.0 Hz, 1H, (C4)-H), 7.21 (app t, *J* = 7.4 Hz, 3H, (C13)-H), 7.28 (app t, *J* = 7.8 Hz, 6H, (C12)-H), 7.40 (app d, *J* = 8.0 Hz, 6H, (C11)-H).**^{13}C NMR (175 MHz, MeOD-d4):** δ_C 18.6 (C5), 28.3 (C1), 32.7 & 47.4 (C7 & C8), 57.9 (C4), 67.9 (C9), 82.4 (C2), 127.8 (C13), 128.9 (C12), 130.8 (C11), 146.2 (10), 175.6 (C3). **IR (oil):** 3056.63, 2974.95, 2929.08, 1725.03, 1594.83, 1487.76, 1443.67, 1391.60, 1366.63, 1253.44, 1212.93, 1143.63, 1079.21, 1033.70, 847.86. **HRMS (ESI$^+$):** Calcd. for $[C_{28}H_{33}NO_2S+H]^+$ = 448.2305; observed 448.2296.

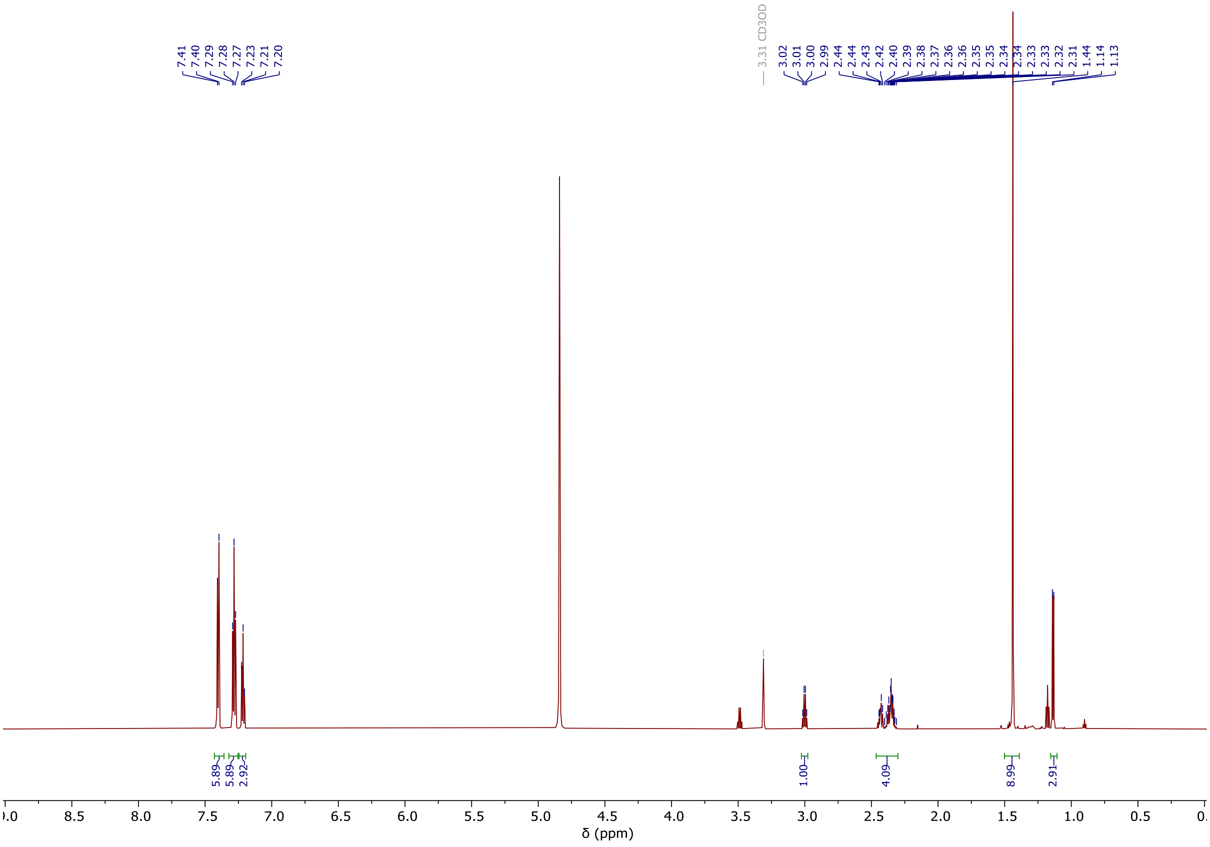

Figure S148: ^{1}H NMR (700 Hz, MeOD-d4, 0.0-9.0 ppm) spectrum to show **16_A-P**.

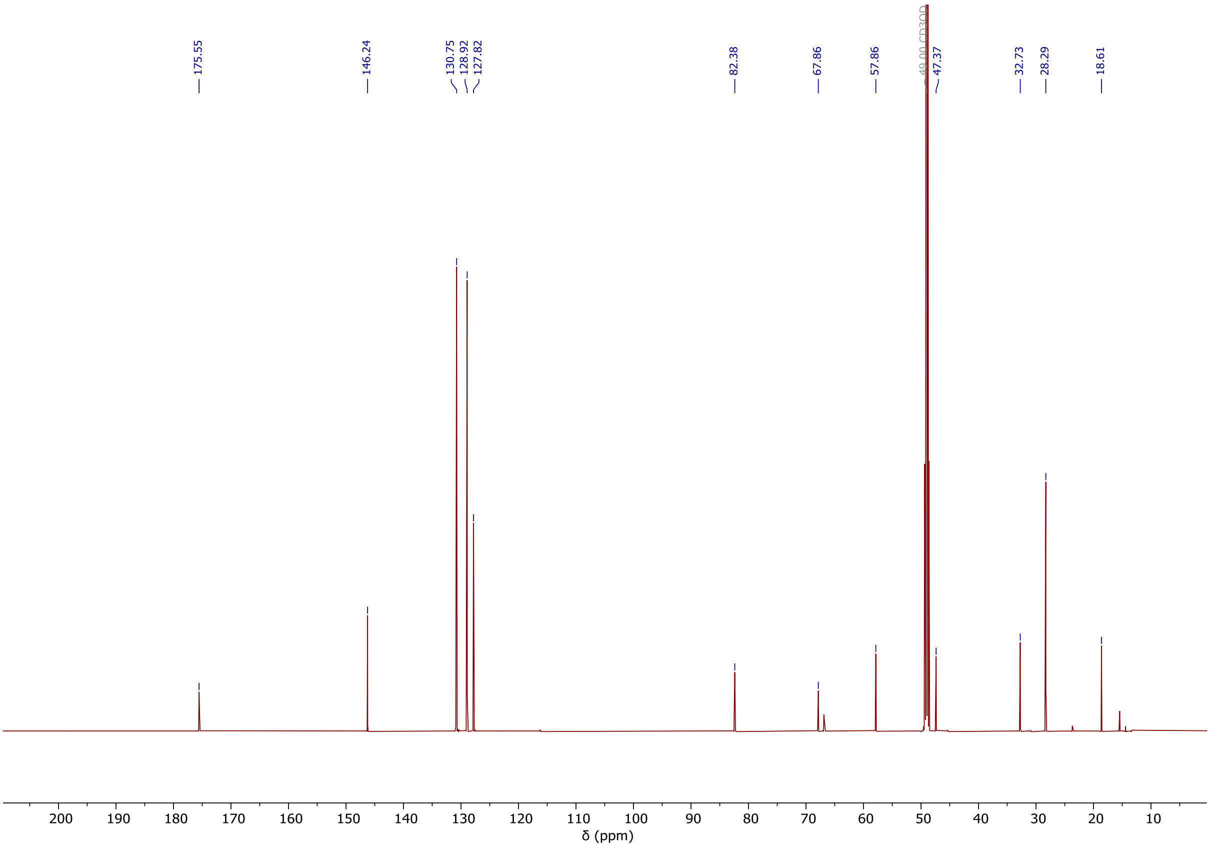

Figure S149: ^{13}C NMR (175 Hz, MeOD-d4, 0.0-210 ppm) spectrum to show **16_A-P**.

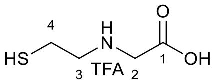

Synthesis of **16_G**·TFA: To a solution of **16_G-P** (2.91 g, 6.92 mmol, 1 equiv.) and triisopropylsilane (13.8 mL, 67.18 mmol, 10 equiv.) in anhydrous CH_2Cl_2 (10 mL) was added trifluoroacetic acid (25 mL). The solution immediately turned yellow and then became colourless within 30 seconds. The reaction mixture was stirred at room temperature for 2 hours and then concentrated *in vacuo*. The residue was triturated with Et_2O (3 × 50 mL) to yield **16_G**·TFA (1.59 g, 6.38mmol, 92%) as white powder.

Compound 16_G·TFA: **^{1}H NMR (700 MHz, D_2O):** δ_H 2.89 (t, *J* = 7.0 Hz, 2H, (C4)-H), 3.34 (t, *J* = 7.0 Hz, 2H, (C3)-H), 3.95 (s, 2H, (C2)-H).**^{13}C NMR (175 MHz, D_2O):** δ_C 20.4 (C4), 48.0 (C3), 50.3 (C2), 116.9 (q, C5), 163.5 (C6), 169.7 (C1).**^{19}F NMR (282 MHz, D_2O):** δ_F -75.71. **IR (solid):** 3012.91, 2976.23, 2824.02, 2425.35, 1949.32, 1720.62, 1658.96, 1587.15, 1463.71, 1432.12, 1343.47, 1267.91, 1244.25, 1178.58, 1138.43, 1075.91, 1010.26, 905.33, 834.97, 793.12, 721.29, 666.64, 602.32, 518.63, 448.17. **MP:** 101-111°C. **HRMS (ESI$^+$):** Calcd. for $[C_4H_9NO_2S+H]^+$ = 136.0427; observed 136.0426.

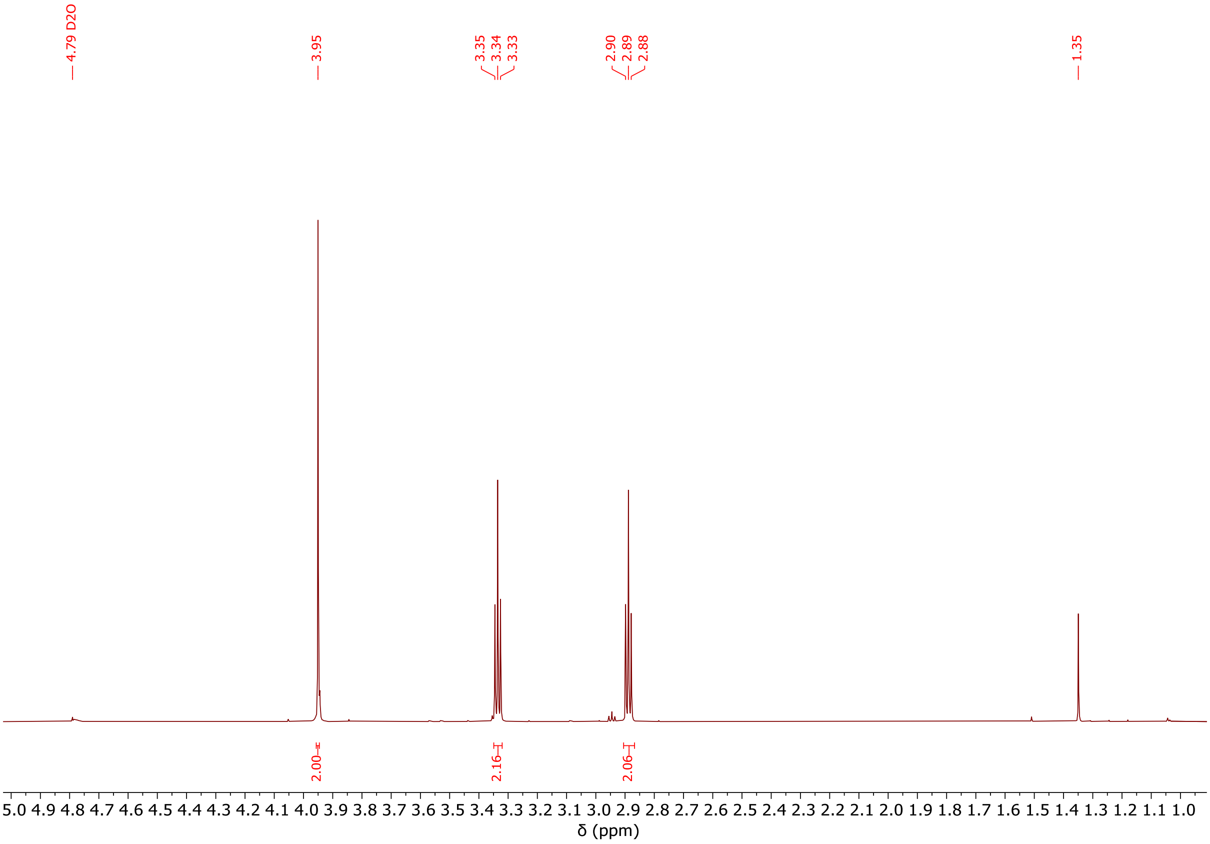

Figure S150: ^{1}H NMR (700 Hz, D_2O, 0.0-5.0 ppm) spectrum to show **16_G·**TFA.

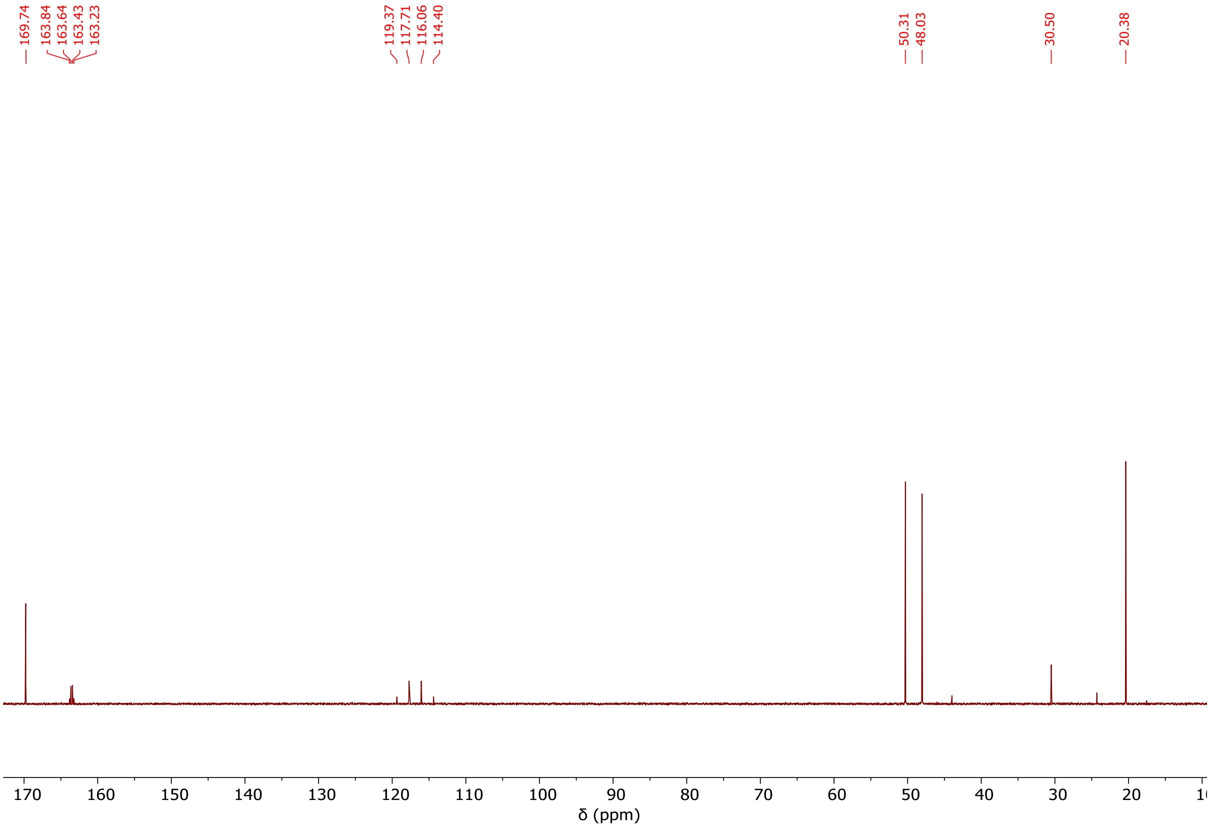

Figure S151: ^{13}C NMR (175 Hz, D_2O, 10-170 ppm) spectrum to show **16$_G$**·TFA.

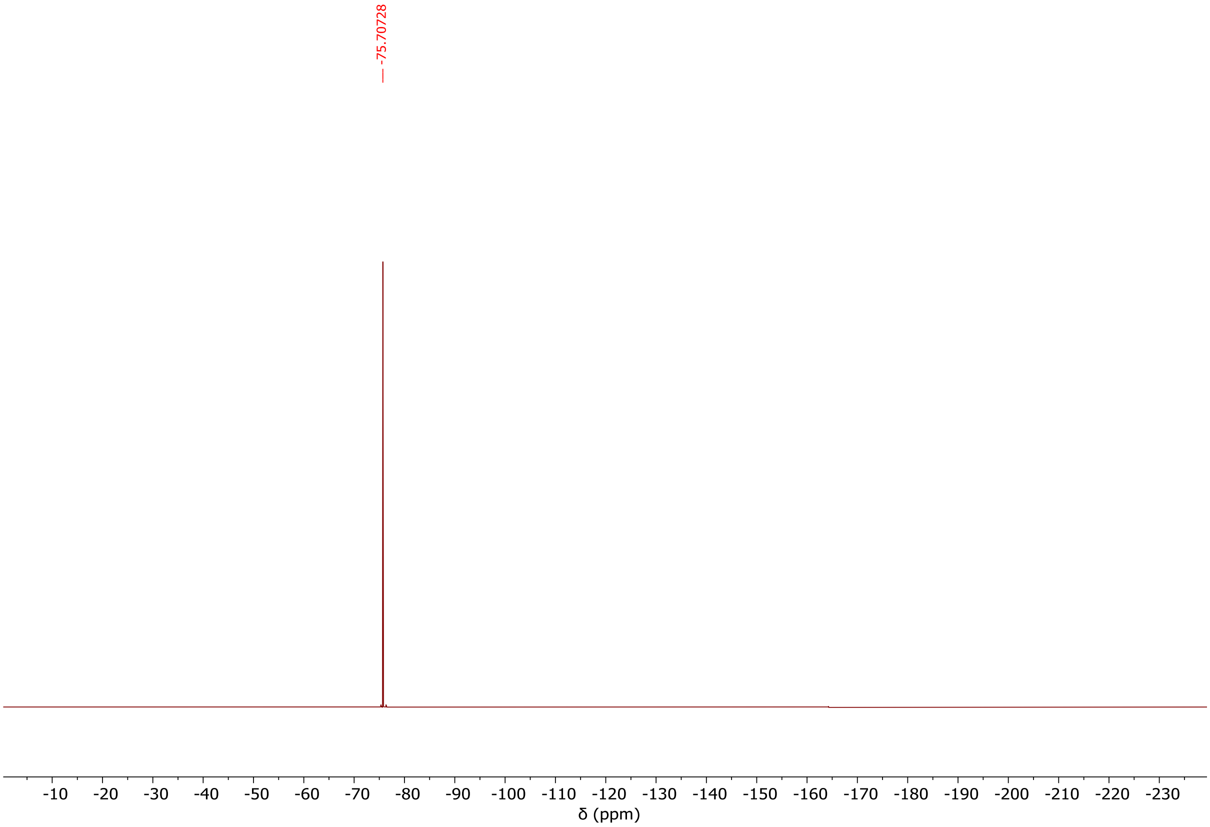

Figure S152: ^{19}F NMR (282 Hz, D_2O, -240-0.0 ppm) spectrum to show **16$_G$**·TFA.

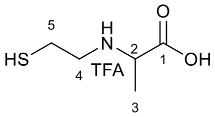

Synthesis of **16_A**·TFA: To a solution of **16_A-P** (0.95 g, 2.12 mmol, 1 equiv.) and triethylsilane (3.4 mL, 21.22 mmol, 10 equiv.) in anhydrous CH_2Cl_2 (10 mL) was added TFA (7.9 mL, 63.67 mmol, 30 equiv.). This solution was stirred at room temperature for 8 hours and then concentrating *in vacuo*. The residue was triturated with *n*-hexane (3 × 20 mL) to yield **16_A**·TFA (609 mg, 2.31 mmol) as a viscous, pale-yellow oil. **16_A**·TFA was found to be 85% pure against an MSM standard, therefore the yield was 93%.

Compound 16_A·TFA**: ^{1}H NMR (700 MHz, D_2O):** δ_H 1.61 (d, *J* = 7.3 Hz, 3H, (C3)-H), 2.89 (t, *J* = 6.9 Hz, 2H, (C5)-H), 3.34 (dtd, *J* = 19.6, 6.9, 12.7 Hz, 2H, (C4)-H), 4.12 (q, *J* = 7.3 Hz, 1H, (C2)-H). **^{13}C NMR (175 MHz, D_2O):** δ_C 14.6 (C3), 20.6 (C5), 48.9 (C4), 56.3 (C2), 172.7 (C1). **^{19}F NMR (282 MHz, D_2O):** δ_F -75.57. **IR (oil):** 2992.15, 2772.63, 2468.38, 1726.24, 1658.95, 1453.60, 1422.04, 1177.56, 1127.05, 990.40, 830.33, 795.69, 718.55, 517.13, 437.65, 411.26. **HRMS (ESI$^+$):** Calcd. for $[C_5H_{11}NO_2S+H]^+$ = 150.0583; observed 150.0582.

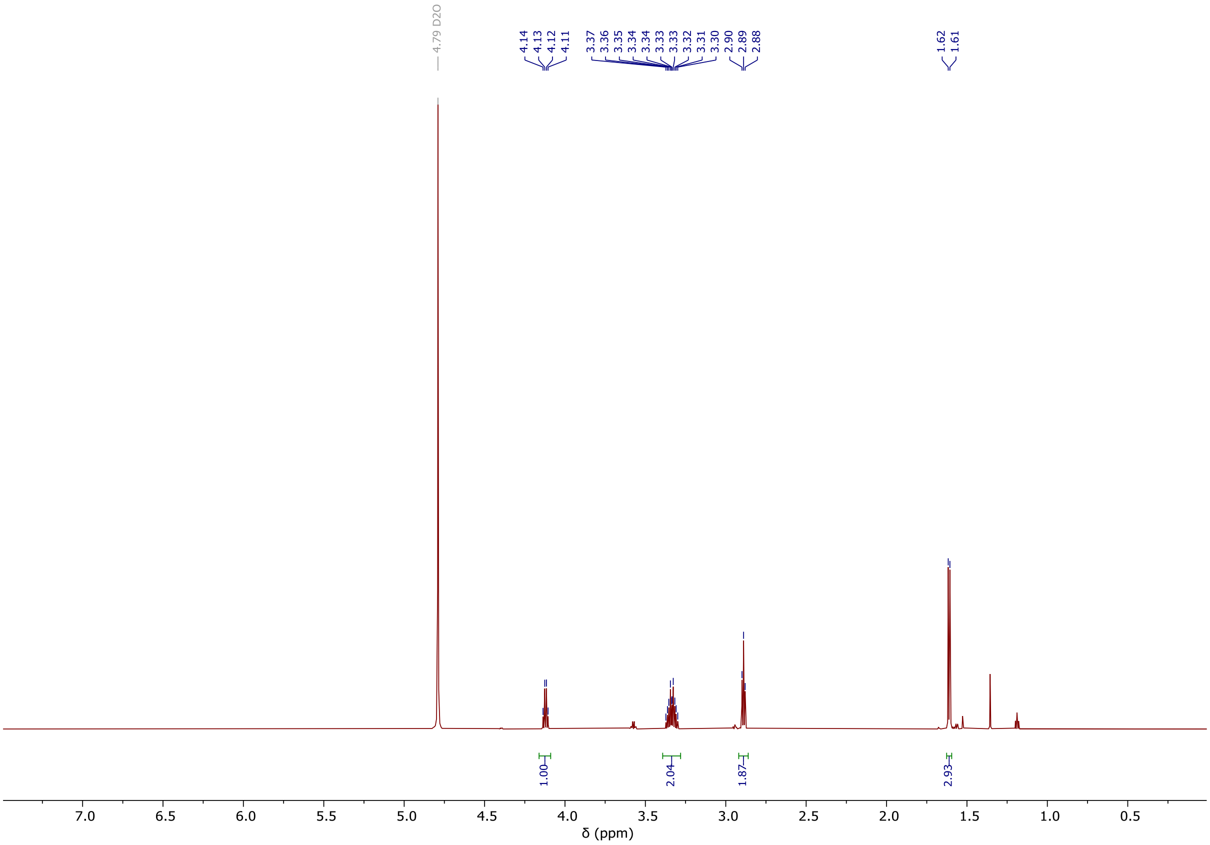

Figure S153: ^{1}H NMR (700 Hz, D_2O, 0.0-8.0 ppm) spectrum to show **16_A**·TFA.

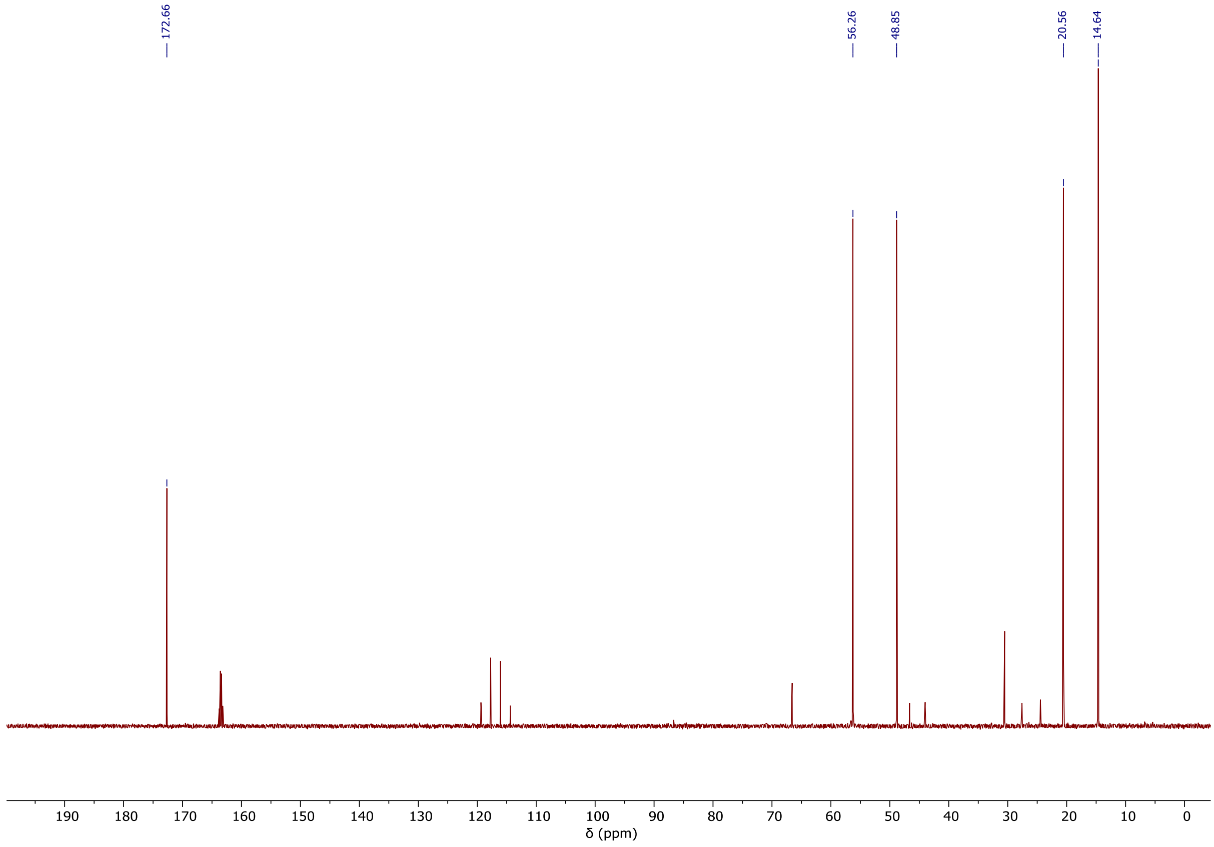

Figure S154: ^{13}C NMR (175 Hz, D_2O, 0.0-200 ppm) spectrum to show **16_A**·TFA.

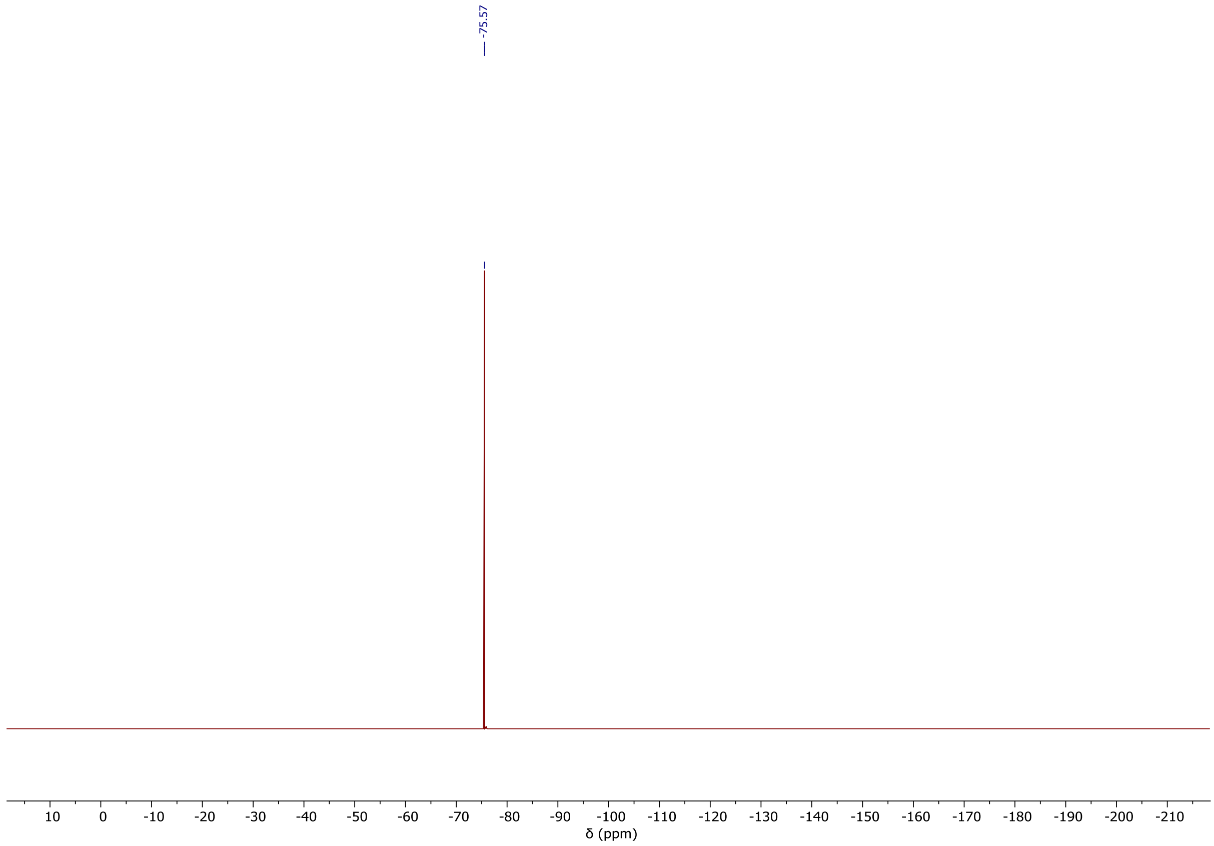

Figure S155: ^{19}F NMR (282 Hz, D_2O, -220-20 ppm) spectrum to show **16_A**·TFA.

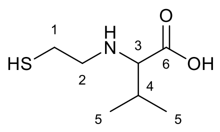

A solution of *S*-trityl cysteamine **5-P** (1.00 g, 3.13 mmol, 1 equiv.), racemic ethyl 2-bromoisovalerate (0.51 mL, 3.13 mmol, 1 equiv.) and NEt_3 (0.87 mL, 6.26 mmol, 2 equiv.) in anhydrous tetrahydrofuran (10 mL) was heated at 70 °C for an hour and then (refluxed) at 80 °C for 48 hours. The solution was then cooled to room temperature, silica added and concentrated *in vacuo*. The residue (with silica) was loaded onto a column and purified via flash column chromatography (SiO_2; eluting with a gradient of *n*-hexane/Et_2O 100:00 → 50:50) to yield **16_V-P** (331 mg, 0.74 mmol, 24%). **16_V-P** was redissolved in CH_2Cl_2 (5 mL). NEt_3 (0.31 mL, 2.21 mmol) and $(Boc)_2O$ (323 mg, 1.48 mmol) were then added, and this solution was stirred at room temperature. After 22 hours, the solution was concentrating *in vacuo*. The residue was purified by flash column chromatography (SiO_2; eluting with a gradient of n-Hexane/Et_2O 100:00 → 00:100). The product was dissolved in EtOH (3 mL), THF (2 mL) and aqueous NaOH (3M, 0.44 mL, 1.32 mmol) and stirred at room temperature for 24 hours. The solution was then concentrated *in vacuo*. H_2O (20 mL) was added to the residue and the solution was adjusted with HCl (3 M) to pH 1.5. The aqueous solution was then extracted with a mixture of CH_2Cl_2/isopropanol. The organics were dried over $MgSO_4$, filtered and concentrated *in vacuo*. The residue was then purified by flash column chromatography (SiO_2; eluting with a gradient of CH_2Cl_2/MeOH 100:00 → 70:30). The product was dissolved in anhydrous CH_2Cl_2 (5 mL). Triethyl silane (0.65 mL, 4.06 mmol) was added to the product solution, followed by trifluoroacetic acid (1.51 mL, 12.18 mmol) and then the solution was stirred at room temperature for 3.5 hours. The solution was then concentrated *in vacuo*, and the residue dissolved in Et_2O (~15 mL). The addition of HCl (2 mL, 2 M in Et_2O) led to precipitation as a white solid, which was then triturated with Et_2O (3 × 20 mL), dissolved in H_2O (10 mL) and lyophilised. The product was dissolved in H_2O (1 mL) and the solution was adjusted to pH 12.5 with NaOH (2 M), and heated at 60°C for 5 hours. The solution was cooled to room temperature and lyophilised to yield a ratio of disulfide **35_V**/ethyl ester **16_V-P1** (95:5). The product was dissolved in H_2O (1.5 mL), an excess of TCEP was added and the solution was adjusted to pH 7.5. The concentration of **16_V** (135 mM) was then determined against an MSM internal standard by ^1H NMR spectroscopy.

Compound 16_V: ^1H NMR (400 MHz, D_2O): δ_H 1.04 (d, *J* = 7.0 Hz, 3H, (C5)-H), 1.09 (d, *J* = 7.1 Hz, 3H, (C5')-H), 2.31 (obs. M, 1H, (C4)-H), 2.91 (obs. m, 2H, (C1)-H), 3.29 (m, 2H, (C2)-H), 3.68 (d, *J* = 4.3 Hz, (C3)-H). **^{13}C NMR (100 MHz, D_2O):** δ_C 17.7 (C5), 18.7 (C5'), 20.4 (C1), 29.8 (C4), 50.6 (C2), 68.0 (C3), 172.4 (C6). **HRMS (ESI$^+$):** Calcd. for $[C_{14}H_{28}O_4N_2S_2+H]^+$ = 353.1563; observed 353.1563.

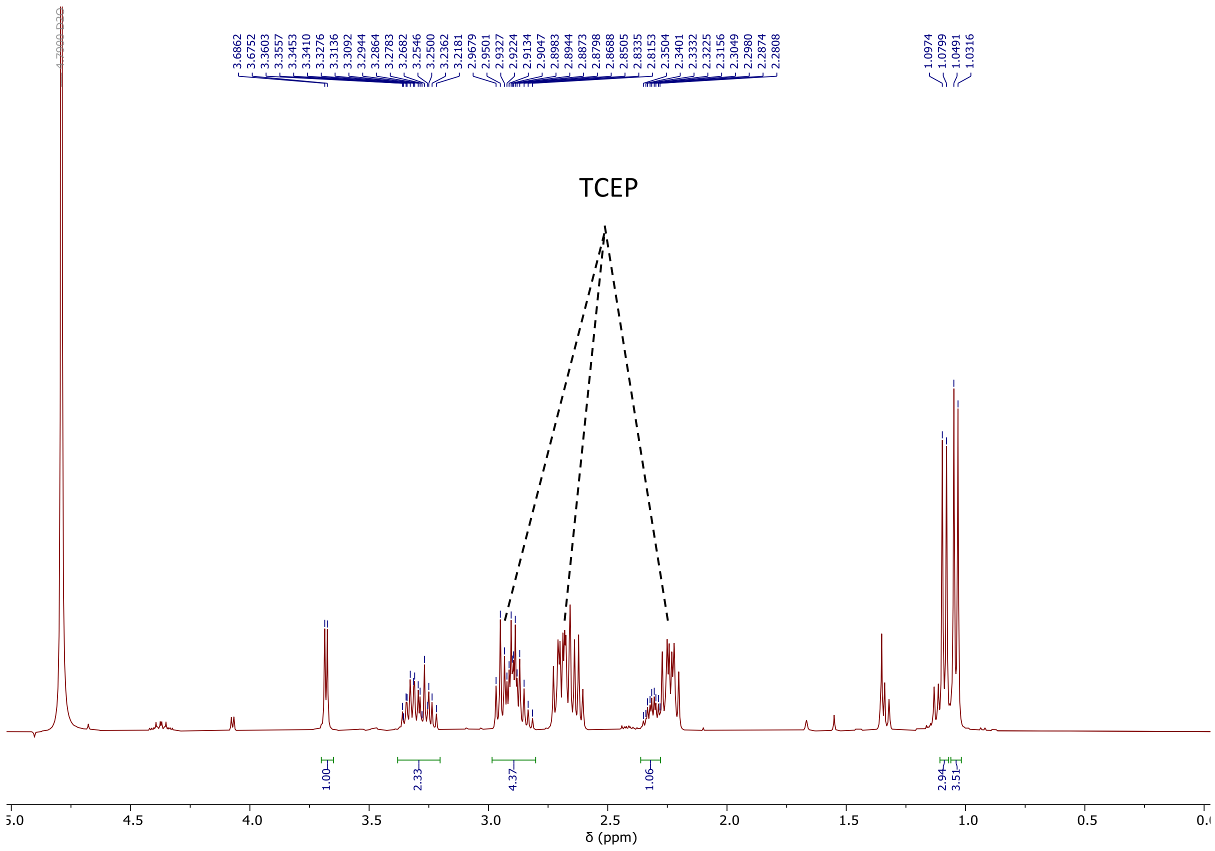

Figure S156: ^{1}H NMR (400 Hz, D_2O, 0.0-5.0 ppm) spectrum to show $\mathbf{16_V}$.

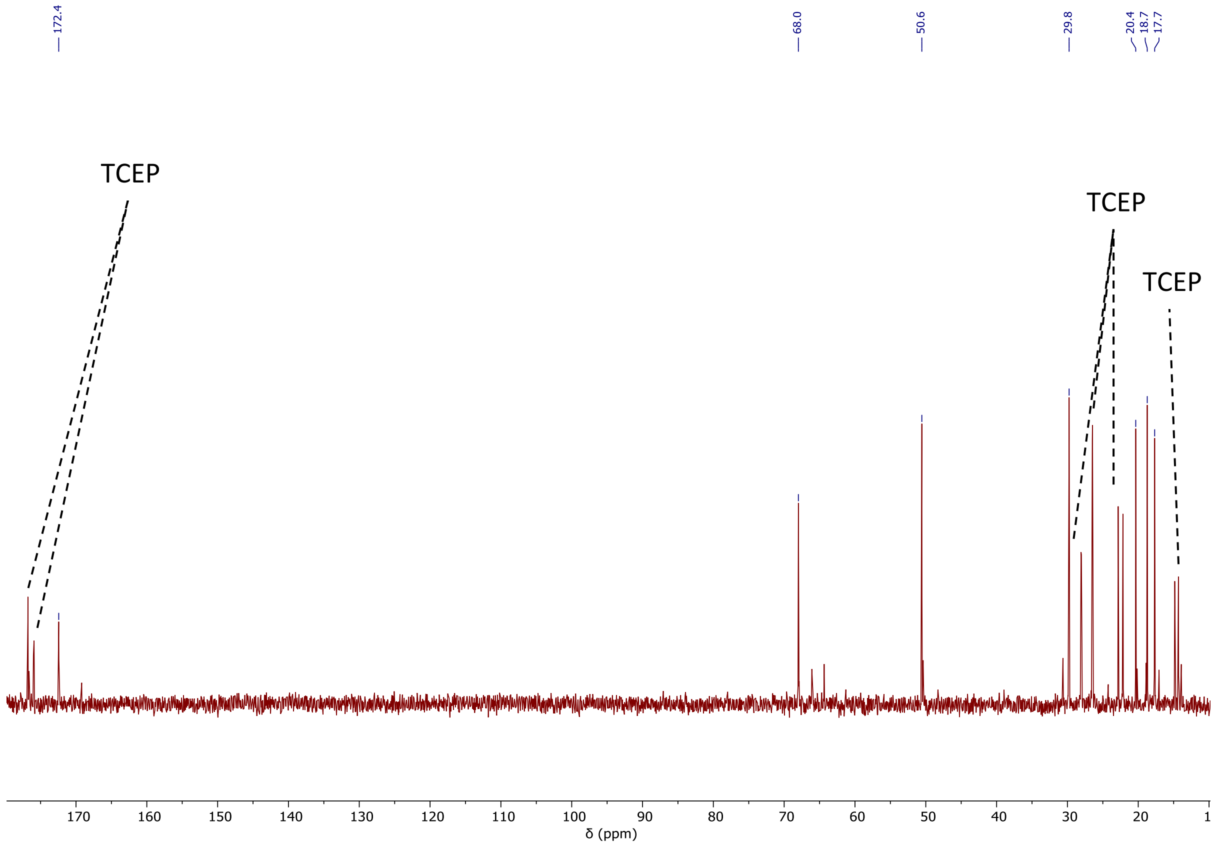

Figure S157: ^{13}C NMR (100 Hz, D_2O, 0.0-180 ppm) spectrum to show $\mathbf{16_V}$.

Synthesis of **17$_G$**:

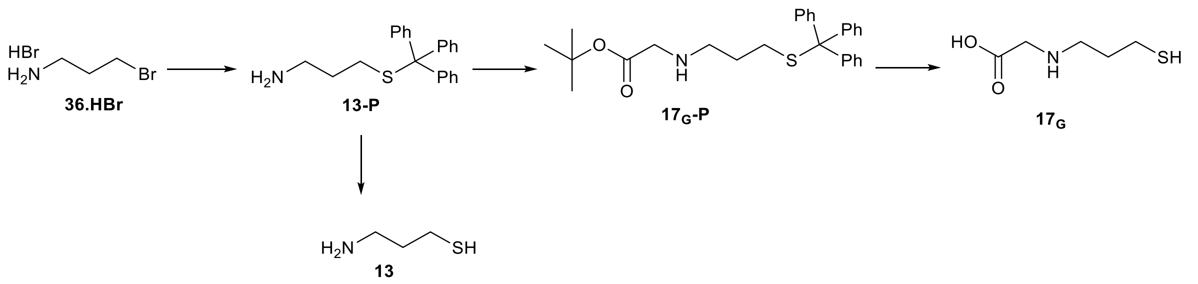

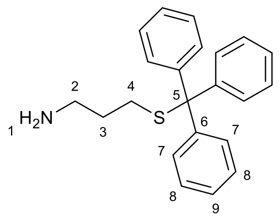

Synthesis of **13-P**: NaH (60% dispersion in mineral oil, 382 mg, 15.92 mmol, 2.2 equiv.) was washed with *n*-hexane (3 × 5 mL) under argon atmosphere in flame dried glassware and then cooled to 0 °C. To the solids was added triphenylmethanethiol (2.00 g, 7.24 mmol, 1 equiv.) in DMF (17.5 mL) and the reaction was stirred at 0 °C. After 10 minutes, 3-bromopropanamine hydrobromide **36**·HBr (1.58 g, 7.24 mmol, 1 equiv.) in DMF (9 mL) was added dropwise over 15 minutes. Upon complete addition, the reaction mixture was allowed to warm to room temperature and stirred for 16 hours. H_2O (35 mL) and EtOAc (35 mL) were then added and the organics were separated. The aqueous phase was then extracted with EtOAc (2 × 50 mL). The combined organics were dried over $MgSO_4$, filtered, and concentrated in vacuo. The residue was heated to 50 °C and CH_2Cl_2 (35 mL) was added, resulting in the crystallisation of solids, which were filtered and dried under a vacuum to yield **13-P** (1.50 g, 62%) as a white powder.[4]

Compound 13-P: ^{1}H NMR (700 MHz, DMSO-d6): δ_H 1.60 (p, *J* = 7.6 Hz, 2H, (C3)-H), 2.18 (t, *J* = 7.5 Hz, 2H, (C4)-H), 2.67 (t, *J* = 7.6 Hz, 2H, (C2)-H), 7.26 (m, 3H, (C9)-H). 7.31 (m, 6H, (C7)-H), 7.34 (m, 6H, (C8)-H), 7.71 (br s, 2H, (C1)-H). **^{13}C NMR (175 MHz, DMSO-d6):** δ_C 26.2 (C3), 28.2 (C4), 38.1 (C2), 66.2 (C5), 126.8 (C9), 128.1 (C8), 129.1 (C7), 144.4 (C6). **IR (solid):** 2963.66, 2913.88, 1593.45, 1485.80, 1440.25, 1181.01, 1079.35, 1033.32, 1000.51, 753.64, 740.46, 698.22, 675.93, 621.96. **MP:** 150-160 °C. **HRMS (ESI$^+$):** Calcd. for $[C_{22}H_{23}NS+H]^+$ = 334.1630; observed 334.1622.

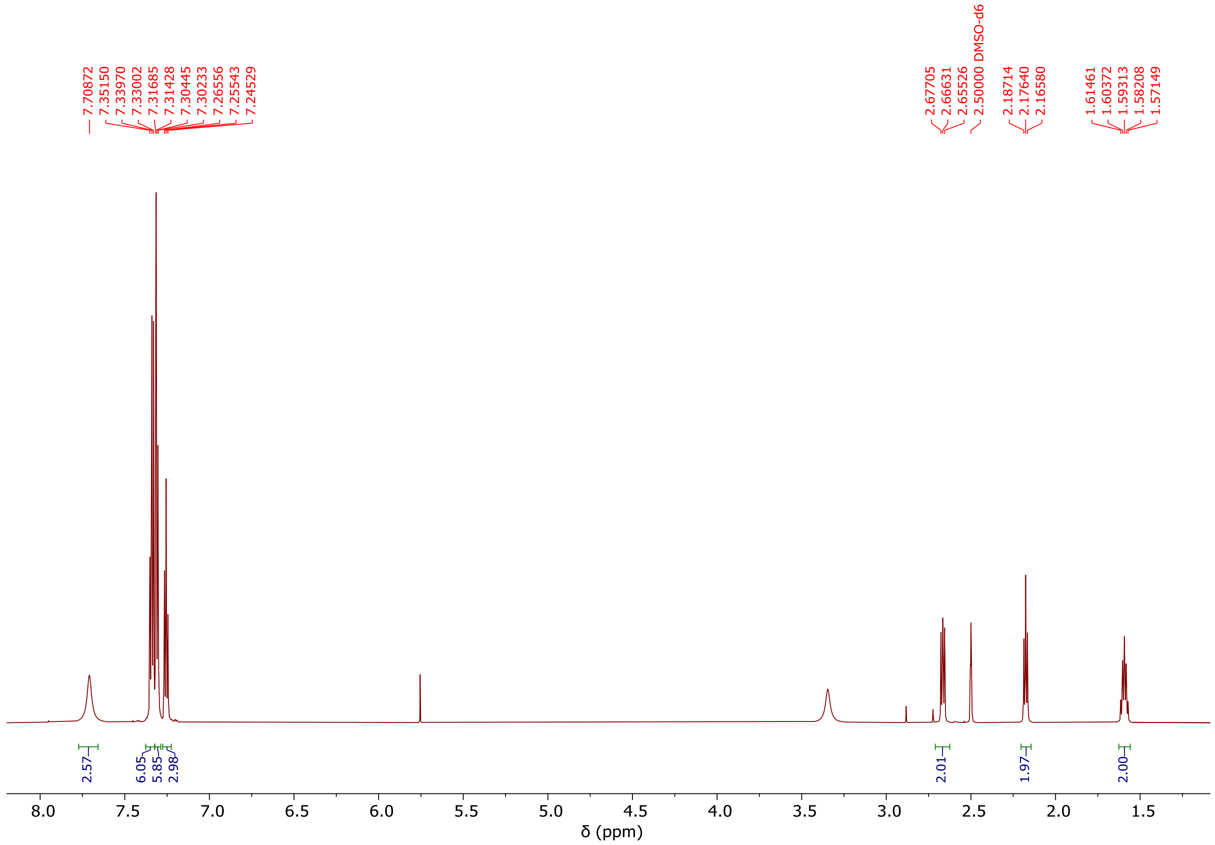

Figure S158: ^{1}H NMR (700 MHz, DMSO-d6, 1.0-8.0 ppm): Spectrum to show *S*-trityl homocysteamine **13-P**.

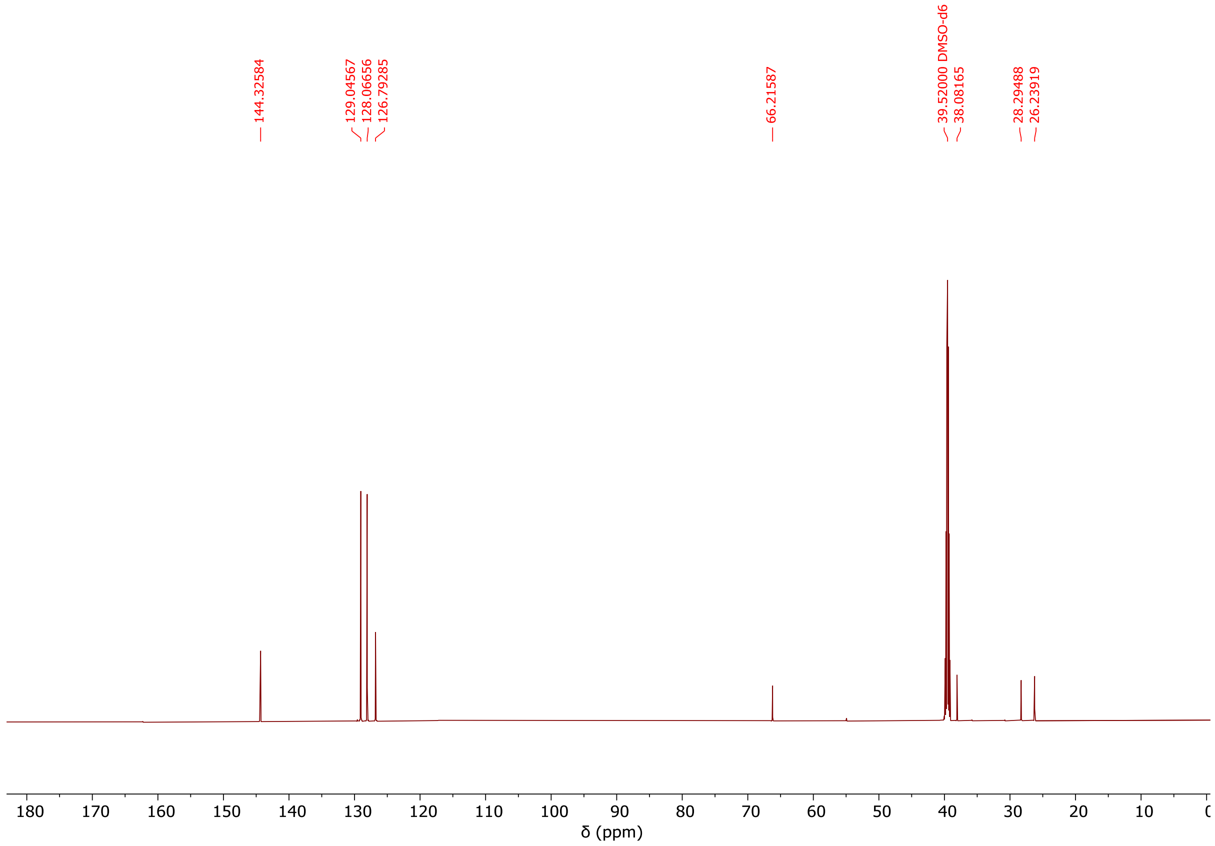

Figure S159: ^{13}C NMR (175 MHz, DMSO-d6, 0.0-180 ppm): Spectrum to show *S*-trityl homocysteamine **13-P**.

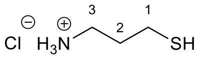

Synthesis of **13·**HCl: Trifluoroacetic acid (4.46 mL, 36.6 mmol, 30 equiv.) was added to a solution of *S*-trityl homocysteamine **13-P** (400 mg, 1.20 mmol, 1 equiv.) and triethylsilane (1.53 mL, 9.60 mL, 8 equiv.) in anhydrous CH_2Cl_2 (5 mL). The resultant solution was initially yellow and then became colourless within 1 minute. The solution was stirred for 10 mins and then concentrated *in vacuo*. The residue was dissolved in Et_2O (10 mL) and HCl (3 mL, 2 M in Et_2O) was added. The resultant precipitate was isolated by centrifugation and the supernatant was removed. The residual viscous oil was then triturated with Et_2O (5 × 10 mL) and dissolved in H_2O (1.5 mL) to yield **13·**HCl (402 mM, 0.61 mmol, 51%, determined against a potassium hydrogen phthalate internal standard). The solution was used without further purification.

Compound 13·HCl: **^{1}H NMR (700 MHz, H_2O/D_2O 9:1):** δ_H 1.90 (app. dt, *J* = 14.4, 7.0 Hz, 2H, (C2)-H), 2.57 (t, *J* = 7.0 Hz, 2H, (C1)-H), 3.05 (app. br. s, 2H, (C3)-H). **^{13}C NMR (175 MHz, H_2O/D_2O 9:1):** δ_C 21.3 (C1), 31.3 (C2), 38.9 (C3). **HRMS (ESI$^+$):** Calcd. for $[C_3H_9NS+H]^+$ = 92.0529; observed 92.0533.

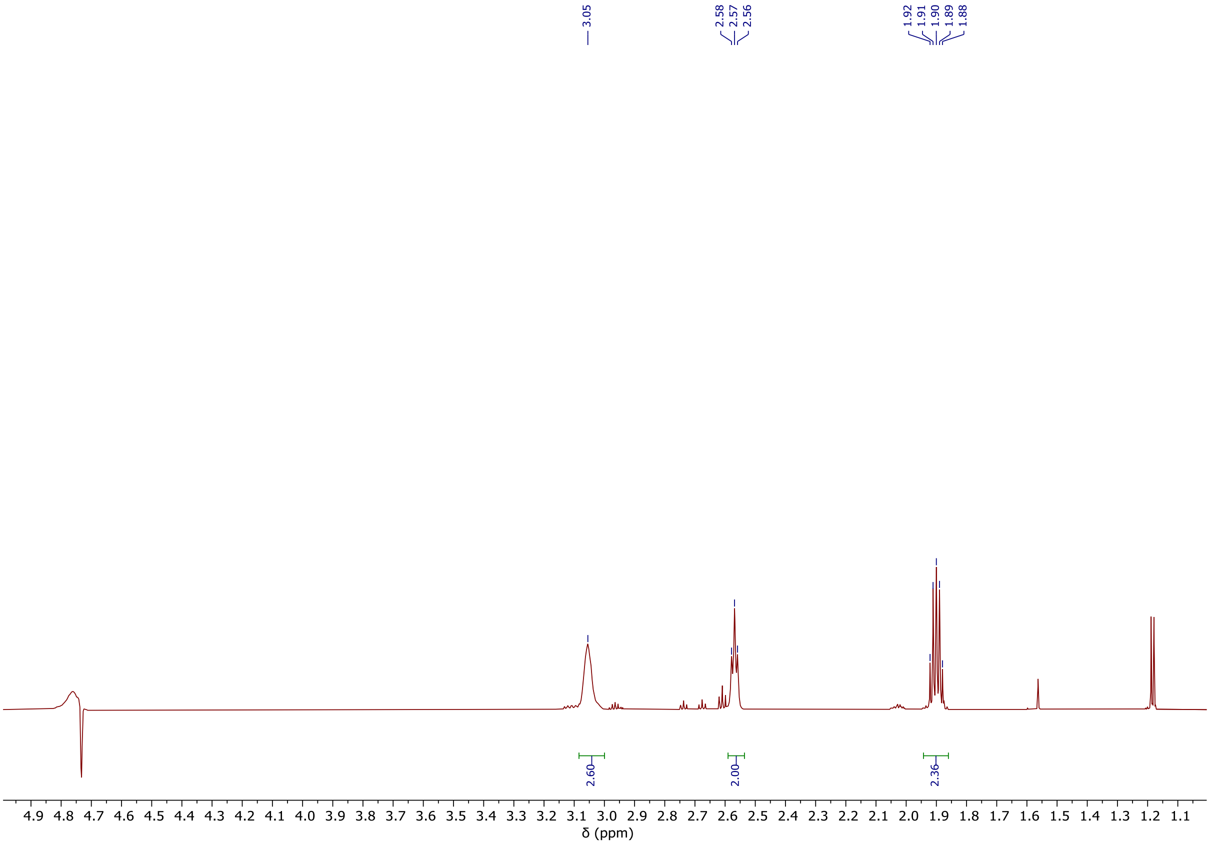

Figure S160: ^{1}H NMR (700 MHz, H_2O/D_2O 9:1, noesygppr1d, 1.0-5.0 ppm): Spectrum to show homocysteamine **13**·HCl.

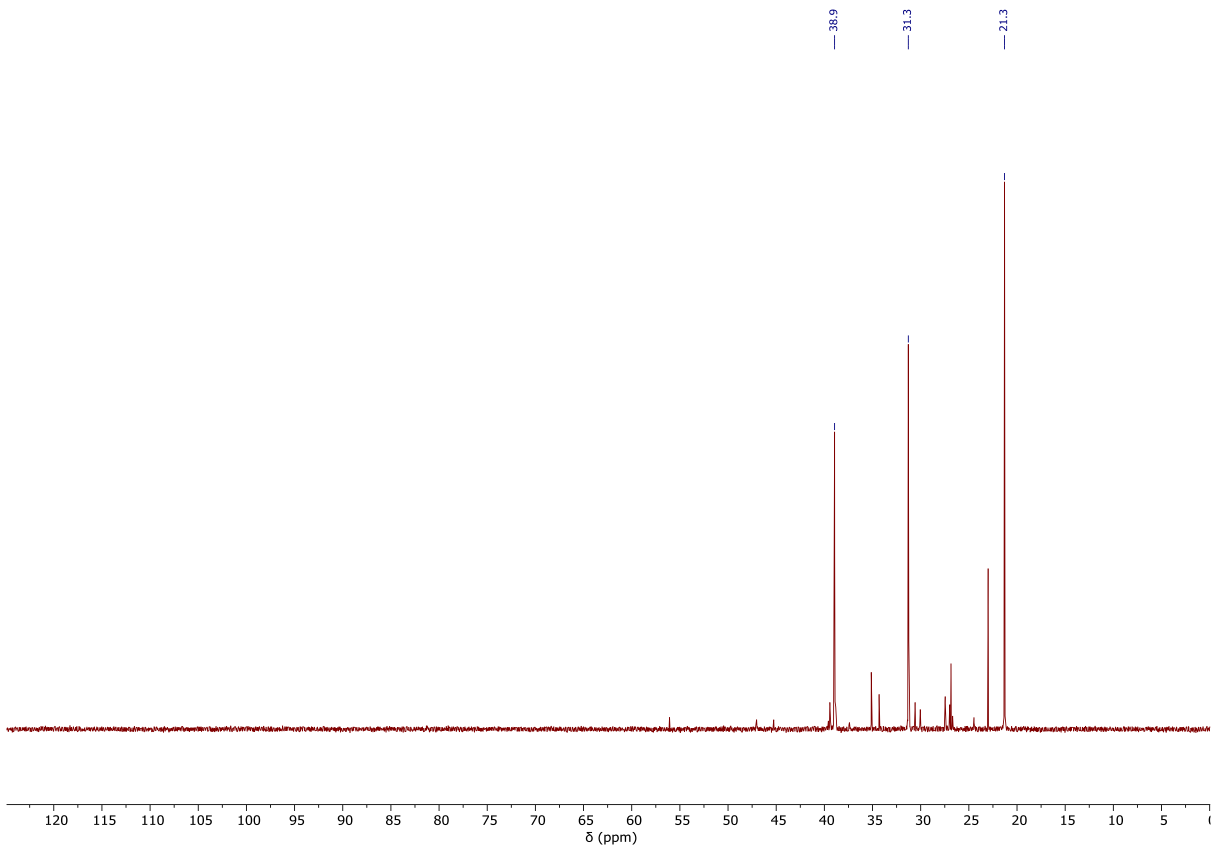

Figure S161: ^{13}C NMR (175 MHz, H_2O/D_2O 9:1, 0-130 ppm): Spectrum to show homocysteamine **13**·HCl.

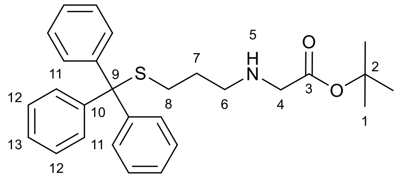

Synthesis of **17_G-P**: A solution of *tert*-butyl bromoacetate (0.59 mL, 3.99 mmol, 1 equiv) in anhydrous tetrahydrofuran (7.5 mL) was added dropwise to a stirred solution of **13-P** (1.33 g, 3.99 mmol, 1 equiv) and NEt_3 (1.11 mL, 7.98 mmol, 2 equiv.) in anhydrous tetrahydrofuran (7.5 mL) at room temperature over a period of 30 minutes. The resultant solution was stirred for 4 hours at room temperature. Silica was added to the reaction and concentrated *in vacuo*. The residue was loaded on to a column and purified via flash column chromatography (SiO_2; eluting with a gradient of 40-60 petroleum ether/EtOAc/NEt_3 100:0:1 → 50:50:1) to yield **17_G-P** (0.91 g, 51%) as a viscous, colourless oil.

Compound 17_G-P: Rf (*n*-Hexane/EtOAc 1:1) = 0.42. **^{1}H NMR (700 MHz, $CDCl_3$):** δ_H 1.45 (s, 9H, (C1)-H), 1.56 (p, *J* = 7.2 Hz, 2H, (C7)-H), 2.21 (t, *J* = 7.2, 2H, (C8)-H), 2.54 (t, *J* = 7.2, 2H, (C6)-H), 3.21 (s, 2H, (C4)-H), 7.20 (m, 3H, (C13)-H), 7.28 (m, 6H, (C12)-H), 7.41 (m, 6H, (C11)-H). **^{13}C NMR (175 MHz, $CDCl_3$):** δ_C 28.3 (C1), 29.2 (C7), 29.9 (C8), 48.6 (C6), 51.6 (C4), 66.7 (C9), 81.4 (C2), 126.7 (C13), 128.0 (C12), 129.8 (C11), 145.1 (C10). **IR (solid):** 3056.54, 2976.07, 2928.74, 1730.19, 1594.70, 1487.87, 1443.30, 1391.70, 1366.75, 1224.90, 1148.58, 1081.52, 1033.32, 874.20, 740.94, 696.82, 675.74, 617.59, 505.78. **HRMS (ESI$^+$):** Calcd. for $[C_{28}H_{33}NO_2S+H]^+$ = 448.2305; observed 448.2297.

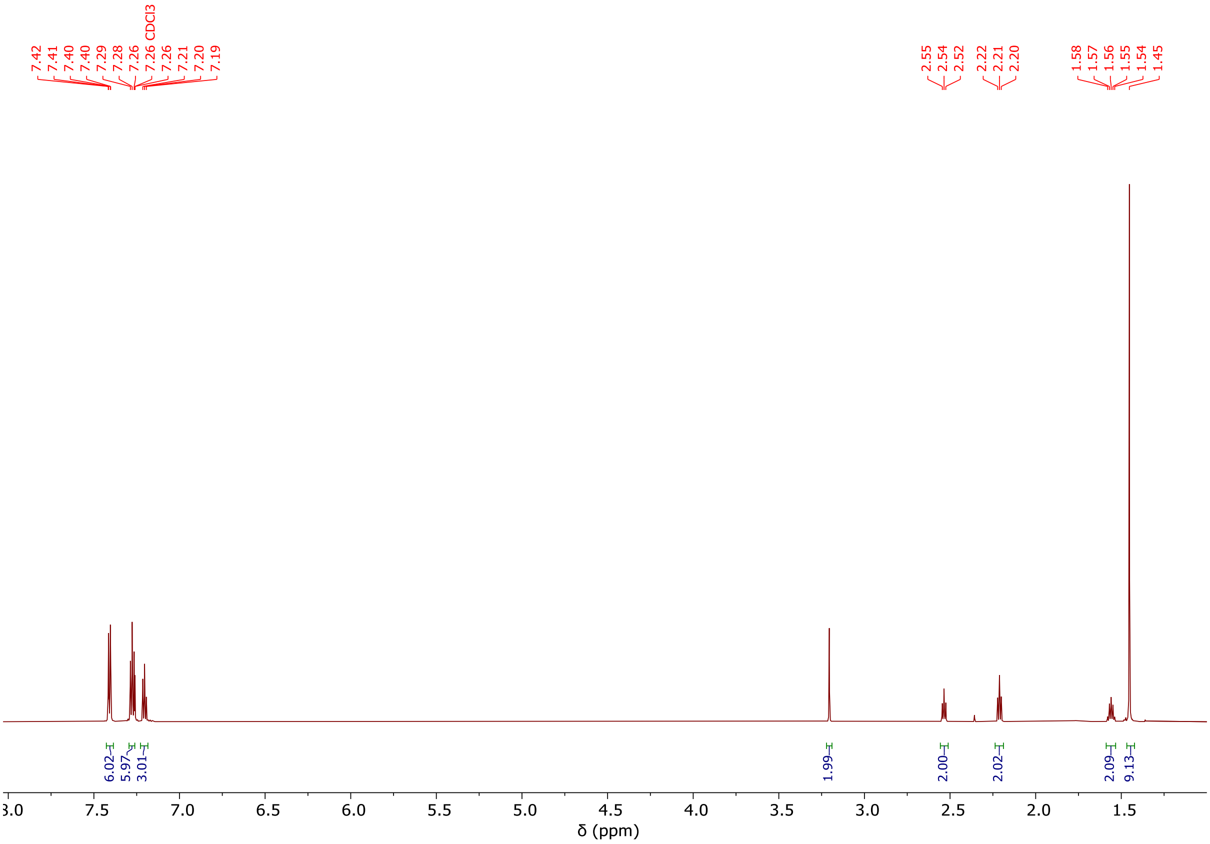

Figure S162: ^{1}H NMR (700 MHz, $CDCl_3$, 1.0-8.0 ppm): Spectrum to show **17_G-P**.

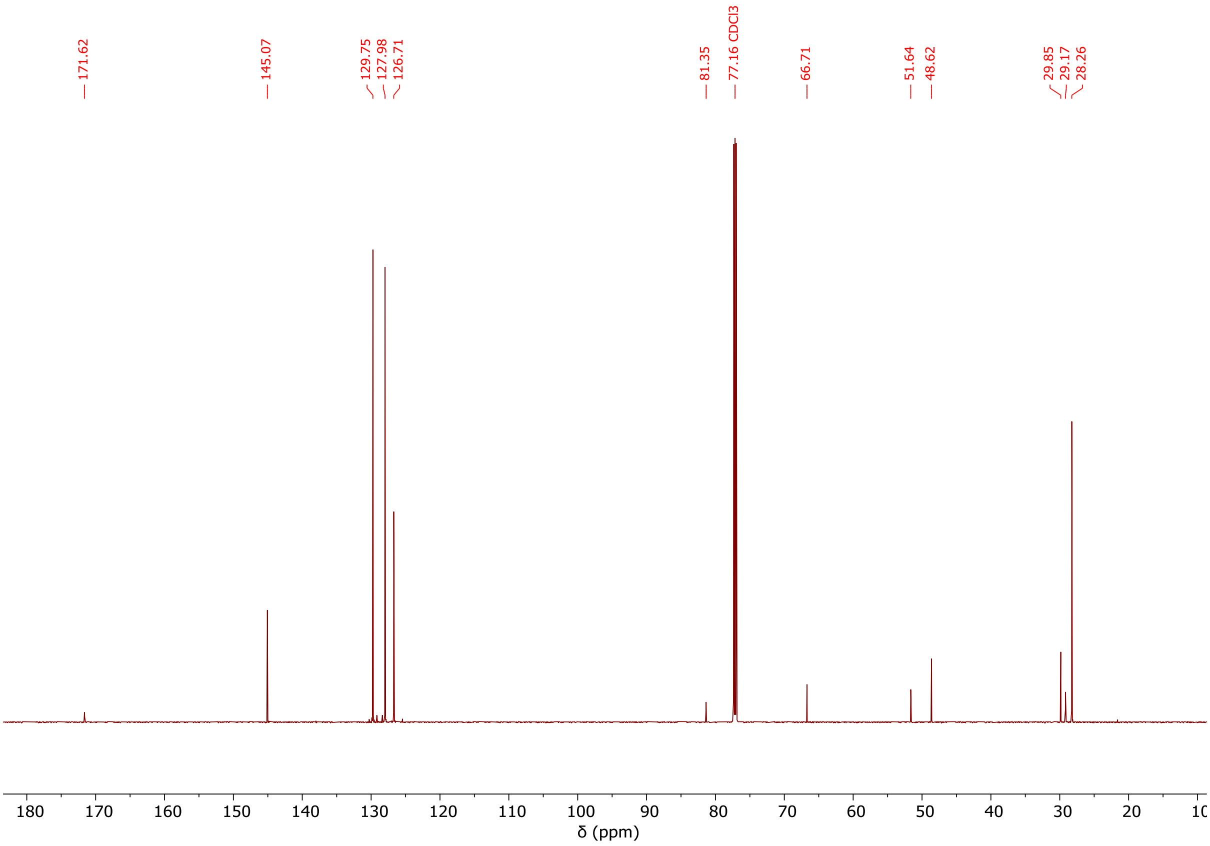

Figure S163: ^{13}C NMR (175 MHz, $CDCl_3$, 10-180 ppm): Spectrum to show **17_G-P**.

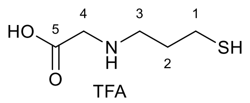

Synthesis of **17_G**·TFA: Trifluoroacetic acid (9.85 mL, 79.55 mmol, 30 equiv.) was added to a solution of **17_G-P** (1.18 g, 2.65 mmol, 1 equiv.) and triisopropylsilane (5.43 mL, 26.52 mmol, 10 equiv.) in anhydrous CH_2Cl_2 (6 mL). The resultant solution was initially deep yellow and became colourless within 1 minute. The solution was then stirred for 1.5 hours at room temperature and concentrating *in vacuo*. The residue was washed with Et_2O (3 × 50 mL) to yield **17_G**·TFA (340 mg, 49%) as a viscous, colourless oil.

Compound 17_G·TFA: **^{1}H NMR (400 MHz, D_2O):** δ_H 2.01 (p, *J* = 7.1 Hz, 2H, (C2)-H), 2.62 (t, *J* = 6.90 Hz, 2H, (C1)-H), 3.23 (t, *J* = 7.72 Hz, 2H, (C3)-H), 3.91 (s, 2H, (C4)-H). **^{13}C NMR (100 MHz, D_2O):** δ_C 21.1 (C1), 29.8 (C2), 46.7 (C3), 48.2 (C4), 169.9 (C5). **^{19}F NMR (376 MHz, D_2O):** δ_F -75.57. **IR (solid):** 2959.97, 2807.04, 1732.84, 1664.77, 1459.43, 1405.89, 1312.21, 1242.56, 1174.18, 1127.05, 901.51, 829.71, 795.62, 718.54, 663.30, 595.69, 517.62, 440.23. **HRMS (ESI$^+$):** Calcd. for $[C_5H_{11}NO_2S+H]^+$ = 150.0583; observed 150.0580.

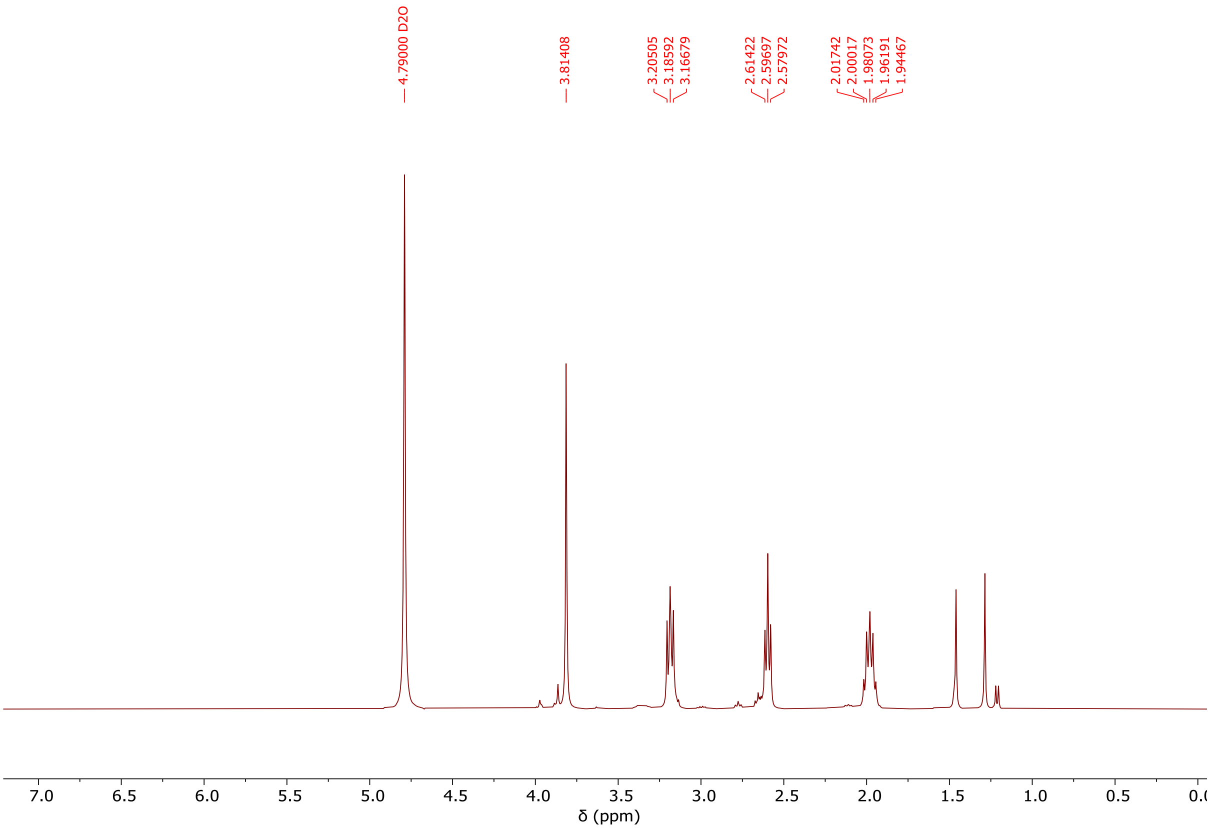

Figure S164: ^{1}H NMR (400 MHz, D_2O, 0.0-7.0 ppm): Spectrum to show **17_G**·TFA.

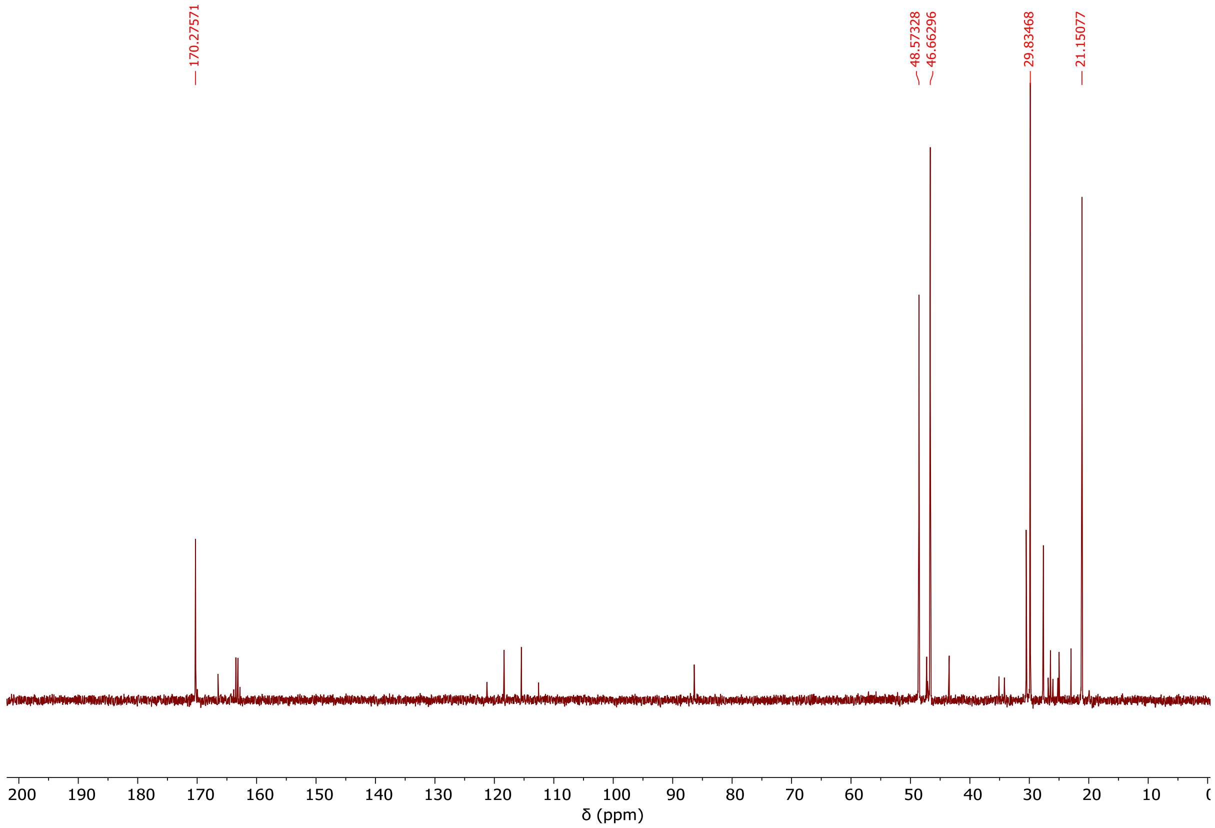

Figure S165: ^{13}C NMR (100 MHz, D_2O, 0.0-200 ppm): Spectrum to show $\mathbf{17_G}$·TFA.

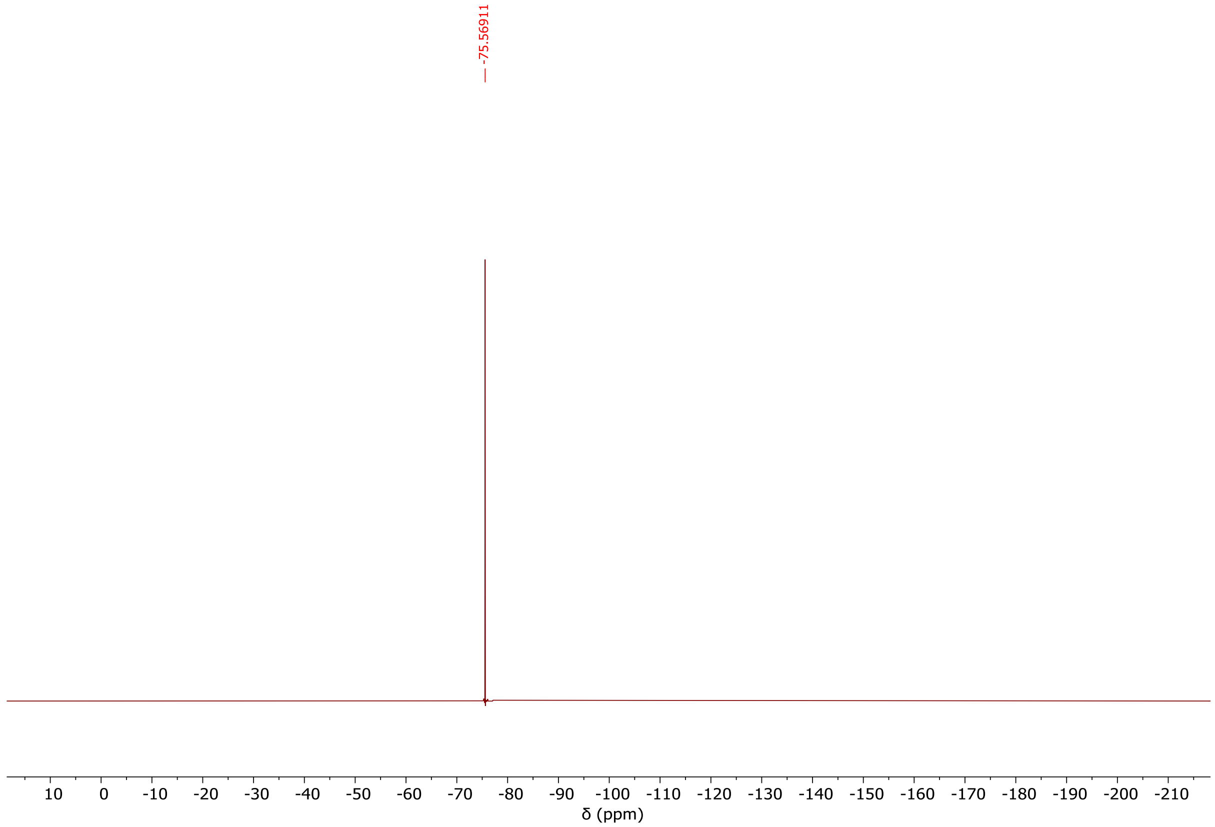

Figure S166: ^{19}F NMR (376 MHz, D_2O, -220-20 ppm): Spectrum to show $\mathbf{17_G}$·TFA.

Synthesis of auxiliary **30$_G$**:

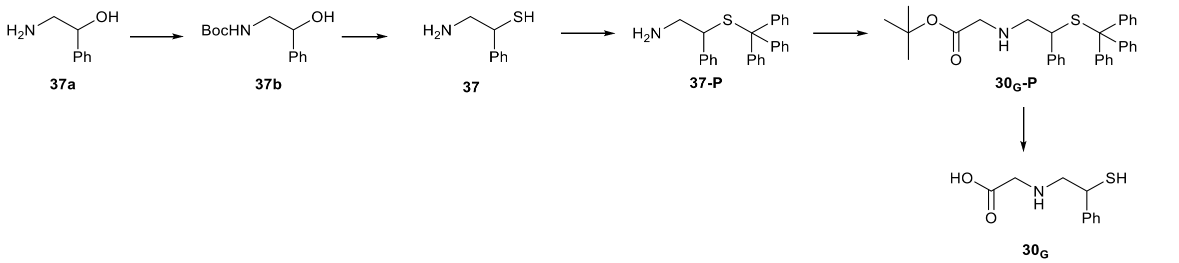

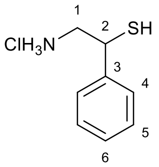

Synthesis of **37**·HCl: A solution of $(Boc)_2O$ (2.63 g, 12.03 mmol, 1.1 equiv.) in anhydrous CH_2Cl_2 (5 mL) was added to amino alcohol **37a** (1.50 g, 1.93 mmol, 1 equiv.) in anhydrous CH_2Cl_2 (15 mL) at 0°C, and the resultant solution was stirred for 5 mins at 0°C and then warmed to room temperature. After 1.8 hours at room temperature, NEt_3 (1.83 mL, 13.12 mmol, 1.2 equiv.) was added and the solution was stirred at room temperature. After 30 mins the solution was diluted with CH_2Cl_2 (20 mL) and the organics were washed with $NaHCO_3$ (40 mL; sat. aqueous solution) and brine (40 mL). The organics were then dried over $MgSO_4$, filtered and concentrated *in vacuo* to yield N-Boc-amino alcohol **37b** (2.51 g, 9.90 mmol, 91%). Diisopropyl azodicarboxylate (2.95 mL, 14. 98 mmol, 1.5 equiv.) was added to a solution of PPh_3 (3.93 g, 14.98 mmol, 1.5 equiv.) in anhydrous THF (50 mL) in flame-dried glassware at 0°C and the reaction was stirred for 30 minutes. A thick suspension formed. N-Boc amino alcohol **37b** (2.37 g, 9.99 mmol, 1 equiv.) and thioacetic acid (1.07 mL, 14.98 mmol, 1.5 equiv.) were added to the suspension at 0 °C and he suspension was stirred for 1 hour at 0 °C. The suspension was then allowed to warm to room temperature and stirred for 1 hour – the suspension became a clear yellow solution during this period. The reaction was concentrated *in vacuo* and purified via flash column chromatography (SiO_2; eluting with a gradient of 40-60 petroleum ether/EtOAc 100:00 → 70:30). $OPPh_3$ was observed to crystallise from the eluent. The product was concentrated *in vacuo* and then dissolved in H_2O/MeOH/THF (25 mL, 1:4:5). NaOH (1.27 g, 31.70 mmol) was added to the solution under a nitrogen atmosphere and the solution was stirred at room temperature. After 20 mins, the product was concentrated in vacuo and the residue was dissolved in H_2O (25 mL). The solution was adjusted to pH 7 with HCl (4 M) and extracted into CH_2Cl_2 (3 × 50 mL). The combined organics were dried over $MgSO_4$, filtered and concentrated in vacuo. HCl (10 mL, 2 M in Et_2O) was added to the residue and the resultant solution was stirred for 2 hours at room temperature under a nitrogen atmosphere. The precipitate was isolated by filtration and the white solids were triturated with Et_2O (2 × 50 mL) to yield the amino-thiol hydrochloride **37**·HCl (457 mg) as a white solid. The supernatant

(organics) were concentrated *in vacuo* and HCl (5 mL, 2 M in Et_2O) was added. The solution was stirred for 2 hours and the precipitate was isolated by filtration. The solids were combined and triturated with Et_2O (2 × 30 mL) to yield **37**·HCl (703 mg, 3.71 mmol, 37% over 3 steps) as a white powder.

Compound 37·HCl: **^{1}H NMR (700 MHz, D_2O):** δ_H 3.52 (ABX, *J* = 13.1, 7.9 Hz, 1H, (C1)-H), 3.59 (ABX, *J* = 13.1, 7.9 Hz, 1H, (C1)-H), 4.3 (t, *J* = 7.9 Hz, 1H, (C2)-H), 7.43-7.46 (m, 1H, (C6)-H), 7.48-7.53 (m, 4H, (C4)-H & (C5)-H). **^{13}C NMR (175 MHz, D_2O):** δ_C 40.9 (C2), 46.7 (C1), 127.6 (C4), 129.3 (C6), 130.1 (C5), 140.2 (C3). **IR (solid):** 3407.27, 2934.37, 2839.12, 2730.65, 2615.85, 2477.00, 1983.56, 1596.47, 1506.66, 1488.93, 1452.69, 1382.30, 1328.44, 1252.33, 1184.10, 1139.78, 1101.26, 1030.32, 963.61, 906.30, 868.87, 823.58, 754.25, 693.86, 669.84, 655.31, 615.77, 520.38, 438.55. **HRMS (ESI$^+$):** Calcd. for $[C_8H_{11}NS+H]^+$ = 154.0685; observed 154.0681.

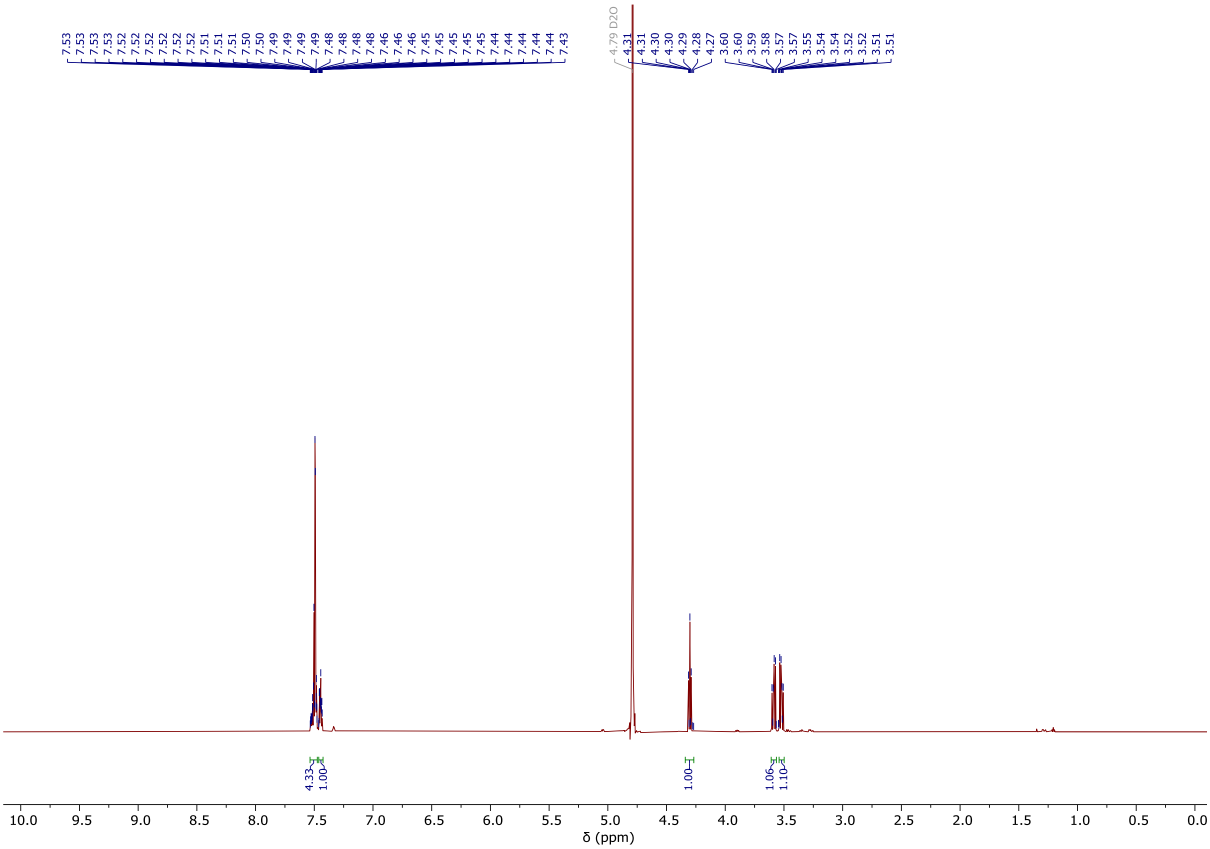

Figure S167: ^{1}H NMR (700 MHz, D_2O, 0.0-10.0 ppm): Spectrum to show **37**·HCl.

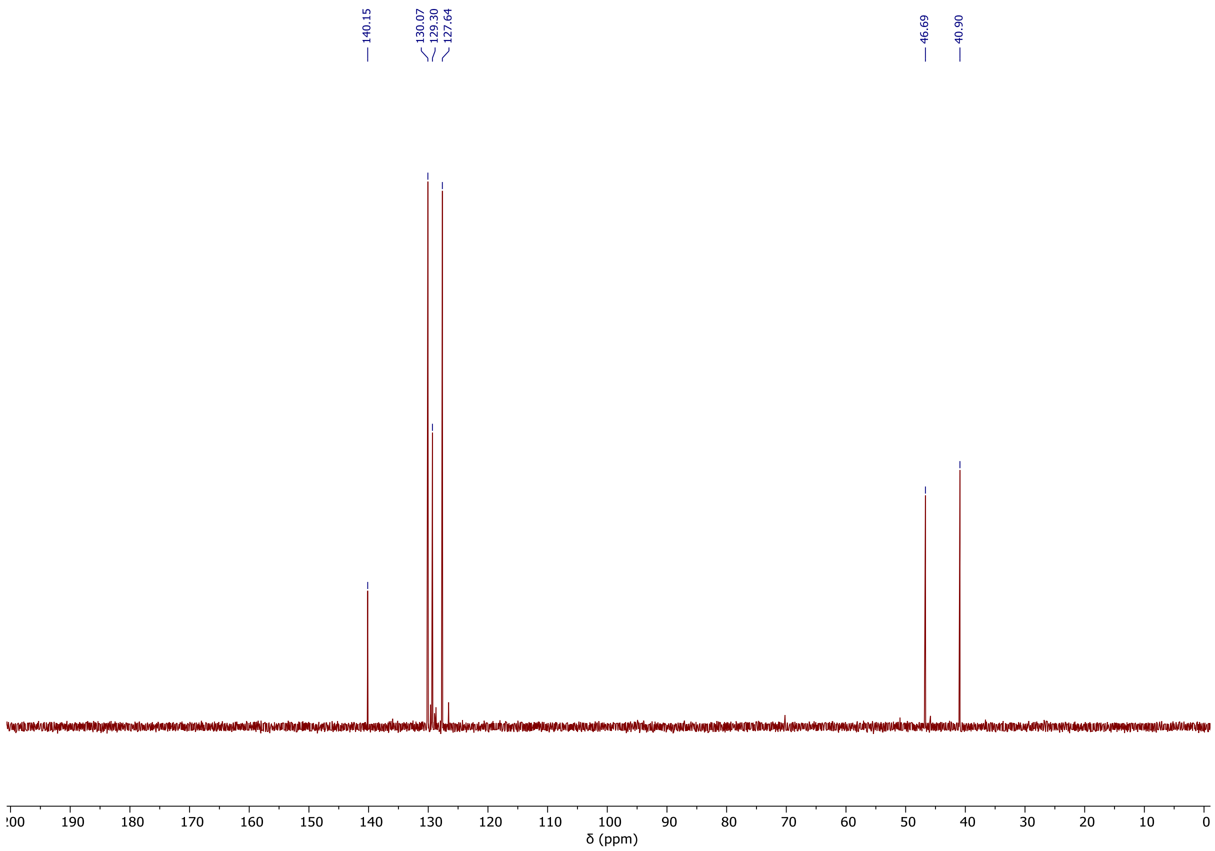

Figure S168: ^{13}C NMR (175 MHz, D_2O, 0.0-200 ppm): Spectrum to show **37**·HCl.

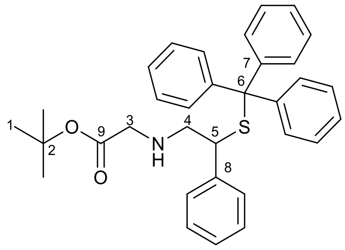

Synthesis of **30_G-P**: Trityl chloride (864 mg, 3.10 mmol, 1 equiv.) was added to a solution of **37**·HCl (589 mg, 3.10 mmol, 1 equiv.) in trifluoroacetic acid (2.5 mL) and the resultant solution was stirred at room temperature for 3 hours. The solution was concentrated *in vacuo* and the residue dissolved in EtOAc (15 mL). The organics were extracted with NaOH (4 × 5 mL, 3 M), H_2O (5 mL), $NaHCO_3$ (2 × 5 mL, sat. aqueous solution) and brine (3 × 5 mL). The organics were then dried over $MgSO_4$, filtered and concentrated *in vacuo*. The product was purified by flash column chromatography (SiO_2; eluting with a gradient of CH_2Cl_2/MeOH 100:00 → 90:10) to yield *S*-tritylated amino-thiol **37-P** (994 mg, 2.51 mmol, 81%) as a sticky white solid. The white solids were dissolved in anhydrous tetrahydrofuran (10 mL) and NEt_3 (0.70 mL, 5.03 mmol, 2 equiv.) was added. Then a solution of tert-butyl bromoacetate (0.37 mL, 2.51 mmol, 1 equiv.) in anhydrous tetrahydrofuran (5 mL) was added dropwise over 15 minutes. Upon complete addition, the solution was stirred for 19 hours at room temperature and then concentrated in vacuo. The product was purified by flash column chromatography (SiO_2; eluting with a gradient of *n*-hexane/Et_2O 100:00 → 50:50) to yield **30_G-P** (861 mg, 1.69 mmol, 67%) as a sticky, white solid.

Compound 30_G-P: ^{1}H NMR (500 MHz, MeOD-d4): δ_H 1.39 (s, 9H, (C1)-H), 2.22 (ABX, *J* = 12.4, 5.6 Hz, 1H, (C4)-H), 2.79 (ABX, *J* = 12.4, 10.0 Hz, 1H, (C4)-H), 2.77 (AB, *J* = 17.1 Hz, 1H, (C3)-H), 2.90 (AB, *J* = 17.1 Hz, 1H, (C3)-H), 3.35 (ABX, *J* = 9.9, 5.6 Hz, 1H, (C5)-H), 7.10 (m, 2H, Ar-H), 7.17 – 7.31 (m, 12H, Ar-H), 7.38 (m, 6H, Ar-H). **^{13}C NMR (125 MHz, MeOD-d4):** δ_C 28.3 (C1), 50.3 (C5), 51.4 (C3), 55.5 (4), 70.1 (C6), 82.4 (C2), 127.8 - 131.0 (Ar), 142.6 (C8), 146.1 (7), 172.0 (C9). **IR (solid):** 3057.01, 3027.37, 3002.15, 2975.96, 2930.39, 1730.86, 1595.81, 1489.04, 1444.42, 1391.41, 1366.37, 1226.01, 1143.31, 1082.07, 1032.43, 1001.16, 937.89, 846.19, 764.64, 739.99, 694.84, 674.37, 617.93, 577.98, 528.82, 505.24. **HRMS (ESI$^+$):** Calcd. for $[C_{33}H_{35}NO_2S+H]^+$ = 510.2461; observed 510.2466.

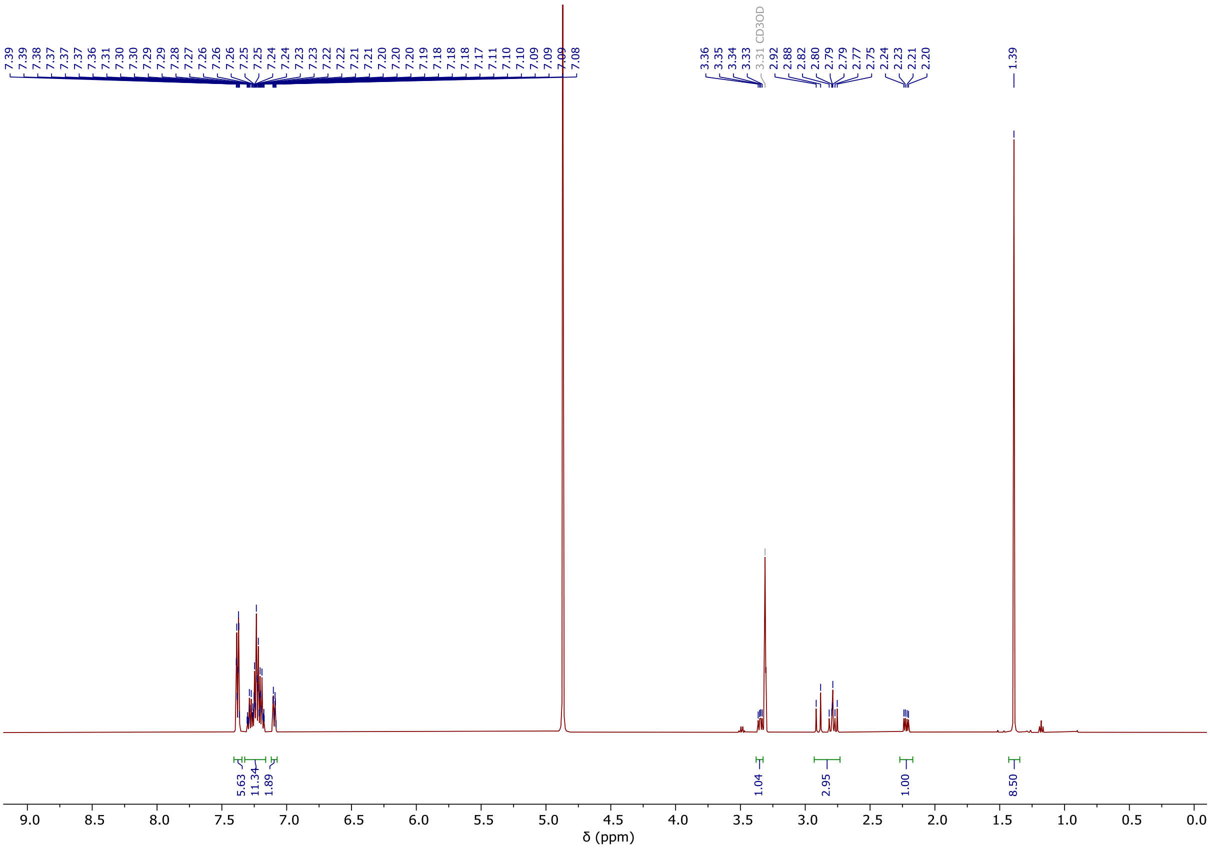

Figure S169: ^{1}H NMR (500 MHz, MeOD-d4, 0.0-9.0 ppm): Spectrum to show **30_G-P**.

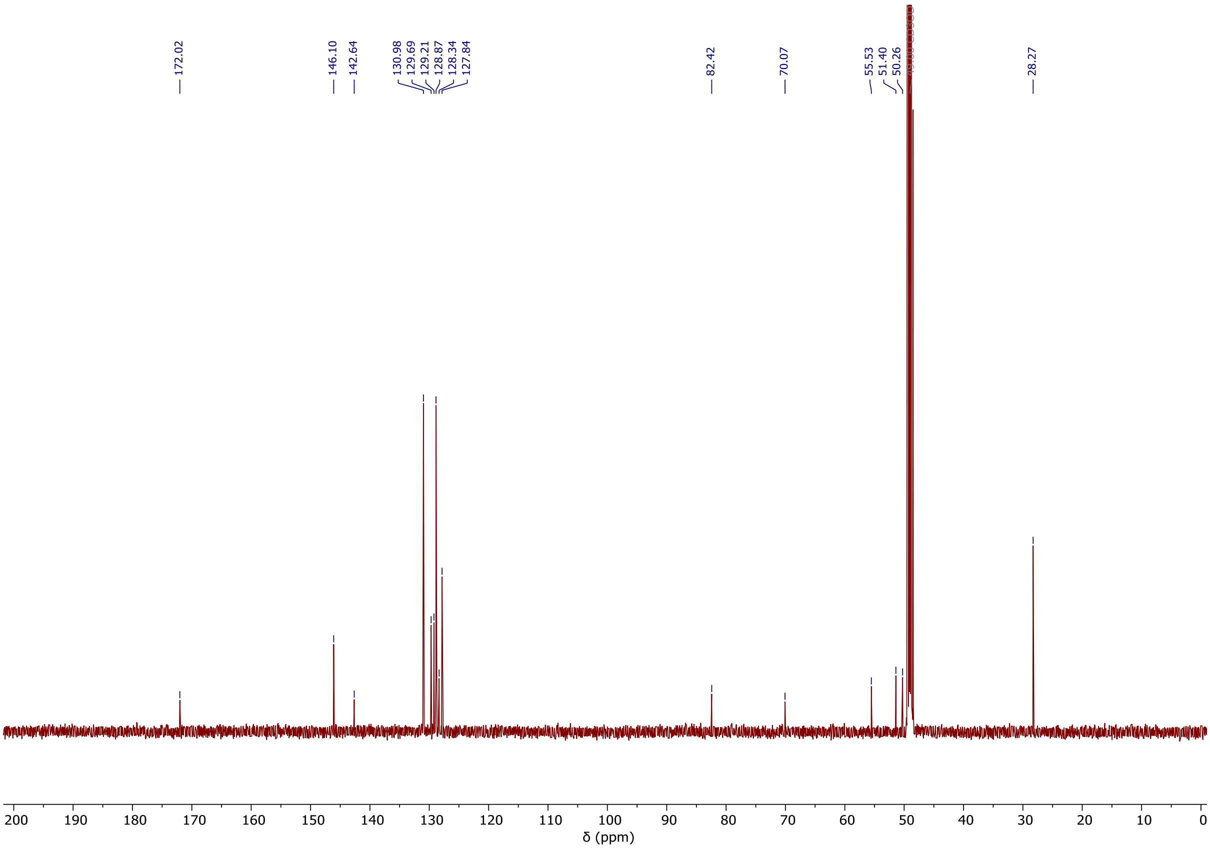

Figure S170: ^{13}C NMR (125 MHz, MeOD-d4, 0.0-200 ppm): Spectrum to show **30_G-P**.

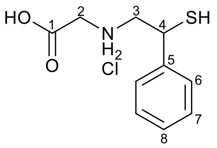

Synthesis of **30_G**·HCl: Trifluoroacetic acid (4.65 mL, 37.43 mmol, 30 equiv.) was added to a solution of **30_G-P** (636 mg, 1.25 mmol 1 equiv.) and triethylsilane (2.00 mL, 12.48 mmol, 10 equiv.) in anhydrous CH_2Cl_2 (5 mL) and the resulting solution was stirred for 3 hours and then concentrated *in vacuo*. Et_2O (20 mL) and then HCl (5 mL; 2 M in Et_2O) were added to the residue and then stirred. After 5 minutes the resultant suspension was concentrated *in vacuo*. The residue was triturated with Et_2O (3 × 20 mL) to yield **30_G**·HCl (298 mg, 1.20 mmol, 96%) as a white solid.

Compound 30_G·HCl: ^{1}H NMR (500 MHz, D_2O): δ_H 3.61 (ABX, *J* = 12.8, 8.1 Hz, 1H, (C3)-H), 3.70 (ABX, *J* = 12.8, 7.8 Hz, 1H, (C4)-H), 3.91 (AB, *J* = 20.0 Hz, 1H, (C2)-H), 3.96 (AB, *J* = 20.0 Hz, 1H, (C2)-H), 4.35 (t, *J* = 7.9 Hz, 1H, (C4)-H), 7.41 (m, 1H, (C8)-H), 7.46 (m, 4H, (C6)-H & (C8)-H). **^{13}C NMR (125 MHz, D_2O):** δ_C 40.1 (C4), 48.3 (C2), 54.6 (C3), 127.8 (C6), 129.6 (C8), 130.3 (C7), 140.1 (C5), 169.6 (C1). **IR (solid):** 2925.43, 2772.58, 2617.53, 2509.13, 2409.42, 1749.77, 1550.20, 1492.21, 1452.58, 1411.21, 1375.11, 1306.39, 1215.02, 1069.04, 1028.39, 893.19, 851.20, 821.02, 755.65, 692.41, 661.79, 604.70, 514.21. **MP:** 118.5-124.0 °C. **HRMS (ESI$^+$):** Calcd. for $[C_{10}H_{13}NO_2S+H]^+$: 212.0740; observed 212.0741.

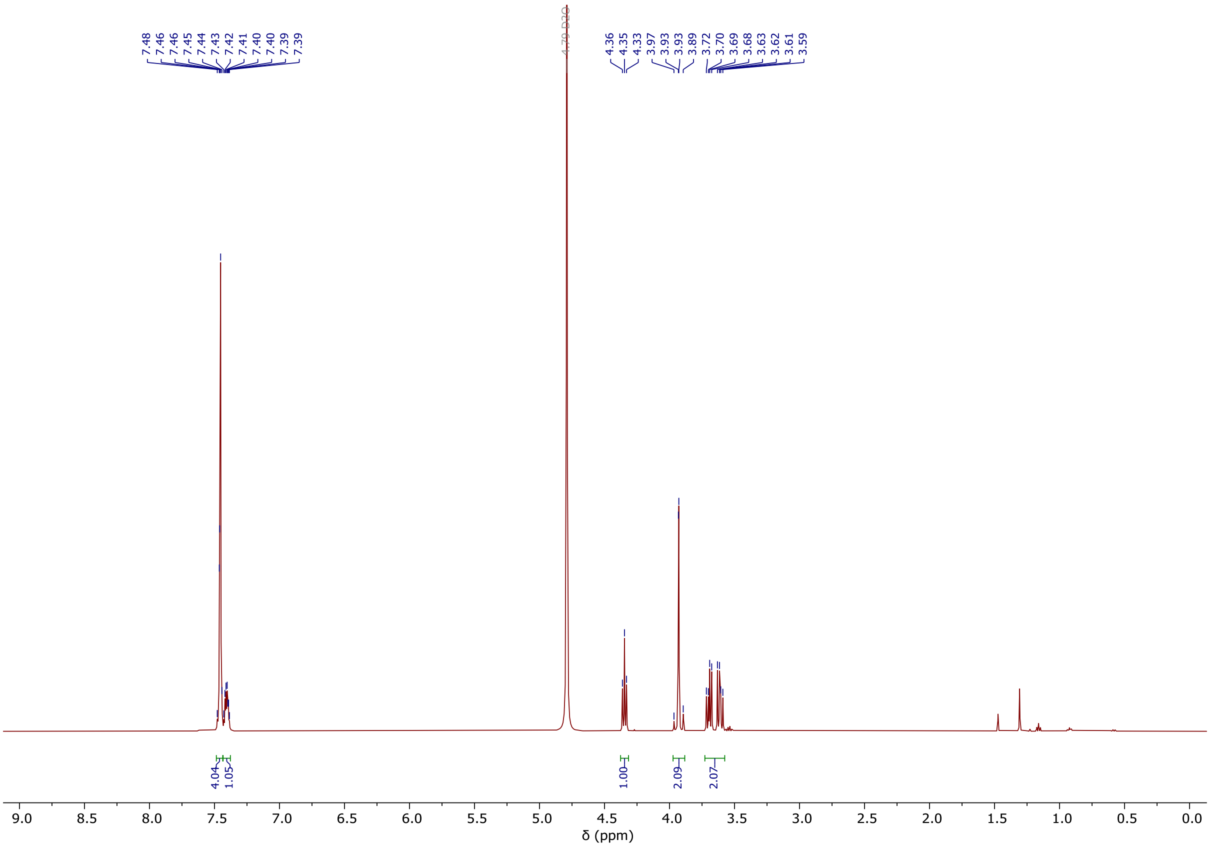

Figure S171: ^{1}H NMR (500 MHz, D_2O, 0.0-9.0 ppm): Spectrum to show **30_G**·HCl.

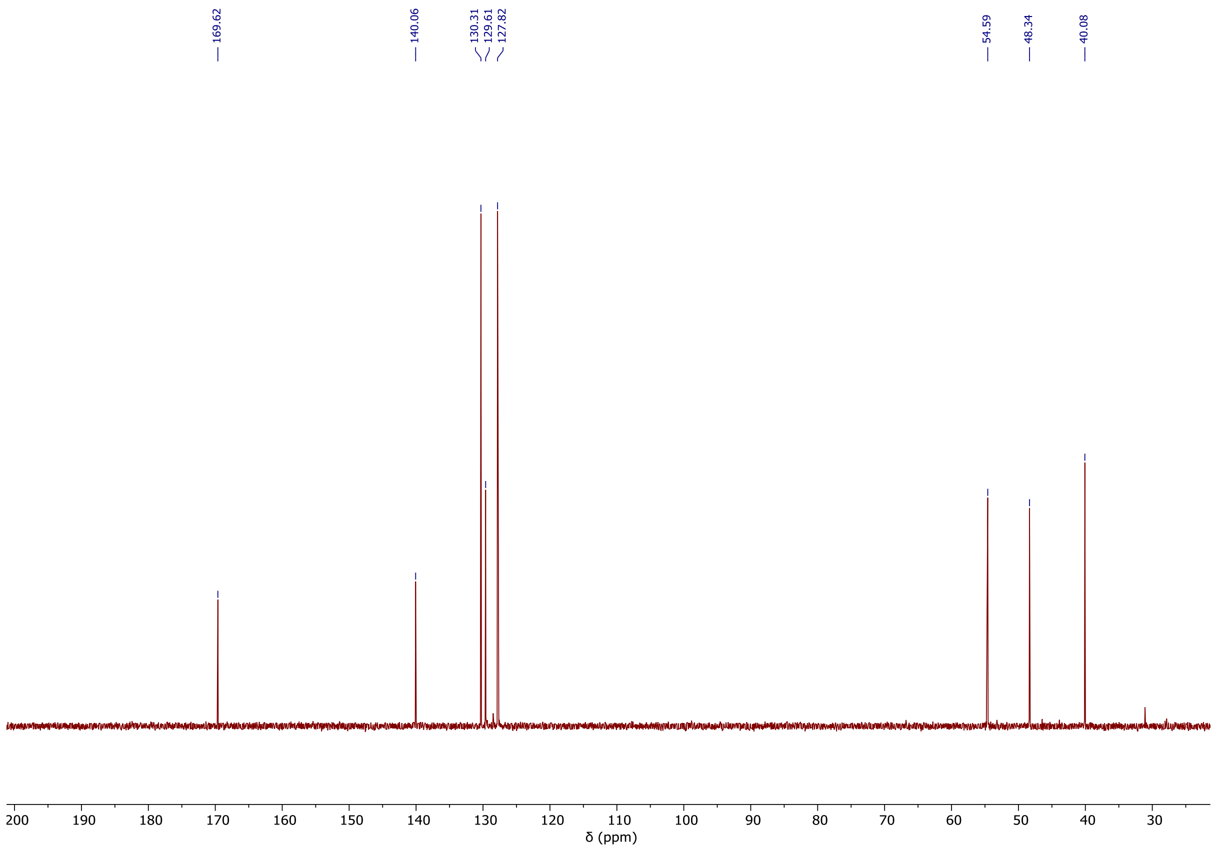

Figure S172: ^{13}C NMR (125 MHz, D_2O, 20-200 ppm): Spectrum to show $\mathbf{30_G}$·HCl.

Synthetic preparation of ligation product **10a**:

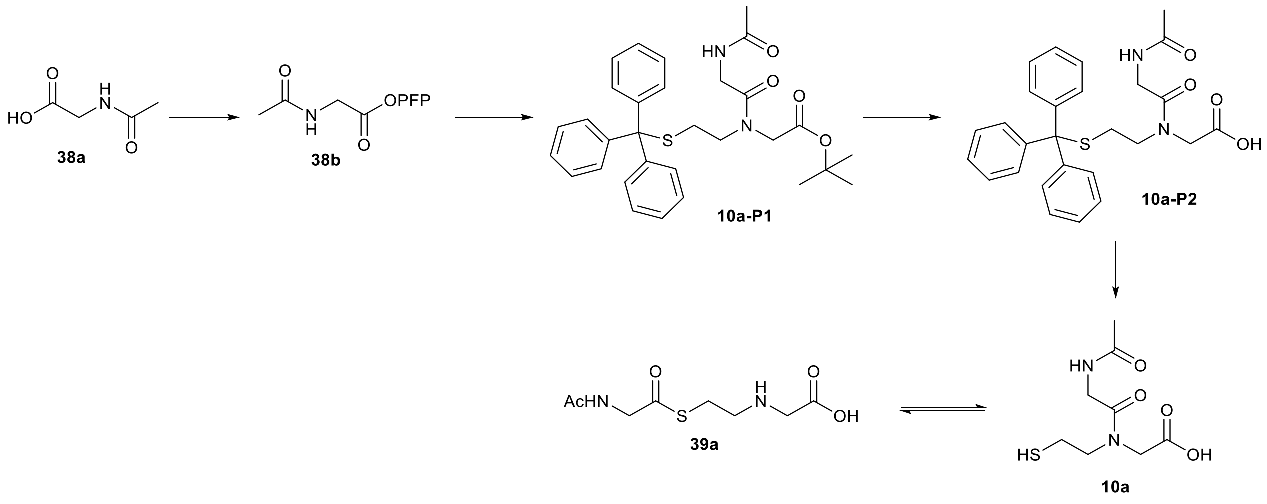

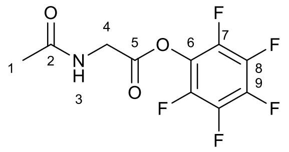

Synthesis of **38b**: **38a** (2.00 g, 17.08 mmol, 1 equiv.), pentafluorophenol (4.72 g, 25.62 mmol, 1.5 equiv.) and EDC·HCl (9.82 g, 51.24 mmol, 3 equiv.) were dissolved in anhydrous CH_2Cl_2 (40 mL) at 0°C. The resultant solution was stirred for 10 mins, then warmed to room temperature and stirred for a further 16 hours. The solution was diluted with CH_2Cl_2 (40 mL) and the organics were washed with H_2O (2 × 40 mL), $NaHCO_3$ (2 × 40 mL; sat. aqueous solution) and brine (40 mL). The organics were then dried over $MgSO_4$, filtered and concentrated in vacuo. The product was purified via flash column chromatography (SiO_2; Petrol 40-60/EtOAc 100:00 → 00:100) to yield **38b** (2.3 g, 12.7 mmol, 74%) as a white solid.

Compound 38b: Rf (Hexane/EtOAc 1:1) = 0.33, **^{1}H NMR (700 MHz, CDCl$_3$):** δ_H 2.09 (s, 3H, (C1)-H), 4.42 (s, 2H, (C4)-H), 6.07 (broad-s, N-H, 1H, H3). **^{13}C NMR (175 MHz, CDCl$_3$):** δ_C 22.98 (C1), 40.85 (C4), 124.7 (C6), 137.4 (ArF), 138.8 (ArF), 139.2 (ArF), 140.40 (ArF), 140.7 (ArF), 141.8 (ArF), 166.61 (C5), 170.48 (C2). **^{19}F NMR (658 MHz, CDCl$_3$):** δ_F -161.77 (m, 2F, F8), -157.03 (t, 1F, F9), -152.27 (m, 2F, F7). **IR (solid):** 3272.57, 3263.43, 2960.03, 1796.73, 1786.74, 1648.66, 1516.97, 1470.88, 1406.79, 1371.39, 1305.19, 1260.70, 1159.99, 1136.06, 1103.22, 1030.21, 989.81, 905.82, 741.70, 706.06, 679.90, 626.00, 597.91, 543.91, 485.26, 442.34. **MP:** 101.4-109.0 °C. **HRMS (ESI$^+$):** Calcd. for $[C_{10}H_6NF_5O_3+H]^+$ = 284.0341; observed 284.0338.

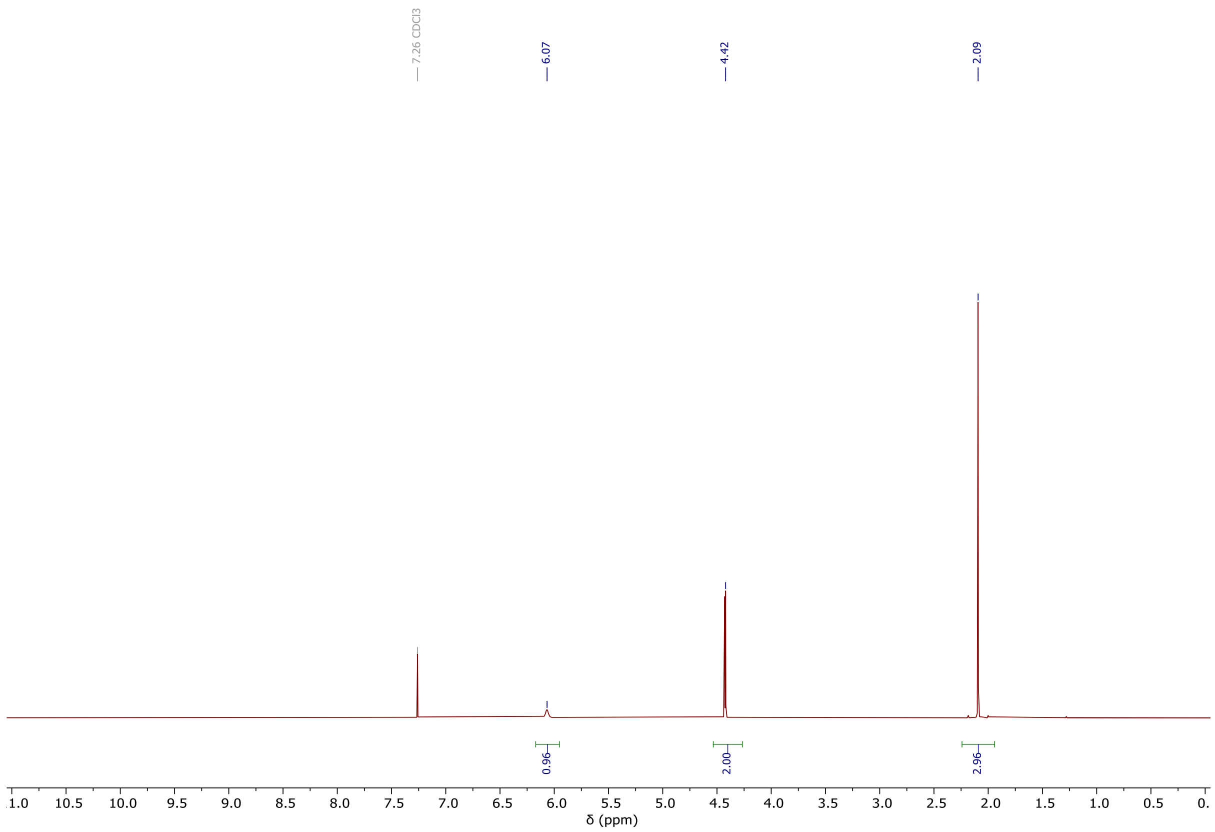

Figure S173: ^{1}H NMR (700 MHz, $CDCl_3$, 0.0-11.0 ppm): Spectrum to show **38b**.

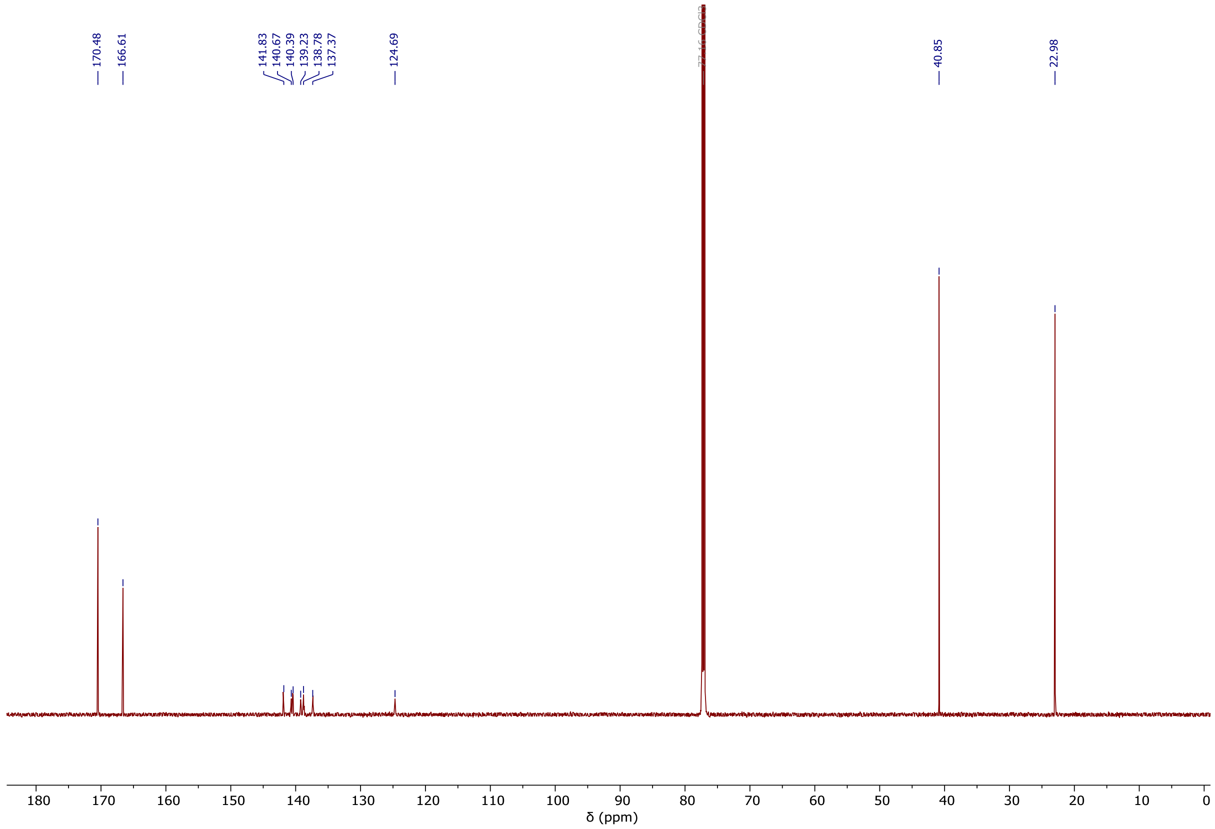

Figure S174: ^{13}C NMR (175 MHz, $CDCl_3$, 0.0-185 ppm): Spectrum to show **38b**.

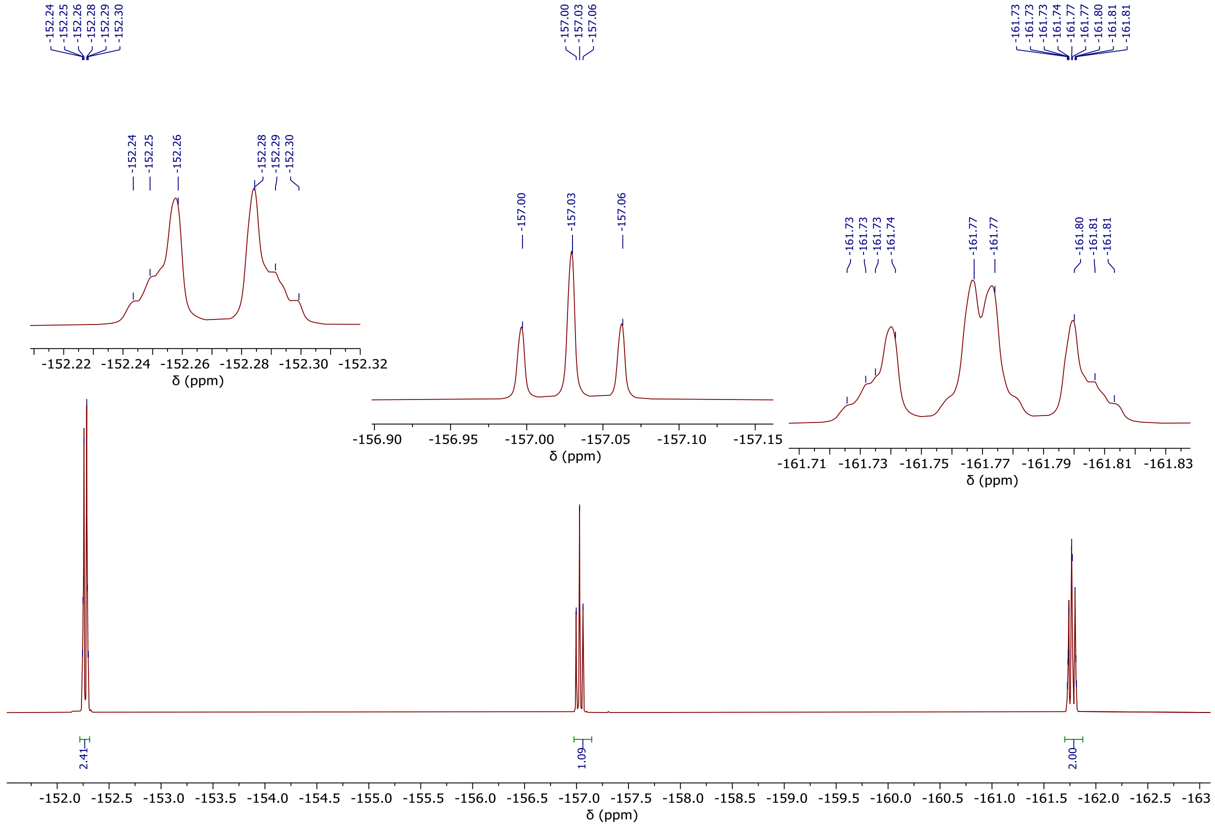

Figure S175: ^{19}F NMR (658 MHz, $CDCl_3$, -163—152 ppm): Spectrum to show **38b**.

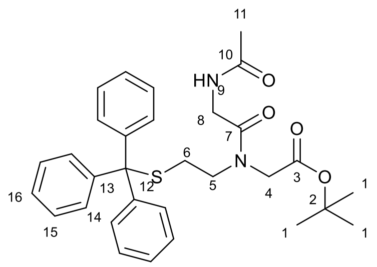

Synthesis of **10a-P1**: To a solution of **38b** (1.68 g, 5.93 mmol, 1 equiv.) and **16_G-P** (2.57 g, 5.93 mmol, 1 equiv.) in anhydrous CH_2Cl_2 (30 mL) was added NEt_3 (1.23 mL, 8.89 mmol, 1.5 equiv.). The resultant solution was then stirred at room temperature. After 16 hours the solution was diluted with CH_2Cl_2 (100 mL) and washed with $NaHCO_3$ (3 × 50 mL, sat. aqueous solution). The aqueous solutions were combined then extracted with CH_2Cl_2 (50 mL), and the combined organics were dried over $MgSO_4$, filtered and concentrated *in vacuo*. The concentrate was recrystallised from Et_2O and further purified by flash column chromatography (SiO_2; CH_2Cl_2/MeOH 100:00 → 90:10) to yield **10a-P1** (2.33g, 4.37 mmol, 74%) as a white powder.

Compound 10a-P1 (Rotamer A & B): ^{1}H NMR (700 MHz, DMSO-d6): δ_H 1.33 & 1.35 (2 × s, 9H, (C1)-H), 1.82 & 184 (2 × s, 3H, (C11)-H), 2.30 & 2.46 (2 × t, *J* = 7.8 Hz, 2H, (C6)-H), 2.96 & 2.99 (2 × app. obs. s, 2H, (C5)-H), 3.53 & 3.83 (2 × s, 2H, (C4)-H), 3.72 & 3.75 (2 × d, *J* = 5.61 Hz, 2H, (C8)-H), 7.25 & 7.25 (m, 3H, (C16)-H), 7.33 (m, 12H, (C14-15)-H), 7.92 & 7.92 (2 × t, *J* = 5.61 Hz, 1H, (C9)-H). **^{13}C NMR (175 MHz, DMSO-d6):** δ_C 22.4 & 22.4 (2 × C11), 27.5 & 27.6 (2 × C1), 28.8 & 29.7 (2 × C6), 40.0 & 40.1 (2 × C8), 46.8 & 47.2 (2 × C5), 48.5 & 49.7 (2 × C4), 66.3 & 66.7 (2 × C12), 80.7 & 81.7 (2 × C2), 126.8 & 126.9 (2 × C16), 128.0, 128.1, 129.1 & 129.2 (2 × C14 & 2 × C15), 144.2 & 144.3 (2 × C13),167.8 & 168.4 (2 × C3), 168.6 & 169.1 (2 × C7), 169.2 & 169.2 (2 × C10). **IR (solid):** 3358.46, 2974.61, 2916.51, 2336.94, 2316.06, 2149.39, 2027.95, 2014.84, 1739.03, 1671.94, 1633.82, 1520.95, 1461.48, 1444.10, 1413.67, 1365.95, 1350.47, 1229.80, 1206.67, 1149.07, 1129.25, 857.19, 741.65, 667.40, 673.99, 666.91, 621.95, 582.66, 470.19, 451.07. **MP:** 158.7-160.5 °C, **HRMS (ESI$^+$):** Calcd. for $[C_{31}H_{36}N_2O_4S+H]^+$ = 533.2469; observed 533.2456.

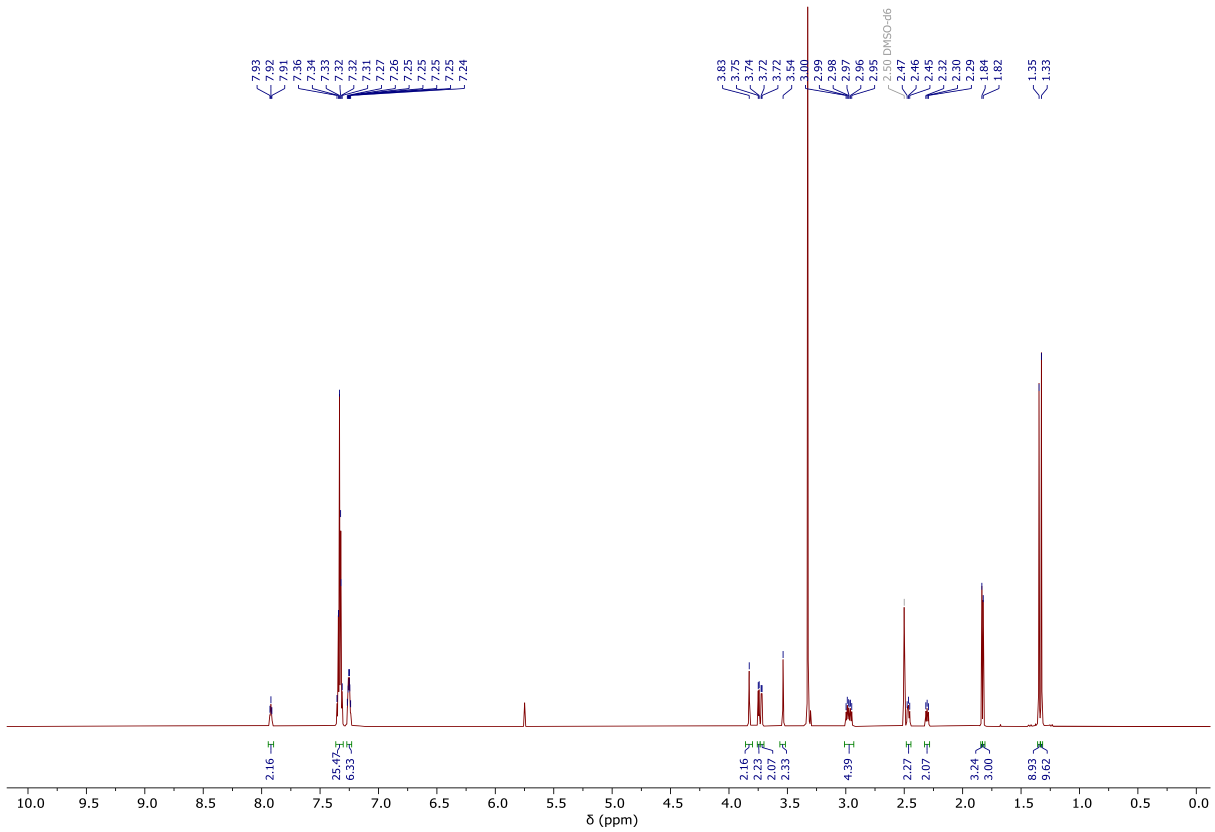

Figure S176: ^{1}H NMR (700 MHz, DMSO-d6, 0.0-10.0): Spectrum to show **10a-P1**.

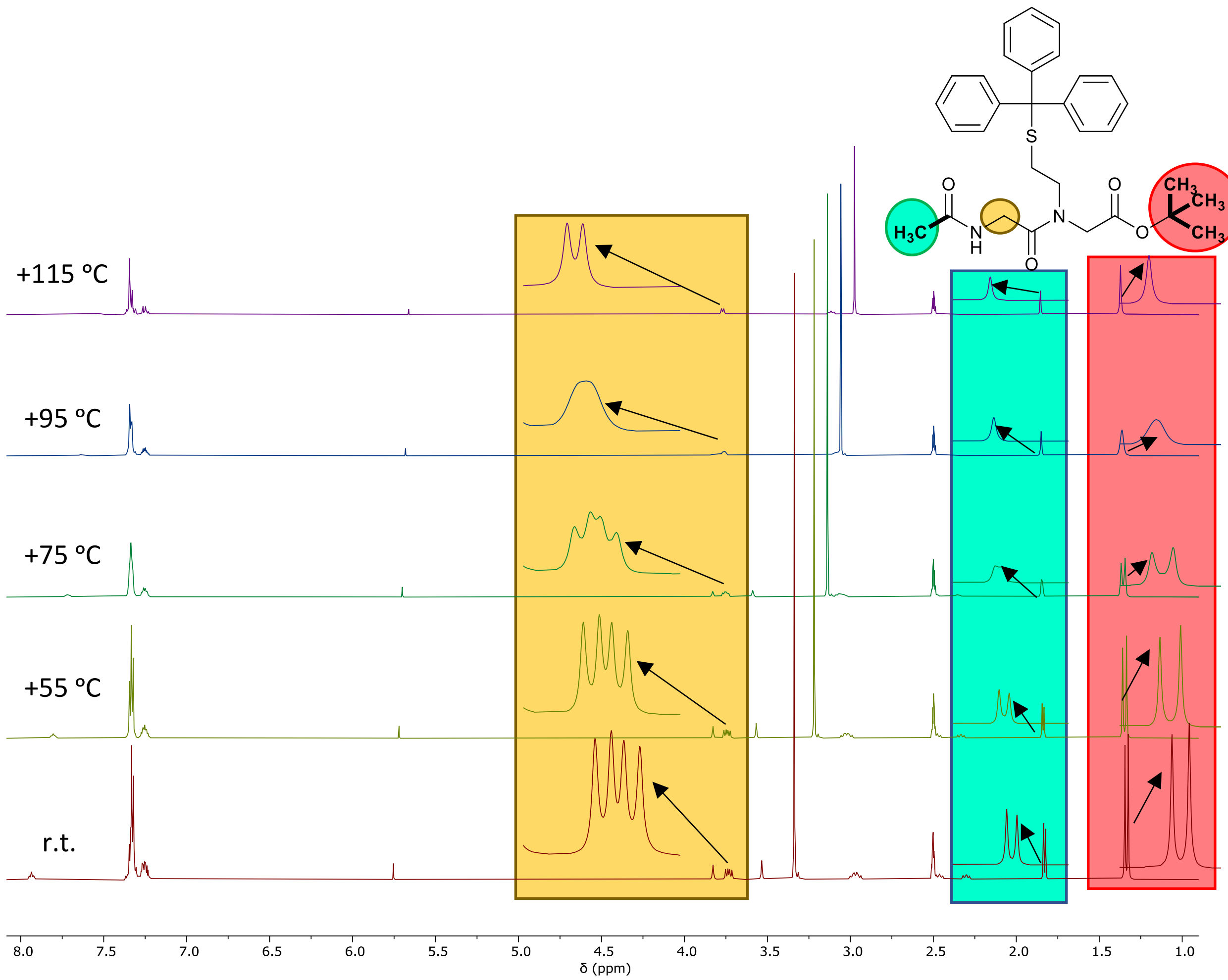

Figure S177: ^{1}H NMR (400 MHz, DMSO-d6, 1.0-8.0 ppm, 19 – 115ºC) spectra to show the temperature dependant coalescence of rotameric NMR resonances of **10a-P1**.

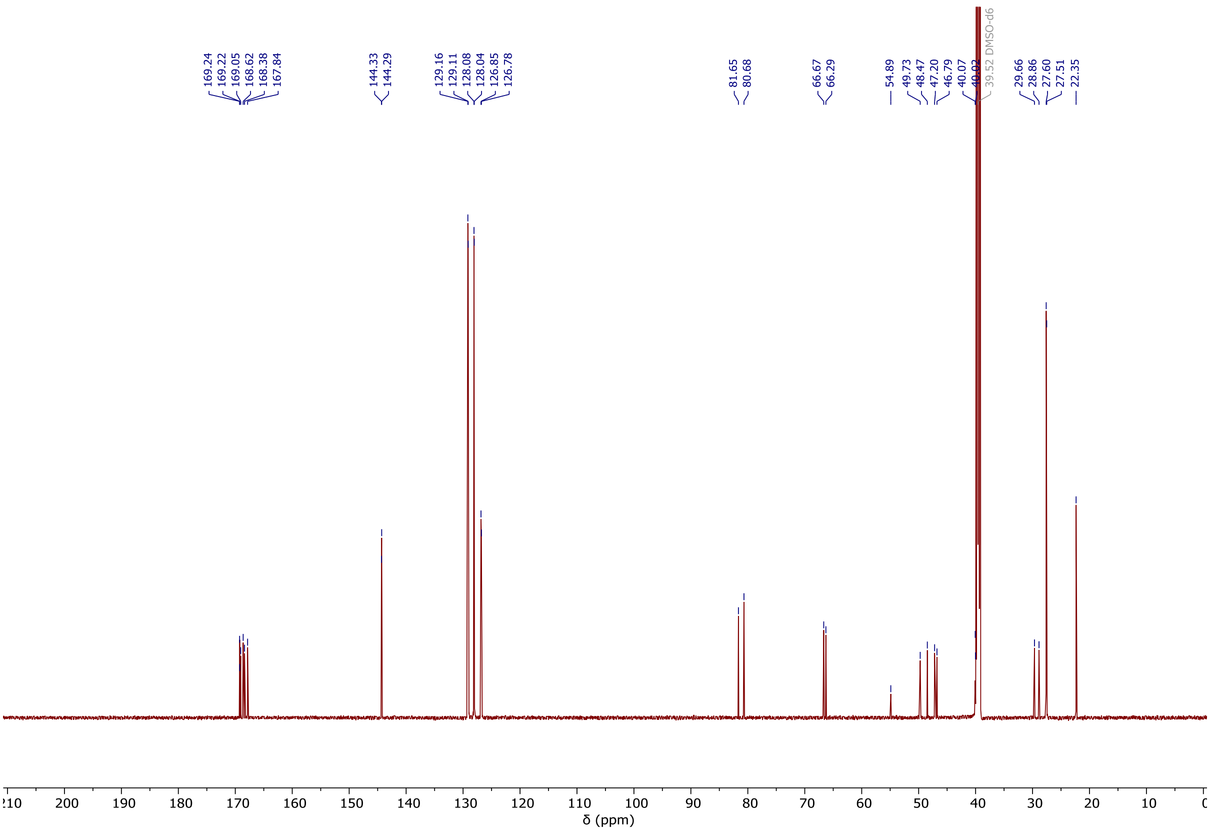

Figure S178: ^{13}C NMR (175 MHz, DMSO-d6, 0.0-210 ppm): Spectrum to show **10a-P1**.

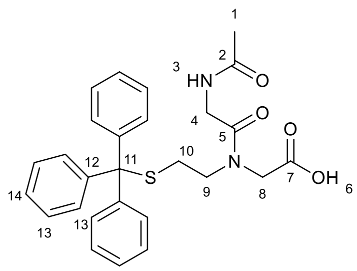

Synthesis of **10a-P2**: To a solution of **10a-P1** (1.69 g, 3.17 mmol, 1 equiv.) in MeOH (11 mL) was added NaOH (3.17 mL, 1 M, 1 equiv.), which immediately formed suspension. Tetrahydrofuran (20 mL) was then added to the suspension and the resultant solution was stirred at room temperature. After 16 hours, the solution was concentrated *in vacuo* and the residue was triturated with Et_2O (5 × 20 mL). The solids were then dissolved in H_2O (40 mL) and the solution was adjusted to pH 4.0 with HCl (1-4 M). The solution was extracted with EtOAc (3 × 70 mL) and the combined organics were dried over $MgSO_4$, filtered and concentrated *in vacuo*. The product was purified by flash column chromatography (SiO_2; CH_2Cl_2/MeOH 100:00 → 80:20) to yield **10a-P2** (1.20 g, 2.52 mmol, 80%) as a white powder. The product was observed to be a mixture of two rotamers (~1:1).

Compound 10a-P2 (Rotamer A & B): ^{1}H NMR (700 MHz, DMSO-d6): δ_H 1.83 & 1.84 (2 × s, 3H, (C1)-H), 2.33 (t, *J* = 7.58 Hz, 2H, (C10)-H) & 2.47 (t, *J* = 7.63 Hz, 2H, (C10)-H), 2.99 & 3.00 (2 × app. t, 2H, (C9)-H), 3.62 & 3.87 (2 × s, 2H, (C8)-H), 3.73 (d, *J* = 4.03 Hz, 2H, (C4)-H) & 3.74 (d, *J* = 5.40 Hz, 2H, (C4)-H), 7.25 (m, 3H, (C14)-H), 7.33 (m, 12H, (C13)-H), 7.70 & 7.90 (2 × app. t, 1H, (C3)-H), 12.55 & 12.81 (br-s, 1H, (C6)-H). **^{13}C NMR (175 MHz, DMSO-d6):** δ_C 22.3 & 22.4 (2 × C1), 29.0 & 30.0 (2 × C10), 39.9 & 40.0 (2 × C4), 46.6 & 46.9 (2 × C9), 47.4 & 48.9 (2 × C8), 66.2 & 66.6 (2 × C11), 126.8 & 126.8 (2 × C14), 128.0, 128.1, 129.1 & 19.1 (4 × C13), 144.3 & 144.4 (2 × C12), 168.6 & 169.0 (2 × C5), 169.2 & 169.2 (2 × C2), 170.2 & 170.7 (2 × C7). **IR (solid):** 3373.97, 3242.99, 3047.11, 1716.59, 1646.05, 1606.95, 1570.08, 1437.92, 1217.83, 1197.74, 1163.03, 1025.75, 840.64, 744.79, 697.58, 669.22, 624.17, 462.37. **MP:** 183.5-187.8 °C, **HRMS (ESI$^+$):** Calcd. for $[C_{27}H_{28}N_2O_4S+H]^+$ = 477.1842; observed 477.1839.

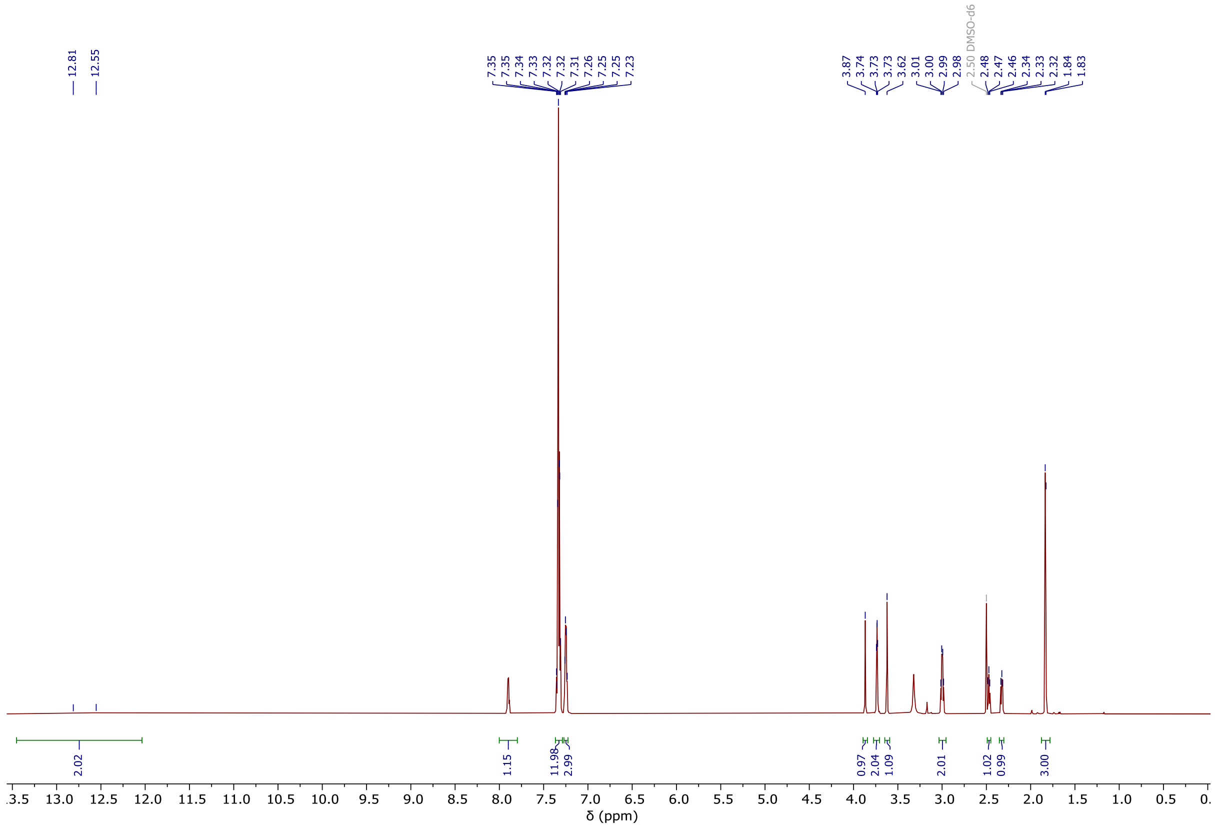

Figure S179: ^{1}H NMR (700 MHz, DMSO-d6, 0.0-13.5 ppm): Spectrum to show **10a-P2**.

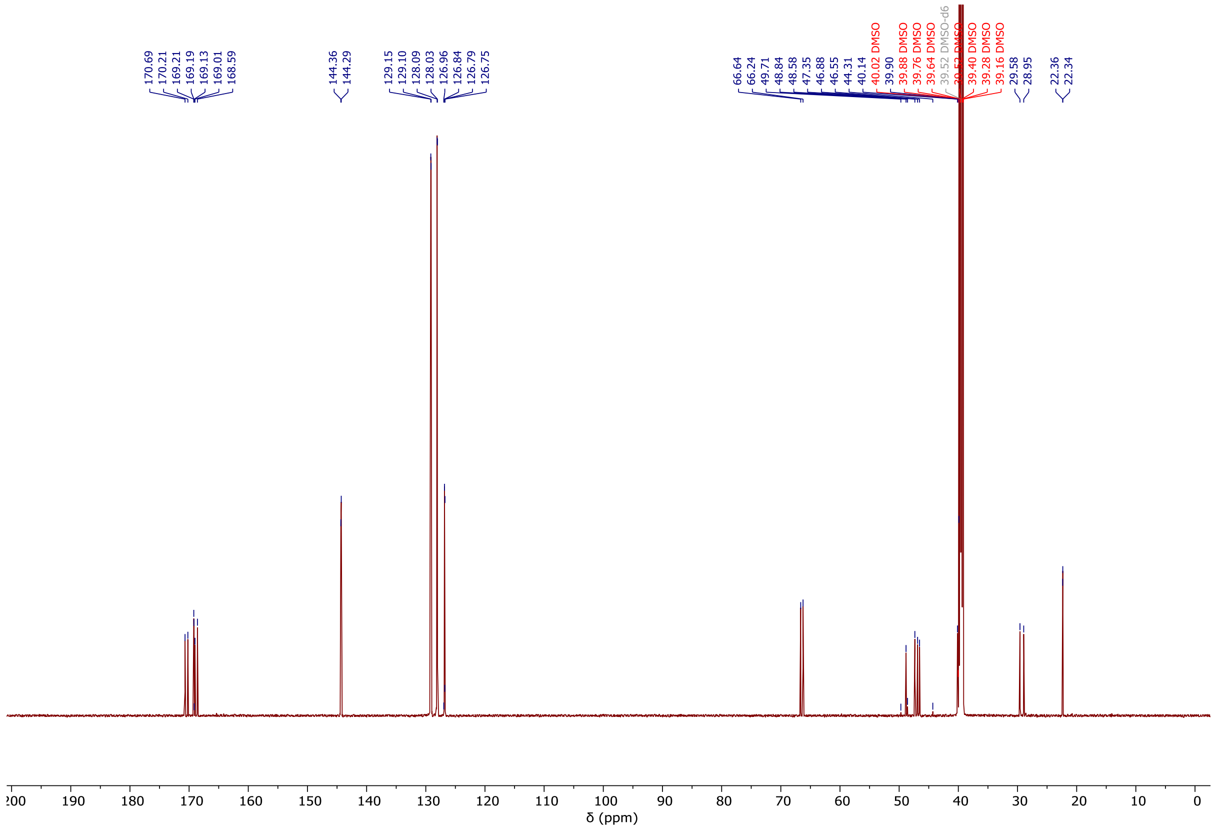

Figure S180: ^{13}C NMR (175 MHz, DMSO-d6, 0.0-200 ppm): Spectrum to show **10a-P2**.

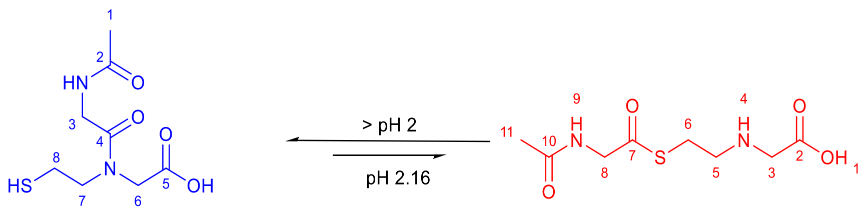

10a **39a**

Synthesis of **10a**: Trifluoroacetic acid (2.60 mL, 21 mmol, 20 equiv.) was added to a solution of **10a-P2** (500 mg, 1.05 mmol, 1 equiv.) and triethylsilane (TES) (0.84 mL, 5.25 mmol, 5 equiv.) in anhydrous CH_2Cl_2 (5 mL). The solution was observed initially to be yellow upon addition of TFA, and then became colourless within 1 minute. The solution was stirred for 10 mins and then concentrated *in vacuo*. The residue was triturated with *n*-hexane (3 × 10 mL) and then purified via flash column chromatography (SiO_2; CH_2Cl_2/MeOH (100:00 → 80:20) to yield **10a** as a viscous, colourless oil (195.2 mg, 0.83 mmol, 79%). The product was dissolved in H_2O (8.33 mL), and an aliquot (1 mL) was diluted with H_2O (775 μL), D_2O (200 μL) and MSM (25 μL, 1 M; internal standard). The resultant aliquot was pH 2.16. After 24 hours at pH 2.2, **10a** (80%) and **39a** (20%) was observed by NMR spectroscopy. The solution (1 mL) was adjusted to pH 7 or to pH 9. After 20 mins, at pH 7 or 9, **10a** (>95%) was observed by NMR spectroscopy. **10a** was observed to form two rotamers, whereas **39a** was not observed to be rotameric (See Supplementary figures 181-183). The product solution (7 mL) was adjusted to pH 7.0 with NaOH (1-3 M) and diluted to 14 mL (H_2O) and then stored at -12 °C as a 50 mM solution.

Compound 39a: ^{1}H NMR (700 MHz, H_2O/D_2O 9:1, pH 2.2): δ_H 2.12 (s, 3H, (C11)-H), 3.29 (m, 2H, (C6)-H), 3.33 (m, 2H, (C5)-H), 3.77 (s, 2H, (C3)-H), 4.21 (app. d, *J* = 6.1 Hz, 2H, (C8)-H). **^{13}C NMR (175 MHz, D_2O, pH 2.2):** δ_C 21.6 (C11), 24.7 (C6), 47.2 (C5), 49.4 (C3), 49.5 (C8), 171.0 (C2), 175.8 (C10), 200.8 (C7).

Compound 10a (Rotamer A & B): ^{1}H NMR (700 MHz, H_2O/D_2O 9:1, pH 7.0): 2 × 2.09 (2 × s, 3H, (C1)-H), 2.73 (app. t, *J* = 7.2 Hz, 2H, (C8)-H & 2.82 (t, *J* = 7.0, 2H, (C8)-H), 3.57 (app. t, *J* = 7.2 Hz, (C7)-H) & 3.60 (t, *J* = 7.0 Hz,2H, (C7)-H), 3.94 & 4.04 (2 × s, 2H, (C6)-H), 4.03 & 4.31 (2 × d, *J* = 5.8 Hz, 2H, (C3)-H).

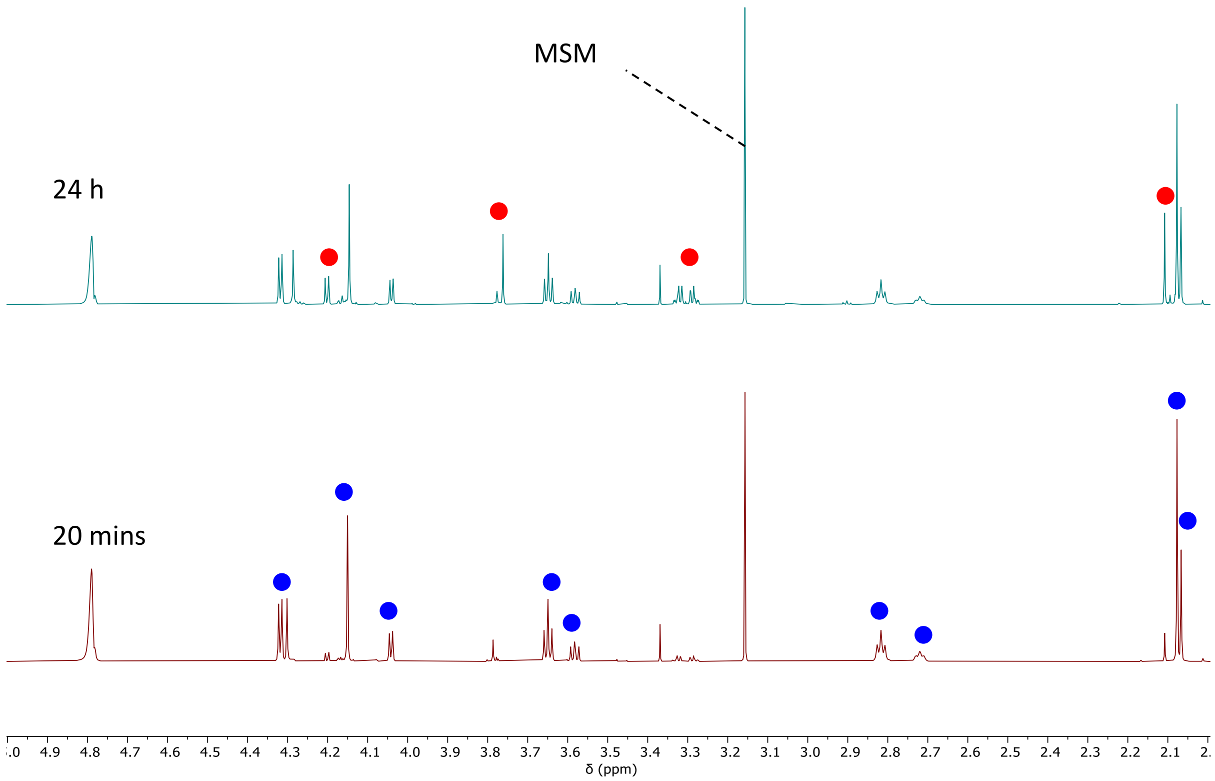

Figure S181: ^{1}H NMR (700 MHz, H_2O/D_2O 9:1, noesygppr1d, 2.0-5.0 ppm) spectra to show the formation of **39a** from **10a** (50 mM) with MSM (25 mM, internal standard) at pH 2.16 and room temperature.

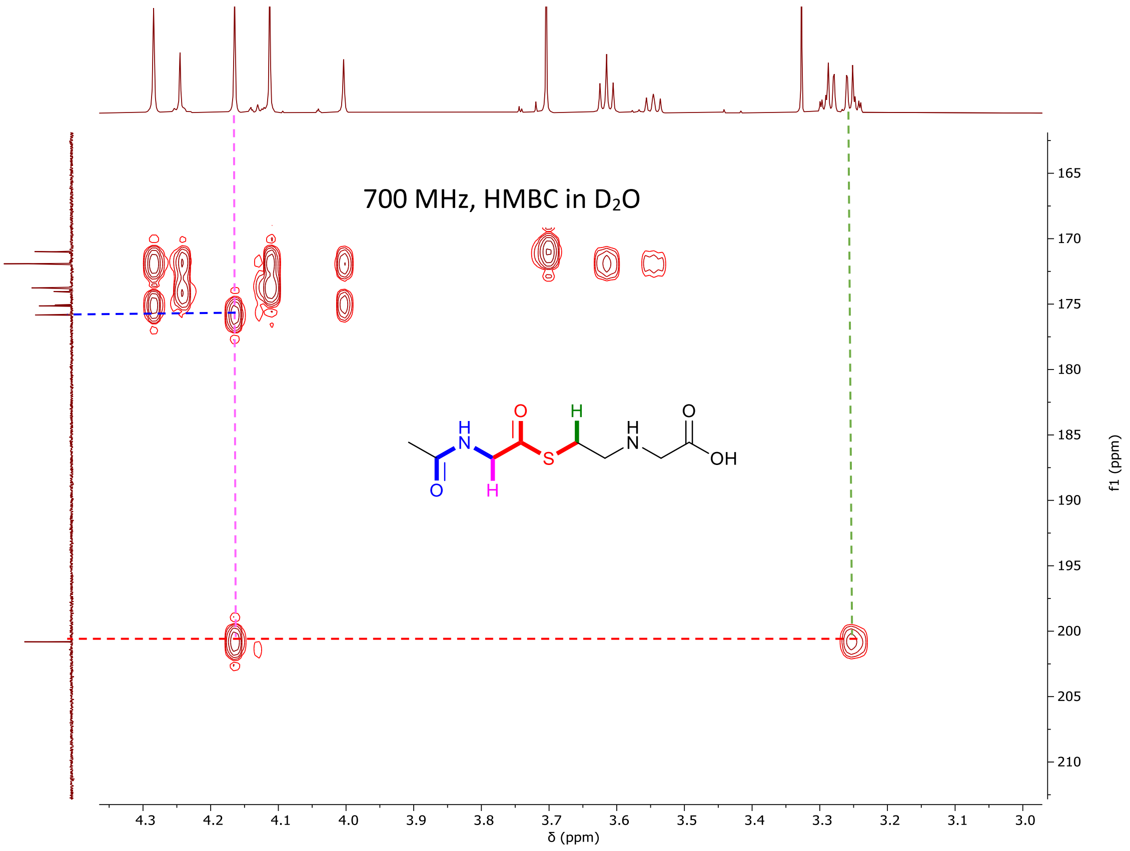

Figure S182: ^1H– ^{13}C HMBC (^1H: 700 MHz [3.0-4.4 ppm], ^{13}C: 175 MHz [160-215 ppm] in D_2O) spectrum to show the diagnostic $^3J_{CH}$ and $^2J_{CH}$ coupling of H_6 and H_8 to a thioester carbonyl carbon (C7) at pH 2.16, that is characteristic of **39a** formation.

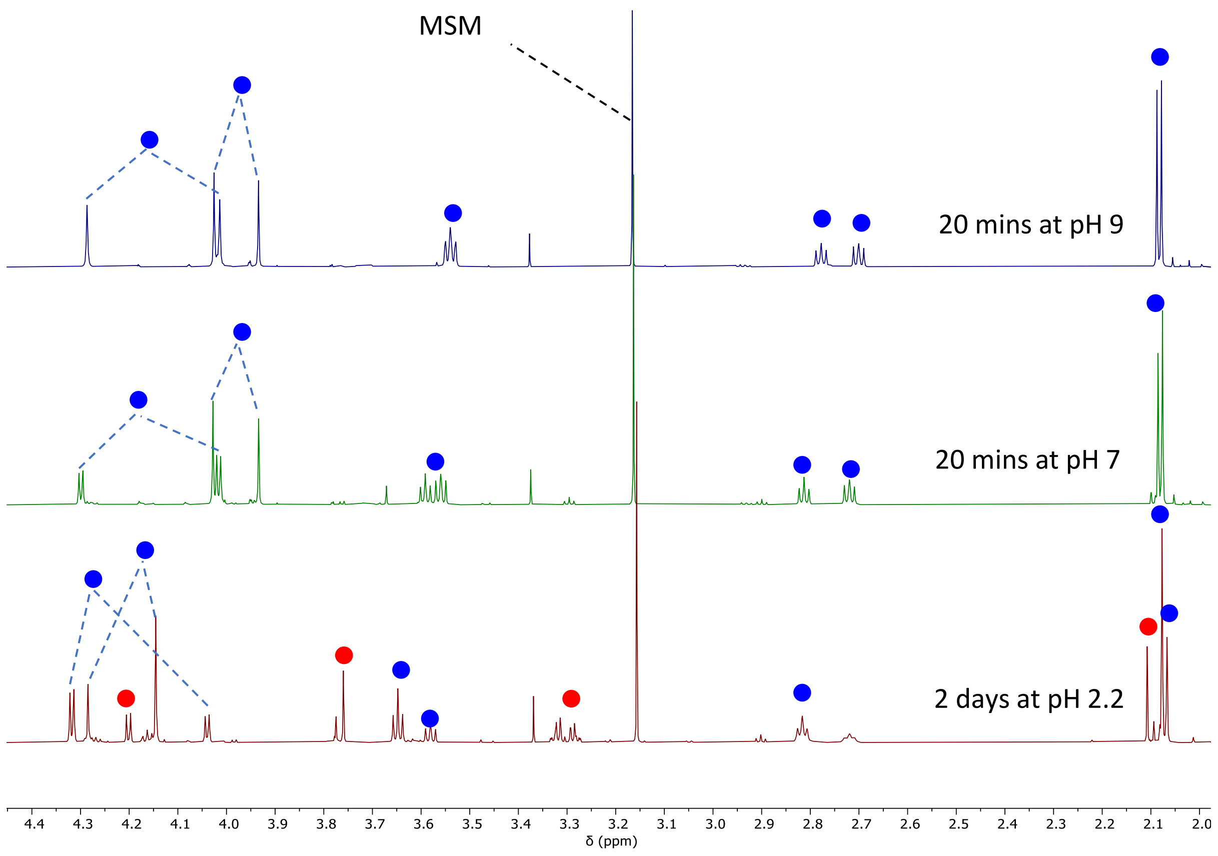

Figure S183: ^{1}H NMR (700 MHz, H_2O/D_2O 9:1, noesygppr1d, 2.0-4.4 ppm) spectra to show the formation of **10a** (middle and top spectra) from a mixture of **10a** and **39a** (50 mM – combined, bottom spectrum) with MSM (25 mM; internal standard) at pH 7 or 9 and room temperature.

.

Synthesis of **19a**:

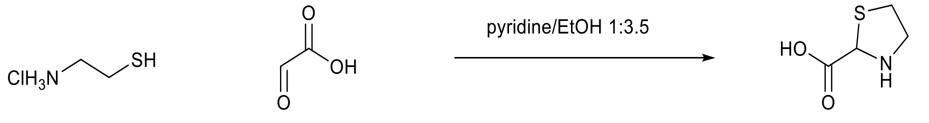

5.HCl **18a** **19a**

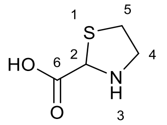

Synthesis of **19a**: To cysteamine hydrochloride **5**·HCl (6.04 g, 53.23 mmol, 1 equiv.) and glyoxylic acid monohydrate **18a** (4.90 g, 53.23 mmol, 1 equiv.) were added pyridine (10 mL) and EtOH (35 mL). The resultant gelatinous suspension was stirred at room temperature for 2 hours. The solids were then isolated by filtration and dissolved in a minimum volume of hot EtOH/H_2O (7:3). The solution was allowed to cool to room temperature and crystallisation was observed. After 16 hours, the solids were isolated by filtration to yield **19a** (4.26 g, 32.0 mmol, 60%) as a crystalline white powder.[5]

Compound 19a: ^{1}H NMR (700 MHz, D_2O): δ_H 5.19 (s, 1 H, (C2)-H), 3.80 (dt, *J* = 11.76 & 6.37 Hz, 1H, (C4)-H), 3.69 (dt, *J* = 11.69 & 6.68 Hz, 1H, (C4)-H), 3.28 (m, 2H, (C5)-H).**^{13}C NMR (175 MHz, D_2O):** δ_C 30.1 (C5), 49.9 (C4), 63.0 (C2), 172.3 (C1). **IR (solid):** 3037.04, 2222.51, 1602.95, 1445.21, 1356.98, 1333.11, 1308.74, 1264.86, 1212.35, 1188.01, 1140.31, 908.55, 873.22, 735.77, 701.50, 607.26, 569.86, 517.69, 431.83. **MP:** 176.1-179.2 °C, Lit: 181-182 °C[3], **HRMS (ESI$^+$):** Calcd. for $[C_4H_7NO_2S+H]^+$ = 134.0270; observed 134.0270.

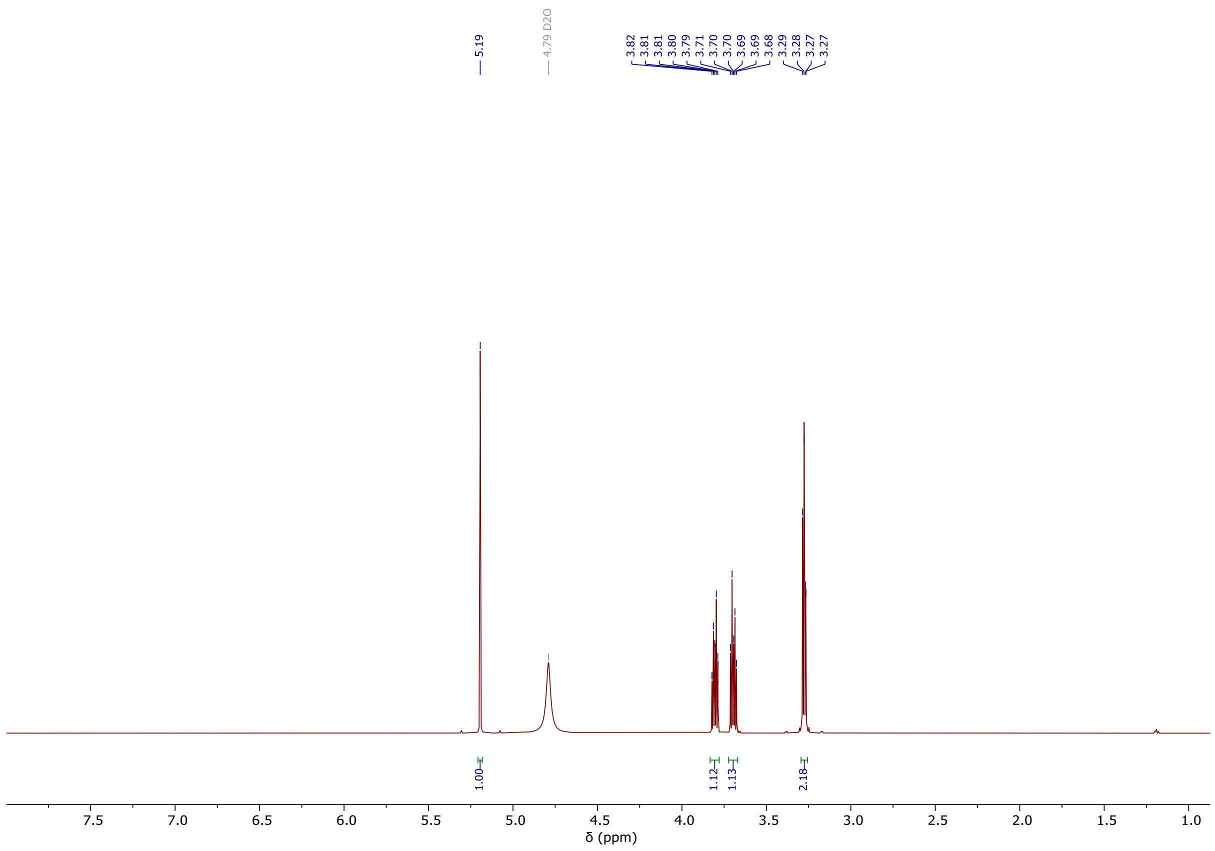

Figure S184: ^{1}H NMR (700 MHz, D_2O, 1.0-8.0 ppm): Spectrum to show **19a**.

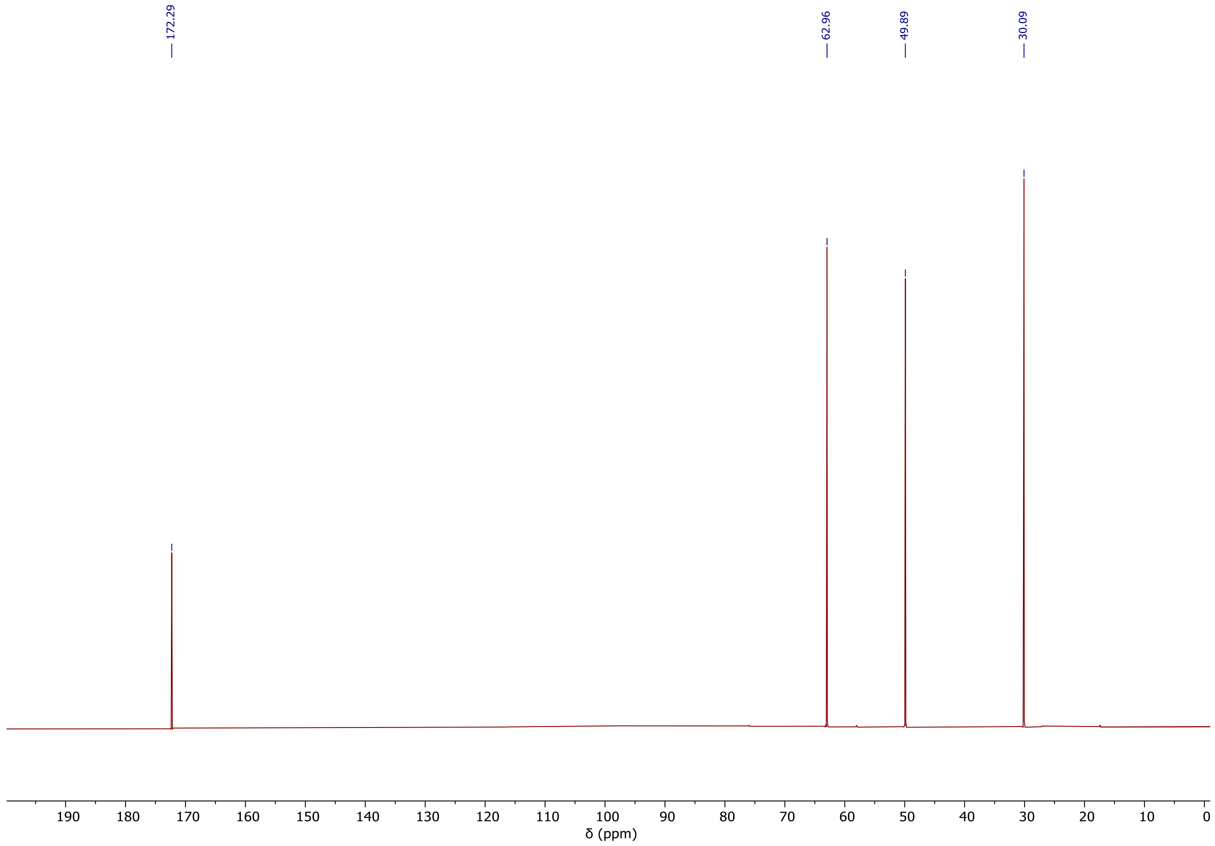

Figure S185: ^{13}C NMR (175 MHz, D_2O, 0.0-200 ppm): Spectrum to show **19a**.

References:

1 S. F. Loibl, Z. Harpaz and O. Seitz, *Angew. Chem - Int. Ed.*, 2015, **54**, 15055–15059.

2 S. F. Loibl, A. Dallmann, K. Hennig, C. Juds and O. Seitz, *Chem. - A Eur. J.*, 2018, **24**, 3623–3633.

3 G. R. Naumiec, G. Lincourt, J. P. Clever, M. A. McGregor, A. Kovoor and B. Deboef, *Org. Biomol. Chem.*, 2015, **13**, 2537–2540.

4 S. DeBonis, D. A. Skoufias, R. Indorato, F. Liger, B. Marquet, C. Laggner, B. Joseph and F. Kozielski, *J. Med. Chem.*, 2008, **51**, 1115–1125.

5 I. Lalezari and E. L. Schwartz, *J. Med. Chem.*, 1988, **31**, 1427–1429.
